# CD33 and clusterin interact biophysically and genetically to modulate Alzheimer risk

**DOI:** 10.1038/s41467-026-75140-3

**Published:** 2026-07-17

**Authors:** Roger B. Dodd, Masahiro Enomoto, Ye Zhou, Kanayo Satoh, Yalun Zhang, Fusheng Chen, Beatrice Acheson, Deniz Ghaffari, Elizabeth Sayn-Wittgenstein, Jamie J. Manning, George V. Dukas, Ronak Patel, Alondra Schweizer Burguete, Mason J. Kralovec, Mamunur Rashid, Jennifer L. Hall, Kirstin A. Tamucci, Zena Chatila, Minghua Liu, Annie J. Lee, Badri N. Vardarajan, Mariko F. Taga, Sirkku Pollari, Alon Rabinovich, Cory D. Rillahan, Andrey A. Bobkov, Eduard Sergienko, William Meadows, Seema Qamar, Suzanne J. Randle, Christopher M. Johnson, Jean Sevalle, Jennifer Griffin, Christopher Bohm, Mitsuhiko Ikura, Xunde Xian, Joachim Herz, Megan A. Kelly, Jennifer West, Sandeep Satapathy, Mark R. Wilson, Jonathan A. Javitch, Paul E. Fraser, David A. Bennett, Philip L. De Jager, Zvi Fishelson, Dan Frenkel, Wesley B. Asher, Elizabeth M. Bradshaw, Peter St George-Hyslop

**Affiliations:** 1 Cambridge Institute for Medical Research, Cambridge, UK; 2 Princess Margaret Cancer Centre, University Health Network, Toronto, ON Canada; 3 Department of Medical Biophysics, Temerty Faculty of Medicine, University of Toronto, Toronto, ON Canada; 4 Department of Medicine (Neurology), Tanz Centre for Research in Neurodegenerative Diseases, Temerty Faculty of Medicine, University of Toronto, Toronto, ON Canada; 5 Department of Medicine (Division of Neurology), University Health Network, Toronto, ON Canada; 6 Department of Psychiatry and Molecular Pharmacology and Therapeutics, Vagelos College of Physicians and Surgeons, Columbia University, New York, NY USA; 7 Division of Molecular Therapeutics, New York State Psychiatric Institute, New York, NY USA; 8 Carol and Gene Ludwig Center for Research on Neurodegeneration, Taub Institute for Research on Alzheimer’s Disease and the Aging Brain, Department of Neurology, Vagelos College of Physicians and Surgeons, Columbia University, New York, NY USA; 9 Division of Translational Neurobiology, Departments of Neurology, Vagelos College of Physicians and Surgeons, Columbia University, New York, NY USA; 10 Center for Translational and Computational Neuroimmunology, Departments of Neurology Columbia University Medical Center, New York, NY USA; 11 Sanford Burnham Prebys Medical Discovery Institute, La Jolla, CA USA; 12 Department of Neurobiology, George S. Wise Faculty of Life Sciences, School of Neurobiology, Biochemistry and Biophysics, Tel Aviv University, Tel Aviv, Israel; 13 Department of Pediatric Oncology, Dana Farber Cancer Institute, and Division of Hematology/Oncology, Boston Children’s Hospital, Boston, MA USA; 14 MRC Laboratory of Molecular Biology, Cambridge, UK; 15 Department of Molecular Genetics, Center for Translational Neurodegeneration Research9, University of Texas Southwestern, Dallas, TX USA; 16 Department of Neuroscience, Neurology and Neurotherapeutics, University of Texas Southwestern, Dallas, TX USA; 17 School of Science, Faculty of Science, Medicine & Health, Molecular Horizons Research Institute, University of Wollongong, Wollongong, NSW Australia; 18 Rush Alzheimer’s Disease Center, Rush University Medical Center, Chicago, IL USA; 19 Department of Cell and Developmental Biology, Faculty of Medicine, and Sagol School of Neuroscience, Tel Aviv University, Tel Aviv, Israel; 20 Yusuf Hamied Department of Chemistry, University of Cambridge, Cambridge, UK

**Keywords:** Alzheimer's disease, Alzheimer's disease

## Abstract

Mechanisms linking CD33 variants to Alzheimer Disease (AD) are poorly defined. Here, we combine structural, cellular, and genetic analyses to delineate how the CD33^M^ splice isoform, upregulated in carriers of CD33 risk alleles, modulates microglial function. We show that CD33^M^ ectodomain dimerizes, enabling binding of large multi-sialylated molecules. We demonstrate that another AD risk protein - clusterin (CLU) ± Aβ oligomers (but not ApoE) binds with nanomolar avidity to CD33^M^, but not CD33^m^. We show that in human monocytes CD33^M^:CLU binding induces CD33^M^ ITIM phosphorylation, recruits SHP-1, suppresses Aβ phagocytosis, and impairs clearance of amyloid plaques. We identify a soluble CD33^M^ ectodomain fragment (sCD33^M^) - absent from CD33^m^-expressing cells - which could contribute to the role of CD33^M^ in AD. Genetic analyses confirm that CD33:CLU interaction modulates amyloid burden, cognition, and disease risk. These findings define a mechanistic CLU:Aβ:CD33^M^ axis, highlighting CD33^M^ dimerization and ligand-binding sites as potential therapeutic targets.

## Abstract (web summary)

Dodd et al. solved the structure of the CD33 receptor, showing that CD33M but not CD33m isoform binds clusterin, but not APOE. Binding triggers CD33 immunoinhibitory signaling, suppresses microglial protective function and increases risk for Alzheimer’s disease.

## Introduction

**Fig. 1:**
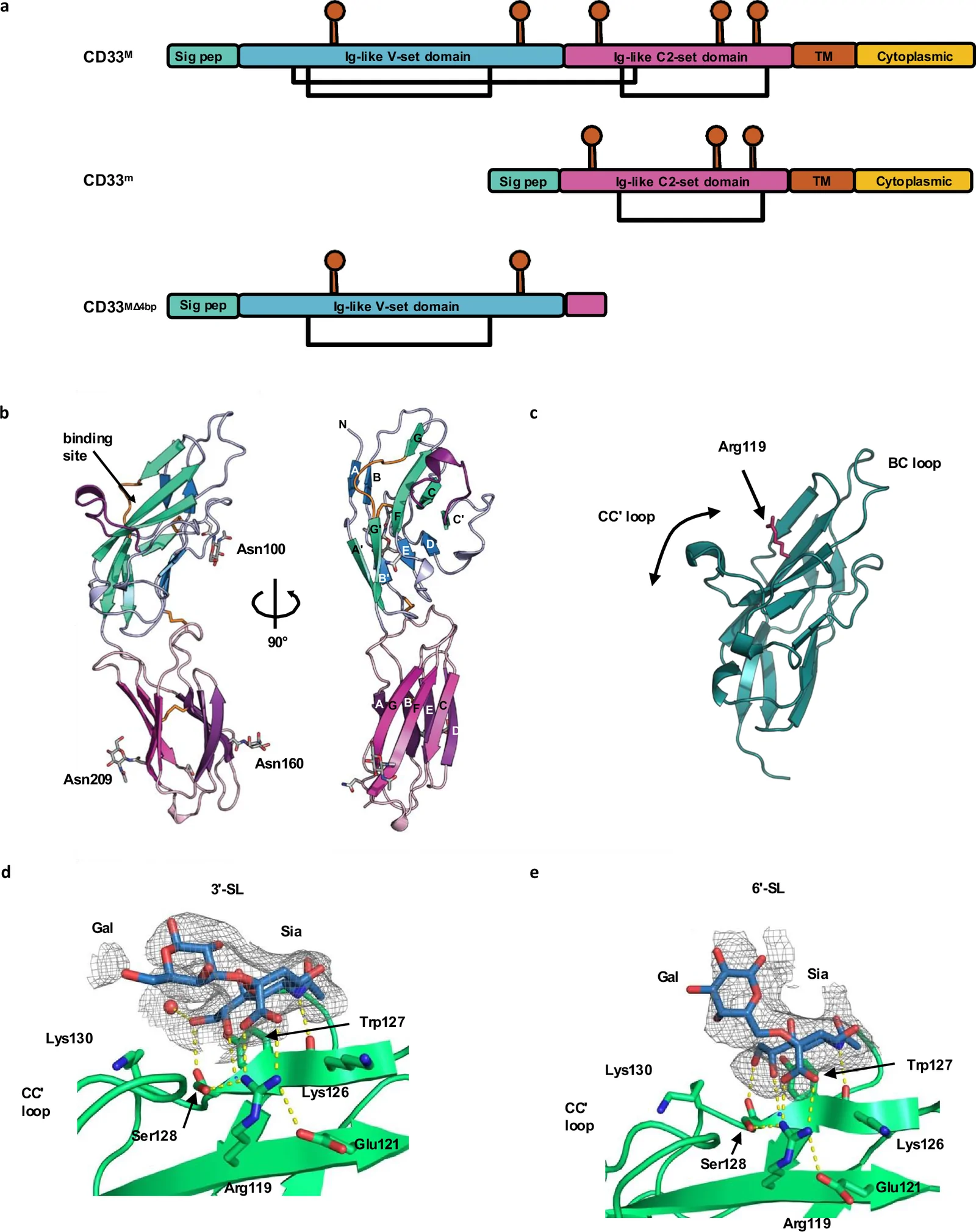
Structural organization of the CD33 extracellular region and ligand-binding features. **a** Domain organization of the full-length human CD33 major isoform (CD33^M^), the alternative splice variant lacking exon 2 (CD33^m^), and the CD33^MΔ4bp^ variant. The signal peptide (green), V-set and C2-set Ig-like extracellular domains (blue and purple), transmembrane region (red), and cytoplasmic tail (yellow) are shown. N-linked glycosylation sites (red lollipops) and conserved disulfide bonds (black lines) are shown. **b** Crystal structure of the CD33^M^ ectodomain is shown in two orientations rotated 90° around the vertical axis. The V-set (green/blue) and C2-set (pink/purple) β-sheets and loops are highlighted, with the CC′ and GG′ loops in purple and orange, respectively. A stabilizing disulfide bond between the BB′ loop and E strand and glycosylations at Asn100, Asn160, and Asn209 are resolved. **c** Structural comparison of the CD33 V-set domain emphasizing the pre-positioned orientation of Arg119 (magenta) and variability in the BC and CC′ loops across Siglec family members. **d**, **e** Bound 3′- and 6′-sialyllactose ligands (carbon atoms = light blue, nitrogen = dark blue, oxygen = red) with omit *mFo-DFc* electron density contoured at 2σ are shown within the canonical sialic acid binding pocket. The electron density maps support inclusion of sialic acid and Gal residues, but insufficient to model Glc. Unlike other Siglecs, the CC′ loop does not directly contribute ligand contacts.

The sialic acid-binding immunoglobulin-like lectin-3 receptor (Siglec-3/CD33) gene encodes a 67-kDa type I transmembrane protein expressed in monocytes and microglia^1,2^. CD33 contains one immunoreceptor tyrosine-based inhibitory motif (ITIM) and one ITIM-like motif in its cytoplasmic tail. Like other Siglecs, it recognizes sialylated ligands presented in cis or trans. Ligand engagement triggers partitioning into lipid-rich raft domains in cell membranes, SRC-kinase-dependent phosphorylation of the ITIM and ITIM-like domains, recruitment of SH2-containing phosphatases (SHP-1/SHP-2), and dephosphorylation of downstream targets such as spleen tyrosine kinase (SYK)^3^. The net effect of this cascade is suppression of microglial activation^3–10^. The importance of CD33 to microglial signaling and function is underscored by the fact that CD33 also interacts with other microglial signaling networks including TREM2^11^ and DAP12/TYROBP^12^. CD33 exists as two isoforms arising from alternative splicing of Exon 2 (Uniprot P20138; Fig. 1a)^13–15^. The full-length CD33^M^ contains a V-set and a C2-set Ig-like domain^16–20^. The V-set domain possesses the sialic acid ligand-binding site encoded by Exon 2. The truncated CD33^m^ (CD33ΔE2) retains the C2-set domain but lacks a binding site.

Genome-wide association studies implicate CD33 as a risk modifier for late-onset Alzheimer’s disease (AD) (meta-analysis OR = 0.94; 95 % CI 0.91–0.96; *p* = 3 × 10⁻⁶)^21–31^. The risk-associated single-nucleotide polymorphism rs3865444 modulates both overall CD33 levels and exon-2 splicing. The major (“C”) allele increases total CD33 expression and favors CD33^M^. The minor (“A”) allele lowers expression and favors CD33^m^^13,14,32,33^.

Functional studies show that C-risk allele carriers have impaired uptake of dextran and Aβ1-42 by monocytes, and elevated brain Aβ by PET imaging^7–11,13,14,32,33^. Intriguingly, the effects on brain Aβ are similar in heterozygous and homozygous C-risk carriers^13^. The relative roles of CD33^M^ and CD33^m^ in AD pathobiology remain debated. Some studies attribute the pathogenic effect to the immuno-inhibitory CD33^M^ isoform, others to the loss of CD33^m^ isoform^7–10,13,14,32^. Models in murine BV2 and human microglial-like cells have been proposed to support both interpretations^7–10,13,14,32^. Consequently, defining the molecular basis of CD33^M^’s dominant inhibitory function and identifying its activating ligands remain key questions.

To address these questions, we solved the crystal structure of the CD33^M^ extracellular domain (ECD). We show that it forms stable dimers with a large double ligand binding pocket. We identified clusterin (CLU) and CLU + Aβ1-42 oligomers, but not ApoE, as ligands. We show that CLU interacts specifically with CD33^M^ dimers, but not CD33^M^ monomers or CD33^m^ dimers, and triggers CD33 ITIM/ITIM-like phosphorylation and CD33^M^ signaling. Activation of CD33^M^ signaling in human monocytes impairs their ability to engulf Aβ and to clear amyloid plaques from ex vivo cortical slices from Amyloid Precursor Protein (APP)-transgenic mice. Analysis of multi-omic human genetic and transcriptional data from the ROSMAP study confirms that genotypes at CLU and CD33 interact to specify risk for clinical AD and related neuropathological phenotypes including severity of Aβ pathology. Collectively, these findings highlight the structural, molecular, cellular, and genetic mechanisms through which genetic variants in CD33 and CLU inhibit protective microglial functions and promote AD risk. Our results also suggest therapeutic strategies and clinical subgroups that might be most suited to CD33-directed therapies.

## Results

### Overall structure of the CD33^M^ ectodomain

We purified and crystallized CD33 ECD proteins by expressing them in HEK293F cells. CD33^m^ did not yield high-quality crystals. Its fold is inferred from CD33^M^. CD33^M^ ECD crystallized orthorhombically with four chains (A–D) showing minimal loop differences (2.24 Å resolution; mean RMSD 1.3 Å; Table 1). Unless noted, chain B is described. The protein adopts a two-domain fold. The N‑terminal V‑set domain (21–139) and C2‑set domain (143–232) are linked by residues 140–142 with a 135° hinge (Fig. 1b, Supplementary Fig. 1A–C). A Cys36–Cys169 disulfide bond stabilizes the linker (Supplementary Fig. 1A). The V‑set β‑sandwich, stabilized by Cys41–Cys101 (Fig. 1a–c), resembles the overall conformations of sialoadhesin^19^, Siglec‑5^20^, and Siglec‑7^16–18^ (Supplementary Fig. 1B). The C2‑set β‑sandwich is stabilized by Cys163–Cys212 (Fig. 1a, b) and differs from Siglec‑5 by lacking a C′ strand and by possessing a D strand, generating a 4‑stranded ABED sheet (Supplementary Fig. 1C). N‑linked GlcNAc densities occur at Asn100, Asn160, and Asn209, with weaker signals at Asn113 and Asn230 (Supplementary Fig. 1D). Asn100, implicated in auto‑inhibition^34^, lies on the same chain as the ligand site but on the opposite face (Fig. 1b).

**Table 1:** X-ray diffraction data for CD33^M^ ECD

	CD33 (unliganded)	Complex with 3’-sialyllactose	Complex with 6’-sialyllactose
Wavelength	0.920 Å	0.920 Å	0.920 Å
Resolution range	62.32–2.24 (2.32–2.24)	62.5–2.66 (2.755–2.66)	42.57–2.48 (2.569–2.48)
Space group	P 21 21 21	P 21 21 21	P 21 21 21
Unit cell	65.0 127.0 143.0	65.1 127.1 142.8	64.5 123.7 141.6
Total reflections	270867 (26337)	147214 (15020)	156800 (15975)
Unique reflections	56518 (5617)	34631 (3420)	39754 (3915)
Multiplicity	4.8 (4.7)	4.3 (4.4)	3.9 (4.1)
Completeness (%)	0.98 (0.99)	1.00 (1.00)	0.97 (0.98)
Mean I/sigma(I)	11.92 (1.90)	11.70 (1.35)	8.05 (1.28)
Wilson B-factor	48.38	58.87	62.56
R-merge	0.0696 (0.785)	0.0902 (0.919)	0.0996 (0.926)
R-meas	0.0782 (0.883)	0.1032 (1.045)	0.1151 (1.068)
CC1/2	0.998 (0.664)	0.997 (0.600)	0.989 (0.638)
CC*	1 (0.893)	0.999 (0.866)	0.997 (0.882)
Reflections used in refinement	56515 (5617)	34626 (3419)	39739 (3912)
Reflections used for R-free	2792 (256)	1707 (150)	1909 (150)
R-work	0.193 (0.292)	0.209 (0.318)	0.229 (0.425)
R-free	0.233 (0.337)	0.244 (0.358)	0.251 (0.473)
CC(work)	0.955 (0.776)	0.945 (0.770)	0.950 (0.703)
CC(free)	0.927 (0.752)	0.896 (0.580)	0.946 (0.651)
Number of non-hydrogen atoms	7039	6960	6941
macromolecules	6639	6673	6636
Ligands	168	251	280
Protein residues	860	851	848
RMS (bonds)	0.005	0.003	0.004
RMS (angles)	0.97	0.64	0.64
Ramachandran favored (%)^a^	98	97	97
Ramachandran allowed (%)^a^	2.1	2.6	3
Ramachandran outliers (%)^a^	0.12	0.12	0.12
Rotamer outliers (%)^a^	0.66	0.53	0.27
Clashscore^a^	1.94	1.03	2.93
Average B-factor	64.33	61.06	79.79
Macromolecules	63.79	60.57	78.08
Ligands	N.A.	75.95	121.86
Solvent	52.74	48.19	63.34
Number of TLS groups	12	14	17

The ECD crystalizes orthorhombically with four chains (A–D) showing minimal loop differences (2.24 Å resolution; mean RMSD 1.3 Å; Table 1). The protein adopts a two-domain fold.

^a^Clashscore, Ramachandran statistics and rotamer outliers were all determined using Molprobity via the PHENIX interface^138^. Source data are available in the Protein Data Bank under accession codes 5IHB (Apo CD33), 5J06 (3-SL complex), and 5J0B (6-SL complex), available at: 10.2210/pdb5IHB/pdb; 10.2210/pdb5J06/pdb; and 10.2210/pdb5J0B/pdb, respectively.

### Structure of the ligand binding site

The structure of the ligand binding site was solved for both unbound CD33^M^ (2.66 Å) and for CD33^M^ with bound 3′‑sialyllactose (3′‑SL) or 6′‑sialyllactose (6′‑SL) (2.48 Å) (Fig. 1c–e; Supplementary Fig. 1D). The ligand-binding site displays canonical Siglec features^16,19,20^ including: (i) an invariant F‑strand arginine (Arg119) whose guanidino group engages ligand sialic acid, and (ii) elements from the G strand, GG′ and CC′ loops (Fig. 1c–e; Supplementary Fig. 1D, E). Our structure agrees with; (i) four other CD33 V-set structures in the Protein Data Bank (PDB 6D48, 6D49, 6D4A and 7AW6; (ii) an unpublished AlphaFold2 model, and (iii) an MD/NMR hybrid (PDB 9BET; BMRB 31167)^35^ (RMSD 0.3–0.4 Å; Supplementary Fig. 1B). Both α2‑3 and α2‑6 linked glucans fit without direct contacts to subterminal sugars (Fig. 1d, e), consistent with promiscuous binding of various sialylated glycans by Siglecs^36,37^. However, the ligand binding site of CD33^M^ displays three unique features (Fig. 1c; Supplementary Fig. 1B). First, Arg119 is pre‑positioned for carboxylate engagement (Fig. 1c; Supplementary Fig. 1E, F), rather than rotating 180° as in Siglec‑5. Second, Lys126 (G strand) and Lys130 (GG′) form a favorable electrostatic surface for binding negatively charged sugars (Fig. 1d, Supplementary Fig. 1B–F). Third, the CC′ loop does not directly interact with ligands, unlike the equivalent loops of other Siglecs (e.g., via Tyr68 on Siglec 5)^20^. The greater flexibility of the CD33^M^ CC′ loop may permit binding of larger sialylated ligands (Fig. 1c–e, Supplementary Fig. 1B–F).

### CD33 forms dimers

**Fig. 2:**
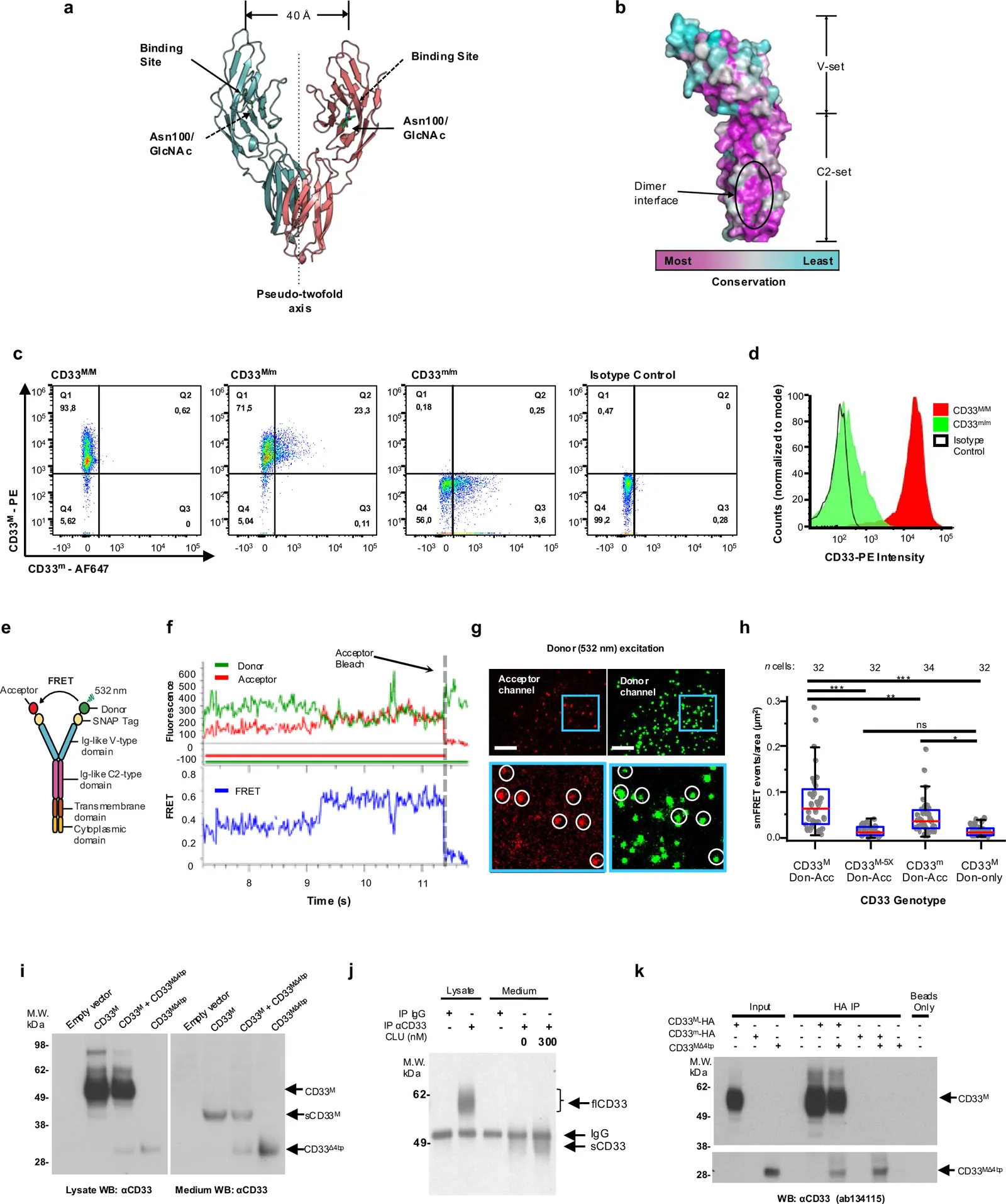
CD33 forms dimers. **a** Cartoon showing dimerization of two CD33^M^ ectodomain chains (teal and pink) mediated by their C2-set domains. The C2-set dimer and the rigid V-set/C2-set junction position the sialic acid-binding sites ~40 Å apart. **b** Surface conservation of the dimer interface (conserved = purple, non-conserved = cyan). **c** Flow cytometry of cells expressing CD33^M^ alone (CD33^M/M^), CD33^m^ alone (CD33^m/m^), or both (CD33^M/m^) demonstrate robust CD33^M^ surface expression in CD33^M/M^ and CD33^M/m^ cells. Most CD33^M/M^ cells are surface CD33^M^-positive. Only a small portion of CD33^M/m^ cells are CD33^m^-positive. Less than half of CD33^m/m^ cells are surface CD33^m^-positive. Representative result from three independent experiments (*n* = 6 biological replicates each). The MFI statistics are in Supplementary Fig. 2J. Source data are provided as a Source data file. **d** Single staining with HIM3-4 confirms higher surface presentation of CD33^M^ in CD33^M/M^ cells (red) than CD33^m^ in CD33^m/m^ cells (green). **e** Cartoon of SNAP_f_-CD33^M^ labeled with donor/acceptor fluorophores for smFRET imaging in live cells. **f** Representative fluorescence intensity time trace (top panel) and corresponding smFRET time trace (bottom panel) for an individual SNAPf-CD33^M^ dimer. The signal was derived during tracking. **g** Representative smFRET image from an image sequence (frame 2, 0.03 s) of labeled SNAPf-CD33^M^ (top panel) excited by the donor laser (532 nm) with the enlarged view (bottom panels) showing sensitized acceptor signals that have colocalizing trajectories with their corresponding donors, both delineated by white circles (Supplementary Fig. 2K for methodology). Four independent biological replications. **h** Distributions of smFRET events per cell area for SNAPf-CD33^M^, -CD33^M-5X^, and -CD33^m^, all labeled with donor and acceptor (Don-Acc) as well as SNAPf-CD33^M^ labeled with only donor (Don-only). Data points represent the total number of freely diffusing smFRET trajectories per area for each cell. Box plots indicate the median (central line) and interquartile range (25th and 75th percentiles). Whiskers represent the points that fall within 1.5 × interquartile range. The indicated total number of cells (*n* cells) were collected over 4 independent experiments for the CD33^M^ and CD33^m^ samples, and 3 independent experiments for the CD33^M-5X^ and CD33^M^ donor-only samples (**g**, **h**). *p < 0.05; **p < 0.01; ***p < 0.001. Ordinary one-way ANOVA with Šídák’s multiple comparisons test. Source data are provided as a Source data file. Exact *p*-values are in Supplementary Table 7. **i** CD33^MΔ4bp^ variant forms a stable soluble protein in cell lysates and is secreted into the media of HEK293 cells expressing CD33^MΔ4bp^. A similar soluble CD33 protein is detected in the medium of HEK293 cells expressing CD33^M^. Representative blot for *n* = 3 independent experiments. Source data are provided as a Source data file. A cartoon of domain structures and epitope tags is in Supplementary Fig. 2M. **j** Western blot analysis of CD33 immunoprecipitated by the CD33 V-set domain antibody from lysates and conditioned medium of iMGs. Notably, sCD33 increases upon stimulation of the iMG cells with 300 nM CLU (lane 5). Three independent biological replications with similar results. Source data are provided as a Source data file. **k** Western blot of lysates and HA-immunoprecipitation products from HEK293 cells expressing CD33^M^-HA, CD33^m^-HA, and CD33^MΔ4bp^. CD33^MΔ4bp^ co-precipitate with CD33^M^ and CD33^m^. These studies do not distinguish whether this interaction occurs in the intracellular compartment or at the cell surface. Representative blots for *n* = 3 independent biological replicates. Source data are provided as a Source data file.

Unexpectedly, the crystal structure reveals that CD33^M^ ECD forms asymmetric dimeric quaternary associations (A:D and B:C) (Fig. 2a). Previous work reports that CD33 forms nanoclusters^3^. The crystal structure provides no mechanism for this, but nanoclusters could arise via engagement of large multivalent ligands that bind several CD33 dimers. The dimer interface for the A:D dimer is created mainly by C and D strands and is conserved (> 60% identity) across 105 Siglec C2 set domains (Supplementary Fig. 1G). The Evolutionary Protein:Protein Interface Calculator predicts a biological interface (Pbio = 1.0)^38^. The dimer interface covers 843 Å² and is stabilized by: (i) main chain H bonds (residues 176–181), (ii) hydrophobic side chain interactions (Ile176, Phe177, Trp179, Leu180, Leu187), and (iii) polar interactions between residues surrounding the hydrophobic core (Arg190A–Ser178D; Gln213D–Gly188A; Lys215D–Arg190A) (Supplementary Fig. 1H–J). The same interface appears in the unpublished PDB 7AW6 CD33^M^ structure (Supplementary Fig. 1B, panel iii). Although dimer asymmetry is unusual, other asymmetric dimers are known (e.g., α catenin^39^; EGFR^40^).

Dimerization of CD33^M^ ECD positions the two ligand binding sites ~40 Å apart, oriented outward (Fig. 2a). This double binding site likely enhances selection of larger ligands and increases binding of weakly interacting partners by avidity effects. To confirm the biological existence of CD33^M^ dimers, we apply four orthogonal approaches. First, Blue‑Native PAGE of 1% digitonin solubilized membranes from human THP‑1 and human blood monocyte cells demonstrates that endogenous CD33 migrates at ~240-kDa (Supplementary Fig. 2A), consistent with two ~75‑kDa glycoproteins plus a ~ 70‑kDa digitonin micelle^13,33^. The poor resolution of Blue Native gels precludes resolution of size differences between CD33^M^/CD33^M^, CD33^M^/CD33^m^, and CD33^m^/CD33^m^ dimers. Second, reciprocal co‑IPs confirm that the expected CD33^M^/CD33^M^, CD33^M^/CD33^m^, and CD33^m^/CD33^m^ dimers co-precipitate in lysates of cells expressing combinations of myc- or HA-tagged CD33^M^ and CD33^m^ (Supplementary Fig. 2B). Third, mutagenesis of selected residues to disrupt the hydrophobic and polar interactions in the dimer interface discloses that two simultaneous mutants (Arg190Glu + Gln213Ala) and four simultaneous mutants (Ile176Ala + Phe177Ala + Ser178Ala + Trp179Ala) do not affect glycosylation and dimerization. However, 5 simultaneous mutants (Phe177Ala, Trp179Ala, Leu180Ala, Arg190Ala, Gln213Ala) (CD33^M‑5X^) disrupt dimerization, but still form stable, glycosylated monomers (Supplementary Fig. 2B), arguing against misfolding as the cause of non-dimerization (Supplementary Figs. 2B and 3C). Fourth, size exclusion chromatography-multi-angle light scattering (SEC-MALS) of Endo H-trimmed CD33 ECD show a mass of 50-kDa by UV (47-kDa RI) at 10 µM and of 44/41-kDa at 2 µM (monomer 25-kDa), indicating predominant CD33 ECD dimers. Parallel studies of glycosylated ECD show a mass of 43-kDa for the protein and 18-kDa for the carbohydrate component at 10 µM, giving the glycosylated protein a particle mass of 61-kDA (Supplementary Fig. 2C). This is consistent with the expected mass of GlcNAc2Man9 glycans synthesized in the presence of kifunensine. Based on these analyses, the estimated Kd of the dimer is ~10⁻⁷ M, indicating that dimerization occurs at physiological concentrations. Analytical ultra-centrifugation sedimentation velocity (AUC-SV) and sedimentation equilibrium (AUC-SE) experiments confirm these results (Kd = 7.1 ± 0.01 × 10⁻⁷ M (deglycosylated) and 3.0 ± 0.1 × 10⁻⁷ M (glycosylated) (*n* = 3; Supplementary Fig. 2D–I). These experiments indicate that dimerization is likely to occur physiologically.

### CD33^M^ is targeted to the cell surface

We generated HEK293 cells expressing equal quantities of total HA-tagged CD33^M/M^, CD33^m/m^, or CD33^M/m^. The HA-tag allows measurement of CD33 abundance across different constructs and cells. While we are aware that over-expression of proteins can cause their mislocalization, prior work shows that CD33^M^ and CD33^m^ are correctly expressed in HEK293 cells^3^. Surface abundance of these proteins in HEK293 cells was assessed by flow cytometry with antibodies specific to CD33^M^ (WM53), CD33^m^ (A16121H), or both (HIM3‑4). Across 6 replicates in 3 experiments (1 × 10⁵ cells/condition), 81.2% ± 9.3% of CD33^M/M^ cells are surface‑positive for CD33^M^. Only 36.1% ± 2.9% of CD33^m/m^ cells are positive for CD33^m^ (Fig. 2c). Furthermore, the intensity of surface CD33^m^ in CD33^m/m^ cells is much lower than CD33^M^ in CD33^M/M^ cells (Fig. 2d, Supplementary Fig. 2J). The surface abundance of CD33^M-5X^ could not be assessed in this experiment because the mutant destroys the recognition motif of the WM53 antibody. Intriguingly, 83.2% ± 6.5% of CD33^M/m^ cells display CD33^M^ at the cell surface, but only 17.7% ± 1.8% co‑express both CD33^M^ and CD33^m^ at the surface (Fig. 2c, Supplementary Fig. 2J). Taken in conjunction with functional data described below, the quantitatively similar preferential surface expression of CD33^M^ in heterozygous and homozygous C-risk allele carriers may explain why brain Aβ levels are similar in heterozygous and homozygous C-risk carriers^13^.

### CD33 receptor dimers imaged at the cell surface by smFRET

To assess dimerization of full-length CD33 receptors in the plasma membrane of mammalian cells, we use single-molecule fluorescence resonance energy transfer (smFRET)^41^. Tetracycline‑regulated Chinese Hamster Ovary lines expressing SNAPf‑tagged CD33^M^, CD33^m^, or CD33^M‑5X^ are labeled stochastically with membrane‑impermeant LD555p (donor) and LD655 (acceptor) dyes and then imaged by total internal reflection fluorescence microscopy (TIRF) (Fig. 2e). Tetracycline concentrations are empirically adjusted to maintain similar cell-surface densities across the CD33 isoforms and 5X mutant (0.34–0.60 molecules/µm², Supplementary Fig. 2K)^41^. Freely diffusing smFRET events per cell area report dimerization (Fig. 2f–h)^41^. CD33^M^ (donor+acceptor) show numerous smFRET events. When expressed at the same level, donor‑only controls and CD33^M‑5X^ show little to no dimerization. In agreement with our flow cytometry data, CD33^m^ produced fewer freely diffusing dimerization events than CD33^M^ but more than donor‑only (Fig. 2g, h; Supplementary Fig. 2K). These differences are not due to differences in time spent in other diffusion states, nor due to differences in cell surface expression, which was equivalent in these experiments. Our results suggest that the dimer site serves as a major interface stabilizing CD33 dimers that is necessary but not sufficient for cell surface presentation. Indeed, as described below, we do not exclude the possibility that other motifs within the N-terminal domain of CD33^M^ not shared with CD33^m^ may also contribute to dimerization and cell-surface trafficking.

### A CD33 frameshift mutant does not dimerize and does not protect against AD

Recent interest has focused on a rare 4-base pair deletion in exon 3 (rs201074739 -“CD33^MΔ4bp^”)^42^. The variant is assumed to be an unstable “null allele” because it putatively encodes a peptide containing residues 1–155 of CD33^M^, comprising the signal peptide and V-set domain but lacking most of the linker and all C2-set, TM, and ITIM domains (Fig. 1a). The variant is of interest because it is not protective against AD (*p* = 0.133; OR 0.90, 95% CI 0.79–1.03, *n* = 21,982 AD and 41,944 controls)^43^. This has been interpreted to support the view that the protective effect of CD33^m^ is not due to simple loss of CD33^M^ (as expected if CD33^MΔ4bp^ is a null allele), but instead reflects an intrinsic protective effect of CD33^m^^43^. To test these assumptions, we express CD33^MΔ4bp^ in HEK293 cells. Contrary to expectations, CD33^MΔ4bp^ protein is present as a stable glycosylated protein in cell lysates and in the media. Even more unexpectedly, although CD33^MΔ4bp^ lacks the C2-set dimer site, it co-precipitates with CD33^M^ or CD33^m^ in membrane fractions of cells co-expressing CD33^MΔ4bp^ and either CD33^M^ or CD33^m^ (Fig. 2k; Supplementary Fig. 2M). Given these results, we ask whether CD33^M^ physiologically forms a soluble secreted fragment by proteolytic shedding at the cell surface similar to APP and TREM2^44–48^. In support of this, we observe: (i) a truncated ectodomain (sCD33^M^) recognized by antibody EPR4423 (residues 30–80)^49^ in the media of HEK293 cells expressing CD33^M^ (Fig. 2i), and (ii) a fuzzy band <49 kDa (likely glycosylated) species in the media of wild-type human iPSC-derived macrophages/microglia (iMGs) expressing endogenous CD33 (Fig. 2j). This species is not observed in conditioned media from cells expressing CD33^m^. The abundance of sCD33^M^ in microglial supernatants is significantly reduced by 5 µM batimastat (Supplementary Fig. 2L), implicating a metalloprotease-dependent shedding mechanism similar to sAPP and sTREM2^44–48^. These experiments raise two exciting new ideas. First, because the shed extracellular domains of CD33^M^ (either CD33^MΔ4bp^ or sCD33^M^) possess intact ligand binding sites, they could form functional chimeric receptors with CD33^M^ (Supplementary Fig. 2M). Second, CD33^MΔ4bp^ and sCD33^M^ might bind to other cell-surface sialylated molecules, potentially altering their signaling activities. In this context, it is noteworthy that sCD33^M^ is exclusively a product of CD33^M^ and not of CD33^m^-expressing cells. Consequently, if sCD33^M^ (and CD33^MΔ4bp^) are functionally active, this difference that may contribute to the differential effects of CD33^M^ and CD33^m^ on AD risk.

### CD33^M^ differentially binds AD-relevant ligands - CLU but not ApoE

**Fig. 3:**
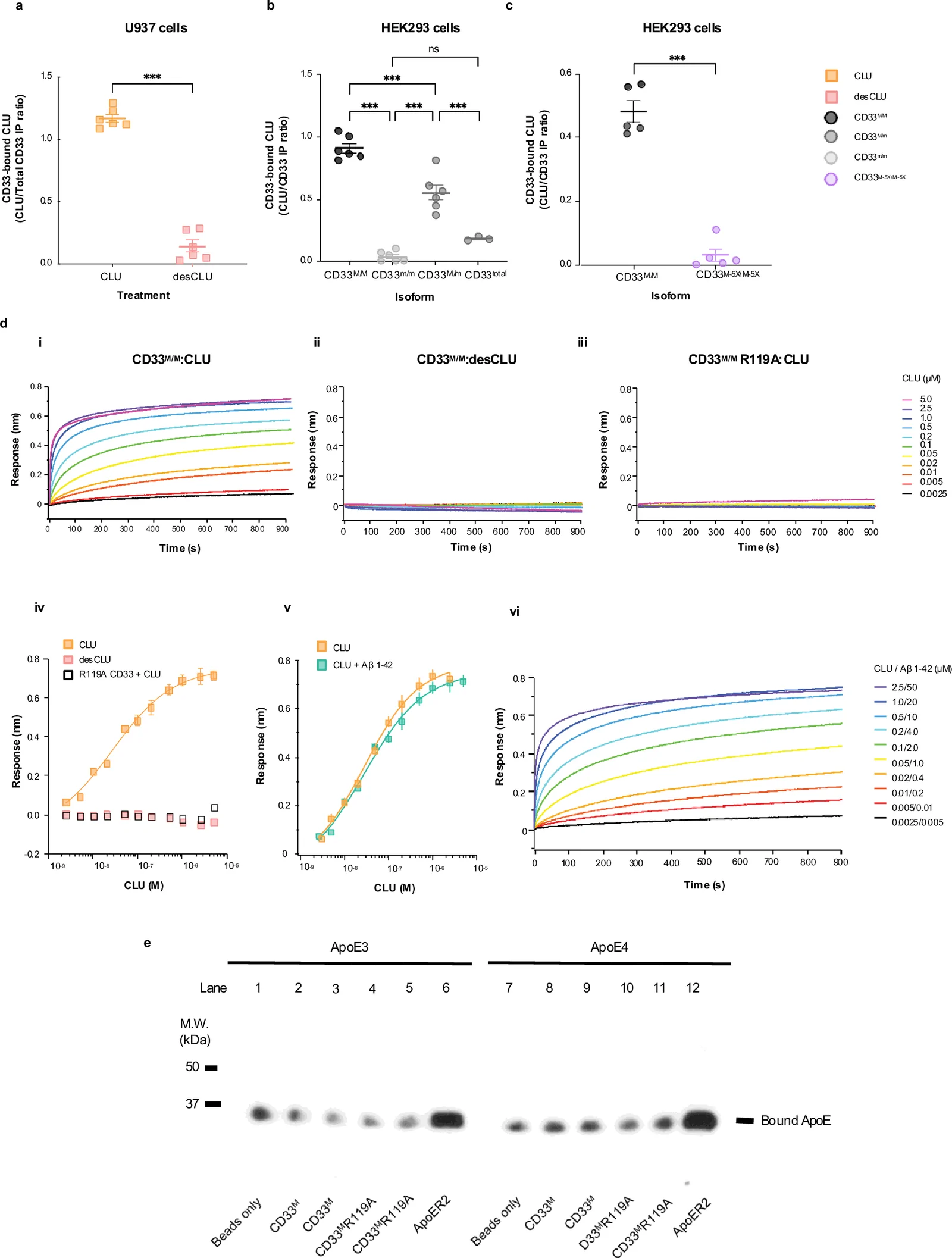
CLU and CD33 interact in vitro and in cells. **a** Endogenous CD33^M^ co-immunoprecipitates native, sialylated CLU, but not desCLU in U937 cells. CD33-bound CLU is expressed as the ratio of CLU to CD33 band intensities in arbitrary units (arb. units). Representative western blot in Supplementary Fig. 3C, left panel. ***p < 0.001; two-tailed Student t-test; error bars = mean ± SEM. CLU: 1.23 ± 0.04; desCLU: 0.23 ± 0.051; *n* = 5 biological replicates. Source data are provided as a Source data file. Exact *p*-values are reported in Supplementary Table 7. **b** CD33^M^ but not CD33^m^ co-immunoprecipitates with CLU. HEK293 cells expressing HA-tagged exogenous CD33^M^, CD33^m^ or CD33^M^ + CD33^m^. CD33-bound CLU is expressed as ratio of co-precipitated CLU to CD33 band intensities (probed with HA antibody) in arbitrary units. CD33^M^:CLU ratio in column 3 and CLU:CD33. Total (CD33^M^ + CD33^m^) ratio in column 4. A representative blot in Supplementary Fig. 3C, right panel. ***p < 0.001; One-way ANOVA with Tukey’s multiple comparisons; error bars = mean ± SEM CD33^M^: 0.85 ± 0.023; CD33^m^: 0.004 ± 0.00043; CD33^M/m^: 0.45 ± 0.041; *n* = 6 biological replications. Source data are provided as a Source data file. Exact *p*-values are reported in Supplementary Table 7. **c** CD33^M-5X^ mutation of the dimer interface significantly reduces CLU co-precipitation in HEK293 cells despite similar protein abundance. A representative western blot is in Supplementary Fig. 3D. ***p < 0.001, Two-tailed, unpaired Student’s t-test, error bars = mean ± SEM, CD33^M^: 0.425 ± 0.0073; CD33^M-5X^: 0.012 ± 0.006; *n* = 5 biological replicates. Each lane represents one independent biological replication. Source data are provided as a Source data file. Exact *p*-values are reported in Supplementary Table 7. **d** CLU binds to CD33^M^ ECD with nanomolar avidity via sialic acids: BLI curves of CLU binding to: (i) CD33^M^ ECD WT, (ii) desialylated-CLU to CD33^M^ ECD WT, and (iii) CLU to CD33^M^ ECD R119A mutant. Each binding curve is the average of four independent measurements. (iv) Steady-state BLI analysis of the CLU binding to CD33^M^ ECD WT (yellow) or CD33^M^ ECD R119A (open) and desialylated-human CLU to CD33^M^ ECD WT (pink). Equilibrium dissociation constant (Kd) is 27.9 ± 7.2 nM. Error bars = SD. *n* = 4 biological replicates; (v) Steady-state BLI analysis of the CLU/Aβ 1-42 mixture (teal) or CLU alone (yellow) binding to CD33^M^ ECD WT. (v) Equilibrium Dissociation Constant (Kd) of CLU/Aβ42 is 28.2 ± 4.9 nM. Error bars = SD. *n* = 4 biological replicates. Curve fitting for (iv, v) was performed using the Hill Equation. (vi) BLI curves of CLU/Aβ 1-42 mixture binding to CD33^M^ ECD WT. Each binding curve is the average of four independent measurements after subtraction by the curve of Aβ 1-42 alone with the same concentration. Error bars = SD. Source data are provided as a Source data file. **e** ApoE does not bind to CD33 but binds ApoER2. Representative western blots for co-immunoprecipitation of ApoE3 or ApoE4 isoforms with His-tagged CD33 or ApoER2. ApoE was detected by immunoblots using an anti-ApoE antibody. Beads only and ApoER2 ectodomain with His-tag are used for negative and positive controls, respectively. *N* = 4 biological replicates. Source data are provided as a Source data file.

CD33 recognizes heavily sialylated glycans^50–52^, including hepatitis B surface antigen and RPTPζS53L^53^. However, its double ligand-binding sites in different orientations suggest potential for binding large sialylated molecules. In companion work (Vo et al.), we show that CD33 binds CD45, a large surface glycoprotein. Here, we examined whether CD33 also recognizes soluble multiply sialylated ligands such as CLU (~45 kDa)^54^) or ApoE (~34-kDa), both of which are products of AD risk genes^14,32,33,55–63^. To address this question, we grew human U937 cells (expressing endogenous CD33) and HEK293 cells (expressing exogenous CD33) in media containing physiological concentrations of CLU (60 nM) or ApoE (3 µg/ml), and immunoprecipitated CD33 from the cell lysates. Native and recombinant sialylated CLU (but not desCLU) co-precipitate with CD33^M^ in U937 cells and with CD33^M^ in HEK293 cells expressing CD33^M^ alone or CD33^M^ and CD33^m^ (Fig. 3a, b). CLU does not co-precipitate CD33^m^ or CD33^M-5X^ (Fig. 3b, c).

To quantify binding avidities, we apply bio-layer interferometry (BLI) using immobilized CD33^M^ ECD (0.5 µM) exposed to CLU (2.5 nM–5 µM). CLU strongly binds sialylated but not desialylated CD33^M^ (Kd = 28.9 ± 10.3 nM) at physiological concentrations (CSF CLU ≈ 60 nM^64^) as confirmed by microscale thermophoresis (Kd = 55.1 ± 2.6 nM; Supplementary Fig. 3G). CD33^M^ R119A does not bind CLU (Fig. 3d). The absence of CLU binding to CD33^M^ R119A is not attributable to misfolding of CD33^M^ R119A because far-UV CD spectra confirm its proper β-sheet folding (~ 215 nm; Supplementary Fig. 3H). BLI experiments were not technically possible for CD33^m^ and CD33^M-5X^ ECDs.

To our surprise, fully sialylated ApoE ε3 and ε4 secreted from HEK293 cells do not bind CD33^M^ although both isoforms bind ApoER2^65^ (Fig. 3e). Why CD33 binds CLU but not ApoE is unclear. It may arise from differences in the number and/or spatial orientation of glycans on CLU and ApoE. Indeed, ApoE undergoes variable and cell-type-specific patterns of O-glycosylation under different activation states^66–68^.

### CD33 interacts with CLU ± Aβ in AD brain

**Fig. 4:**
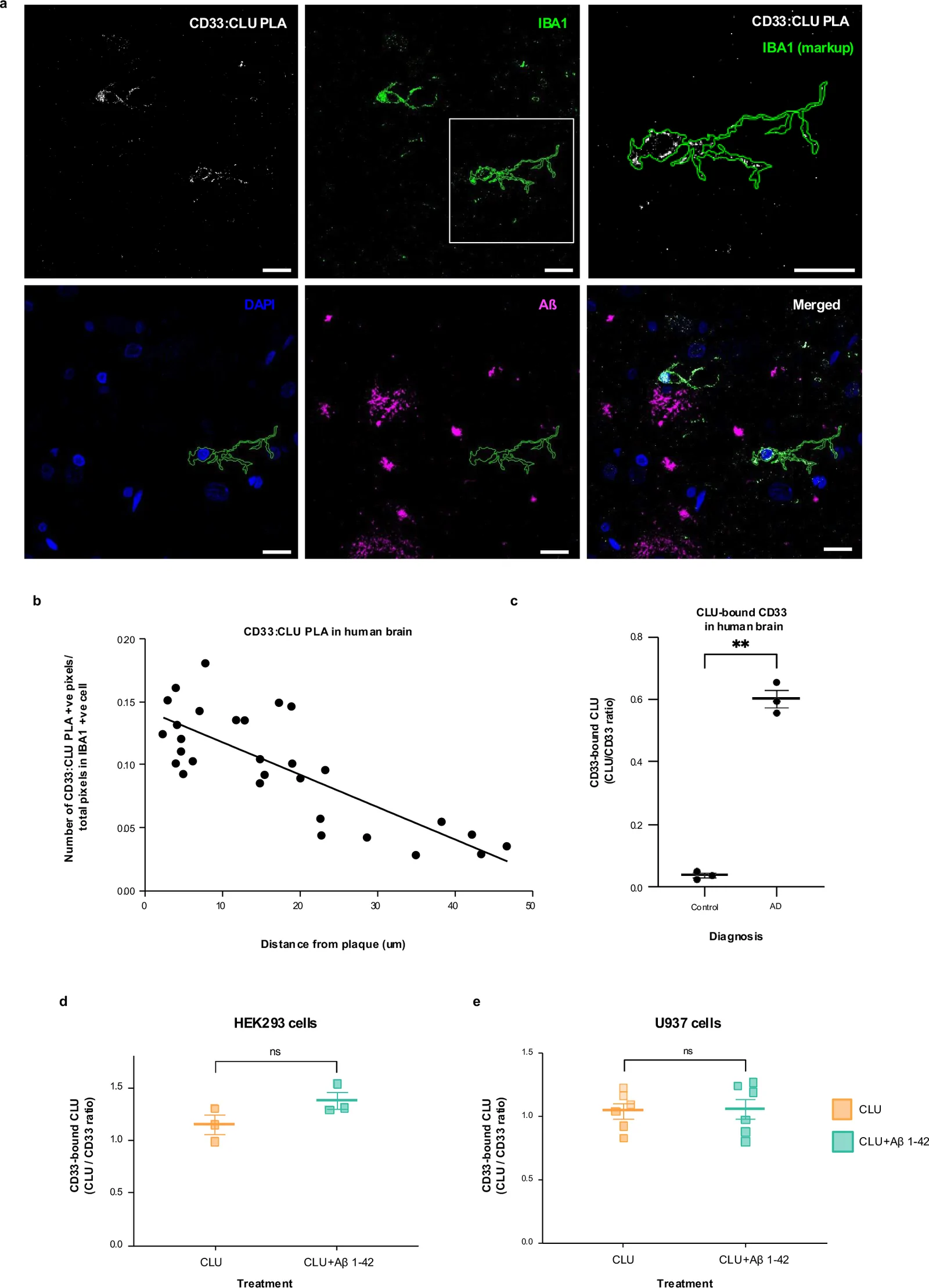
CD33 interacts with CLU and CLU Aβ complexes in human AD brain and cultured cells. **a** PLA demonstrates CD33:CLU complexes in microglia near plaques in human AD brain (63X, scale bar = 50 µm). FFPE sections were stained with 6E10 Aβ = violet; IBA1 microglia = green; PLA = red; DAPI nucleus = blue. Enlarged view of the PLA signal is in the upper right panel. Representative images of 29 microglia from *n* = 3 AD-affected brains. **b** PLA signal intensity negatively correlates with plaque distance from nearest amyloid plaques. The proportion of CD33:CLU positive pixels in each microglia was plotted relative to the distance of that cell from the edge of a plaque (r^2^ = 0.6235; Spearman R = −0.739, *p* < 0.0001, *n* = 29 microglia from *n* = 3 independent AD-affected brains. Source data are provided as a Source data file. Exact *p*-values are r in Supplementary Table 7. **c** AD brain lysates show increased CD33–CLU co-immunoprecipitation compared to controls and *n* = 3 A.D. brains, and *n* = 3 control brains. Representative western blots are in Supplementary Fig. 4A. **p < 0.01; Two-tailed unpaired t-test with Welch’s correction; error bars = mean ± SEM; *n* = 3 independent samples. Brains were selected based on equal expression levels of both CLU and CD33 from a panel of *n* = 53 control and AD cerebral cortices. Source data are provided as a Source data file. Exact *p*-values are reported in Supplementary Table 7. **d** Native human CLU or CLU + Aβ binds exogenous CD33^M^ in HEK293 cells with no significant difference. NS = not significant; Two-tailed unpaired t-test with Welch’s correction; error bars = mean ± SEM, CLU: 1.145 ± 0.0904; CLU + Aβ1-42: 1.379 ± 0.0798; *n* = 3 biological replicates. Each dot represents one independent biological replication of the respective condition. Source data are provided as a Source data file. Exact *p*-values are reported in Supplementary Table 7. **e** Native human CLU or CLU + Aβ binds similarly to endogenous CD33^M^ in human U937 monocytic cells. Representative co-IP western blot is in Supplementary Fig. 4B). NS = not significant; Two-tailed unpaired t-test with Welch’s correction; error bars = mean ± SEM;CLU: 1.042 ± 0.06016, CLU + Aβ1-42:1.056 ± 0.08200; *n* = 6 independent biological replicates. Each dot represents one independent biological replication. Source data are provided as a Source data file. Exact *p*-values are reported in Supplementary Table 7.

Next, to determine whether the CD33^M^:CLU interaction occurs in vivo and its potential relevance to AD, we apply two independent assays. First, we use in situ proximity ligation assays (PLA)^69–72^ on frozen post-mortem Braak Stage 6 AD brain to show that endogenous CLU colocalizes within <40 nm of CD33 on Iba1+ microglia. PLA intensity increases as a function of the proximity of the microglia to Aβ plaques (Fig. 4a, b). In a second, orthogonal approach, to demonstrate in vivo relevance, we use anti-CD33 co-immunoprecipitation assays in lysates from AD cerebral cortex (*n* = 3) and non-AD control cortex (*n* = 3) to re-test an interaction between endogenous CLU and endogenous CD33 in AD brain tissue (Fig. 4c, Supplementary Fig. 4A). Parallel experiments in human HEK293 cells (expressing exogenous CD33^M^) and U937 cells (expressing endogenous CD33^M^) confirm that CD33^M^ and CLU ± Aβ co-precipitate (Fig. 4d, e, Supplementary Fig. 4B).

The increased CD33:CLU interaction observed in AD brain lysates could reflect the increased expression of CLU and CD33 in AD brain and/or enhanced binding of CD33^M^ to CLU:Aβ complexes, which are enriched in AD brain^73,74^. To test the latter possibility, we applied the same BLI method as above to estimate the avidities of CD33^M^ for CLU alone, CLU + Aβ or Aβ alone. CD33^M^ shows high avidity binding to CLU alone and to CLU + Aβ (Kd ≈ 28 nM), but weak binding to Aβ oligomers alone (Fig. 3d v–vi, Supplementary Fig. 3E, F). In summary, these results suggest that under conditions operative in AD-affected brain tissue, CD33^M^ forms high-avidity interactions with both CLU alone and CLU + Aβ oligomers, but not with ApoE.

### CLU activates CD33^M^ ITIM signaling

**Fig. 5:**
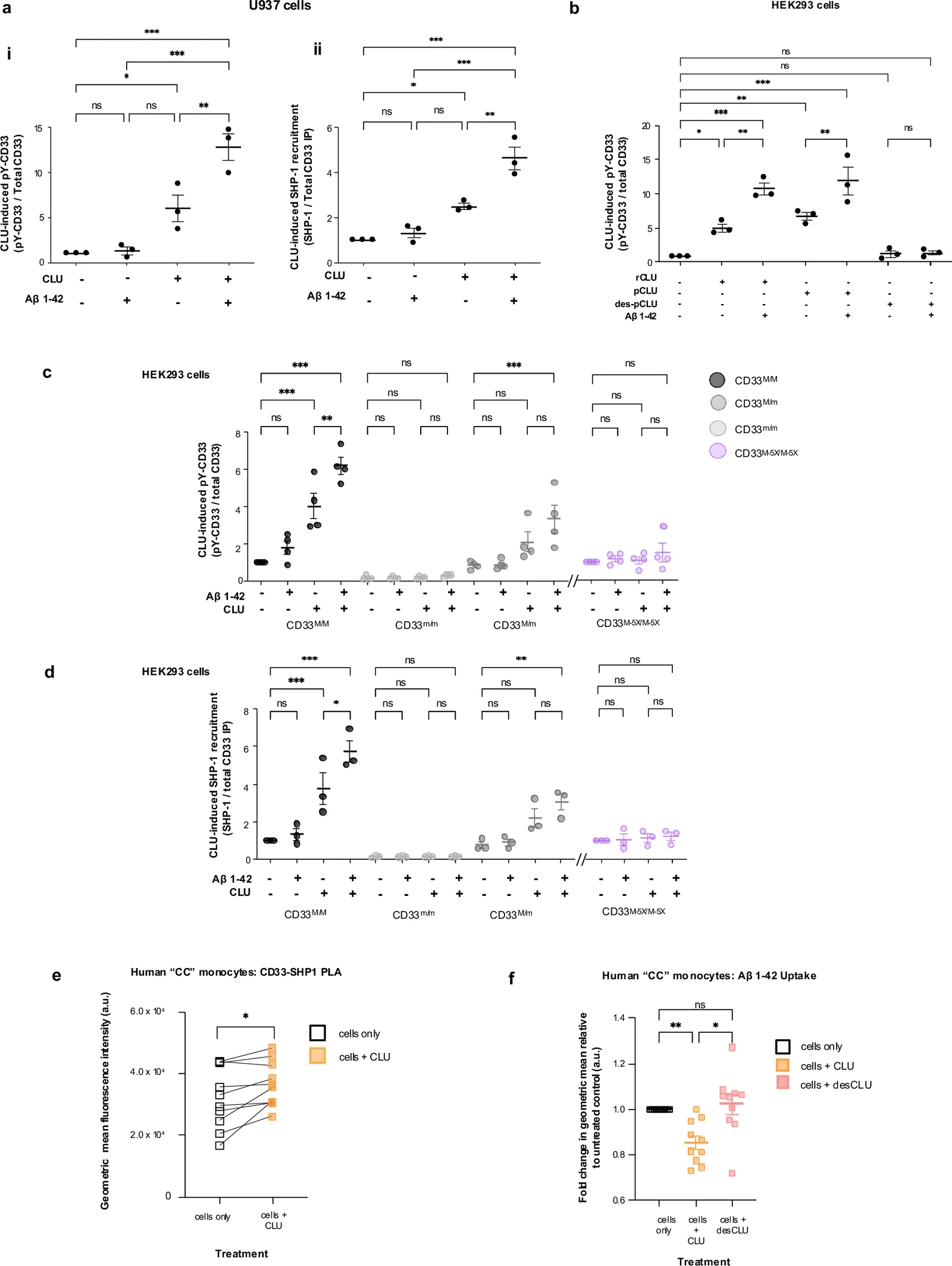
CLU and CLU + Aβ oligomers induce CD33^M^ ITIM phosphorylation and SHP-1 recruitment to CD33^M^/CD33^M^ and CD33^M^/CD33^m^ dimers, but not to CD33^m^/CD33^m^ dimers. **a** Human plasma CLU alone, and human plasma CLU + Aβ oligomers induce ITIM phosphorylation of endogenous CD33 (left panel) and recruitment of endogenous SHP-1 (right panel) in human monocytic U937 cells. Representative western blots are in Supplementary Fig. 5A. *p < 0.05; ***p < 0.001; NS = not significant; One-Way ANOVA with Tukey’s multiple comparisons test; error bars = mean ± SEM; For CD33^M^-pTyr, No treatment: 1.00 ± 0.0; Aβ treatment: 1.367 ± 0.4107; CLU treatment: 6.283 ± 1.667; CLU + Aβ treatment: 11.92 ± 1.116; For SHP-1, No treatment: 1.00 ± 0.0; Aβ treatment: 1.447 ± 0.2726; CLU treatment: 2.589 ± 0.0688; CLU + Aβ treatment: 4.362 ± 0.5971, *n* = 3 independent biological replications. Source data are provided as a Source data file. Exact *p*-values are reported in Supplementary Table 7. **b** Recombinant (rCLU) and native human plasma CLU (pCLU), but not desCLU, activate CD33 phosphorylation in HEK293 cells. Tyrosine phosphorylation is measured by western blot and intensity ratios for pY20-phospho-tyrosine immunoreactive CD33 versus total CD33 by co-IP western blots. Representative western blots are in Supplementary Fig. 5C. error bars = mean ± SEM. *n* = 10 biological replicates. One-way ANOVA with Tukey’s Multiple comparisons test. *p < 0.05; ***p < 0.001; NS = not significant. Source data are provided as a Source data file. Exact *p*-values are reported in Supplementary Table 7. **c** CLU + Aβ induces greater CD33 ITIM phosphorylation than CLU alone, but only in CD33^M^ expressing cells. No CD33 phosphorylation is observed in cells expressing only CD33^m^ and very little in CD33^M-5X^ cells. *n* = 3 replications. error bars = mean ± SEM. Ordinary One-way ANOVA with Sidak’s multiple comparisons test. Representative western blots are in Supplementary Fig. 5D. Source data are provided as a Source data file. *p < 0.05; ***p < 0.001; NS = not significant. Exact *p*-values are reported in Supplementary Table 7. **d** CLU + Aβ induces greater SHP-1 recruitment than CLU alone, but only in CD33^M^ expressing cells, *n* = 3 independent biological replications. Error bars = mean ± SEM. Ordinary One-way ANOVA with Sidak’s multiple comparisons test. Representative blots are in Supplementary Fig. 5D. *p < 0.05; ***p < 0.001; NS = not significant. Exact *p*-values are reported in Supplementary Table 7. **e** Treatment of human monocytes with 60 nM CLU for 15 min at 37 °C increases binding of CD33 and SHP-1. Human monocytes (genotype CD33 rs3865444^CC^) are treated with CLU and then a proximity ligation assay (PLA) is performed to examine CD33-SHP-1 interaction. Median fluorescence intensity of the PLA of CD33-SHP-1 is measured and analyzed in relation to untreated monocytes from the same individuals. Two-tailed paired t-test, *p < 0.05. Error bars = mean ± SEM. Representative histogram is in Supplementary Fig. 5E. *n* = 10 biological replicates. Exact *p*-values are reported in Supplementary Table 7. **f** CD33 rs3865444^CC^ risk genotype-dependent effect of CLU on monomeric Aβ1-42 uptake by human monocytes. Human monocytes (genotype CD33 rs3865444^CC^) are treated with either CLU or desCLU, and the effect on Aβ1-42 uptake is assessed by flow cytometry. CLU significantly reduces the Aβ 1-42 uptake compared to the media control or desCLU treatment. Data are analyzed using geometric mean intensity of HiLyte™ Fluor 647 conjugated Aβ 1-42, *n* = 10 unrelated donors each examined once. Each dot represents an individual donor. Error bars = mean ± SEM. Data were analyzed using Repeated Measures One-Way ANOVA with Tukey’s multiple comparisons test. *p < 0.05; **p < 0.01. Source data are provided as a Source data file. Exact *p*-values are reported in Supplementary Table 7.

These results raise the question of whether CLU ± Aβ oligomers activate CD33^M^ ITIM signaling. Previous work using sodium orthovanadate or activating antibodies indirectly imply that ligand binding to CD33^M^ activates CD33 signaling via: (i) phosphorylation of tyrosine residues in the intracellular ITIM domains (e.g., Tyr340)^75–77^, (ii) recruitment of the Src-homology 2 domain (SH2)-containing protein tyrosine phosphatase 1 (SHP-1), and (iii) inhibition of SYK phosphorylation that regulates the well-documented signaling cascade that mediates the organization of the actin cytoskeleton (for motility and phagocytosis) via the WAVE regulatory complex and ARP2/3 complex^75,76,78–81^. Here, we directly prove that application of 60 nM CLU ± Aβ1–42, but not Aβ1–42 alone, induce rapid (5–15 min) CD33 ITIM tyrosine phosphorylation and SHP-1 recruitment (Fig. 5a (i,ii); Supplementary Fig. 5A). Recombinant (rCLU) and plasma (pCLU) clusterin equivalently activated CD33^M^ ITIM signaling, whereas desCLU is inactive (Fig. 5b, Supplementary Fig. 5B). CD33^m^-only and CD33^M-5X^ cells do not respond to CLU ± Aβ1-42 (Fig. 5c, d, Supplementary Fig. 5C, D). In cells expressing CD33^M^ (either CD33^M/M^ or CD33^M/m^), ITIM signaling is robustly activated by CLU ± Aβ1-42 (Fig. 5b–d). Crucially, in CD33^M/m^ cells, only CD33^M^ is phosphorylated, consistent with inefficient CD33^m^ presentation to the cell surface (Figs. 2g, h and 5c) but with sufficient CD33^M^ homodimers forming at the cell surface for signaling (Fig. 2c, d). We replicate these results in human monocytes and MDMi cells using flow-PLA^82^, western blotting and flow cytometry to show that 60 nM CLU triggers rapid CD33 ITIM phosphorylation and SHP-1 recruitment (Fig. 5e, Supplementary Fig. 5E–G). Furthermore, although CLU and CLU + Aβ1-42 have similar CD33 binding avidities (Fig. 3d, panels v–vi; Supplementary Fig. 3E–G), CLU + Aβ1-42 induces much stronger CD33 ITIM signaling than CLU alone (Fig. 5a–d; *p* < 0.05–0.001). The underlying structural mechanism is unclear. One possibility is that CLU-bound Aβ1-42 oligomers reshape the complex to better fit the CD33^M^ binding pocket and generate more robust allosteric effects on the CD33 ITIM domains.

### CLU but not desCLU modulates monocytic Aβ engulfment

To assess the functional impact of activation of CD33 ITIM signaling by CLU, we use an ex vivo Aβ uptake assay^13^. We exploit the fact that CLU efficiently binds Aβ oligomers but not monomers^73,83^, permitting deconvolution of monomeric Aβ uptake as phagocytic cargo versus Aβ oligomers as a co-ligand with CLU for CD33 receptors. Monocytes from “CC” risk carriers, who express more CD33^M^, exhibit reduced monomeric Aβ uptake compared to “AA” protective donors^13^. Treating “CC” monocytes with 60 nM CLU further inhibits monomeric Aβ uptake (Fig. 5f), while desCLU has no effect.

### CLU inhibits monocyte-mediated clearance of amyloid plaques in AD brain tissue

**Fig. 6:**
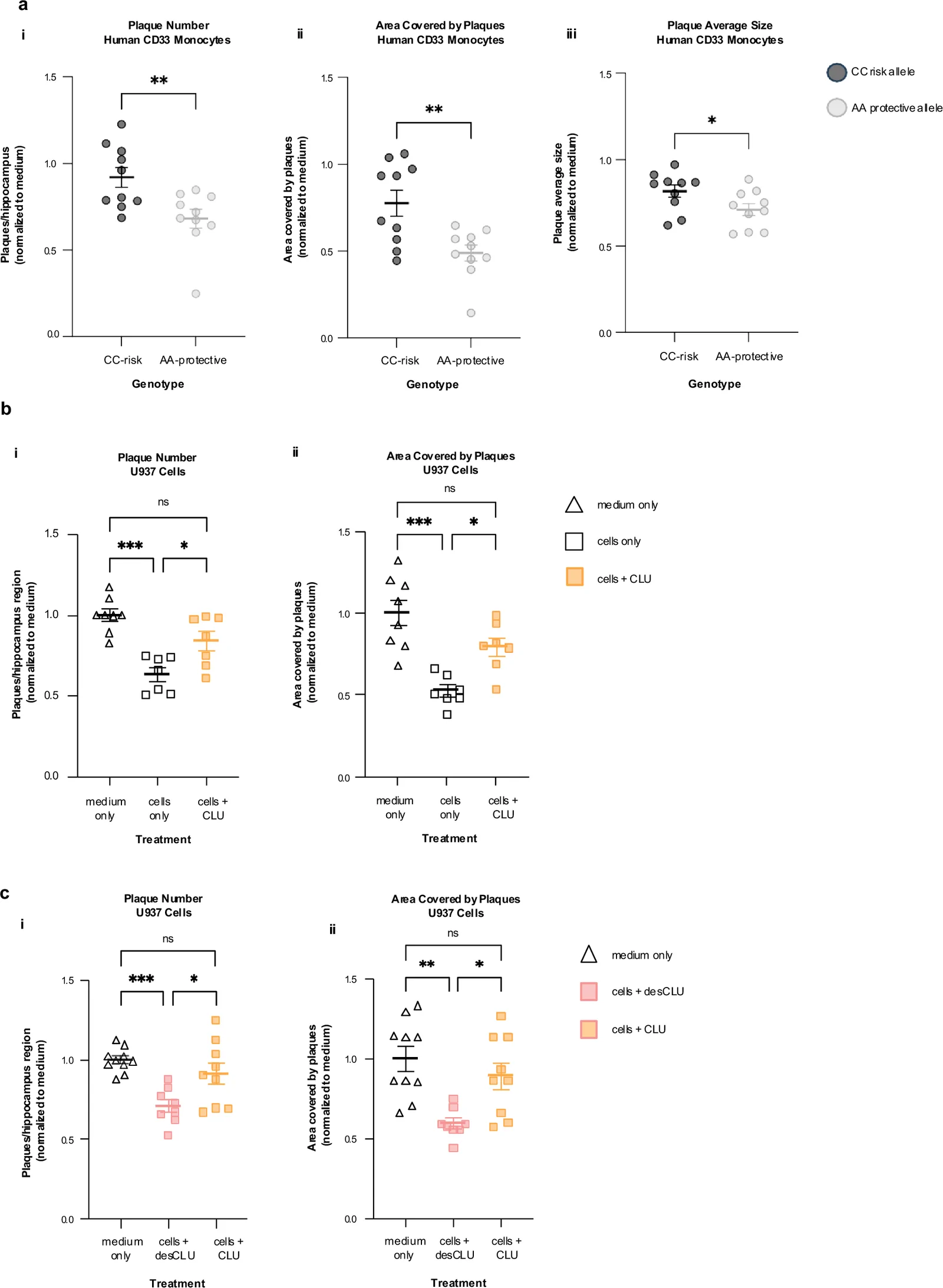
CLU downregulates CD33-mediated clearance of amyloid plaques by human monocytic cells. **a** Monocytes from CD33 “CC” risk allele carriers show reduced plaque clearance in ex vivo 5XFAD brain sections compared to “AA” protective allele carriers. The results are expressed as plaque number (i), brain area covered by plaques (ii), and plaque size (iii). The results are normalized to the results for medium only. *n* = 10 independent replications. ***p < 0.001, *p < 0.05; two-tailed unpaired student’s t-test; error bars = mean ± SEM. Randomly chosen representative images are in Supplementary Fig. 6A. Source data are provided as a Source data file. Exact *p*-values are reported in Supplementary Table 7. **b** In human U937 monocytic cells, CLU impairs plaque clearance in the same assay. Results are expressed as number of plaques (i) or area covered by Aβ (ii) and are normalized to the result for slices exposed to medium only. *p < 0.05, **p < 0.01, ***p < 0.001; One-Way ANOVA with Tukey’s multiple comparisons test; error bars = mean ± SEM, *n* = 8 replications. Representative images are in Supplementary Fig. 6B. Source data are provided as a Source data file. Exact *p*-values are reported in Supplementary Table 7. **c** Desialylated CLU does not impair clearance, confirming that suppression requires sialic-acid-dependent CD33^M^ engagement (*p < 0.05 to ***p < 0.001; *n* = 10). Results are expressed as (i) number of plaques, or (ii) area covered by Aβ. Representative images are in Supplementary Fig. 6C. *p < 0.05, **p < 0.01, ***p < 0.001, one-way ANOVA with Tukey’s multiple comparisons test; error bars = mean ± SEM, *n* = 10 replications. Source data are provided as a Source data file. Exact *p*-values are reported in Supplementary Table 7.

To place these observations in a disease-relevant context, we exploit the previous observation that human monocytes clear amyloid plaques from ex vivo brain slices from the 5XFAD mouse model of AD^84^. Monocytes from “AA” protective carriers clear plaques more effectively than monocytes from “CC” risk carriers (Fig. 6a, Supplementary Fig. 6A, *p* < 0.001). We observe that human U937 monocytic cells (expressing CD33^M^ and mimicking native human monocytes) reduce amyloid plaque pathology compared to medium only (Fig. 6b, Supplementary Fig. 6B, *p* < 0.0001). However, this plaque clearance is attenuated by co-incubation of U937 cells with 60 nM CLU (Fig. 6c, Supplementary Fig. 6C, *p* < 0.04), while desCLU has minimal effect (*p* < 0.02). These results, which are consistent with our prior in vivo PET studies^13^, validate a role for CLU ± Aβ oligomers in modulating CD33 ITIM signaling and in regulating phagocytic clearance of amyloid plaques.

### CLU and CD33 also interact genetically to modulate AD risk in humans

Our biophysical, molecular, and cellular data show robust functional interactions in human cells between CLU and CD33 proteins that modulate risk for AD. To see if these interactions are further reflected in genetic interactions between CLU and CD33, we analyzed the longitudinal clinical, genetic, transcriptional, and neuropathological phenotype data from 1092 participants enrolled in the ROSMAP study^85,86^. This strategy of using whole human data also provides a ground truth validation for our reductionist experiments because it takes advantage of the fact that humans are the only entity that naturally develops AD.

Genomic analyses reveal significant interactions between CD33 and CLU gene expression and risk for AD pathology (*p* = 0.026), cognitive decline (*p* = 0.041), dementia with Lewy bodies (*p* = 0.007), and neocortical Lewy bodies (*p* = 0.018; Supplementary Fig. 6D). Particularly, in brains with low CLU expression, CD33 expression is associated with global AD pathology burden (*p* = 0.002), pathological AD (*p* = 0.0008), clinical AD dementia (*p* = 0.003), and β-amyloid levels (*p* = 0.005; *n* = 546 participants) (Table 2). CD33 is also nominally associated with tangles, cognitive decline, and cerebral amyloid in the low CLU expression group but not in brains with high levels of CLU expression. Causal mediation analyses show that CD33 mediates the association between CLU expression and both clinical AD (*p* = 0.044) and amyloid burden (*p* = 0.006; Supplementary Fig. 6E). Expression Quantitative Trait Locus (eQTL) analyses modestly link CD33 expression to the CLU SNP rs9331947_G (*p* = 0.005), but not vice versa (Supplementary Fig. 6F, G). These genetic interactions are consistent with the biophysical, biochemical and cell biological studies reported above.

**Table 2:** Association between CD33 gene expression and clinico-pathological traits of AD, stratified by CLU gene expression level

	High CLU expressors				Low CLU expressors			
Trait	b	se	t	*p*	b	se	t	*p*
Amyloid	−0.0028	0.0605	−0.0455	0.9637	0.1818	0.0647	2.8101	0.0051
Tangles	−0.0485	0.0703	−0.6896	0.4908	0.1939	0.0805	2.4097	0.0163
Cognitive decline	−0.0003	0.0053	−0.0499	0.9602	−0.0144	0.0056	−2.5675	0.0105
Residual cognition	−0.0325	0.0539	−0.6036	0.5464	−0.0796	0.0549	−1.4489	0.1480
Global AD pathology burden	−0.0243	0.0320	−0.7610	0.4470	0.1111	0.0360	3.0914	0.0021
Pathological AD	−0.1106	0.1118	−0.9892	0.3226	0.3992	0.1193	3.3460	0.0008
AD dementia	0.0225	0.1346	0.1670	0.8674	0.4283	0.1453	2.9468	0.0032
TDP-43 pathology	0.1133	0.1190	0.9524	0.3409	0.1687	0.1325	1.2726	0.2032
Cerebral atherosclerosis	0.0914	0.1161	0.7874	0.4311	−0.1416	0.1183	−1.1977	0.2310
Cerebral amyloid angiopathy	0.0871	0.1143	0.7616	0.4463	0.2521	0.1232	2.0455	0.0408
Arteriolosclerosis	0.1333	0.1151	1.1578	0.2470	−0.2032	0.1174	−1.7301	0.0836
Hippocampal sclerosis	0.0216	0.1942	0.1113	0.9114	0.3751	0.2044	1.8353	0.0665
Dementia with Lewy bodies	−0.0803	0.1373	−0.5849	0.5586	0.2233	0.1389	1.6069	0.1081
Lewy bodies in neocortex	−0.0442	0.1685	−0.2621	0.7933	0.3259	0.1702	1.9146	0.0555

ROSMAP participants were stratified into two groups based on CLU gene RNA expression levels, using the median value (0.0083) as the cutoff: high CLU expressors (*n* = 546, CLU expression ≥0.0083) and low CLU expressors (*n* = 546, CLU expression <0.0083). Within each CLU-defined subgroup, we tested whether CD33 gene RNA expression was associated with clinico-pathological traits of AD. Analyses were conducted in 1092 participants with bulk RNA-sequencing data from the dorsolateral prefrontal cortex in the ROSMAP cohort, b: estimated effect size for CD33 gene expression on the trait; se: standard error of the estimate; t: t-statistic; *p*: *p*-value.

## Discussion

**Fig. 7:**
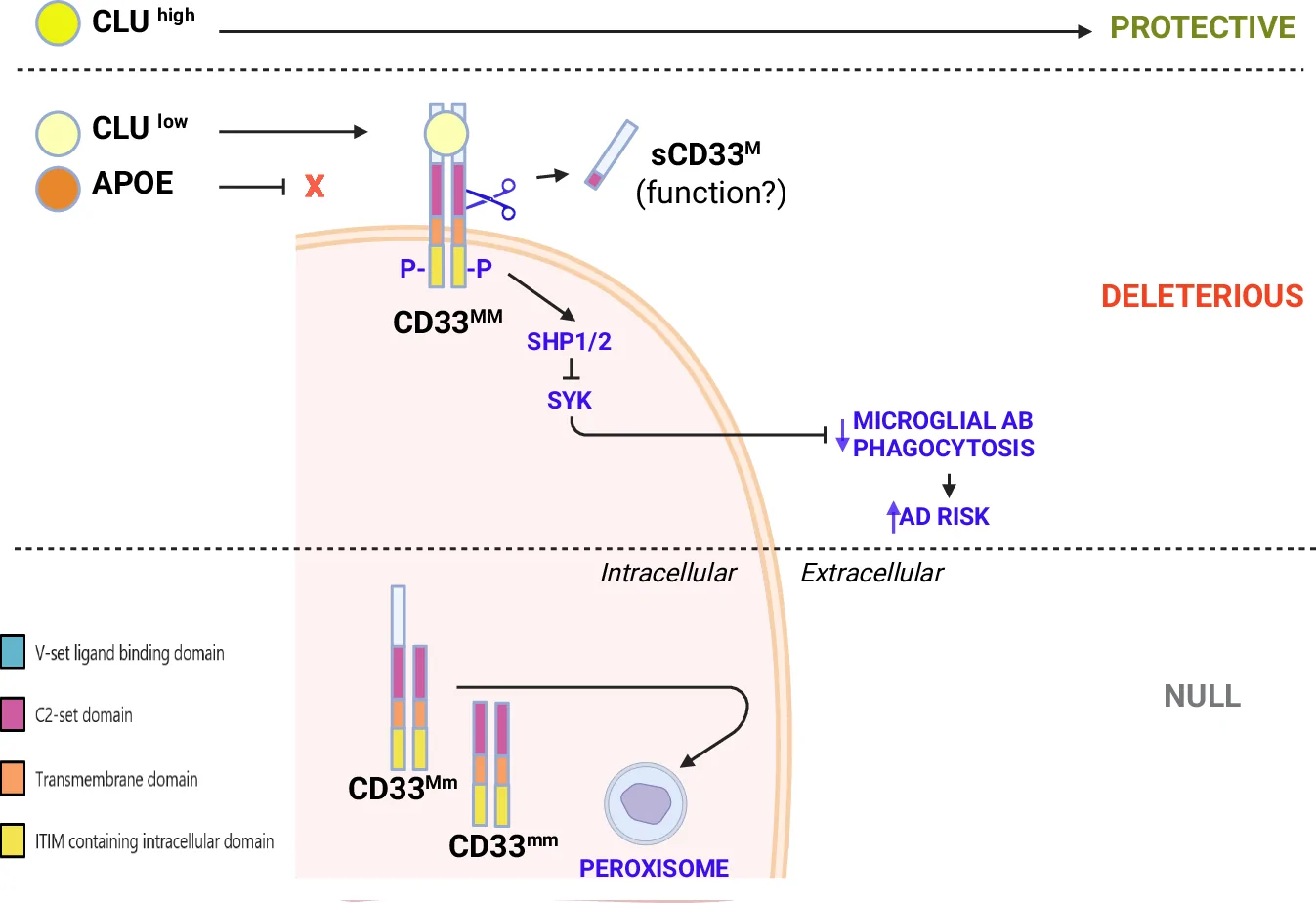
Schematic diagram illustrating the interactions of CLU and CD33 isoforms. High expression of CLU is protective by itself, likely by its chaperone effect limiting Aβ oligomer assembly. At lower levels of CLU expression, the interaction of CLU with dimeric CD33^M/M^ at the cell surface predominates, driving inhibitory CD33M ITIM signaling via recruitment of SHP1/2 in the cytoplasm, downregulating SYK-mediated activation of protective microglial responses including phagocytosis of neurotoxic Aβ species, thereby increasing risk for AD. CD33^M^ undergoes proteolytic cleavage and shedding of the sCD33 ectodomain, in a manner similar to sTREM2. Whether sCD33^M^ has biological activity remains an open question. CD33^m^ is not efficiently trafficked to the cell surface, is not cleaved to produce a long extracellular shed fragment, and as previously reported, is vectored to the peroxisome. Created in BioRender. St George-Hyslop, P. (2026) https://BioRender.com/z48u7y0.

The experiments described here deliver several unanticipated insights into CD33 receptor biology and its role in AD pathogenesis. Our key findings include the structure of the ligand-binding site and the observations that CD33^M^ dimerizes to facilitate selective binding of large sialylated molecules such as CLU and CLU + Aβ oligomers (but not ApoE) (Fig. 7). We show that CD33:CLU binding activates CD33 immunoinhibitory signaling and downregulates microglial clearance of AD-relevant molecules (e.g., Aβ, neurotoxic membrane debris), thereby increasing AD risk (Fig. 7). We also uncover a previously unrecognized shed fragment of the CD33^M^ ECD (sCD33^M^) (Fig. 7), reminiscent of the biologically active sTREM2 fragment^47,48^. Because sCD33^M^ is not produced by CD33^m^-expressing cells, if sCD33^M^ does have biological activity, it may contribute to why expression of CD33^M^ increases risk for AD while CD33^m^ does not.

Our 2.24 Å ectodomain structure provides a view of the CD33^M^ sialic acid binding pocket and the relative orientation of the V-set and C2-set domains. The topology of CD33^M^ broadly conforms to canonical Siglec topology. However, discrete differences exist from other Siglecs in the BC and CC′ loops, in the positioning of the cognate Arg119, and most notably in the formation of dimers via the C2-set domain, a feature not previously highlighted for other Siglecs. Given conservation of the dimer interface residues across human Siglecs, it will be interesting to seek evidence for similar dimerization in other Siglecs^87^.

Dimerization of CD33^M^ has profound effects on CD33 biology, likely contributing to both its subcellular localization and ligand binding specificity. The twin binding sites positioned in divergent orientations appear exquisitely optimized for simultaneous engagement of large, heavily sialylated ligands through interaction of the twin CD33^M^ binding sites with two glycan chains on the same ligand. This configuration mechanistically explains the high avidity (Kd = 28.9 nM) for heavily sialylated CLU compared to mono-sialylated sialyllactose (Kd = 6.1 mM). It also provides a theoretical framework for CD33 receptor clustering in lipid-rich rafts following binding of multiple CD33^M^ receptors to the same large, highly sialylated ligand^3,35^.

The smFRET studies reveal that CD33^M^ dimers are robustly localized to the cell-surface, whereas CD33^m^ is largely sequestered intracellularly. The molecular basis is unclear. Prior work indicates that a peroxisome-targeting sequence, which is present in all CD33 splice forms, encourages partitioning of CD33^m^ into peroxisomes^88^. Our work now identifies ectodomain dimerization as an important feature for partitioning of CD33^M^ to the cell surface. However, this dimerization motif is also present in CD33^m^, which only poorly reaches the cell surface. Our findings suggest that while dimerization is essential for cell surface presentation, additional sequence features, likely within the V-set domain, also contribute to the divergent localization of CD33^M^ to the cell surface and CD33^m^ to peroxisomes. However, regardless of the underlying mechanism, the surface enrichment of CD33^M^ in cells expressing either CD33^M/M^ or CD33^M/m^ allows comparable levels of ITIM tyrosine phosphorylation and SHP1 recruitment upon stimulation with CLU ± Aβ oligomers (Fig. 5c, d, Supplementary Fig. 5C, D, F, G). This provides a molecular explanation for the previous report of similar in vivo brain Aβ loads in heterozygous and homozygous rs3865444 C-risk carriers^13^.

We also identify soluble N-terminal CD33^M^ proteins in the media of cells expressing wild-type CD33^M^ (sCD33^M^) or the CD33^MΔ4bp^ mutant (sCD33^MΔ4bp^) (Fig. 7). These proteins may be analogous to the shed N-terminal fragment of TREM2 (sTREM2), which exerts protective effects against Aβ toxicity and AD progression^47,48^. Both sCD33^M^ and sCD33^MΔ4bp^ coimmunoprecipitate with full-length CD33^M^ (Fig. 2j), suggesting that they might influence signaling either by CD33^M^ itself or by other microglial receptors such as CD45. Indeed, if sCD33^M^ does have deleterious biological activities, its absence from CD33^m^ expressing cells raises the possibility that these putative sCD33^M^-related biological activities could contribute to the higher AD risk associated with expression of CD33^M^.

Our discovery that physiological concentrations of sialylated CLU ± Aβ oligomers are high-avidity ligands for CD33^M^, whereas ApoE is not, is of acute interest to the field. This differential binding underscores fundamental mechanistic distinctions between the way CLU and ApoE lipoproteins modify AD risk. ApoE alters AD risk in part by impairing endolysosomal transport in an isoform-specific (not sialylation-specific) manner^89^. In contrast, CLU modulates AD risk in part by altering CD33^M^ ITIM signaling in microglia (as shown here). CLU also has CD33-independent mechanisms for modulating AD risk including blocking Aβ oligomerization, protection of synapses and reduction of astrocyte reactivity^64,73,83,90,91^).

The mechanistic reason for CD33^M^’s exquisite selectivity for CLU over ApoE is particularly intriguing. One potential explanation is that although both are heavily sialylated AD‑linked proteins^62,92^, the absence of ApoE binding may reflect differences in the orientation and/or type of glycans on ApoE versus CLU. The recently published crystal structure for CLU^93^ may allow interrogation of CD33^M^:CLU ± Aβ complexes to elucidate the structural basis of CD33^M^:CLU selectivity and guide small‑molecule inhibitor design.

Our genetic and eQTL analyses in deeply phenotyped human cohorts provides a compelling and independent line of evidence connecting the CD33:CLU axis to AD risk and AD-relevant clinical traits (Table 2, Supplementary Fig. 6D, E). These data reveal that the pathogenic CD33^M^:CLU interaction is most pronounced at low CLU expression levels, whereas at high CLU expression levels CLU becomes protective. We propose a saturable activation model in which low CLU levels preferentially drive CD33^M^-mediated signaling which inhibits protective microglial responses (CLU^low^ Fig. 7). At elevated CLU expression levels generated by protective CLU alleles^91^, the inhibitory CLU-CD33^M^ effect becomes saturated and overridden by the protective effects of higher CLU expression (CLU^high^ – Fig. 7), which mediate well-documented chaperoning effects of CLU on Aβ, astrocytes and synapses^64,73,83,90,91^. Future dynamic systems biology experiments will be needed to characterize this complex quantitative relationship. However, accounting for this quantitative relationship will likely be essential for the design of future clinical trials and in personalized medicine approaches for therapeutics directed at CD33. Our data suggests that anti-CD33 therapies might be most effective when targeted to CD33-risk allele carriers with low CLU expression.

Finally, our results strengthen the existing concept that microglial function may be achieved by therapeutic inhibition of CD33^M^‑mediated ITIM signaling. Function‑blocking anti‑CD33^M^ antibodies have been proposed^94–97^, but challenges in CNS delivery and amyloid‑related imaging abnormalities (ARIA)^98^ limit their immediate translational potential. The structural insights provided here now provide a blueprint for design of small molecules or aptamers capable of disrupting dimer formation and/or ligand engagement. Brain‑penetrant, cell‑permeable inhibitors of CD33^M^ dimerization or ligand engagement, and antisense oligonucleotides that downregulate exon 2-containing CD33 transcripts are promising avenues. Because CD33 expression is restricted to microglia and monocytes, off‑target effects might be limited. Personalized approaches, particularly in individuals with CD33 risk alleles and low CLU expression, may yield the greatest therapeutic benefit.

In summary, our genetic, structural, and functional data define a mechanistic axis connecting APP/Aβ, CLU, and CD33 in AD biology. CLU (±Aβ) binds CD33^M/M^ dimers (Figs. 3 and 4), activates ITIM signaling (Fig. 5), and thereby suppresses protective microglial functions (e.g., phagocytosis) and amyloid plaque clearance (Fig. 6), as summarized in Fig. 7).

## Methods

The experiments described in this manuscript were approved by and comply with all relevant ethical and safety regulations mandated by the Research Ethics Boards of the University of Cambridge, University of Toronto and Columbia University.

### Protein expression and purification

A construct encoding residues 21–232 of CD33 (encompassing the V-set and C2-set immunoglobulin-like domains) was PCR amplified from an IMAGE clone (id 5217182) and inserted into pHLSec (a kind gift from Prof Radu Aricescu)^99^ via Gibson assembly. IMAGE clone 5217182 contains a sequence for CD33 corresponding to the R69G variant, the most common sequence observed in European (25%) and American (44%) populations (transcript CD33-201, www.ensembl.org). pHLSec encodes an N-terminal signal peptide (derived from RPTPσ) and a C-terminal hexahistidine tag. Protein was expressed by transient transfection of HEK293F cells grown in suspension in Freestyle 293 medium (Life Technologies)^100^. Briefly, cells were centrifuged at 300 × *g* and resuspended in fresh medium at 2.5 × 10^6^ cells/ml immediately prior to transfection. Plasmid DNA was added to the culture at a final concentration of 3 μg/ml and the culture incubated at 37 °C for 5 min. Following incubation, polyethyleneimine at a concentration of 9 μg/ml (from a 1 mg/ml stock in 25 mM HEPES, 150 mM NaCl, pH 7.4) and the mannosidase I inhibitor kifunensine at 5 μM were added. Twenty-four hours later, the culture volume was doubled, the HDAC inhibitor valproate added at 3 mM (to boost protein expression)^101^ and the kifunensine supplemented to return the concentration to 5 μM.

On Day 6 post-transfection, the culture was harvested and the medium mixed with 5 ml pre-washed Ni-Sepharose Excel resin (GE Healthcare) for 60 min at 4 °C. The resin was poured into a column and washed with 200 ml Ni buffer A – 50 mM HEPES, 500 mM NaCl, 25 mM imidazole, pH 7.5. CD33 ECD was then eluted with Ni buffer B – 50 mM HEPES, 500 mM NaCl, 250 mM imidazole, pH 7.5 – concentrated to 10 ml in a Vivaspin 20 10 K MWCO device and loaded on a Superdex 200 26/600 pg column pre-equilibrated in 20 mM HEPES, 200 mM NaCl, pH 7.5. Eluted CD33 ECD was subsequently concentrated to 1 mg/ml, buffer exchanged into 20 mM MES, 200 mM NaCl, pH 6.0 and incubated with 8500 units Endo H_f_ per mg protein for 6 h at RT. Endo H_f_ was removed by incubation with 1 ml amylose resin for 30 min and CD33 ECD was concentrated to 9 mg/ml and snap-frozen in 100 μl aliquots in thin-walled PCR tubes in liquid nitrogen. The final yield was ~60 mg/litre of culture.

Human plasma CLU used for BLI was purified from human plasma as described by Wilson et al.^102^. The purity analyzed by Coomassie Brilliant Blue (CBB)-stained SDS PAGE is almost 100% (Supplementary Fig 3A). To remove conjugated sialic acid groups, purified human CLU (dialyzed into sodium acetate buffer, pH 5.0) was supplemented with neuraminidase (from *Clostridium perfringens*, 1U, Sigma-Aldrich) and incubated for 6 h at 37 °C with gentle mixing on an orbital shaker. Proteins were subsequently analyzed by SDS PAGE (4–12% gradient Bis-Tris Bolt Plus pre-cast gel, ThermoFisher Scientific) and stained with Imperial^TM^ protein stain (ThermoFisher Scientific). Samples were also analyzed by Western blot; 500 µg of treated and untreated hCLU were subjected to SDS PAGE and then electrophoretically transferred to a nitrocellulose membrane, which was blocked for 30 min with 2% (w/v) BSA in PBS containing 0.1% (v/v) Tween 20. Sections of the membrane were then cut and incubated with either G7 (hCLU-specific mouse monoclonal antibody^102^, as hybridoma culture supernatant), or 2.5 µg/ml recombinant human lectin Siglec-1 (6xHis-fusion protein which binds to sialic acid groups; R&D Systems, InVitro Technologies). After an overnight incubation at 4 °C, the blots were washed three times with PBS containing 0.1% (v/v) Tween 20 and then incubated with the appropriate HRP-conjugated secondary antibody (0.32 µg/ml anti-6xHis-HRP antibody, Abcam; 0.4 µg/ml goat-anti-mouse-HRP, Dako, Agilent). Siglec-1 and HRP-conjugated antibodies were diluted in 2% (w/v) BSA in PBS containing 0.1% (v/v) Tween 20. Finally, the blots were washed as before and then developed using Pierce SuperSignal West Pico Chemiluminescent Substrate (ThermoScientific).

### Crystallization and data collection

CD33 ECD was initially set up in 5 × 96 well vapor diffusion screens by an Innovadyne Screenmaker robot and crystals were identified in approximately 10% of drops. The best initial hit was in condition H9 of the MORPHEUS screen—10% PEG 20,000, 20% PEG MME 500, 100 mM Bicine/Tris base pH 8.5, Morpheus amino acids. Optimized crystals grew as clusters of rods in 800 nl + 800 nl drops in 2% PEG 20,000, 4% PEG MME 500, 100 mM Bicine/Tris base pH 8.5, Morpheus amino acids over the course of 1–3 days. Individual rods were isolated, soaked for ~20 s in mother liquor with PEG MME 500 supplemented to 15% for cryoprotection, and cryocooled by plunging into liquid nitrogen. Data were collected at 100 K on beamline I04-1 at the Diamond Light Source, a fixed energy beamline with a wavelength of 0.920 Å.

### Ligand soaking

3ʹ and 6ʹ-sialyllactose (Carbosynth Ltd) were dissolved in crystallization mother liquor (2% PEG 20,000, 4% PEG MME 500, 100 mM Bicine/Tris base pH 8.5, Morpheus amino acids) at a final concentration of 20 mM. Crystals were transferred into 4 μl drops of either oligosaccharide solution and soaked for 10 days before soaking in cryoprotectant solution and cryocooling as for apo crystals. Data for the ligand-soaked crystals were collected on beamline I04-1 at the Diamond Light Source.

### Structure solution and refinement

Data were integrated and scaled using Xia2^103^ with the 3dii protocol (XDS^104^ for integration and processing, XSCALE for scaling), exhibiting an I/σI of 1.90 in the highest resolution shell (2.32–2.24 Å, Table 1). Crystals were in the orthorhombic space group *P*2_1_2_1_2_1_ and the MATTPROB^105^ calculator suggested the presence of 4 copies of CD33 ECD in the asymmetric unit. The structure was solved using PHASER^106^ with a model derived by SCULPTOR^107^ from the structure of Siglec-5 (pdb id 2ZG2, 56% sequence identity)^8^. The initial model had a good fit to the density for the V-set domain, but a very poor fit for the C2-set domain. The model was improved by the application of autobuild^108^ from the PHENIX^109^ software suite, which reduced R_free_ to approximately 0.33. The model was then subjected to multiple iterations of refinement in phenix.refine^110^ coupled with manual rebuilding in COOT^111^, including the use of phenix feature-enhanced maps^112^. Torsion-based non-crystallographic symmetry restraints were applied in phenix.refine, restraining dihedral angles between NCS-related copies. TLS groups were identified automatically using the phenix.find_tls_groups tool and applied during refinement. Where density was supportive of inclusion in the model, *N*-linked *N*-acetylglucosamine residues were added and linked to Asn residues using phenix.carboload. Some level of positive difference density was observed for all five potential *N*-linked glycosylation sites, suggesting that all are glycosylated within cells to some degree. However, *N*-acetylglucosamine residues were only modeled at positions Asn100, 160, and 209 (and not at Asn113 and Asn230). The final model was refined to an R/R_free_ of 0.19/0.23.

Data for the ligand-bound structures were processed and scaled as for the ligand-free structure and extended to a resolution of 2.66 Å (3ʹ-sialyllactose) and 2.48 Å (6ʹ-sialyllactose). The models were initially placed by rigid body refinement of the apo structure and similarly built and refined using iteration of COOT and phenix.refine. Where electron density supported building of ligands, monosaccharide residues were manually placed in COOT and refined in PHENIX. All structural figures were generated using PyMOL^113^.

### Analytical ultra-centrifugation

Sedimentation velocity (SV) analysis was performed in a ProteomeLab XL-I (BeckmanCoulter) analytical ultracentrifuge. Protein samples were loaded in 2-channel cells and centrifuged in an An-50 Ti 8-place rotor at 40,000 rpm, 20 °C for 15 h. Absorbance at 280 nm was used for detection. The samples contained 0.4 mg/ml glycosylated or deglycosylated CD33 ECD, 20 mM HEPES, 200 mM NaCl, pH 7.5. SV data were analyzed using Sedfit software (P. Schuck, NIH/NIBIB).

Sedimentation equilibrium (SE) experiments were performed in a ProteomeLab XL-I (Beckman Coulter) analytical ultracentrifuge. Samples of glycosylated and deglycosylated CD33 ECD at concentrations 0.2, 0.1, and 0.05 mg/ml were loaded in 6-channel equilibrium cells and centrifuged in an An-50 Ti 8-place rotor at 16,000 or 20,000 rpm and 20 °C until equilibrium was reached for each speed. Sample buffer was 20 mM HEPES, 200 mM NaCl, pH 7.5. SE data were analyzed using HeteroAnalysis software (by J.L. Cole and J.W. Lary, University of Connecticut).

### Multi-angle light scattering

SEC-MALS was used to determine the mass of Endo H_f_-treated and non-deglycosylated samples of CD33 ECD. These were resolved on a Superdex 75 10/300 GL analytical SEC column (GE Healthcare) in 20 mM HEPES, 200 mM NaCl, pH 7.5 and elution monitored using absorbance at 280 nm (Agilent 1200 MWD), light scattering (Wyatt Heleos II) and refractive index (Wyatt Optilab rEX). CD33 ECD samples were loaded at two concentrations: 2.5 mg/ml and 0.5 mg/ml (equivalent to 100 µM and 20 µM, CD33 ECD, respectively). Due to the SEC step there is a significant dilution of sample concentration during the SEC-MALS experiment meaning maximal concentration achieved are >10 times less than the loaded concentrations (i.e., <10 and 2 µM CD33 ECD). In the case of the glycosylated samples, the masses of the protein and carbohydrate components were determined using the dual detection method as implemented in Wyatt’s ASTRA software as conjugate analysis. The protein refractive index increment^114^ based on the CD33 ECD sequence used was 0.1885 ml g^−1^ and the extinction coefficient for absorbance at 280 nm was 1252 ml g^−1^ cm^−1^. The *dn/dc* value used for carbohydrate^115^ was 0.145 ml g^−1^ and its UV absorbance at 280 nm zero. The inter-detector delay volumes and associated band broadening constants, as well as the detector intensity normalization constants for the Heleos and the UV intensity calibration, were determined as part of each set of measurements using a known protein standard (BSA).

QELS measurements (DLS) were obtained during SEC MALS runs using a fast response detector in the Helios instrument that replaces one of the static light scattering detector angles. Autocorrelation functions were fit to a translational diffusion of a single species (appropriate because of the SEC step) and converted to hydrodynamic radius (Rh) using a buffer viscosity of 0.93 cP using Wyatt’s ASTRA software.

### Blue-Native PAGE in cells expressing endogenous CD33

1 × 10^6^ human THP-1 cells (ATCC, TIB-202) or peripheral blood monocyte cells from human subjects (obtained with institutionally approved informed consent protocols) were lysed in 100 µl PBS, 1% digitonin (plus protease inhibitors) on ice for 30 min. Insoluble material was pelleted at 20,000 × *g*, 5 min, 4 °C. 10 µl of the lysate was run on a BN-PAGE gel in 30 µl total volume, including G-250 additive at 0.08%. Protein was denatured in gel post-run by soaking in 1% SDS, 15 min and then blotted to PVDF. The membrane was blocked in 5% non-fat milk/PBS/0.1% Tween-20 and probed with rabbit anti-CD33 mAb EPR4423 at 1:1000. Following washing, the blot was probed with goat anti-rabbit-HRP secondary at 1:20,000 and visualized with SuperSignal West Dura substrate on film.

### CD33 dimer formation

The Myc-tagged CD33^M^ containing vector was obtained from OriGene (RC207023L1). The vector for Myc-CD33^m^ was made by removing exon 2 using the following primers and the Q5 Site Directed Mutagenesis Kit (NEB). Fwd: ACTTGACCCACAGGCCCAAAATC; Rev: CTGCCCACAGCAGGGGCA. The Myc tag was then replaced with a 3X HA tag in both vectors, resulting in vectors for Myc-CD33^M^, Myc-CD33^m^, HA-CD33^M^, and HA-CD33^m^. 10 µg of paired vectors were transfected into HEK 293T cells using Lipofectamine 3000 in the following combinations: Myc-CD33^M^/HA-CD33^M^, Myc-CD33^M^/HA-CD33^m^, Myc-CD33^m^/HA-CD33^m^. After 48 h, the proteins were immunoprecipitated with either anti-Myc (Cell Signaling, 2276S) or anti-HA (Abcam, 9110) antibodies and probed with the same antibodies or anti-CD33 (Santa Cruz, 28811) antibody.

### CD33:CLU Co-IP

10 cm dishes containing HEK293T cells (ATCC, CRL-3216) were transfected with Lipofectamine 2000 according to manufacturer’s protocol with pCMV6 plasmids containing either HA-CD33^M^ or HA-CD33^m^-HA alone, or with a 50:50 mixture of pCMV6 plasmids containing either HA-CD33^M^ or myc-CD33^m^. After three days, cells were harvested and incubated with or without 30 nM CLU purified from human plasma as previously described for 1 h on a rotating wheel at 4 °C^90^. Cells were pelleted, extensively washed with PBS to eliminate excess CLU and lysed in 1%CHAPSO/TBS (100 mM NaCl, 50 mM TRIS, 1 mM EDTA, pH 7.4). Lysates were subjected to immunoprecipitation with rabbit anti-HA antibody and protein complexes were precipitated on a protein-G-sepharose matrix in 0.5% CHAPSO/TBS buffer. After 2 washes with IP buffer and one wash with PBS, proteins were eluted with SDS-PAGE loading buffer at 65 °C for 10 min. Proteins were separated on a Novex NuPage Bis-Tris 4–12% gel and blotted onto a PVDF membrane. Membranes were probed for CLU with G7 and 41D mouse anti-CLU antibodies^90^, or mouse anti-HA antibody for CD33 tagged with HA.

### CD33/ApoE Co-IP

20 µg wild-type CD33^M^ or R119A ligand incompetent CD33^M^ proteins or ApoER2 ectodomain fused to His-tag^65^ were incubated with 50 µl ‘Dynabeads His-Tag Isolation and Pulldown’ (Thermo Fisher Scientific, Catalog # 10103D) at 4 °C overnight. Beads were briefly spun down, and washed three times with IP buffer, followed by incubation with 500 µl culture supernatant containing naturally secreted and receptor binding-competent ApoE3 or ApoE4 (3 µg/ml) prepared as previously described^92,116^, for an additional 4 h at 4 °C. Beads were washed three times using wash buffer [500 mM NaCl, 15 mM TrisHCl, 0.5% Triton X-100 (pH 8.0)]. The bound ApoE was separated on 4–15% SDS-PAGE and analyzed by Western blotting using a polyclonal antibody directed against human ApoE.

### CD33 phosphorylation and SHP1 recruitment

HEK293 cells were transfected as above with pCMV6 plasmids containing HA-CD33^M^, or HA-CD33^m^ alone, or HA-CD33^M-5X^ alone or with 50:50 mixtures of pCMV6 plasmids containing either HA-CD33^M^ or HA-CD33^m^ for two days. Cell media were removed and washed with PBS four times, then incubated with CLU 60 nM, Aβ 2 µM, or CLU 60 nM plus Aβ 2 µM for 0, 15 min, cells were lysed in 1X RIPA buffer with PhosSTOP cocktail tablet (Roche) and proteinase inhibitor (Sigma-Aldrich), 1 mg total protein lysis buffer for each sample was performed for immunoprecipitation (protein concentration measured by BCA, Pierce), Rabbit HA antibody (ab9110, Abcam) was added and incubated for 1 h at 4 °C, then Protein G beads were added and rotated for overnight at 4 °C. Beads were washed three times with 1XRIPA washing buffer, eluted in 1 X Nupage LDS buffer at 65 °C for 10 min, supernatant was subjected for western blot with mouse anti-phosphotyrosine antibody (PY20, ab10321, Abcam), or mouse anti-SHP1 antibody (14D5, Invitrogen), or mouse anti-HA antibody (H3663, Sigma-Aldrich).

### Bio-layer interferometry

Binding avidity of CLU to CD33^M^ ECD was analyzed by bio-layer interferometry (BLI) using the Octet RED384 system (ForteBio). Assays were performed at 30 °C in the assay buffer (20 mM HEPES, 200 mM NaCl, 1 mg/mL casein, pH 7.5) with vibration at 1000 rpm. Ni-NTA biosensors loaded with hexahistidine tagged deglycosylated CD33^M^ ECD wild-type (WT) or R119A were incubated with CLU in a range of 0.0025–5 µM to obtain the association curves. Equilibrium dissociation constant (Kd) was analyzed using the averaged binding responses at 895–900 s.

Binding affinity of 3’-sialyllactose to CD33^M^ ECD was analyzed by BLI at 30 °C with vibration at 1000 rpm in the same assay buffer with CD33^M^:CLU ECD interaction. Super Streptavidin biosensors loaded with 3’-sialyllactose-sp-biotin (Sigma-Aldrich) were incubated with CD33^M^ ECD WT or R119A in a range of 15.6–250 µM to obtain the association curves, and subsequently in the assay buffer to obtain the dissociation curves. Kinetics data were analyzed using a 1:2 bivalent analyte model.

### Microscale thermophoresis

Microscale thermophoresis analysis was performed with the Monolith NT.115 instrument (NanoTemper) using excitation power of 20% at 23 °C. Deglycosylated CD33 ECD was labeled with Alexa Fluor 647 via primary amine coupling. The proteins were diluted in 20 mM HEPES (pH 7.5), 150 mM NaCl, 0.05% Tween-20. Kd values were calculated using the MO Affinity Analysis software.

### Far-UV circular dichroism (CD)

Far-UV CD spectra were recorded on a Jasco J-720 spectrometer using a 1 mm path length cuvette at 4 °C. The wild-type (WT) CD33^M^ ECD and R119A (0.2 mg/mL) were prepared in a buffer (20 mM HEPES, 200 mM NaCl, pH 7.5). CD spectra were obtained from 205 to 260 nm, with a 1-nm bandwidth, and 8-s response time and a scan speed of 20 nm/min. Data are averaged from five consecutive scans.

### Flow cytometry

HEK293T cells were plated into 6-well plates one day before transfection. Cells were transfected with GFP (1 µg) plus CD33^M^ (2 µg), CD33^m^ (2 µg) or CD33^M^ + CD33^m^ (1 µg+1 µg) plasmids using Lipofectamine3000. Twenty-four hours after transfection, cells were collected by trypsinization and then washed twice with 1x PBS. Cells then were stained by Viability Dye eFluor450 (65-0863, eBioscience) at 4 °C for 30 min. After washing, cells were resuspended with 1x PBS and 1 million cells per test were then stained with primary antibodies (WM53-PE and A16121H for the double staining; HIM3-4-PE for the single staining) for 30 min at 4 °C. After washed with 1x PBS 3 times, double stained cells were stained with secondary antibody (anti-ratIgG2a-AF647) for 30 min at 4 °C. After being washed 3 times, cells were transferred to flow cytometry test tubes and data were acquired by BD FACS LSRII with corresponding compensation controls. Data were analyzed by FlowJo v10. GFP positive cells were analyzed after sequential gating to exclude debris, doublets, dead cells, GFP negative cells and cells with very high GFP expression that could not be compensated properly.

CD33^M-5X^ cells were not included in the flow cytometry experiments because the 5X dimer site mutant disrupts the recognition motif for the WM53 anti-CD33 antibody used to study CD33^M^ in fluorescence flow cytometry. These cells were studied by surface labeling.

### SNAP-tagged plasmid construction for smFRET studies

Plasmids coding for various CD33 receptors tagged at their amino-terminus with the catalytically enhanced version of the SNAP tag - SNAP_fast_ (SNAP_f_) were constructed using standard subcloning methods by replacing the dopamine D2 receptor coding region of the previously described pcDNA5/FRT/TO-IRES SNAP_f_-D2 vector^117^ with the following coding regions: human CD33^M^ (positions 17–364); human CD33^M-5X^; and human CD33^m^ (positions 13–237). These vectors contain a weak Kozak sequence with thymine at the −3 and +4 positions and a crippled CMV promoter in which the enhancer sequence was partly deleted. All constructed and commercial plasmids were confirmed by whole-plasmid DNA sequencing (Plasmidsaurus).

### Cell culture and generation of stable CHO cell lines for smFRET studies

All Chinese Hamster Ovary (CHO) cell lines (ATCC, CCL-61) were maintained in Ham’s F-12 medium (Corning), 10% Fetal Bovine Serum (Corning), 2 mM L-glutamine (Corning) at 37 °C in 5% CO_2_. For parental LEx-Flp-In T-REx (FITR) CHO cells, a previously described FITR cell line containing an FRT recombination target site^118^ in the CHO genome that confers low expression levels and that also allows for tetracycline-inducible expression^41,119^, the medium contained 15 μg ml^−1^ blasticidin (InvivoGen) and 50 μg ml^−1^ zeocin (Invitrogen). For stable LEx-FITR CHO lines expressing CD33 receptors, the medium contained 500 μg ml^−1^ hygromycin (Thermo Fisher Scientific) and 15 μg ml^−1^ blasticidin. All CHO lines were grown to ~70–80% confluency in six-well tissue culture dishes before transfection for generating stable lines or tetracycline (MilliporeSigma) induction and/or labeling for microscopy.

To generate stable CHO cell lines expressing SNAP_f_-CD33^M^, -CD33^M-5X^, and -CD33^m^, 200 ng of the pcDNA5/FRT/TO-IRES vectors encoding these receptors was co-transfected with 1800 ng of the pOG44 Flp-In Recombinase vector (Invitrogen) into the LEx-FITR CHO lines using Lipofectamine LTX according to the manufacturer’s protocol. Stable cells were selected with 500 μg ml^−1^ hygromycin (Thermo Fisher Scientific) in the presence of 15 μg ml^−1^ blasticidin.

### Sample preparation for TIRF microscopy

The LEx-FITR CHO stables expressing SNAP_f_-CD33^M^, -CD33^M-5X^, or -CD33^m^ were induced with tetracycline (0, 200, and 1000 ng.ml^−1^ for SNAP_f-_CD33^M^, -CD33^m^, and -CD33^M-5X^ expressing stable lines, respectively). 18–24 h before labeling and imaging to yield comparable cell surface expression levels suitable for resolving single molecules. The CHO cells were then prepared for single-molecule imaging by first stochastically labeling with the membrane-impermeant, self-healing SNAP_f_-reactive Lumidyne dyes LD555p-benzylguanine (BG) (Acceptor, 666 nM) and LD655-BG (Donor, 333 nM)^119,120^ in conditioned culture medium for 20 min at 37 °C in 5% CO_2_ under dark conditions. The donor-only control was labeled with 333 nM LD555p-BG in the absence of the acceptor dye following the same procedures above. After labeling, cells were extensively washed with Dulbecco’s Phosphate-Buffered Saline (DPBS) supplemented with 5 mM Glucose (Sigma-Aldrich) and 1 mg.ml^−1^ Bovine Serum Albumin (BSA; Sigma-Aldrich) and then dissociated with PBS-based enzyme-free cell dissociation buffer (Sigma-Aldrich). Cells were then centrifuged at 500 *RCF* for 5 min, resuspended in FluoroBrite DMEM (ThermoFisher) and seeded onto fibronectin-coated (10 ng µl^−1^, Sigma-Aldrich) high-index glass coverslips (1.7 numerical aperture (NA) HIGHINDEX-CG 20 mm diameter, Olympus) and allowed to adhere for 60 min at 37 °C in 5% CO_2_. Prior to use, the coverslips were chemically cleaned in an ultrasonic bath sonicator (CPX3800, Branson Ultrasonics) using the following sequence of steps: 20 min in 10% Alconox (Sigma-Aldrich), 10 min in ultrapure water, twice for 40 min in potassium hydroxide (1 M, Sigma-Aldrich), followed by 20 min in ultrapure water. Once chemically cleaned, the coverslips were further processed under argon plasma (Zepto RIE, Diener Electronic) for 5 min and then coated with 10 ng.μl^−1^ fibronectin (Sigma-Aldrich) for 60 min before seeding with labeled cells.

After the cells adhered onto the coverslips, and immediately before imaging, they were washed with excess DPBS and then assembled onto a microscopy chamber^117^, where they were incubated at room temperature for 30 min in dark conditions in imaging buffer containing 1 mM 3,4-dihydroxybenzoic acid (PCA) and 50 nM protocatechuate 3,4-deoxygenase (PCD) (Sigma-Aldrich) to deplete dissolved ambient oxygen by 50% as previously described^119^.

### TIRF microscopy and smFRET imaging

Image sequences were collected at a time resolution of 15 ms using a customized, objective-based IX81 TIRF microscope equipped with a cellTIRF-4Line illuminator system (Olympus Life Sciences/Evident) containing a custom microscope enclosure to create a controlled, lightproof environment (Precision Plastics Inc.). A 100× oil-immersion 1.70-NA (APON100×HOTIRF, NA 1.70, Olympus) objective was used with a 1.705 NA immersion oil (Cargille Labs) for imaging the samples. An evanescent TIRF field with an approximate penetration depth of 80 nm was used to excite labeled receptors at the proximal plasma membrane using a 532-nm (Opus 3600, Laser Quantum, 3W)^41^ and/or 640-nm (LAS-640-100D, Cell^tirf^, Olympus, 100 mW) lasers. Fluorescence emission was separated from the excitation light using a dual-band laser filter set (ZT523/640rpc, ZET532/640 m, ZET532/640x, Chroma) in combination with an emission side image splitter (OptoSplit 2, Cairn) equipped with a FRET filter set (ZT640rdc, ET585/65 m, ET655lp, Chroma) to spatially separate and project donor and acceptor emission signals side-by-side onto a single quantitative complementary metal oxide semiconductor (qCMOS, ORCA Quest C15550-20UP, Hamamatsu Photonics) camera for detection. The dual-band TIRF–FRET filter configuration allows for the excitation of donor and acceptor fluorophores separately or simultaneously. The microscope is also equipped with nano- and micro-drive stage positioning systems (Mad City Labs Inc.) for locating single living cells.

Before smFRET imaging, cells were briefly excited with both 532-nm and 640-nm laser lines at 10 mW measured at the back focal plane to generate an initial image for quantifying the density of donor- and acceptor-labeled CD33 receptors. SmFRET imaging was then performed by exciting the same cells with only the 532-nm laser (10 mW at back focal plane) and acquiring 5000 frames per image sequence in both donor and acceptor channels.

### Determination of surface density by single-particle detection

The surface density of labeled molecules was determined from an initial image of cells taken from direct and simultaneous excitation before smFRET imaging. The number of donors and acceptors within a region of interest for each cell was determined by the DoG particle-detection function of TrackMate in ImageJ^121,122^. The cell surface area and region of interest was determined by boundary tracing on the projected image, generated from the donor image stack using the ImageJ plugin ZProject with projection type ‘Standard Deviation’^122^.

### Tracking smFRET in living cells

The smFRET image sequences were processed using the previously described and publicly available smCellFRET platform^3^ for tracking and subsequent analysis of smFRET in living cells to detect and quantify the number of sensitized acceptor events and to generate their corresponding smFRET trajectories, fluorescence, and smFRET time traces. The tracking constants for donor and acceptor are shown in Supplementary Table 1; all other calculations were performed as previously reported^3^. For freely diffusing-trajectory (FDT) analysis, freely diffusing smFRET trajectories, as well as freely diffusing segments from smFRET trajectories with more than one diffusion state, were determined by divide-and-conquer, moment-scaling spectrum (DC-MSS) analysis^41,123,124^.

### Plotting and statistics for smFRET data

Plotting, distribution fitting, and statistics for all single-molecule data were carried out using GraphPad Prism (Version 10.3.1 (509) GraphPad Software LLC). To determine *P* values, ordinary one-way ANOVA with Šídák’s multiple comparisons test was performed for multisample comparisons.

### CD33^MΔ4bp^ co-IP

HEK293 cells were transfected as above with single pCMV6 plasmids containing: (i) empty vector (control) alone; or (ii) HA-CD33^M^ alone; or (iii) HA-CD33^m^ alone; or (iii) CD33^ΔM4bp^ alone; or (iv) with equal amounts of two pCMV6 plasmids, one of which contained HA-CD33^MΔ4bp^ and the other contained either HA-CD33^M^ or HA-CD33^m^. Cells were grown for 2 days. Supernatant media were removed. Cells were washed with cold PBS and lysed in 1X IP lysis buffer with PhosSTOP cocktail tablet (Roche) and proteinase inhibitor (Sigma-Aldrich). Equal amounts of cell lysates from each sample were used for immunoprecipitation. Anti-HA magnetic beads (SAE0197, Sigma-Aldrich) were added and incubated for overnight at 4 °C. Beads were washed three times with 1X IP lysis buffer, eluted in 1 X NuPage LDS buffer at 70 °C for 10 min. The supernatant were western blotted and probed with rabbit anti-CD33 Ig-like V type domain antibody (ab134115, Abcam).

### hiPSC maintenance

The FA0000010 (FA10) hiPSC line was generously provided by Barbara Corneo^125^. Undifferentiated hiPSCs were cultured in StemFlex medium (ThermoFisher) supplemented with penicillin/streptomycin (ThermoFisher) on reduced growth factor Cultrex BME (Bio-Techne). Cells were routinely passaged 1–2 times per week using ReLeSR (STEMCELL Technologies) without ROCK inhibitor (Y-27632, Selleck Chemicals) as previously described^126^.

### iPSC-derived microglia (iMG) differentiation

hiPSCs were differentiated into iMGs following previously established protocols with minor modifications^126–128^. Hematopoietic precursor cells (HPCs) were generated using the STEMdiff Hematopoietic Kit (STEMCELL Technologies), following the manufacturer’s instructions. Floating HPCs were gently harvested and cryopreserved at three separate time points: days 11, 13, and 15 (or alternatively, days 12, 14, and 16). Previously cryopreserved HPCs were terminally differentiated into iMGs by plating at a density of 60,000 cells/cm² on reduced growth factor Cultrex BME in microglia differentiation medium composed of DMEM/F12 supplemented with 2X insulin-transferrin-selenium, 2X B27, 0.5X N2, 1X GlutaMAX, 1X non-essential amino acids, 400 µM monothioglycerol, and 5 µg/mL human insulin (ThermoFisher). The medium was freshly supplemented every other day with 100 ng/mL IL-34, 25 ng/mL M-CSF (R&D Systems), and 50 ng/mL TGFβ1 (STEMCELL Technologies) through day 24. On day 25, 100 ng/mL CD200 (Bon Opus Biosciences) and 100 ng/mL CX3CL1 (R&D Systems) were added to the existing supplement regimen and maintained for the remainder of the culture period. For all experiments in this study, iMGs were stimulated and collected between days 35–37.

### CLU stimulation and lysate/media collection

Native human plasma-derived clusterin (BioVendor R&D) was reconstituted in sterile water according to the manufacturer’s instructions. iMGs were treated with 300 nM clusterin or sterile water in complete microglia medium for 24 h. After 24 h, media was collected, centrifuged at 14,000 rpm for 10 min at 4 °C, aliquoted, and snap-frozen on dry ice. iMGs were lysed using Pierce™ IP lysis buffer (ThermoFisher) supplemented with 1X Halt™ Protease and Phosphatase Inhibitor Cocktail and 1 mM sodium orthovanadate (New England Biolabs).

### Full length and soluble CD33 IP from human iMG

CD33 antibody (ab134115, Abcam) and protein A Sepharose beads (GE17-0780-01, Millipore Sigma) were incubated at 4 °C overnight. After washing 2X with 0.2 M Na Borax/Boric acid buffer (pH 9.0), beads were suspended in 0.025 M Borax (pH 9.45) containing 5.2 mg of DMP (dimethyl pimelimidate, D8388, Sigma-Aldrich) and incubated for 30 min at RT. Beads were washed and incubated with 0.2 M ethanolamine (pH 8.0) for 2 h at RT to stop crosslinking. Uncrosslinked antibodies were removed and beads were washed sequentially with 10 mM NH_4_HCO_3_, 100 mM glycine, 100 mM Tris/HCl (pH 8.0) and PBS. The crosslinked beads were kept at 4 °C and used for the following CD33 IP.

Cell lysates and media supernatants from cultures iMGs were incubated overnight at 4 °C with crosslinked beads. Beads were washed 3X with 0.5X IP lysis buffer and 1X with H_2_O and then eluted three times in 0.3% trifluoroacetic acid at RT for 5 min. Eluates were neutralized with 1 M Tris buffer and then concentrated by speed vacuum. The concentrated IP products were mixed with 4XNuPage LDS buffer, heated at 70 °C for 10 min and then subjected to western blotting with mouse anti-CD33 Ig-like C2 type domain antibody (PWS44, Sigma-Aldrich).

### Proximity ligation assay (PLA)

The PLA assay was performed on formalin-fixed paraffin-embedded brain slices from three individuals with Alzheimer’s disease from the Maritime Brain Tissue Bank following the kit instructions (Duolink In Situ Detection Reagents Orange, Sigma-Aldrich DUO92007). Briefly, after deparaffinization and antigen retrieval with sodium citrate buffer (pH 6.0), tissues were permeabilized and blocked non-specific binding then the primary antibodies, rabbit anti-human CD33 (Sigma-Aldrich HPA035832, 1:100) and mouse anti-human clusterin-a (Santa Cruz sc-32264, 1:50), were performed overnight at 4 °C. After several washes with PBS-T (0.1% tween), secondary antibodies conjugated with oligonucleotides (PLA probe mouse PLUS and PLA probe rabbit MINUS, Sigma-Aldrich) are added to the reaction and incubated. The samples were washed with TBS-T (10 mM Tris, pH 7.5, 150 mM NaCl, 0.05% tween, prepared freshly) two times. The Ligation solution, consisting of two oligonucleotides and Ligase, is added and the oligonucleotides hybridize with the two PLA probes and join to a closed circle if they are in proximity theoretically ranging from 0 to 40 nm. Then the samples were washed with TBS-T two times. The Amplification solution, consisting of nucleotides and fluorescently labeled oligonucleotides, was added together with Polymerase. The samples were washed with wash buffer B (0.2 M Tris, pH 7.5, 0.1 M NaCl) three times then, incubated with primary antibodies, rabbit anti-Iba1 (Wako, 01919741, 1:100) and mouse anti-Aβ (Creative Biolabs, NAB61, 1:50) for 1.5 h at room temperature or overnight at 4 °C. After several washes, samples are incubated with secondary antibodies, goat anti-rabbit IgG Alexa Fluor 488 (Jackson ImmunoResearch, 1:200) and goat anti-mouse IgG Alexa Fluor 647 (Jackson ImmunoResearch, 1:200). After several washes with PBS, samples were incubated with 1X TrueBlack for 30 s and washed three times with PBS then mounted with Duolink In Situ Mounting Medium with DAPI (Sigma-Aldrich). The PLA signal was detected with Cy3 filter by confocal fluorescence microscopy (Zeiss LSM880) and analyzed by ZEN (Zeiss).

### Antibodies used

The following antibodies were used: Myc: (Cell Signaling, 22776S); HA: (rabbit polyclonal, Abcam, ab9110; mouse monoclonal clone HA-7, Sigma-Aldrich, H9658; mouse monoclonal, Sigma-Aldrich, H3663); CD33: (Santa Cruz, 28811; rabbit monoclonal, Abcam, Ab134115; mouse monoclonal, Millipore Sigma, 133M-1 (PWS44); mouse monoclonal EMD Millipore, CBL163 (Clone WM53); eBioscience, 12-0339-41; eBioscience, 12-0338-42 (WM53-PE); Biolegend, 94835 (A16121H—not yet commercially released); eBioscience, #12-0339-41 (HIM3-4-PE); rabbit polyclonal, Sigma-Aldrich HPA035832); SHP1: (Thermo Fisher Invitrogen 14D5); Anti-phosphotyrosine (PY20, Abcam Ab10321). CLU: (The anti-clusterin monoclonal antibodies were obtained from Mark Wilson (G7 and 41D hybridoma); mouse monoclonal, Santa Cruz sc-32264, clone 78E); Iba1: (rabbit polyclonal, Wako, 01919741); Aß: (mouse monoclonal, Creative Biolabs, TAB0792CLV, clone NAB61); Isotype control: rat IgG2a (Biolegend #405416, AF647, 1:80 for flow cytometry); mouse IgG1k PE (eBioscience #12-4714-82, 1:80 for flow cytometry); mouse IgGκ (from Wilson Lab for PLA); rat IgG2ak (Biolegend #400501, for flow cytometry); mouse IgG1 (EMD Millipore MABC002, for CBL163 WM53 control). All antibodies have been used in previously published work by other groups.

### CLU and CD33-mediated modulation of degradation of amyloid plaques in ex vivo brain slices

Coronal brain sections (20 µm) were prepared from 6- to 8-month-old 5XFAD mice {Oakley, 2006 #2614} using a cryostat as previously described {Farfara, 2011 #2949}. U937 monocyte cell line (ATCC, CRL-1593.2) at 3 × 10^5^ cells per 100 µl in RPMI 1640 (Biological Industries, 01-100-1A) were plated on unfixed brain sections and incubated at 37 °C 5% CO_2_ for 48 h with or without CLU (60 nM) or 10 µg/ml WM53 anti-CD33 antibody or isotype control. Quantification of hippocampus Aβ burden was done as described {Farfara, 2011 #2949}. Sections were washed three times with PBS (Sigma D8537) and fixed with 4% PFA (paraformaldehyde) for 10 min. Sections were washed three times for five min with PBS. Immunostaining was done with mouse anti-human 6E10 antibody (1:500) (Biolegend: cat # 803001) for 1 h at room temperature. Sections were than washed three times with PBS and stained for goat anti-mouse secondary antibody FITC-conjugated (Invitrogen; A11001) for 1 h at room temperature. Sections were washed three times for 5 min with PBS and quantification was done in a blinded fashion using Imaging Research software from the National Institutes of Health in an unbiased stereological approach.

### Collection of human peripheral blood mononuclear cells

De-identified blood was collected from the New York Blood Center (NYBC) or Partners’ Crimson Biospecimen Repository Core. All blood draws and data analyses were done in compliance with protocols approved by the Institutional Review Boards of each institution. Both males and females were used. Monocytes were separated by Lymphoprep gradient centrifugation (StemCell Technologies). Monocytes were frozen at a concentration of 2 × 10^7^ cells/mL in 10% DMSO (Sigma-Aldrich)/90% fetal bovine serum (vol/vol, Corning). After thawing, monocytes were washed in 10 ml of phosphate-buffered saline. Samples were genotyped for rs8356444 using primers and the TaqMan SNP genotyping assay from ThermoFisher Scientific.

### Amyloid-β (Aβ 1-42) uptake assay

Monocytes were isolated from peripheral blood mononuclear cells (PBMC) by using anti-CD14 + microbeads (Miltenyi Biotech). 100,000 monocytes per well were plated in RPMI with 1% fungizone, 1% penstrep, and 10% FBS in a 96-well plate. The following final concentration was used for the Aβ 1-42 uptake assay: 60 nM Clusterin (BioVendor, NC), 250 nM beta-Amyloid (1-42), HiLyte™ Fluor 647-labeled (Anaspec), 60 nM desialylated CLU. Clusterin or desCLU was preincubated with Aβ 1-42 for 15 min. in the cell culture incubator before being added to the monocytes. For the uptake assay, the preincubated clusterin and Aβ 1-42 mixture was added to the cells and incubated for 1 h at 37 °C. A total of *n* = 10 individuals were used in each treatment condition. After the incubation, cells were washed using staining buffer (1% FBS in PBS) and then followed by incubation with fixable dead cell stain (ThermoFisher, L34955). Cells were fixed with 4% PFA. Aβ 1-42 uptake was measured by using NovoCyte Quanteon Flow Cytometer (Agilent, Santa Clara, CA).

### flowPLA

The flowPLA assay was performed as per the manufacturer’s instruction (Sigma-Aldrich). Briefly, primary monocytes were permeabilized for 10 min on ice, then fixed for 30 min on ice using the Invitrogen Fix & Perm Cell Permeabilization Kit. In the first antibody incubation, rabbit anti-human CD33 (Bioss, bs-2042R, 1:400) and mouse anti-human SHP-1 (Thermo Scientific, MA5-38614, 1:400), were performed overnight at 4 °C. After two washes with Duolink wash buffer (Sigma-Aldrich), secondary antibodies conjugated with oligonucleotides (PLA probe MINUS and PLA probe PLUS, Sigma-Aldrich) were added to the reaction and incubated for 1 h at 37 °C. The samples were then washed with Duolink wash buffer twice. The Ligation solution, consisting of two oligonucleotides and Ligase, was added and the oligonucleotides hybridized to the two PLA probes. Then the samples were washed with Duolink wash buffer two more times. The Amplification solution, consisting of nucleotides and fluorescently labeled oligonucleotides, were added together with the polymerase. The oligonucleotide arm of one of the PLA probes acts as a primer for a rolling-circle amplification (RCA) reaction using the ligated circle as a template, generating a concatemeric (repeated sequence) product. The fluorescently labeled oligonucleotides will hybridize to the RCA product. The samples were washed with Duolink wash buffer and then incubated with the Duolink flowPLA Violet Detection solution. The signal is visible as a distinct fluorescent spot and analyzed by flow cytometry (Novocyte Quanteon Cell Analyzer).

### Statistical analysis of CLU-CD33 interaction in the ROSMAP data

CLU-CD33 gene expression interactions were investigated using the ROSMAP RNA-sequencing and GWAS data that have been previously described to examine the interplay between CLU and CD33 using three models^129,130^. When ROSMAP participants are enrolled, they are not known to have dementia and have agreed to detailed clinical evaluation and brain donation at death^131^. ROSMAP clinical and pathologic methods of characterization have been previously described^132–137^. Linear regression models for quantitative traits and logistic regression models for binary traits were used to test the association of CLU gene expression with CD33 expression in the dorsolateral prefrontal cortex (DLPFC) with AD neuropathological measures. The gene expression levels were adjusted for age, sex, RIN score, postmortem interval, and other technical covariates for RNA-sequencing and the residual expression values were used for interaction testing. A quantitative trait loci approach was used to test whether genetic variation in CLU regulates CD33 expression levels and vice versa. Common SNPs (MAF > 0.01) were tested for association with residual gene expression levels in a linear regression model adjusting for age, sex, and population substructure variables. Lastly, the R mediation package was used to test whether the association of CLU expression with clinical AD, AD neuropathology and cognition is mediated by CD33 levels. This package was also used to test mediation in the other direction (CLU mediates CD33 association with traits) to establish causality. Additionally, we tested the association of CD33 with AD neuropathology independently in the top and bottom 50th percentiles of CLU expression levels. This was to assess the difference in CD33 association with AD neuropathology in the presence of high or low CLU expression.

### Sex and gender

Both male and female human brain samples and human peripheral blood monocytes were used in this study. Female animals were used in the plaque phagocytosis studies. U937 cells are human male cells.

### Human subjects

Monocytes were derived from the peripheral venous blood of healthy control volunteers from the BWH blood bank. Samples were genotyped for rs8356444 using primers and the TaqMan SNP genotyping assay from Applied Biosystems (now ThermoFisher Scientific). All blood draws and data analyses were done in compliance with protocols approved by the Institutional Review Boards of each institution. Anonymized data were used from individuals enrolled in the ROSMAP study.

### Statistics and reproducibility

For simplicity, *p*-values are reported in figures in the style *p* < 0.01. As reported in the figure legends, experiments are done using at least three independent biological samples. Representative images are selected randomly. The entirety of the data is assessed statistically using appropriate statistical tests including ANOVA, unpaired student’s t-test, and Welch’s t-test, and are described for each figure in relevant legends. Statistical treatments are detailed in Supplementary Table 7. Samples sizes were determined empirically, and no data were excluded. Analysts were blinded to the identity of the samples.

### Reporting summary

Further information on research design is available in the Nature Portfolio Reporting Summary linked to this article.

## Supplementary information

Supplementary Information

Reporting Summary

Transparent Peer Review file

## Source data

Source Data for Main Figures

Source Data for Supplementary Information

## Data Availability

Coordinates and structure factors have been deposited in the Protein Data Bank under accession codes 5IHB (Apo CD33), 5J06 (3-SL complex), and 5J0B (6-SL complex), available at: 10.2210/pdb5IHB/pdb; 10.2210/pdb5J06/pdb; and 10.2210/pdb5J0B/pdb, respectively. Source data are provided with this paper and have been deposited in Figshare at: 10.6084/m9.figshare.29604716. The raw single-molecule image data generated and analyzed that support the findings of this study are available from W.B.A. and J.A.J. upon request. These image data are not deposited in a public database because of their large file sizes. Other raw data is available upon request from P.-St.G.-H., E.M.B., or W.B.A. Source data are provided with this paper.

## Code availability

The smCellFRET data analysis pipeline is freely available for academic use. The software and updated versions can be downloaded at http://innovation.columbia.edu/technologies/CU15268. Other software used to collect and analyze data for this work as described in the “Methods” either was published previously or is commercially available.
