# Supplementary material for: CD33 and clusterin interact biophysically and genetically to modulate Alzheimer risk
Source: Nat Commun. 2026 Jul 17;17:8983. doi: 10.1038/s41467-026-75140-3 (PMC13507242; doi:10.1038/s41467-026-75140-3)

SUPPLEMENTARY INFORMATION FILE

- Source images for western blots are at the end of this file.
- Numerical Source Data is available in Excel spread sheets for supplementary data.
- Exact P-values are provided in Supplemental Table 7.

SUPPLEMENTARY FIGURE 1A

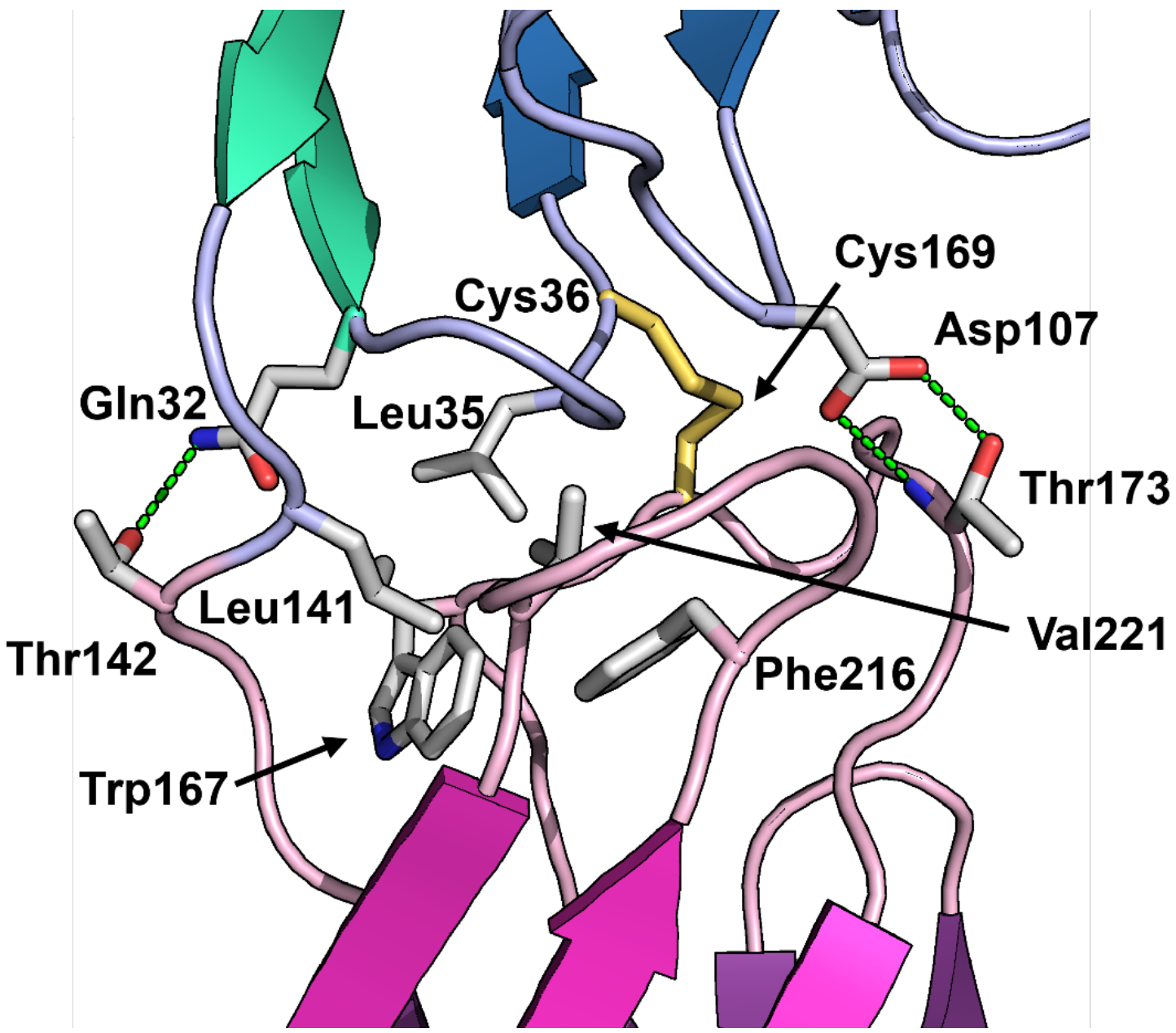

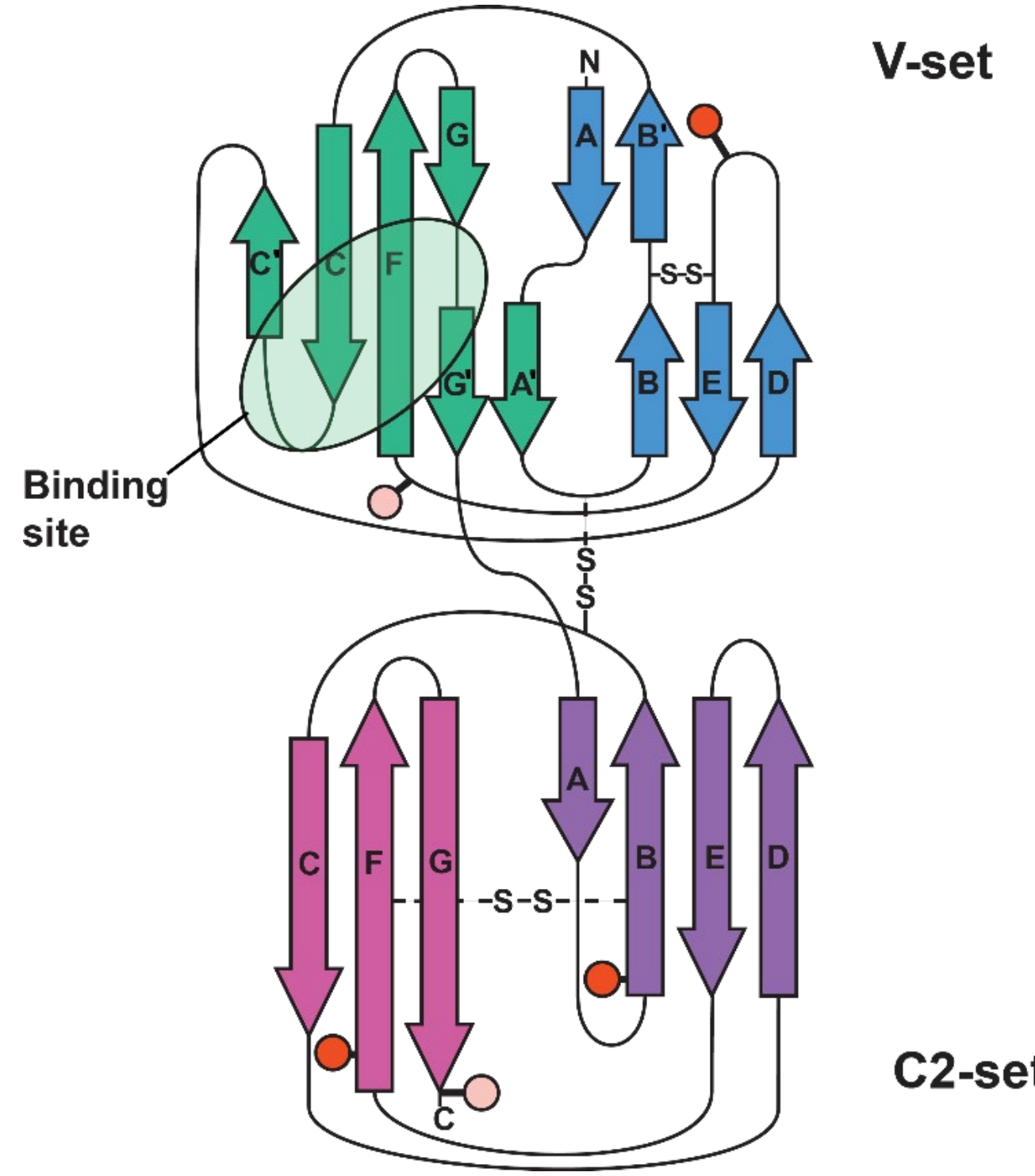

LEFT: Details of the interface between the V-set and C2-set domains. Indicated are the hydrophobic side chains in the centre of the interface and the polar side chains involved in hydrogen bonds (green dotted lines). The interdomain disulphide bond between Cys36 and Cys169 is shown in yellow.

RIGHT: The topology of the V-set and C2-set domains, coloured as in (ii). The glycosylation sites are indicated by red circles (fully occupied) and pink circles (partial occupancy).

SUPPLEMENTARY FIGURE 1B

Structural comparisons to additional CD33 structures and paralogue siglec structures

Structural alignments of i – four CD33 V-set domain structures: 5IHB (lime), 6D48 (purple), 7AW6 (blue) and AF_P20138_F1 (AlphaFold2 model, green); ii V-set domains of sialoadhesin (1OD9, red), CD33 (blue), Siglec-5 (2ZG2, green) and Siglec-7 (2HRL, purple). Significant deviations are limited to the BC loop and the CC′ loop. The CC′ loop occupies conformations varying from more open (CD33[M]) to closed (Siglec-5) over the binding site; **iii full-length ECDs of 5IHB (cyan) and 7AW6 (purple) aligned on C2-set domain only.** All structures were aligned using the align algorithm in PyMOL.

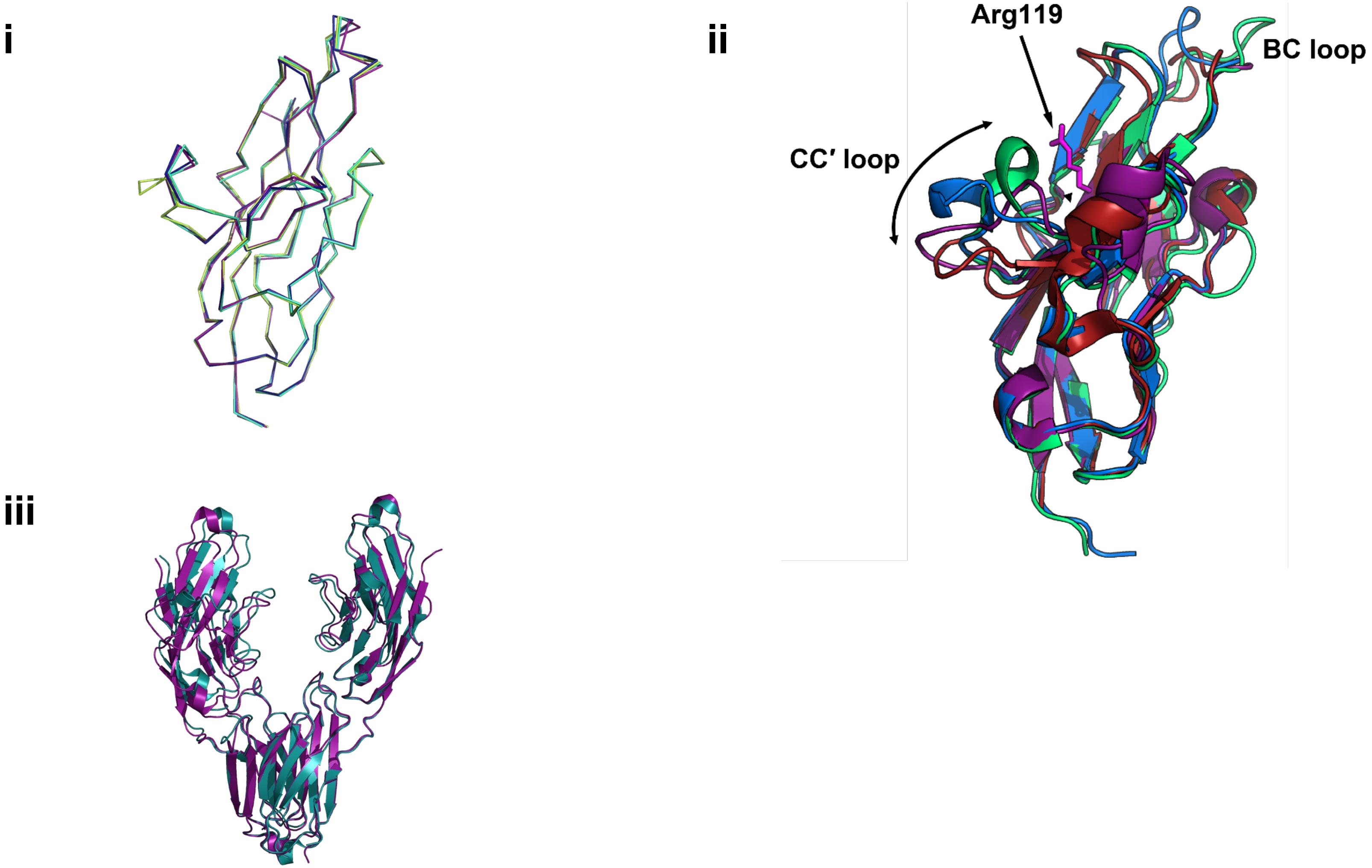

SUPPLEMENTARY FIGURE 1C

Structural alignments of CD33 C2-set domain

Left panel - Cartoon showing a structural alignment between the C2-set domains of CD33 (green) and Siglec-5 (blue), with an RMSD = 0.93 Å. Whereas the CD33 domain possesses a D strand, the region between the C and E strands in Siglec-5 occupies a non-regular loop conformation

Right panel: Structural alignment of the C2-set domains of CD33 (green) and ICAM-3 (purple). Both domains possess a D strand. RMSD = 1.9 Å.

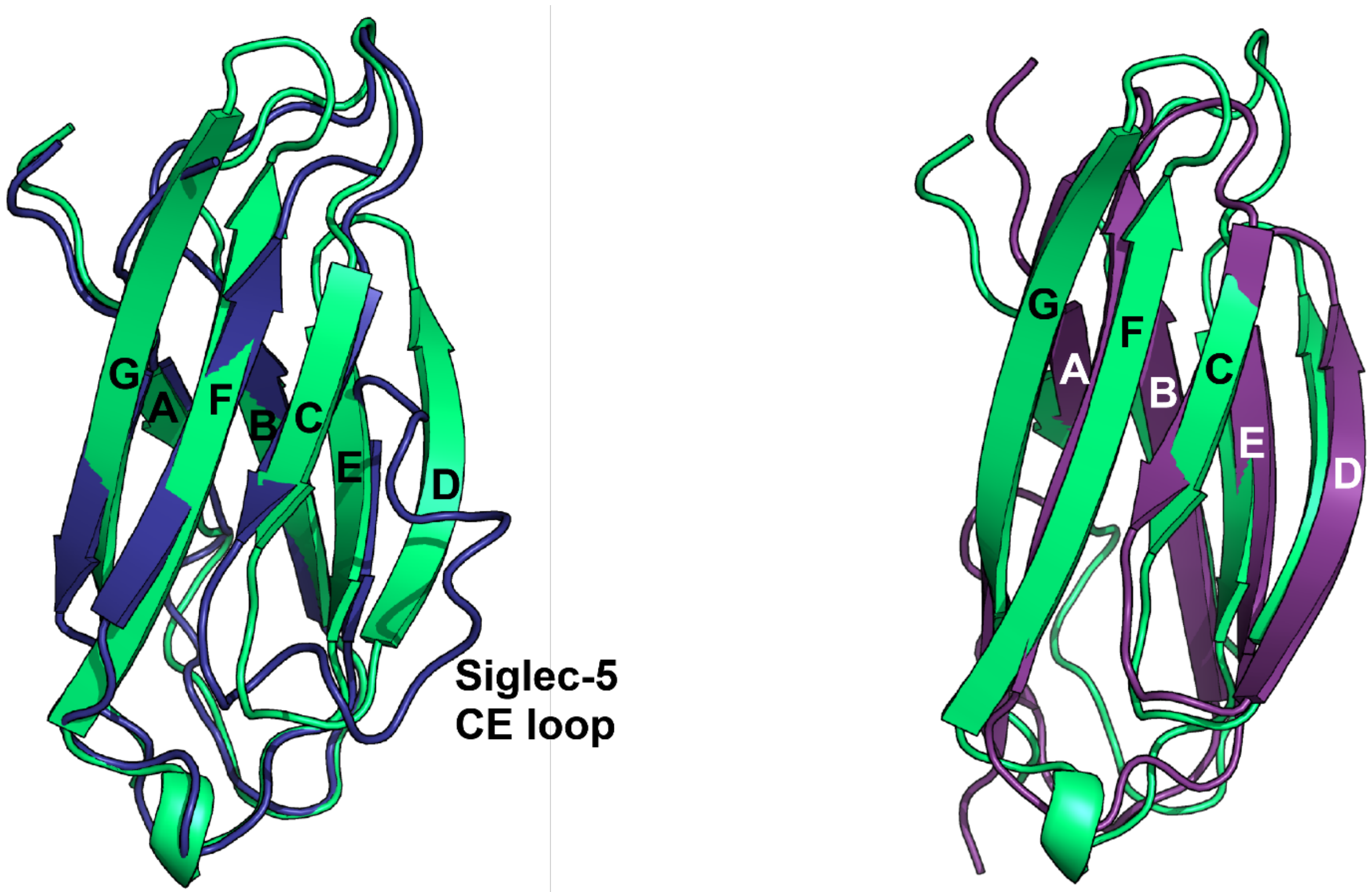

Siglec-5 **ICAM-3**

SUPPLEMENTARY FIGURE 1D

Details of the three *N*-linked GlcNAc residues built per chain

The protein chains at the three glycosylation sites, **i** – Asn100, **ii** – Asn160, **iii** – Asn209, are shown in cartoon form, with the Asn side chains and GlcNAc residues shown as sticks. Simulated annealing *mFo-DFc* omit maps (Asn and GlcNAc omitted) are displayed as a grey meshwork at 2.0 σ.

i

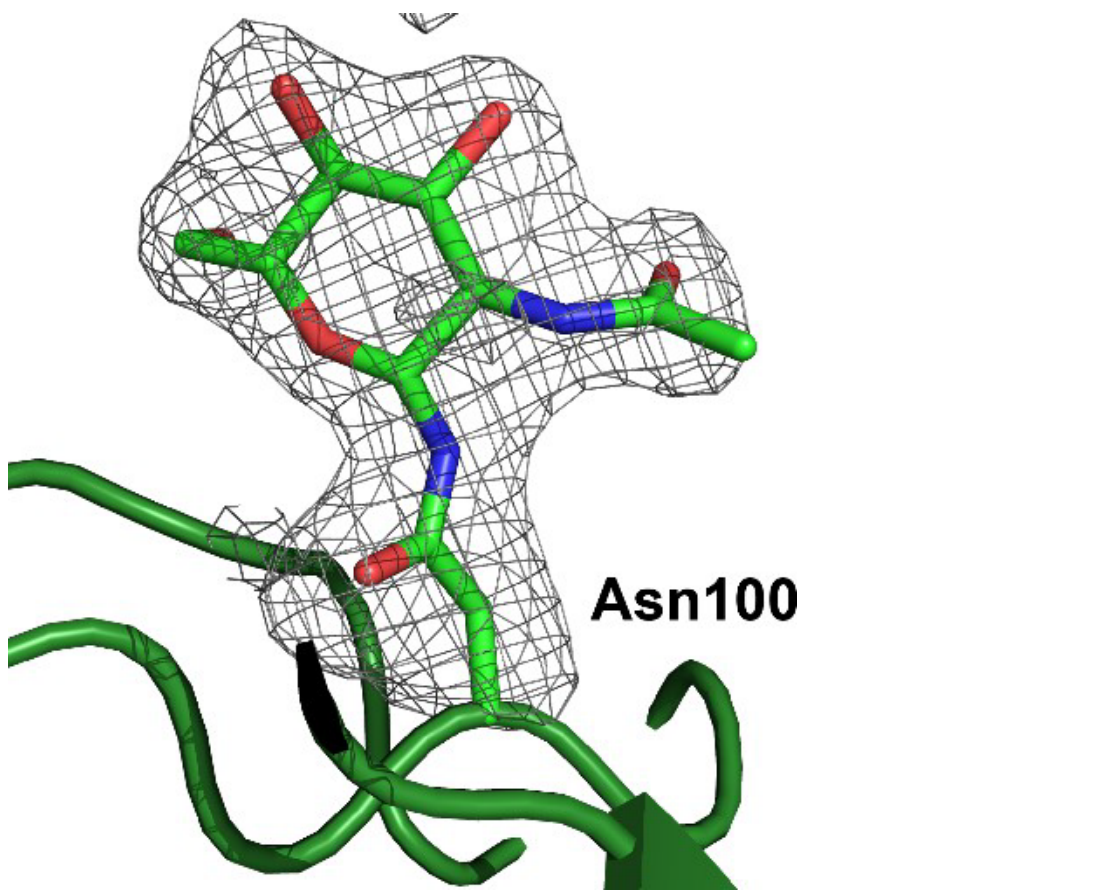

ii

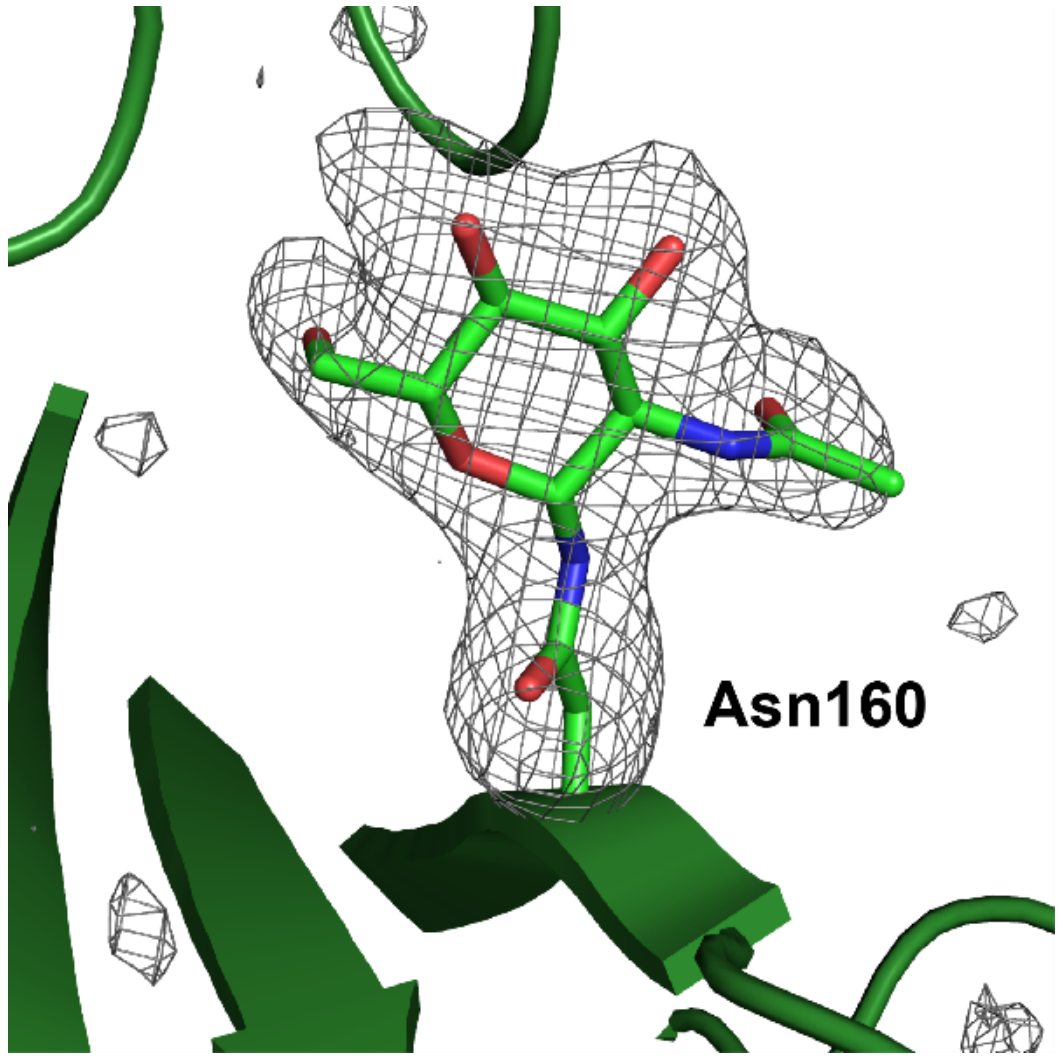

iii

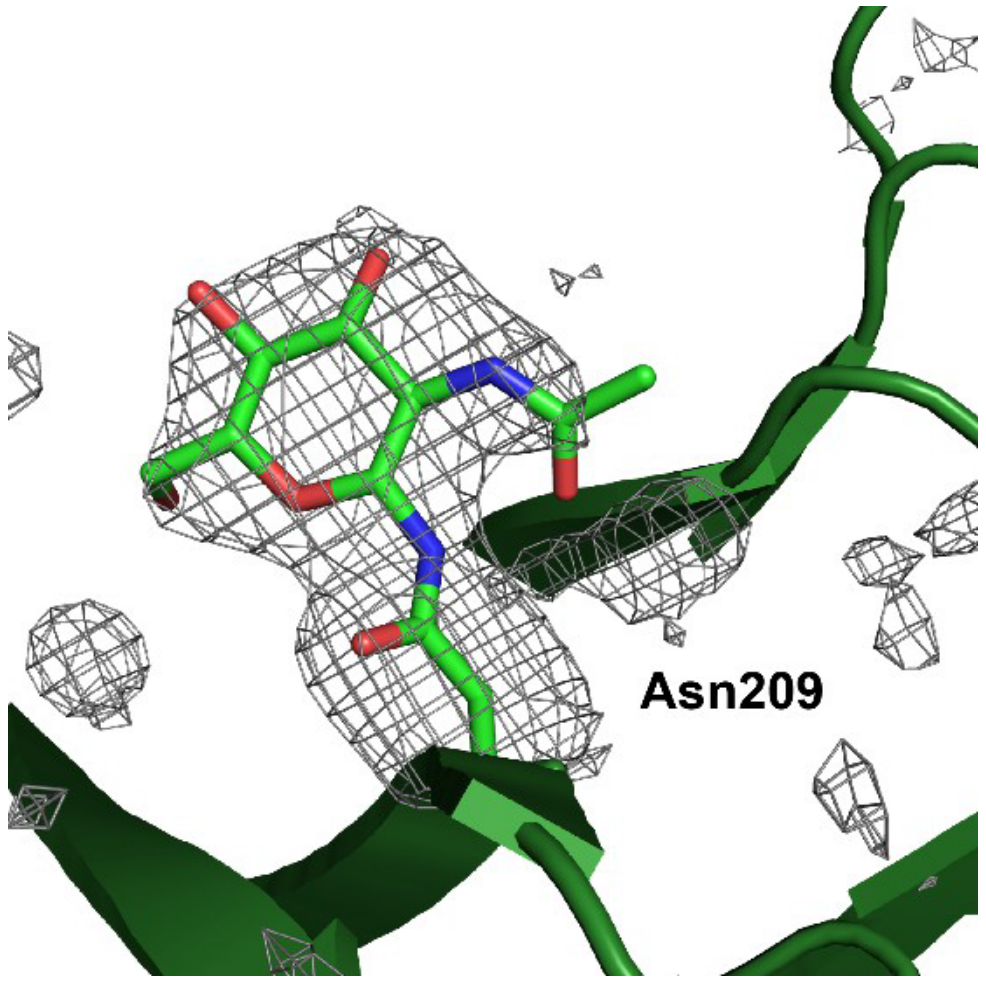

SUPPLEMENTARY FIGURE 1E

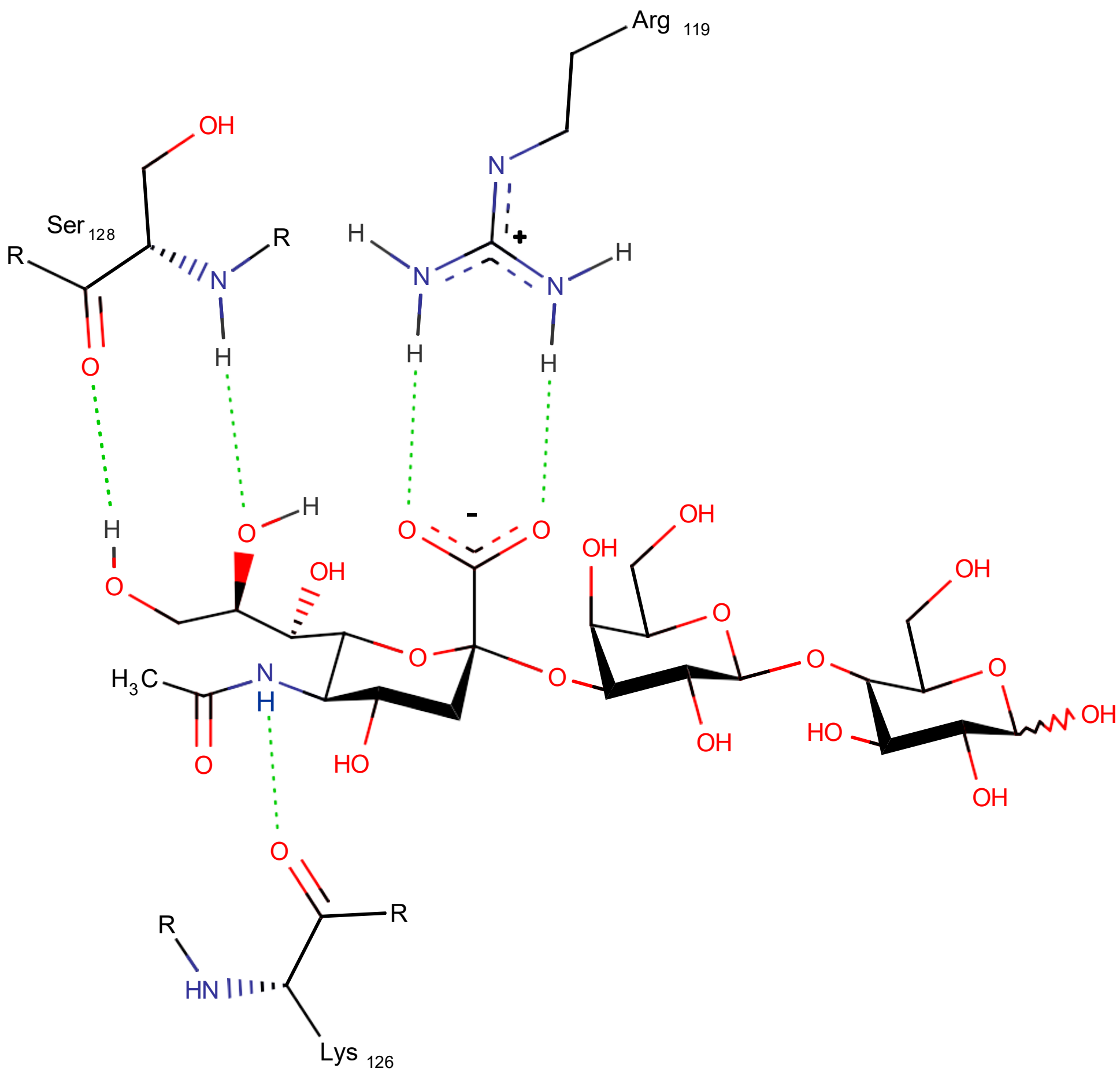

A schematic of the ligand binding site showing the interactions between CD33 and 3’-sialyllactose (identical interactions are seen for 6’-sialyllactose). No interactions are made with the galactose or glucose residues. Figure prepared using PoseView.

SUPPLEMENTARY FIGURE 1F

Top panel:Electron density maps for 3'-sialyllactose and 6'-sialyllactose ligands

The two ligand molecules (each from their respective chain B binding site) are shown in stick format. Simulated annealing *mFo-DFc* omit maps (ligand molecules omitted) are displayed as a grey meshwork at 2.0 σ.

3'-SL

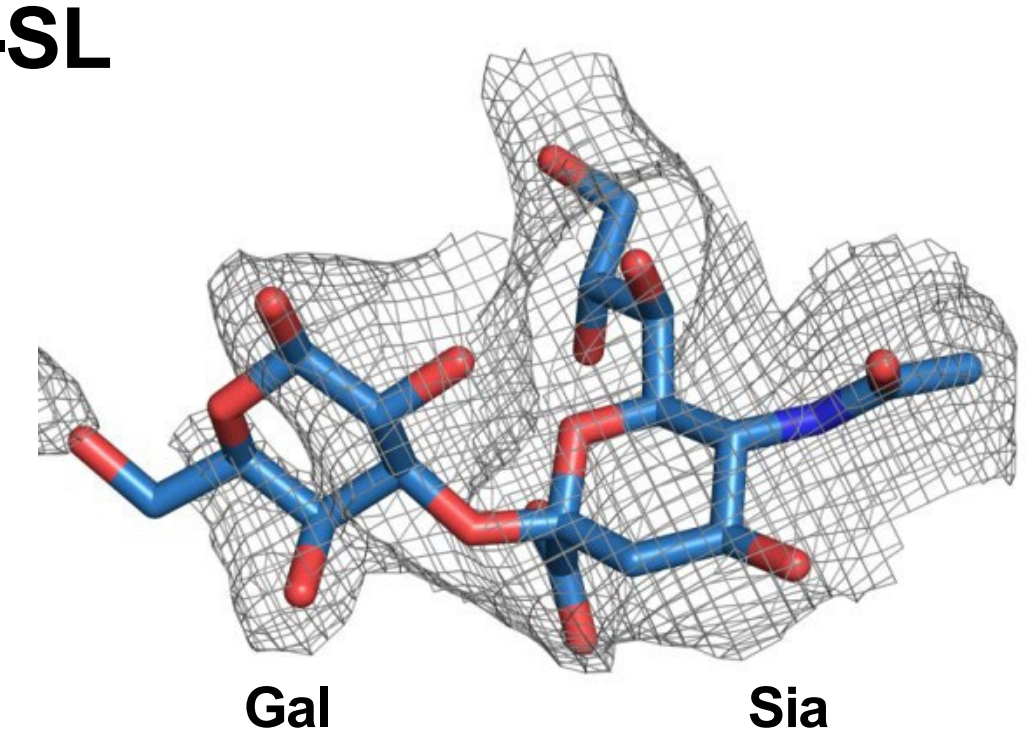

6'-SL

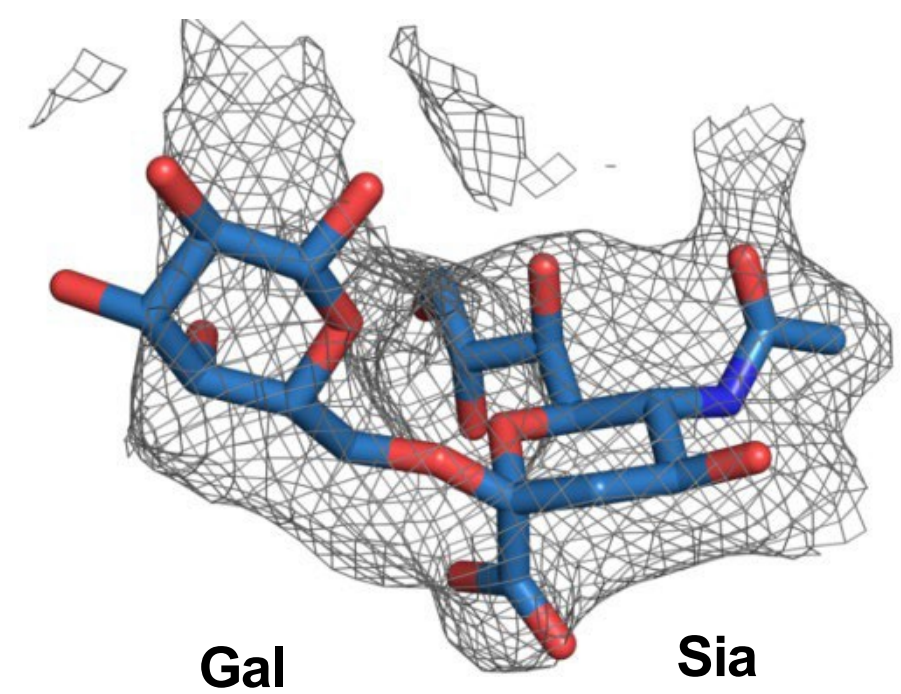

SUPPLEMENTARY FIGURE 1G

Sequence conservation of dimer interface residues in CD33 C2-set domains of multiple human Siglecs. This region was identified by the EPPIC program as likely to form biologically authentic dimer interfaces. Red arrows depict residues determined by EPPIC to have > 95% buried surface area in the interface, green arrows residues with > 70% buried surface area. These are highly conserved in a subset of the sequences.

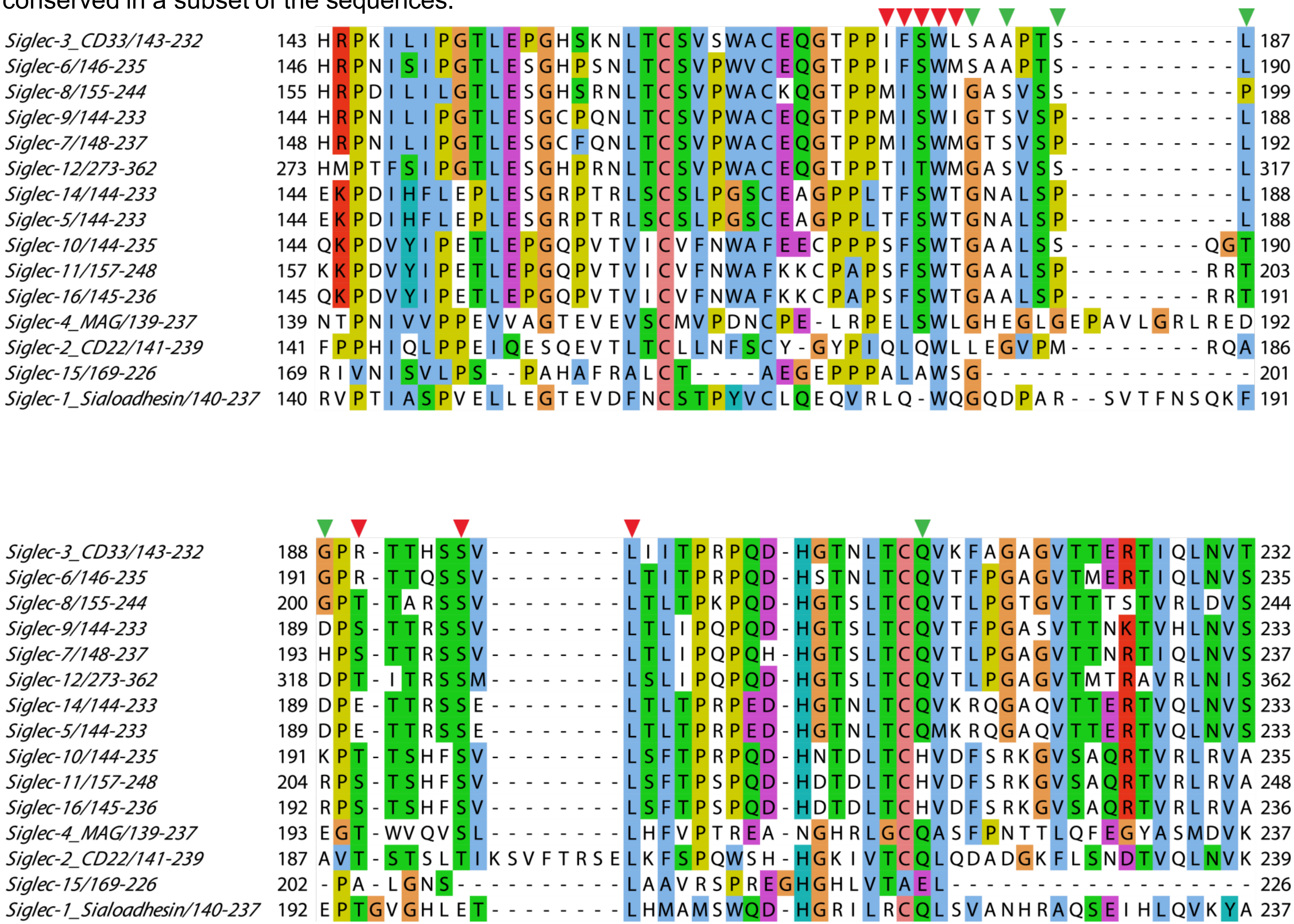

SUPPLEMENTARY FIGURE 1H

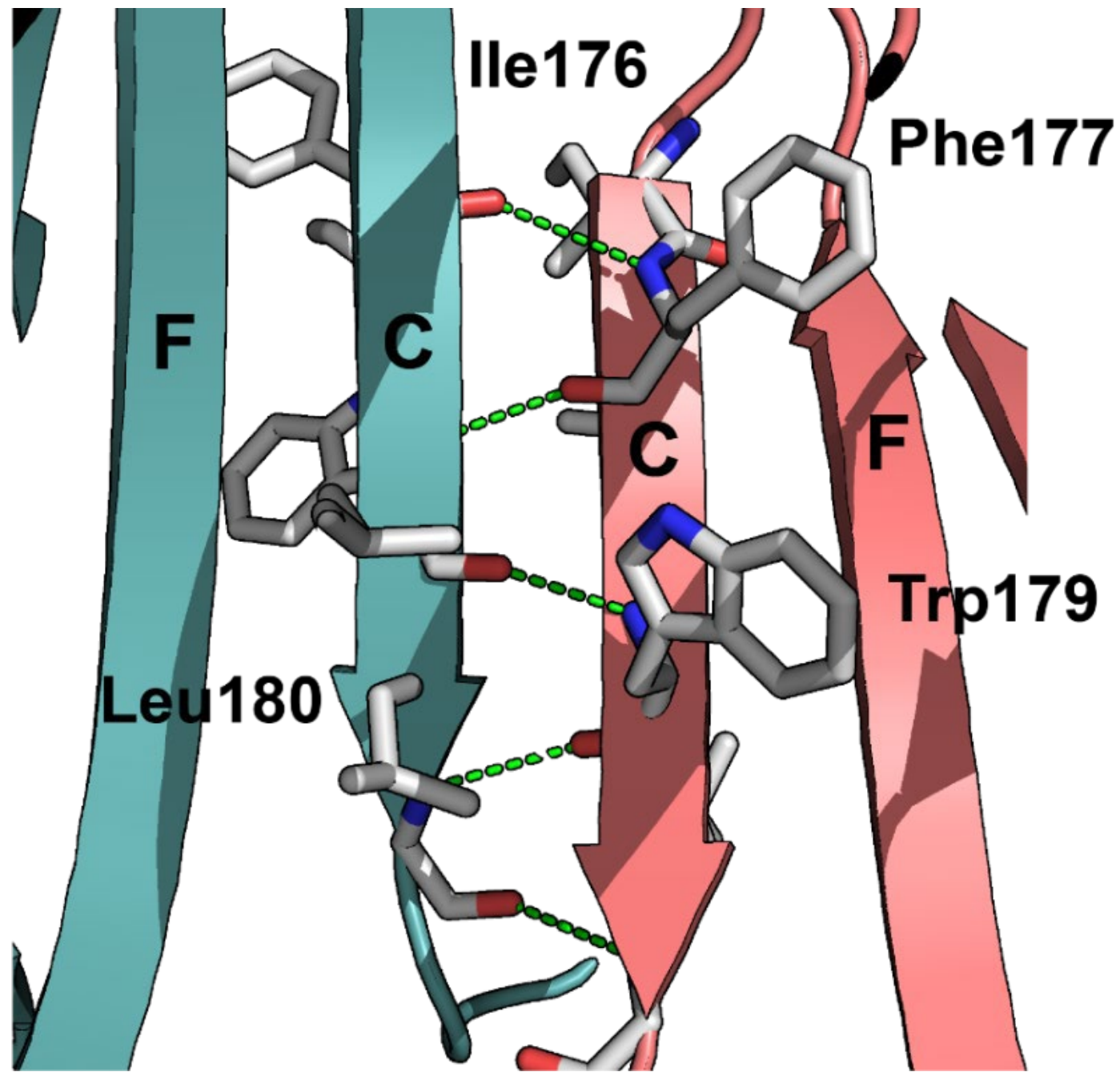

Detail of the β-strand interactions at the centre of the dimer interface, showing parallel arrangement of the two C-strands, with a series of main chain hydrogen bonds (green dotted lines), and with the side chains of several hydrophobic residues forming critical packing interactions (Leu176, Phe177, Trp179 and Leu180). Mutagenesis experiments show that all residues contribute to dimer formation.

SUPPLEMENTARY FIGURE 1I

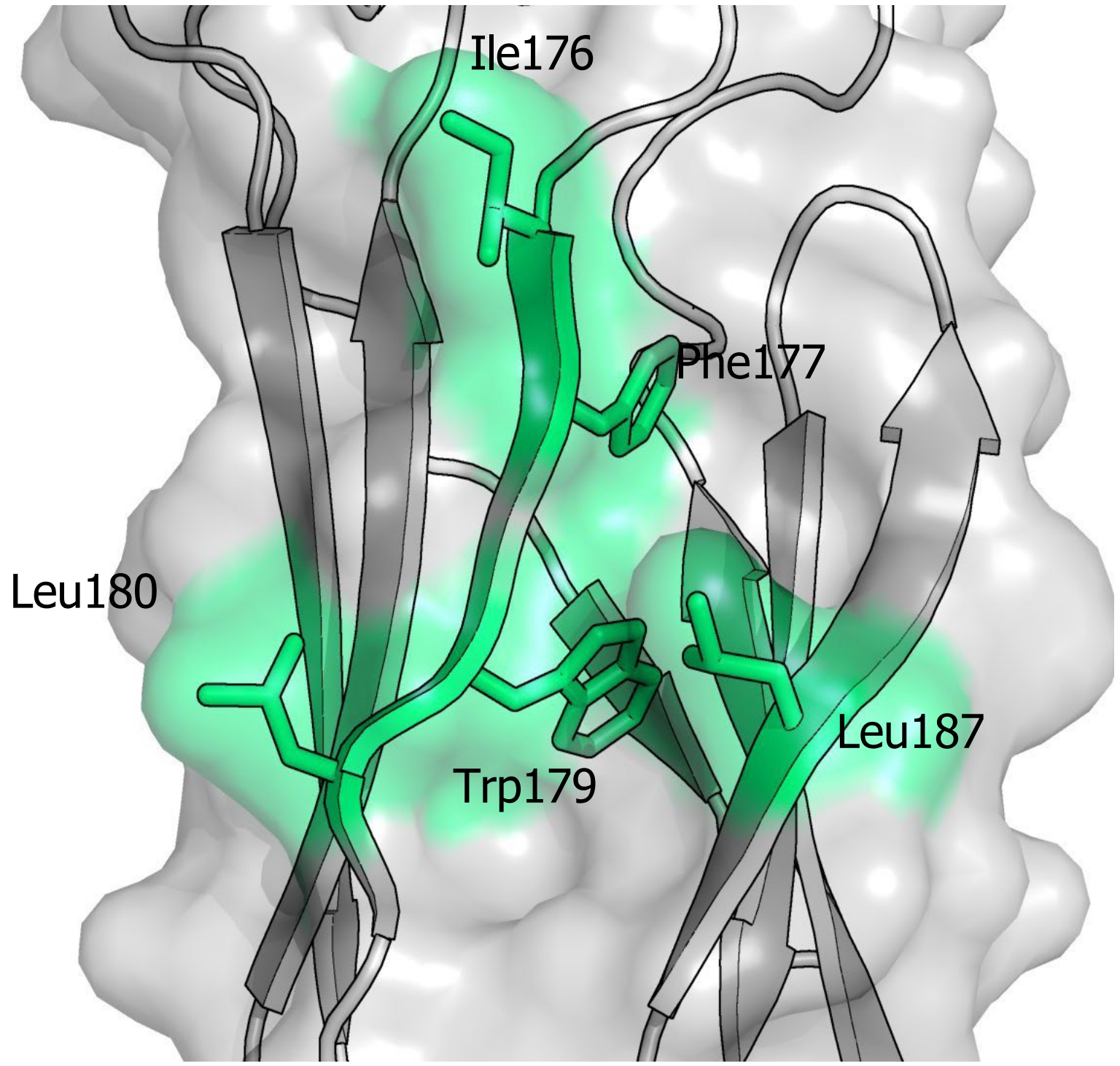

Detail of the hydrophobic residues interacting at the dimeric interface. The surface is coloured green for hydrophobic residues and grey for polar residues. The 5 hydrophobic side chains (Ile176, Phe177, Trp179, Leu180 and Leu187) are shown as sticks.

SUPPLEMENTARY FIGURE 1J

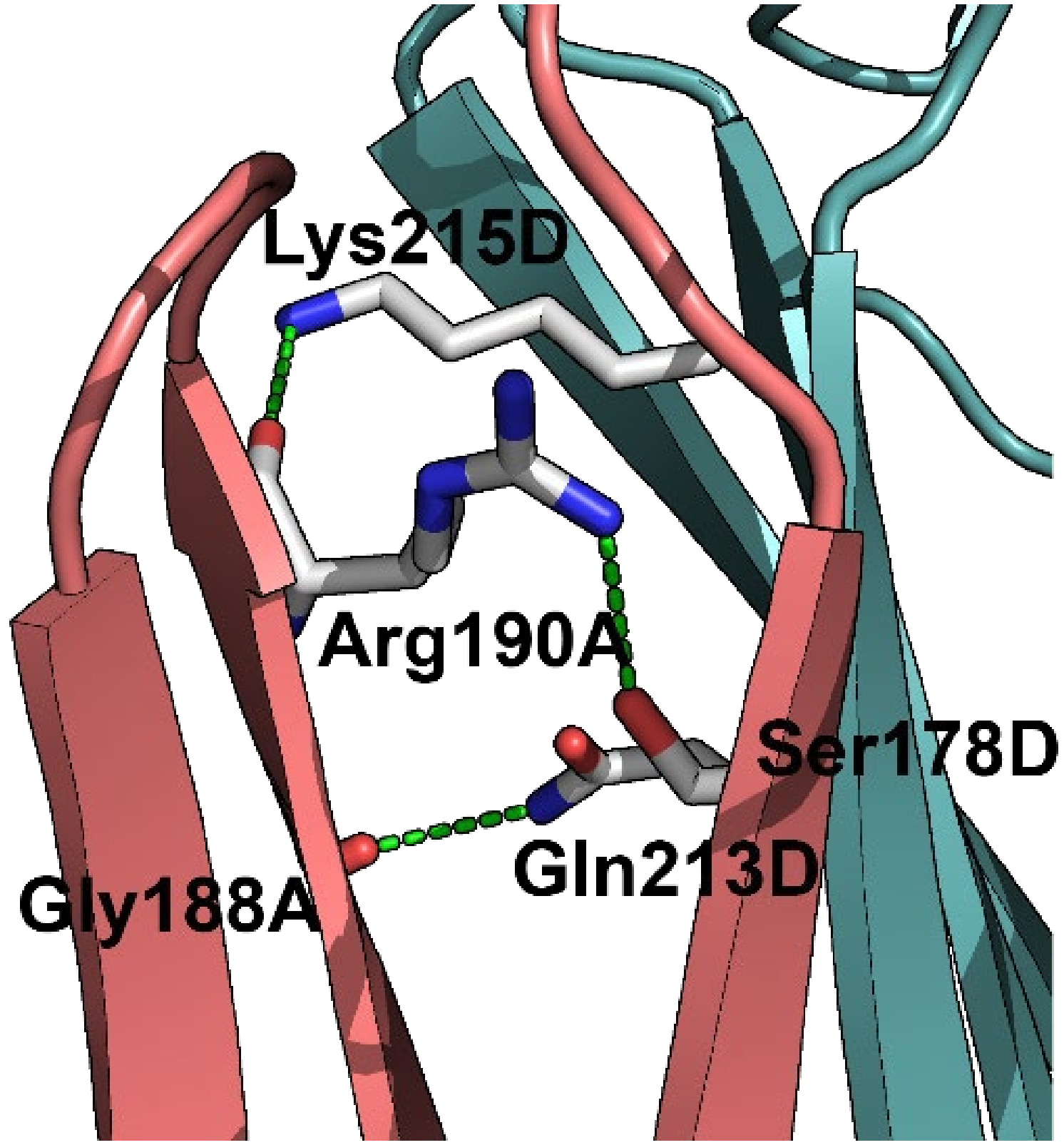

Detail of several hydrogen bonding interactions at the dimeric interface.
Interacting residues are shown as sticks.

SUPPLEMENTARY FIGURE 2A

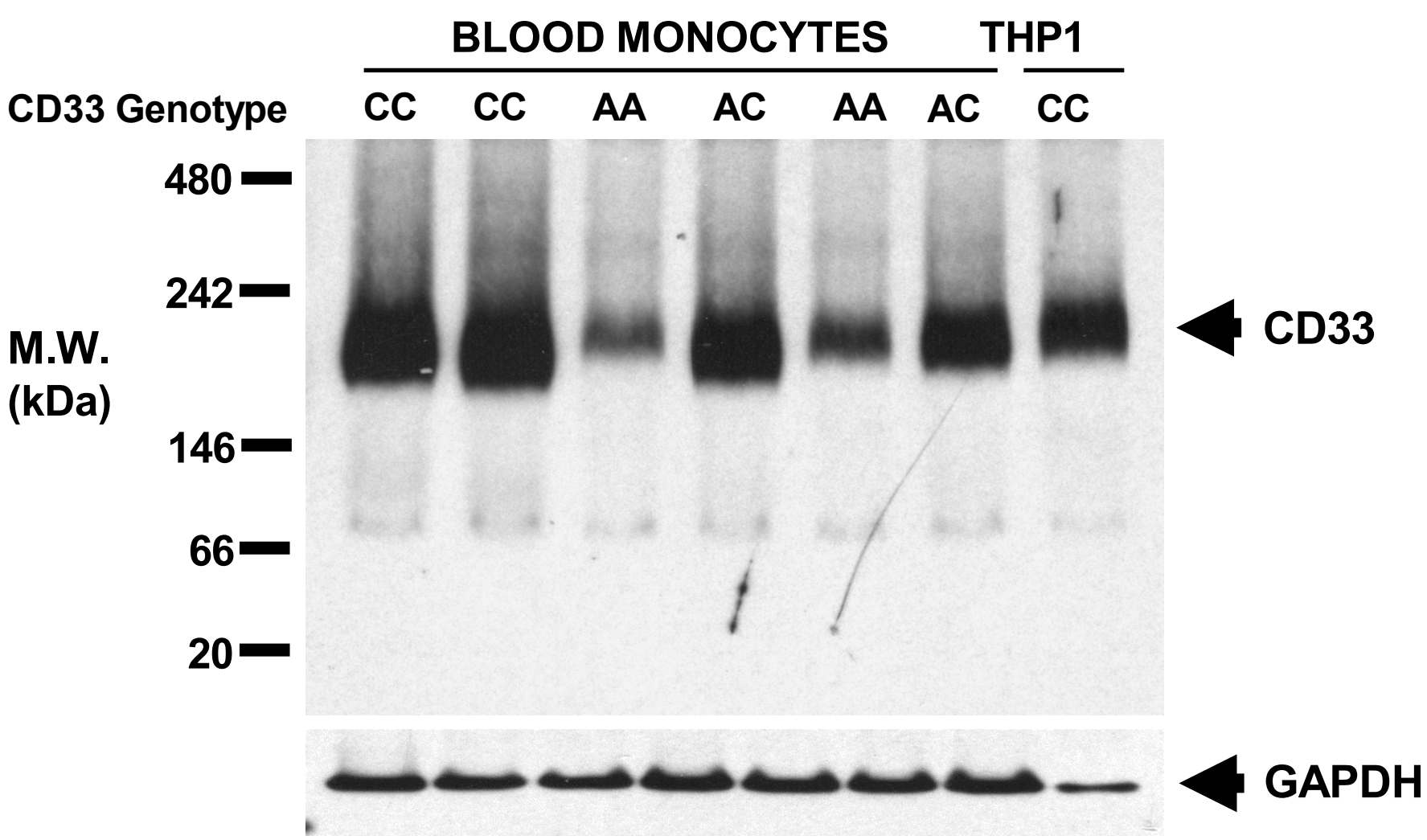

Biological confirmation of dimerization
Blue-Native PAGE analysis of digitonin-lysed native CD33 from fresh human peripheral blood monocytes and THP-1 cells. Western blotting with an anti-CD33 antibody demonstrates a species migrating in excess of the predicted monomeric size of 67-75 kDa.

SUPPLEMENTARY FIGURE 2B

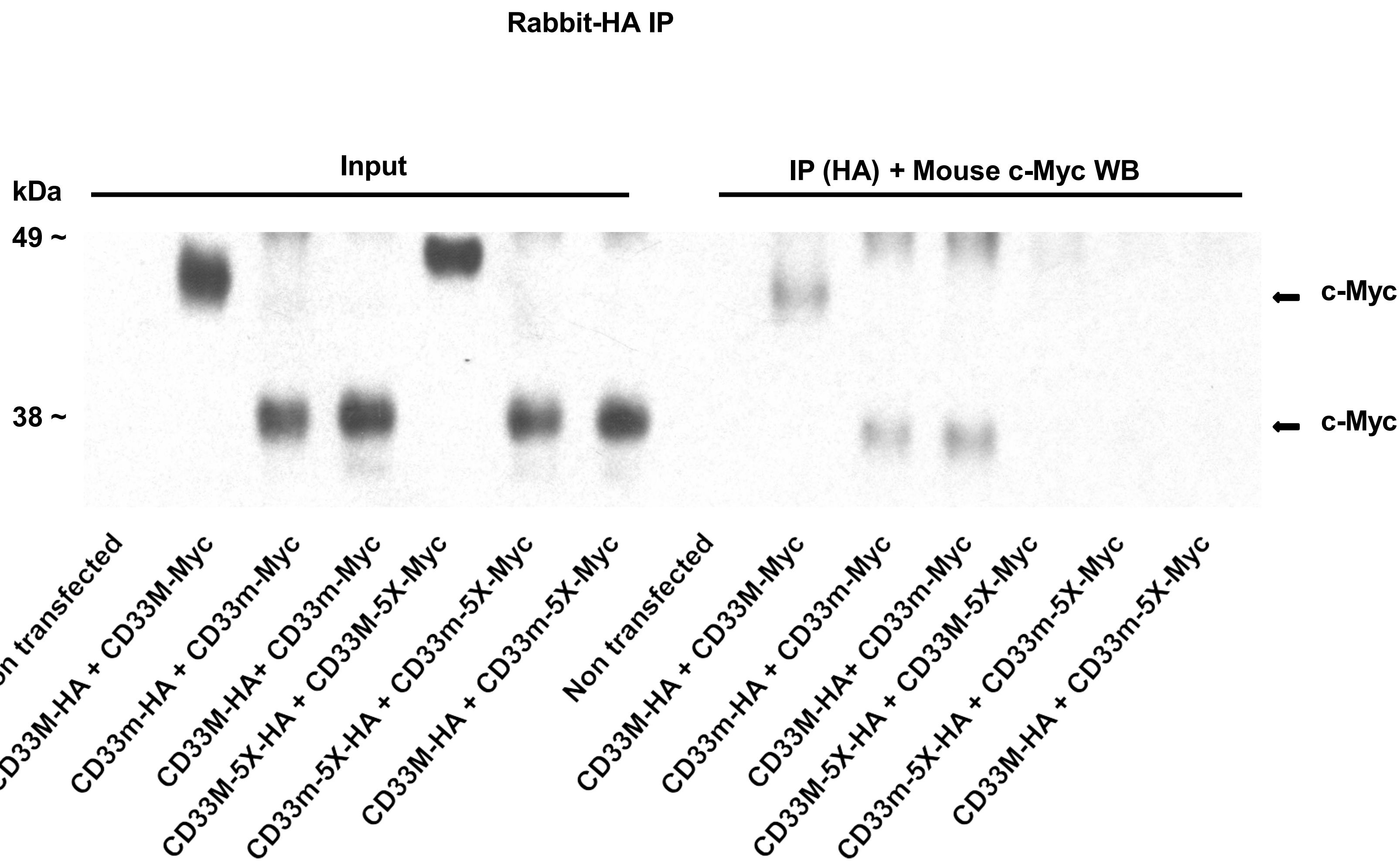

Mutagenesis of Phe177Ala + Trp179Ala + Leu180Ala + Arg190Ala + Gln213Ala residues within the dimer site was performed to disrupt the hydrophobic and polar interactions in the dimer site. Mutation of any combination of 1-4 of these 5 residues had no effect on dimer formation. However. mutation of all 5 residues resulted in formation of stable, fully glycosylated $CD33^{M}$ and $CD33^{m}$ monomers that do not co-precipitate other CD33 peptides

SUPPLEMENTARY FIGURE 2C

SEC-MALS – Endo H_f-treated CD33 ECD

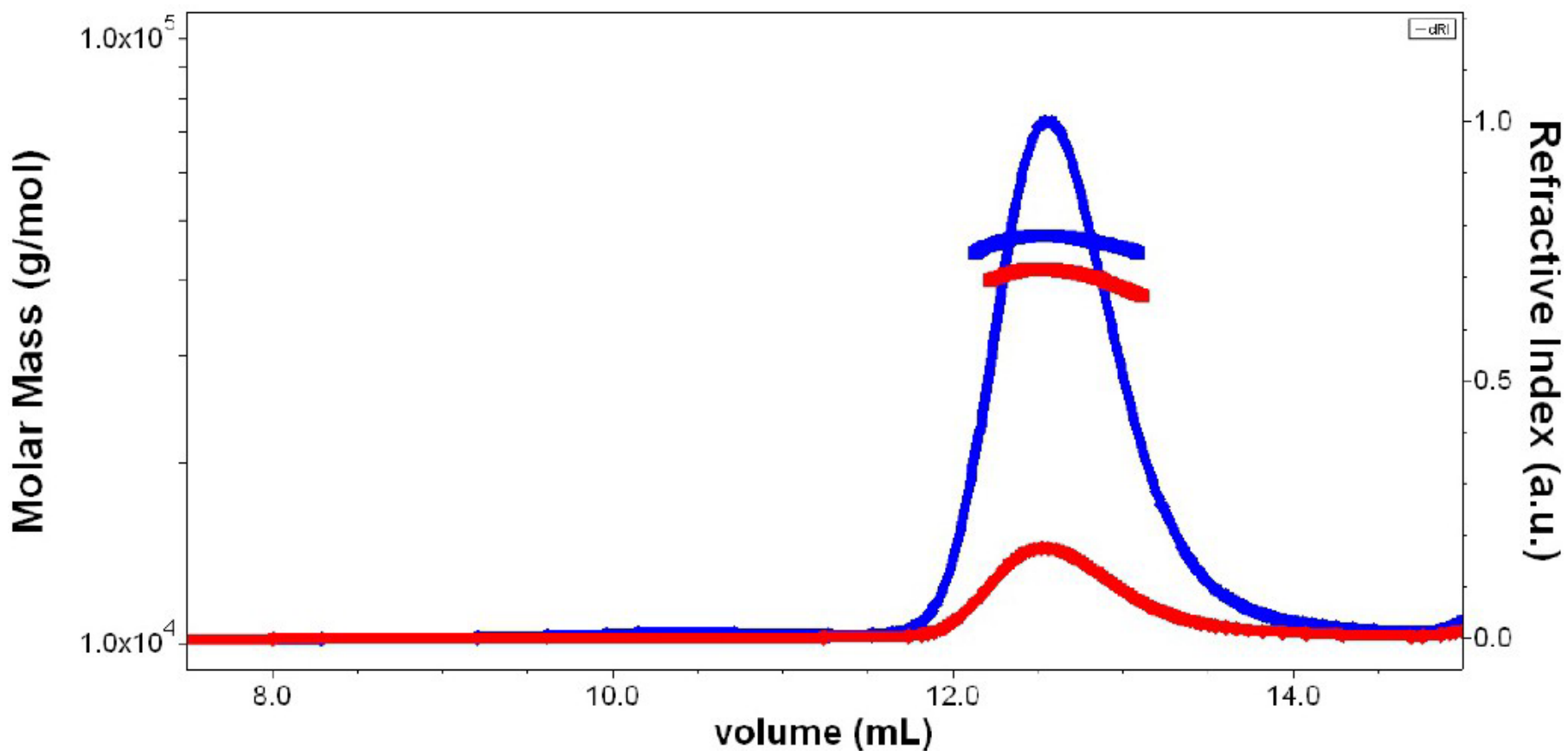

SEC-MALS – Glycosylated CD33 ECD

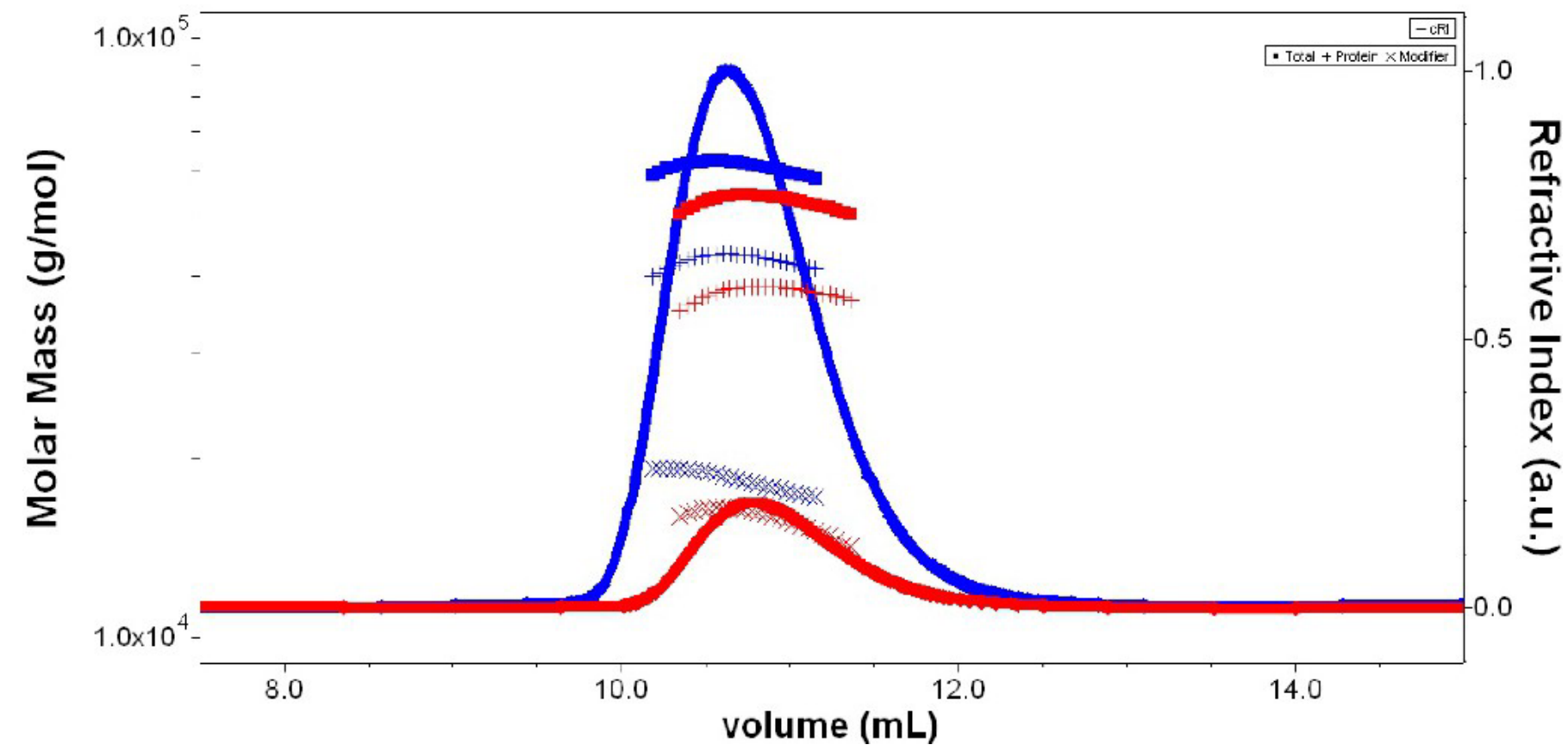

QELS measurements (DLS)

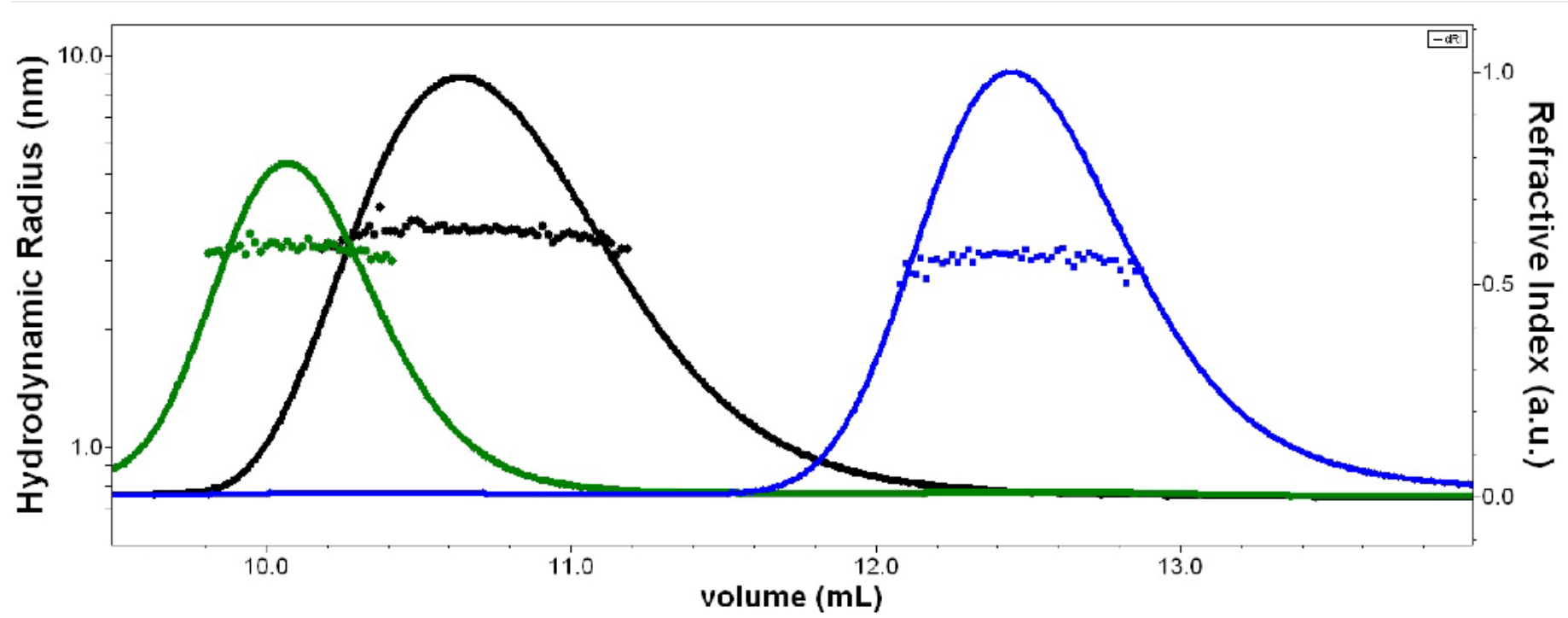

SEC-MALS demonstrates CD33 ECD dimers in solution – see legend in next panel.

LEGEND FOR SUPPLEMENTARY FIGURE 2C

SEC-MALS demonstrates CD33 ECD dimers in solution

Left Panel **- SEC-MALS analysis of Endo H_f-trimmed CD33 ECD**. Shown are plots of refractive index (RI, right axis) and evaluated molar mass (left axis) against elution volume for samples loaded at 2.5 mg/ml (blue) and 0.5 mg/ml (red). The glycan-trimmed sample were considered as all protein but still analysed using either UV and RI as concentration signals. At the higher concentration (~ 10 µM on column), RI indicates a mass of 47 kDa over the central 50 % of the peak while analysis using UV absorbance as the concentration source gives a mass of 50 kDa. These results confirm that CD33 ECD is present predominantly as a dimer in solution at the concentrations achieved over the central 50% of the SEC peak (~ 5-10 µM). At the lower concentration loaded (~ 2 µM on column) the apparent mass is slightly lower at 41 kDa and 44kDa (RI, UV). Consequently we can estimate that the Kd for this dimerisation is much below µM and probably on the order of 100s of nM.

Right Panel **- SEC-MALS of fully glycosylated CD33 ECD**. As for the trimmed sample, protein was loaded at 2.5 mg/ml (blue) and 0.5 mg/ml (red). The glycosylated samples eluted overall earlier than the Endo H-trimmed samples consistent with a higher expected mass and larger hydrodynamic properties contributed by the additional carbohydrate. Due to the significant contribution of glycans to the mass of the system, a dual concentration conjugate analysis was applied (*dn/dc* protein 0.1885 ml g^{-1}, *dn/dc* carbohydrate 0.145 ml g^{-1}, UV protein 1252 ml g^{-1} cm^{-1}, UV carbohydrate no absorbance). This analysis evaluates a protein mass of about 43 kDa (symbol +) and a carbohydrate mass of about 18 kDa (symbol X) consistent with dimer formation and with the predicted mass of the carbohydrate modifications on CD33 giving a total mass around 60 kDa (symbol filled square).

Bottom Panel **- QELS measurements (DLS)** made for the SEC-MALS samples injected at 2.5 mg/ml. The hydrodynamic radius calculated for glycosylated CD33 ECD (black) of 3.5 nm is larger than expected for a 50kDa dimeric protein (for example, larger than that of the BSA standard (green) which is a 66 kDa protein) consistent with the presence of the extensive carbohydrate modifications $GlcNAc_2Man_9$ at 5 positions. Endo H_f-trimmed CD33 ECD (blue) has an Rh of 3.0 nm, which is more consistent with a simple globular protein of 50 kDa mass as expected following the almost complete removal of its carbohydrate adducts.

SUPPLEMENTARY FIGURE 2D

Analytical Ultracentrifugation (AUC) Sedimentation Velocity (SV) Experiment

Raw data from AUC SV scans for deglycosylated CD33.

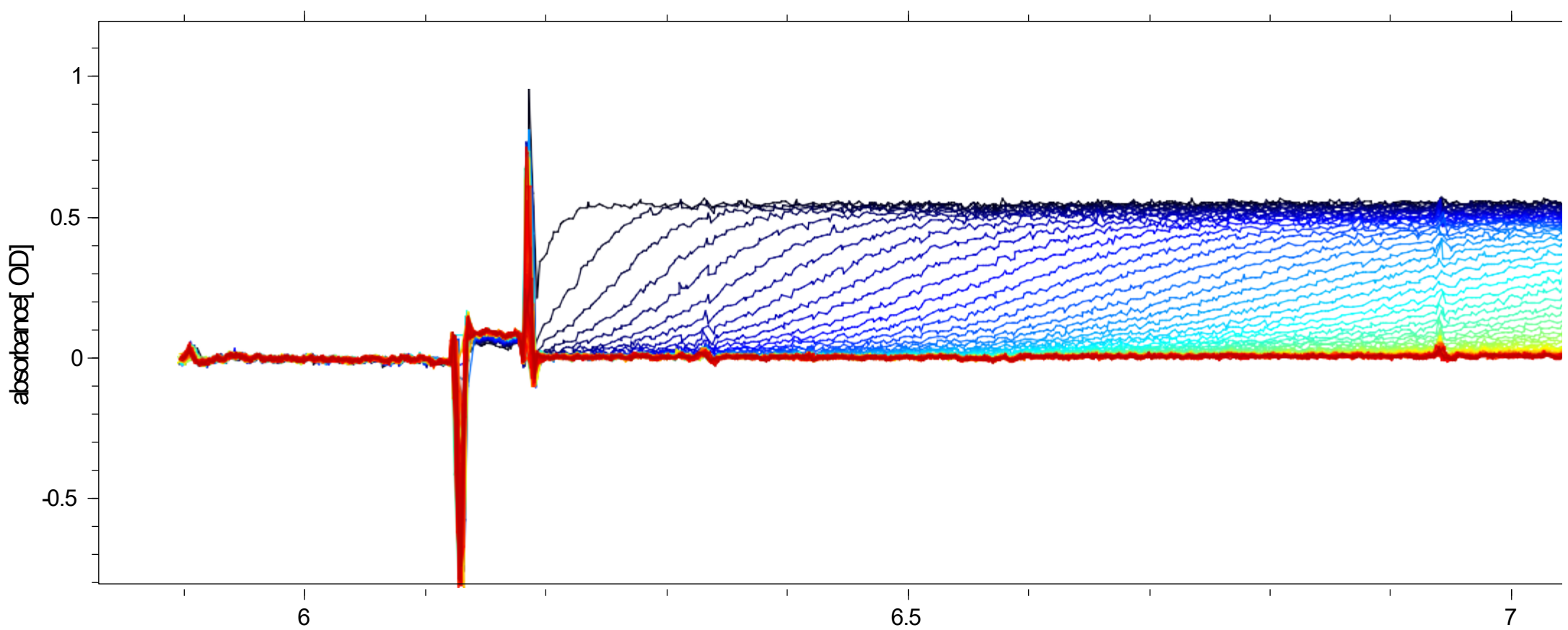

Dimerization Kd = 0.70 μM

C(s) distribution for deglycosylated CD33.

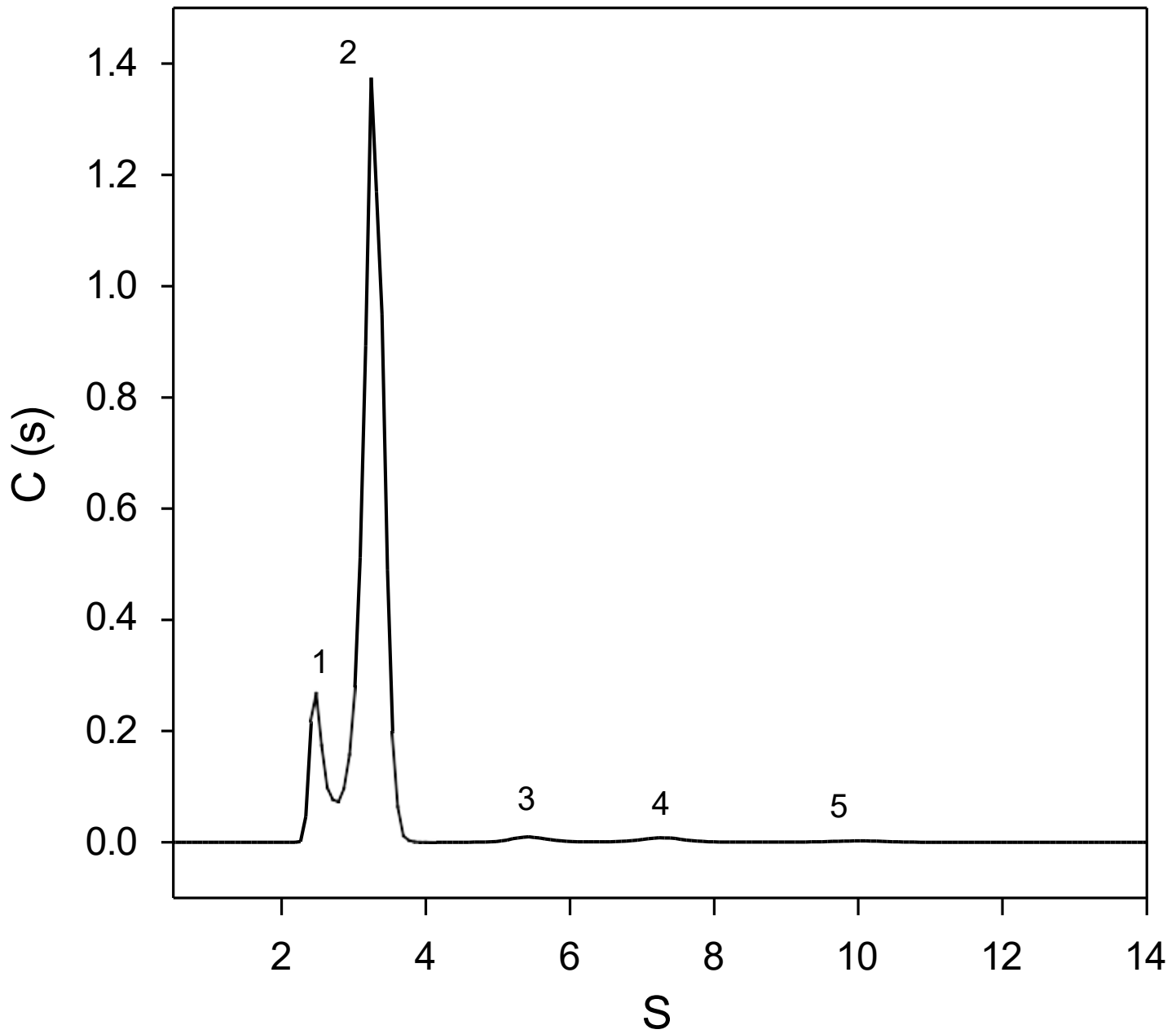

	S	S (20w)	%	app. Mw (kDa)
1	2.548	2.705	13.5	31.6
2	3.261	3.462	83.9	45.7
3	5.481	5.819	1.1	99.6
4	7.272	7.720	1.1	152.1
5	9.935	10.546	0.4	243.0

Sedimentation velocity (SV) analysis CD33 dimerization was performed in a ProteomeLab XL-I (BeckmanCoulter) analytical ultracentrifuge. Protein samples were loaded in 2-channel cells and centrifuged in an An-50 Ti 8-place rotor at 40,000 rpm, 20 °C for 15 hours. Absorbance at 280 nm was used for detection. The samples contained 0.4 mg/ml glycosylated or deglycosylated CD33 ECD, 20 mM HEPES, 200 mM NaCl, pH 7.5. SV data were analysed using Sedfit software (P. Schuck, NIH/NIBIB). Raw data is available in Excel sheets

SUPPLEMENTARY FIGURE 2E

Analytical Ultracentrifugation (AUC) Sedimentation Velocity (SV) Experiment

Raw data from selected AUC SV scans for glycosylated CD33.

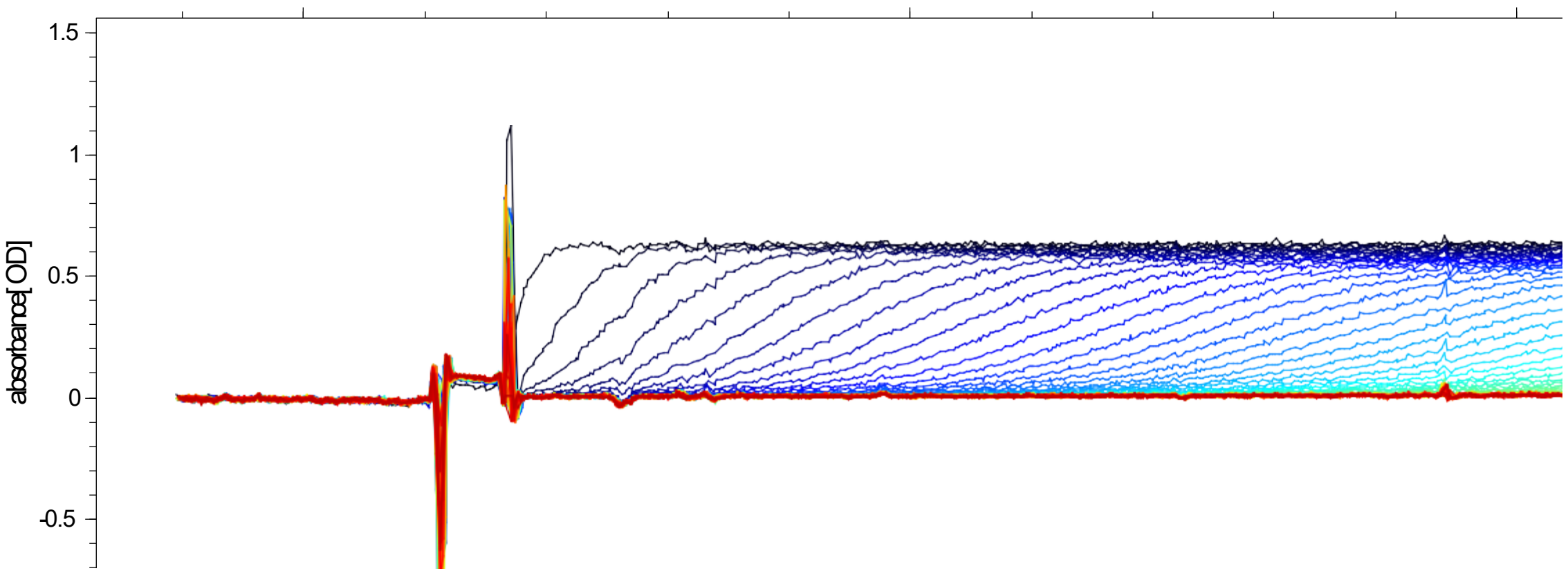

C(s) distribution for glycosylated

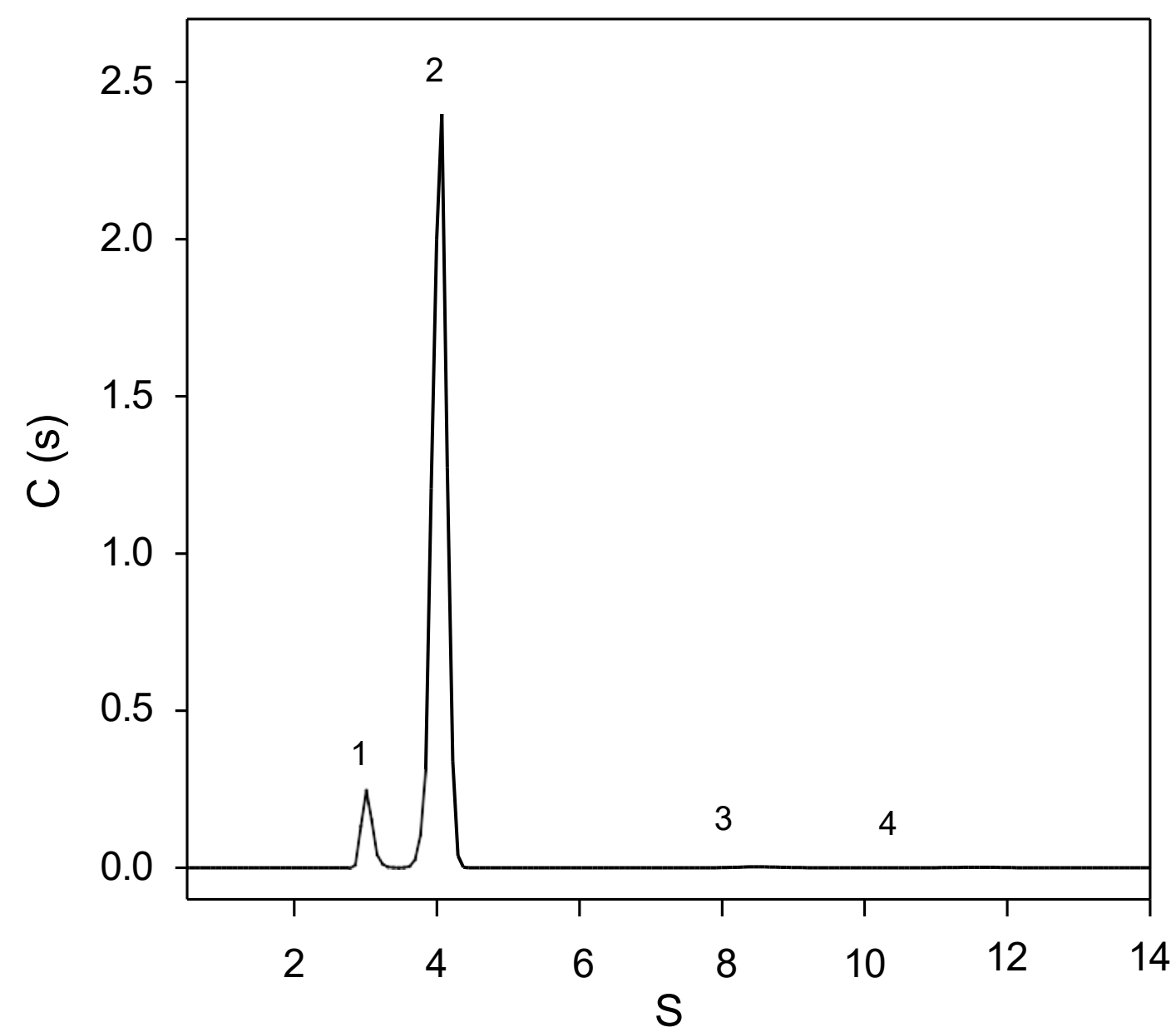

Dimerization Kd = 0.19 μM

	S	S (20w)	%	app. Mw (kDa)
1	3.034	3.214	7.3	37.0
2	4.033	4.271	92.2	56.6
3	8.543	9.048	0.3	174.6
4	11.560	12.244	0.2	274.7

Sedimentation velocity (SV) analysis CD33 dimerization was performed in a ProteomeLab XL-I (BeckmanCoulter) analytical ultracentrifuge. Protein samples were loaded in 2-channel cells and centrifuged in an An-50 Ti 8-place rotor at 40,000 rpm, 20 °C for 15 hours. Absorbance at 280 nm was used for detection. The samples contained 0.4 mg/ml glycosylated or deglycosylated CD33 ECD, 20 mM HEPES, 200 mM NaCl, pH 7.5. SV data were analysed using Sedfit software (P. Schuck, NIH/NIBIB). Raw data is available in Excel sheets

LEGENDS FOR SUPPLEMENTARY FIGURES 2F-I

Sedimentation equilibrium (SE) experiments to analyse CD33 dimerization were performed in a ProteomeLab XL-I (Beckman Coulter) analytical ultracentrifuge. Samples of glycosylated and deglycosylated CD33 ECD at concentrations 0.2, 0.1 and 0.05 mg/ml were loaded in 6-channel equilibrium cells and centrifuged in an An-50 Ti 8-place rotor at 16,000 or 20,000 rpm and 20 °C until equilibrium was reached for each speed. Sample buffer was 20 mM HEPES, 200 mM NaCl, pH 7.5. SE data were analysed using HeteroAnalysis software (by J.L. Cole and J.W. Lary, University of Connecticut).

SUPPLEMENTARY FIGURE 2F

Analytical Ultracentrifugation (AUC) Sedimentation Equilibrium (SE) Experiment

AUC SE raw data (black) from 16,000 rpm run are shown for 0.2, 0.1 and 0.05 mg/ml deglycosylated CD33 (from left to right). Global fit (blue) was used to determine dimerization Kd assuming Mw=25.0 kDa for monomer .

Dimerization Kd= 0.72 µM

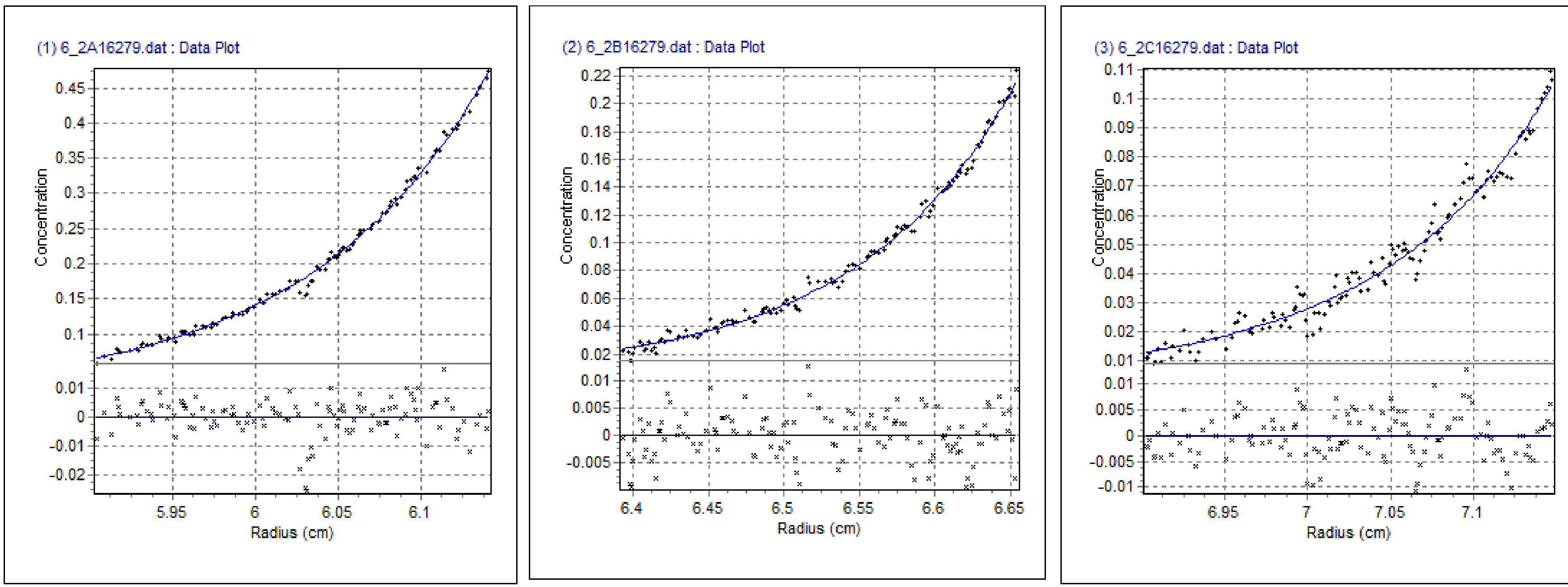

SUPPLEMENTARY FIGURE 2G

Analytical Ultracentrifugation (AUC) Sedimentation Equilibrium (SE) Experiment

AUC SE raw data (black) from 20,000 rpm run are shown for 0.2, 0.1 and 0.05 mg/ml deglycosylated CD33 (from left to right). Global fit (blue) was used to determine dimerization Kd assuming Mw=25.0 kDa for monomer .

Dimerization Kd= 0.72 μM

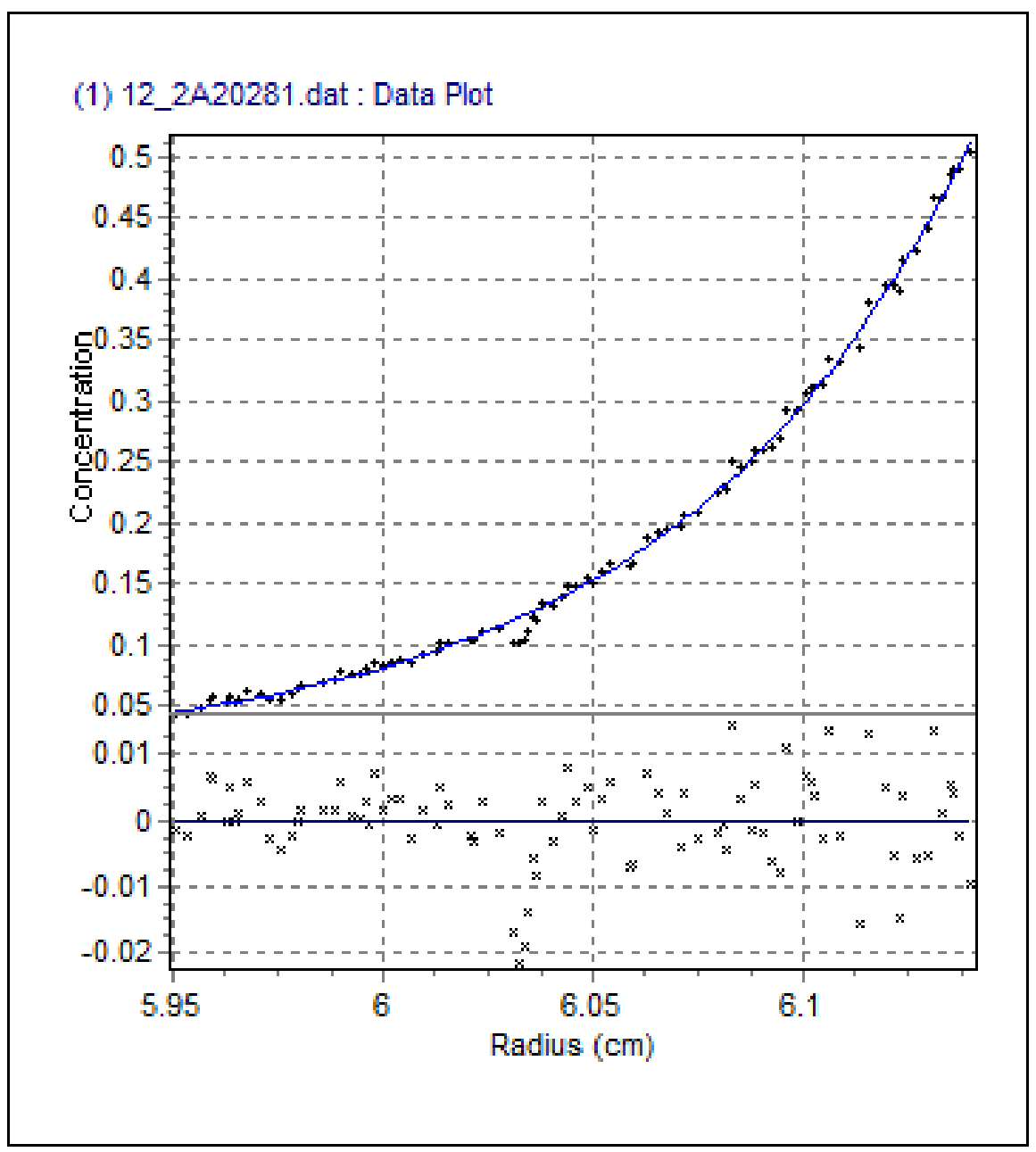

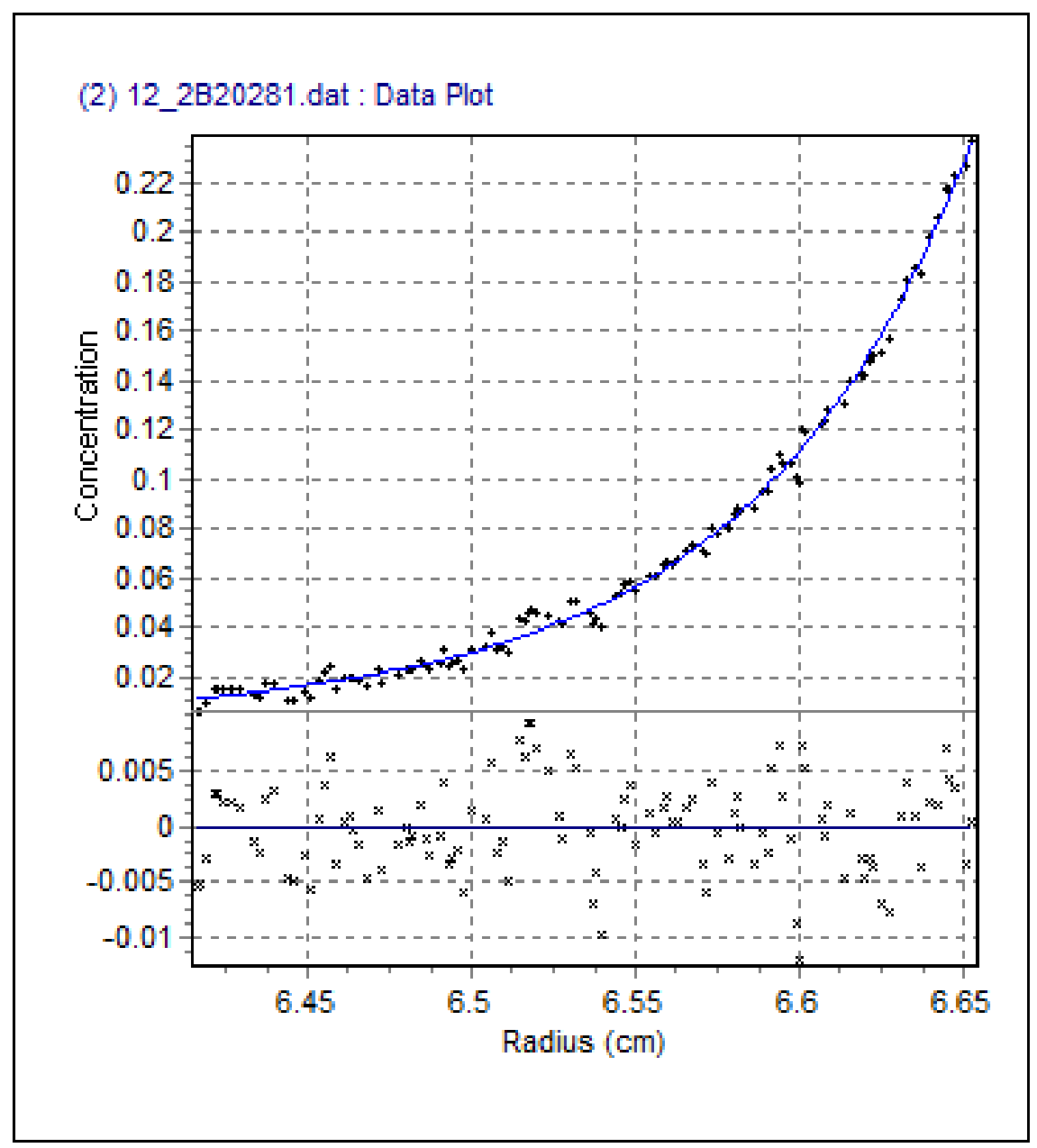

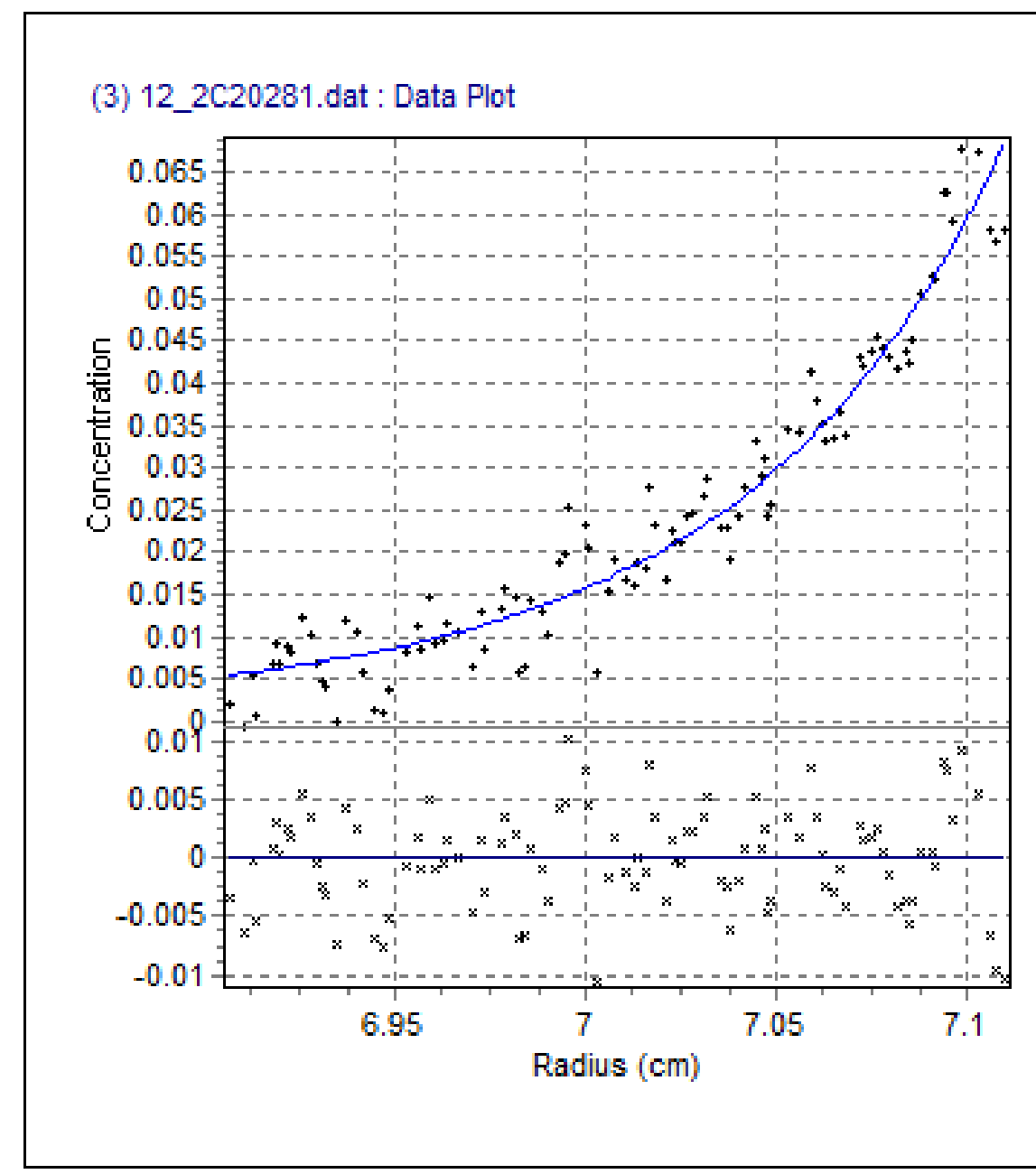

SUPPLEMENTARY FIGURE 2H

Analytical Ultracentrifugation (AUC) Sedimentation Equilibrium (SE) Experiment

AUC SE raw data (black) from 16,000 rpm run are shown for 0.2, 0.1 and 0.05 mg/ml glycosylated CD33 (from left to right). Global fit (blue) was used to determine dimerization Kd assuming Mw=31.3 kDa for monomer .

Dimerization Kd= 0.53 µM

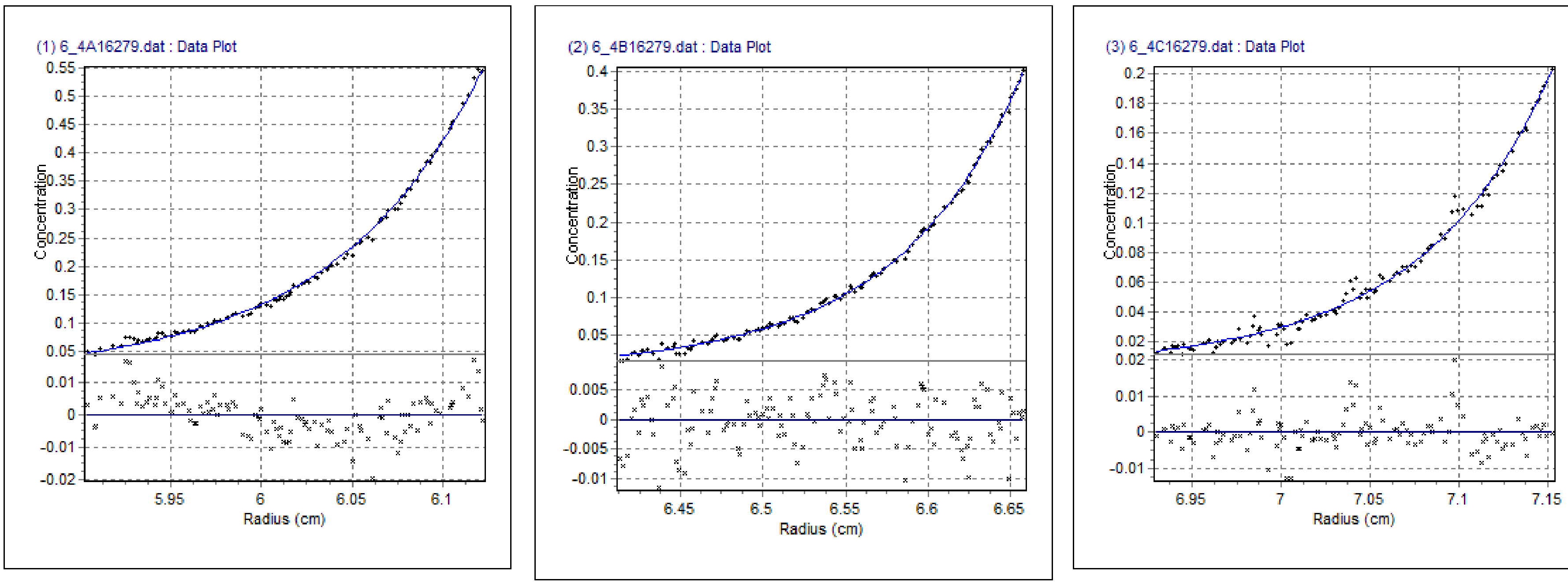

SUPPLEMENTARY FIGURE 2I

Analytical Ultracentrifugation (AUC) Sedimentation Equilibrium (SE) Experiment

AUC SE raw data (black) from 20,000 rpm run are shown for 0.2, 0.1 and 0.05 mg/ml glycosylated CD33 (from left to right). Global fit (blue) was used to determine dimerization Kd assuming Mw=31.3 kDa for monomer .

Dimerization Kd= 0.22 µM

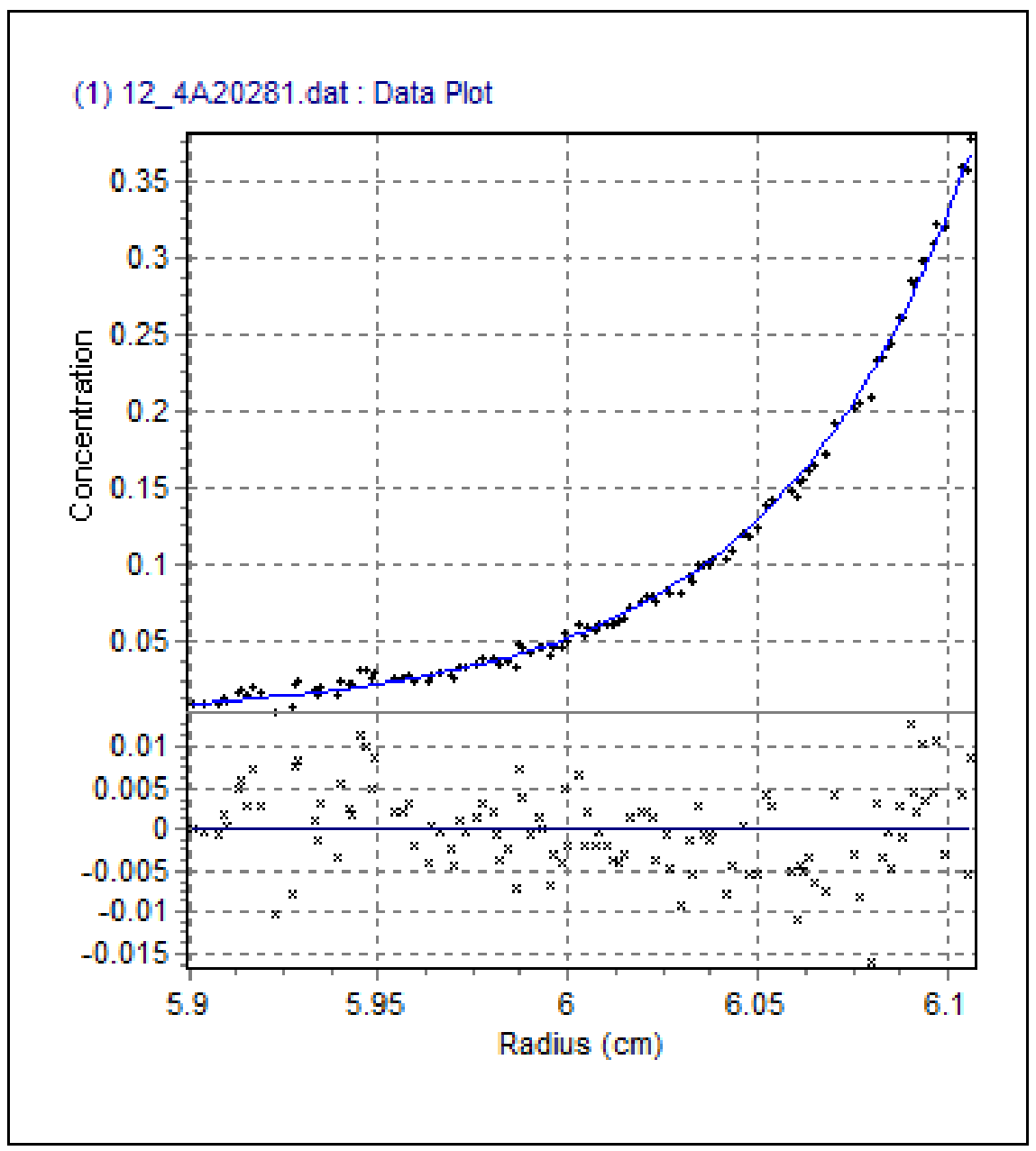

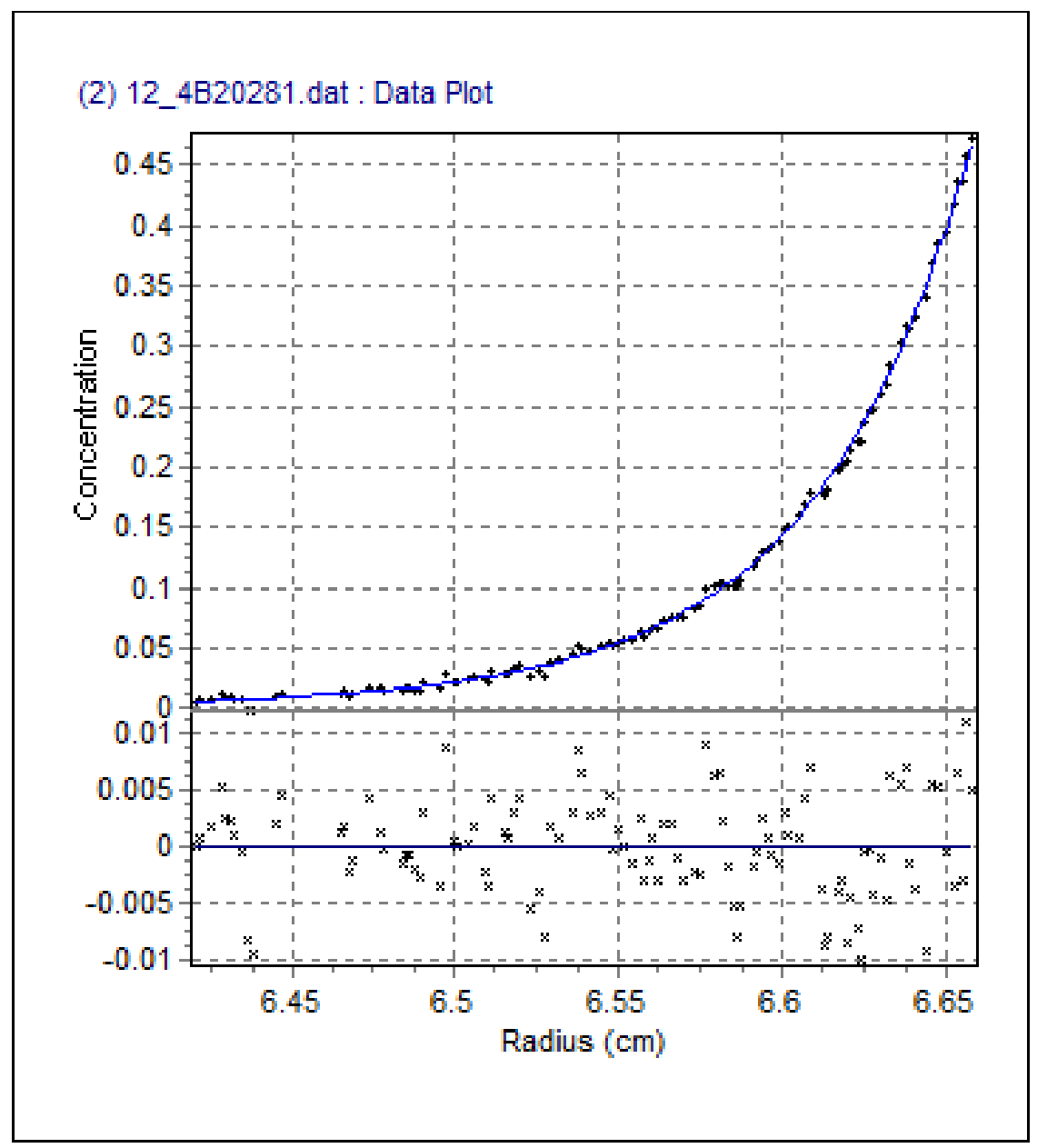

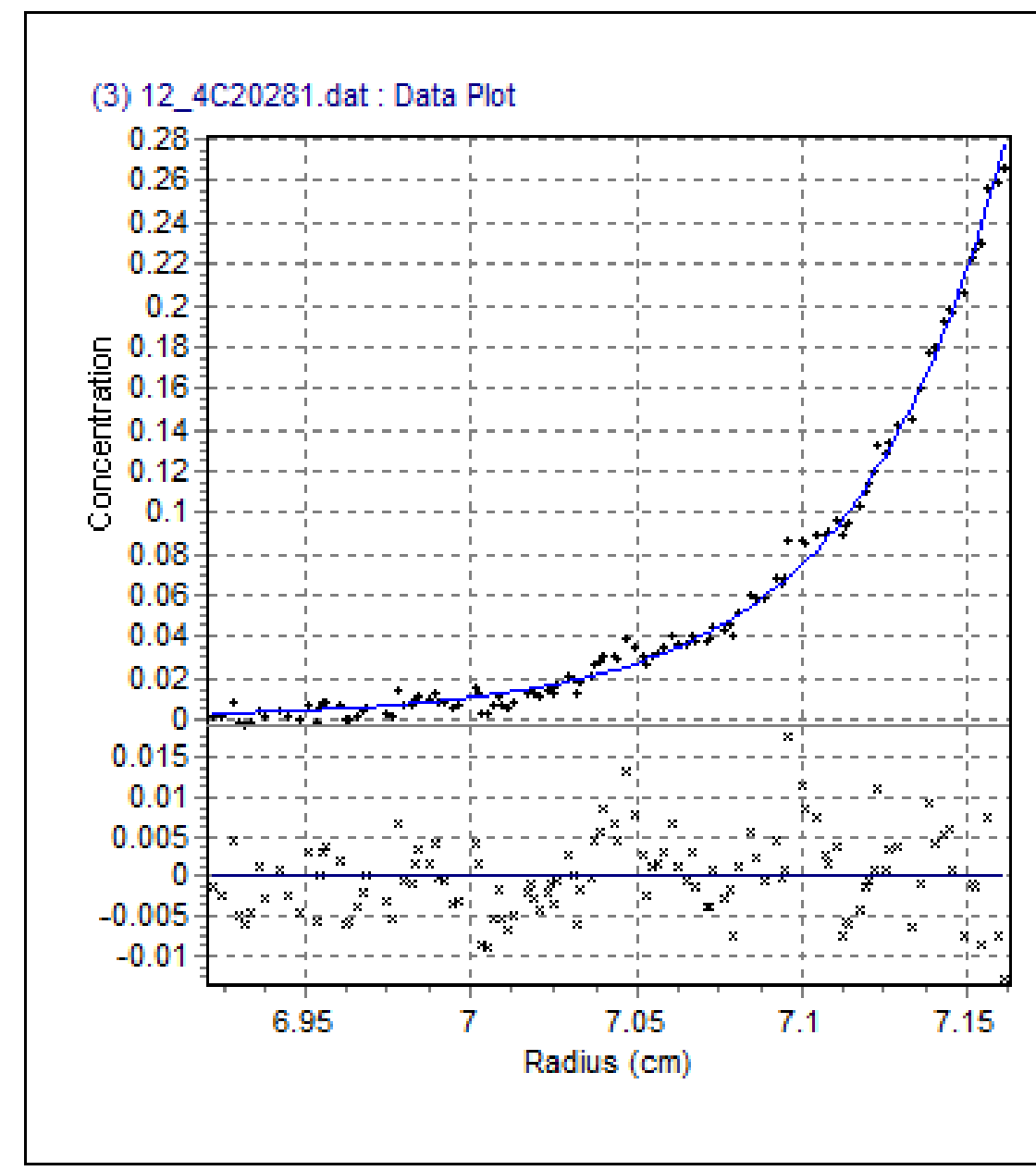

SUPPLEMENTARY FIGURE 2J

Flow cytometry analysis by double staining on $CD33^M$/$CD33^M$, $CD33^m$/$CD33^m$ or $CD33^M$/$CD33^m$ transfected cells using antibodies specific to $CD33^M$ (WM53) and $CD33^m$ (A16121H) respectively. Mean Fluorescence Intensities (MFIs) of cells expressing $CD33^M$ (upper) and $CD33^m$ (lower) were quantified. Error bars represent SEM. Statistical analysis was performed using one-way ANOVA followed by Tukey's test (n = 6, in 3 independent experiments, ***$p<0.001$ *vs* M/m).

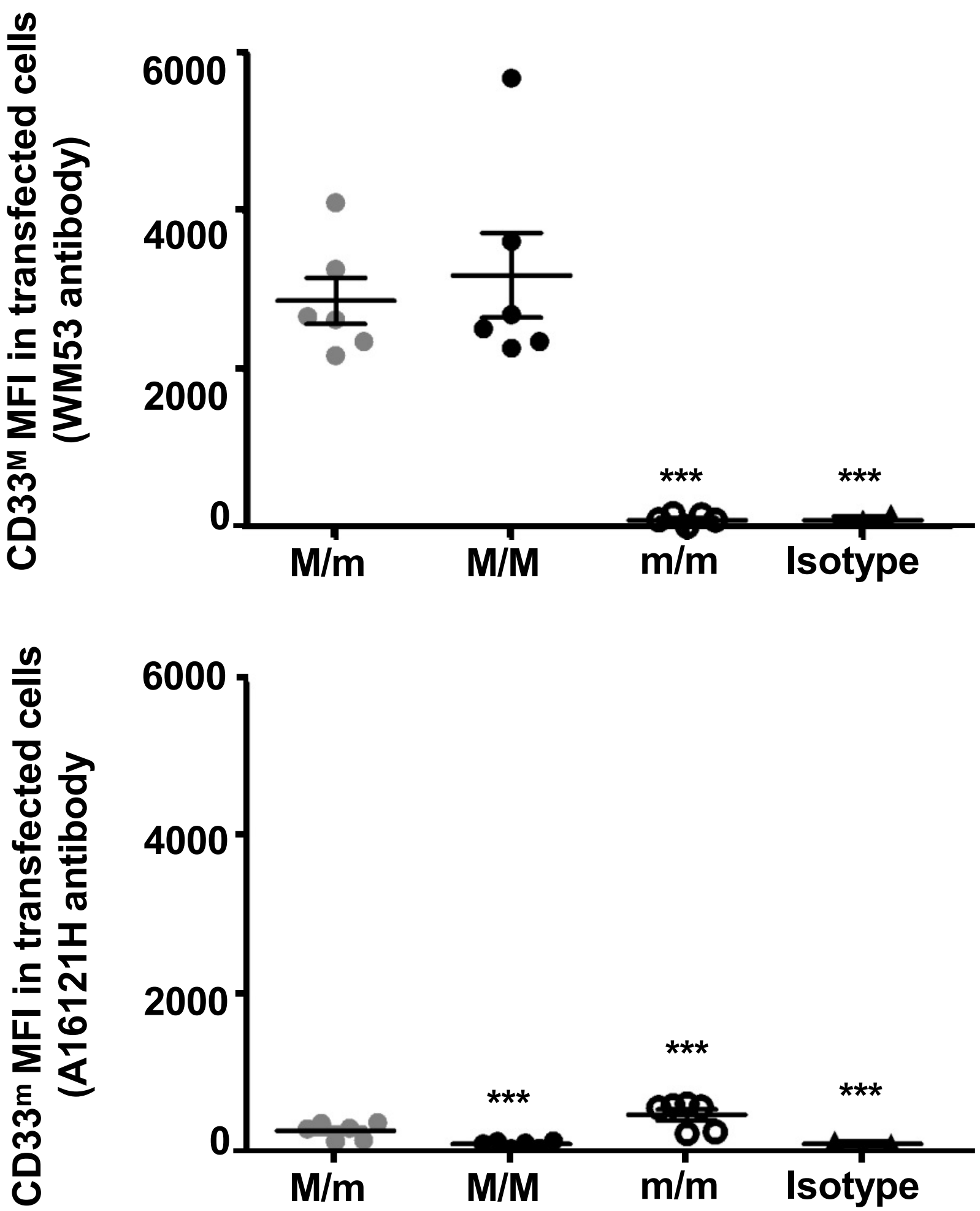

SUPPLEMENTARY FIGURE 2K

Supplementary Data Figure 1K: **Quantification of the surface density and diffusion states of labeled CD33 receptor samples**. **A**, Surface densities prior to smFRET imaging of donor and acceptor labeled samples for smFRET studies. Dots represent the number of acceptor (nAcc) or donor (nDon) particles per area for the indicated number of single cells (*n* cells) collected over 4 independent experiments for the CD33M and CD33m samples, and 3 independent experiments for the CD33M-5X and CD33M donor only samples. Box plot details are described in the legend of Fig. 2f-g. The median density of total labeled (acceptor + donor) receptors ranged from 0.34 – 0.60 molecules/μm². The median density of the donor only labeled CD33M sample was 0.22 molecules/μm² and near zero acceptors detected as expected. **B**, Total fraction of time spent in the immobile, confined, free, and directed diffusion states assigned by DC-MSS. Dots represent individual cell means and the middle and upper/lower lines depict the overall mean (values shown) and standard deviation, respectively, for 32 cells for the CD33M sample and 34 cells for the CD33m sample collected over 4 independent experiments.

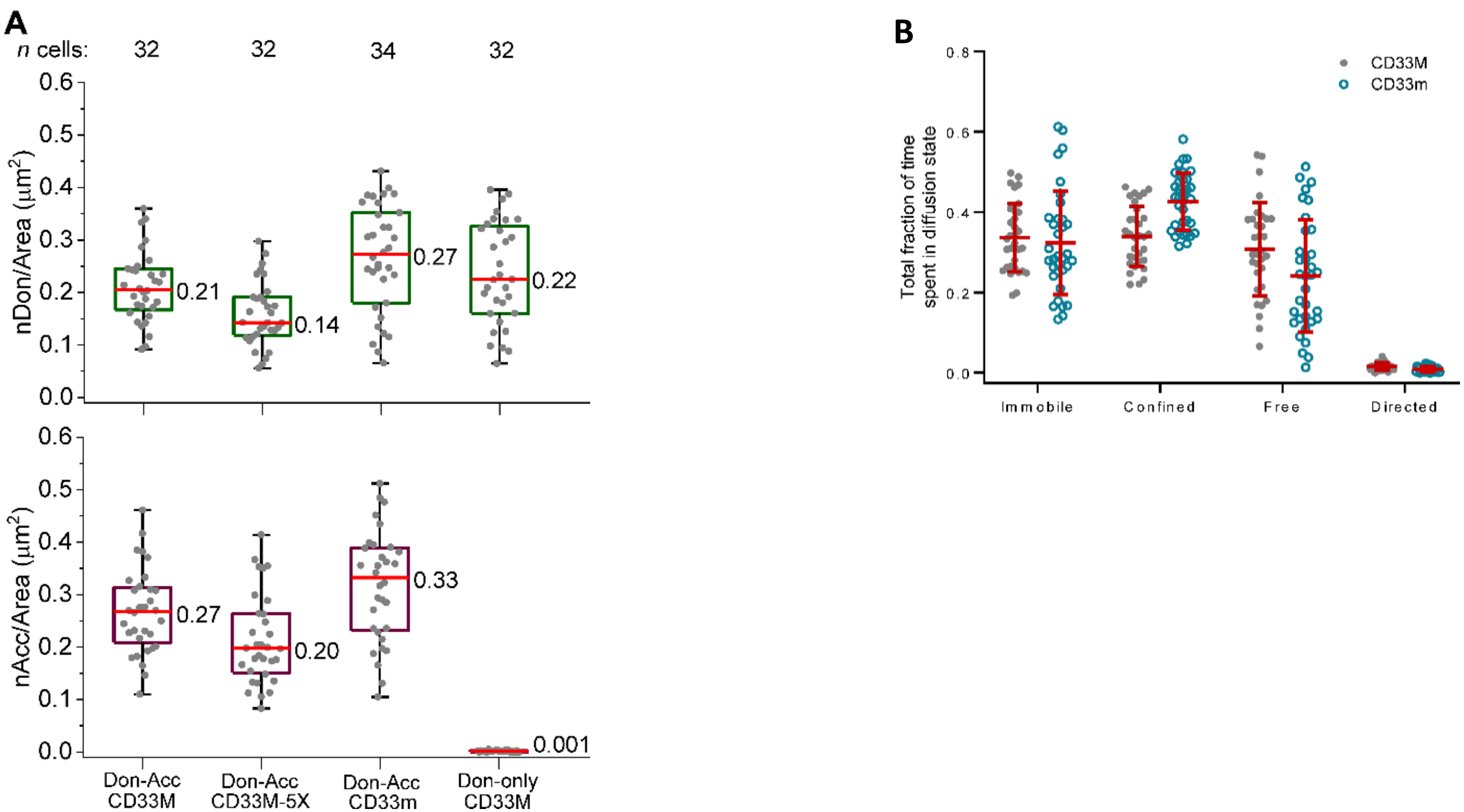

SUPPLEMENTARY FIGURE 2L

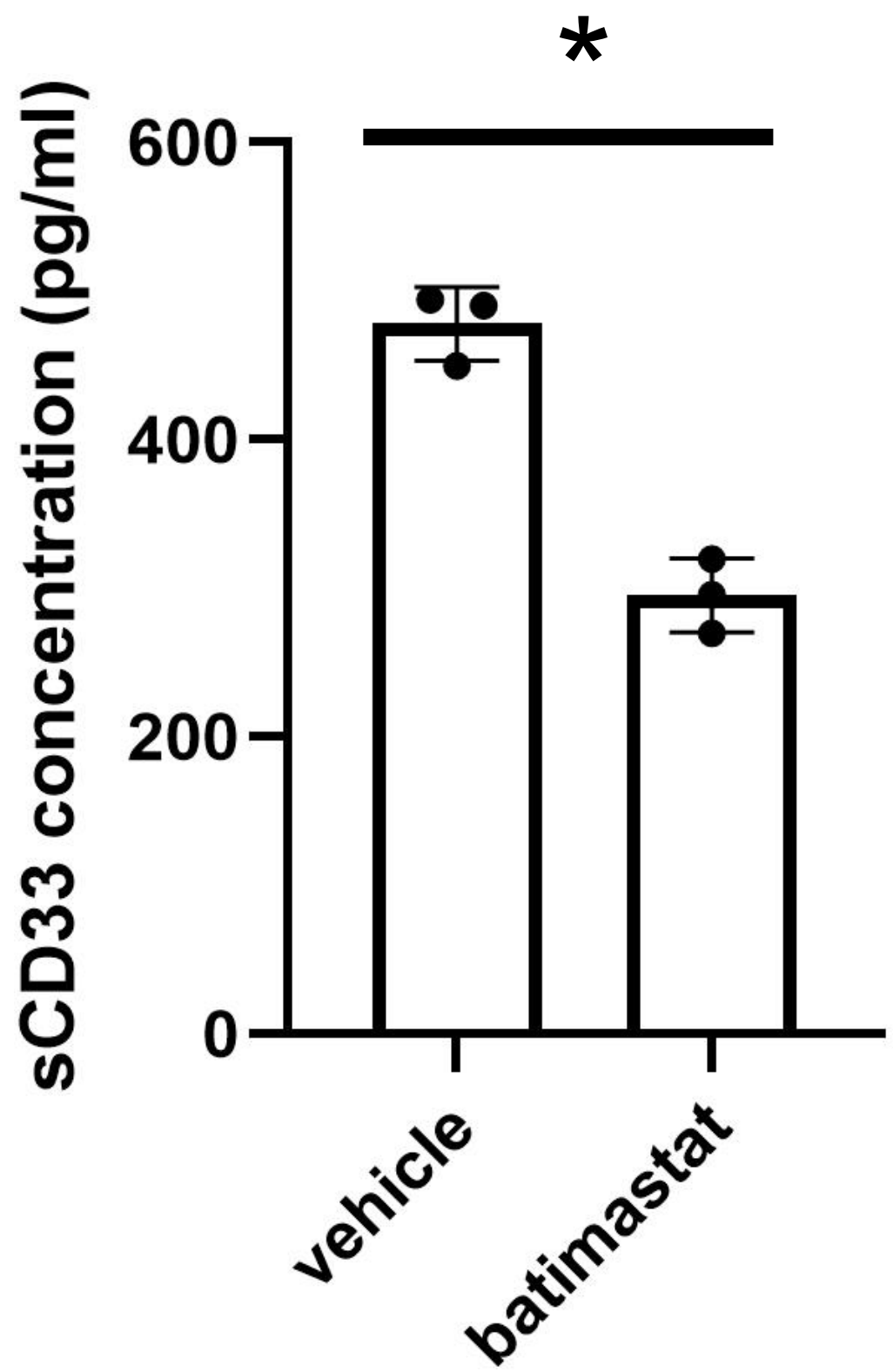

A broad-spectrum sheddase inhibitor, batimastat could reduce soluble CD33 in iMG conditioned medium. Differentiated iMGs were treated with 5 µM batimastat for 24 hours. Conditioned media was collected, centrifuged at 14,000 rpm for 10 minutes at 4°C. sCD33 were measured by ELISA (Abcam, ab283542) following the manufacturer's protocol. Three independent wells of each treatments were collected, and all samples were assayed in duplicates. Statistical analysis was performed using paired t-test, $p=0.0134$.

SUPPLEMENTARY FIGURE 2M

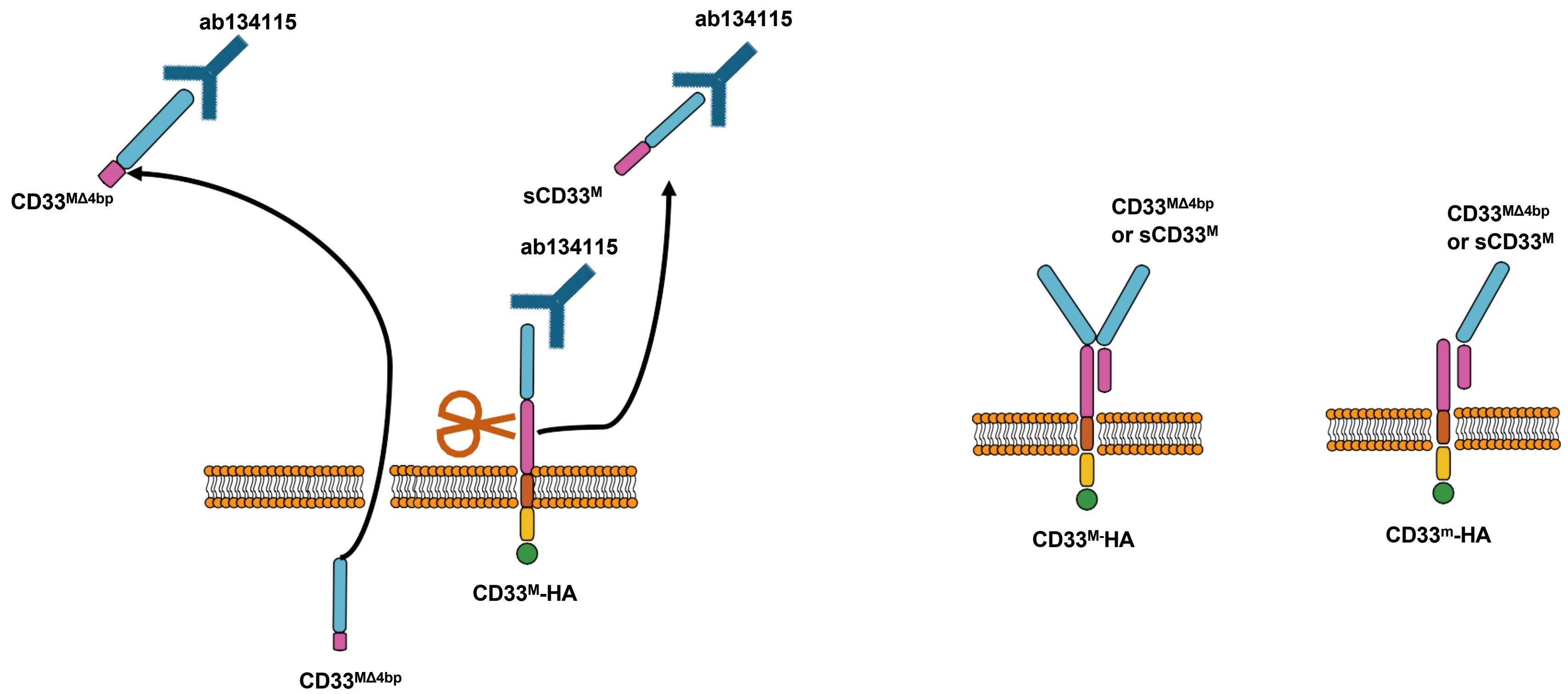

Cartoons depicting: **Left**: the putative secretion of CD33^{MΔ4BP} and putative endoproteolytic shedding of sCD33^{M}, and the tagging and western blot strategy used here. The scissor cartoon depicts the site of a putative endoproteolytic cleavage or splice site that generates the endogenous soluble CD33^{M} ectodomain. **Right**: the putative binding of CD33^{MΔ4BP} (or speculatively sCD33^{M}) to the extracellular domains of CD33^{M} or CD33^{m}. The CD33^{MΔ4BP}:CD33^{M} binding site is not defined., but could potentially recreate a dimeric ligand pocket with CD33^{M} monomers, and a monomeric ligand pocket with CD33^{m}.

SUPPLEMENTARY FIGURE 3A

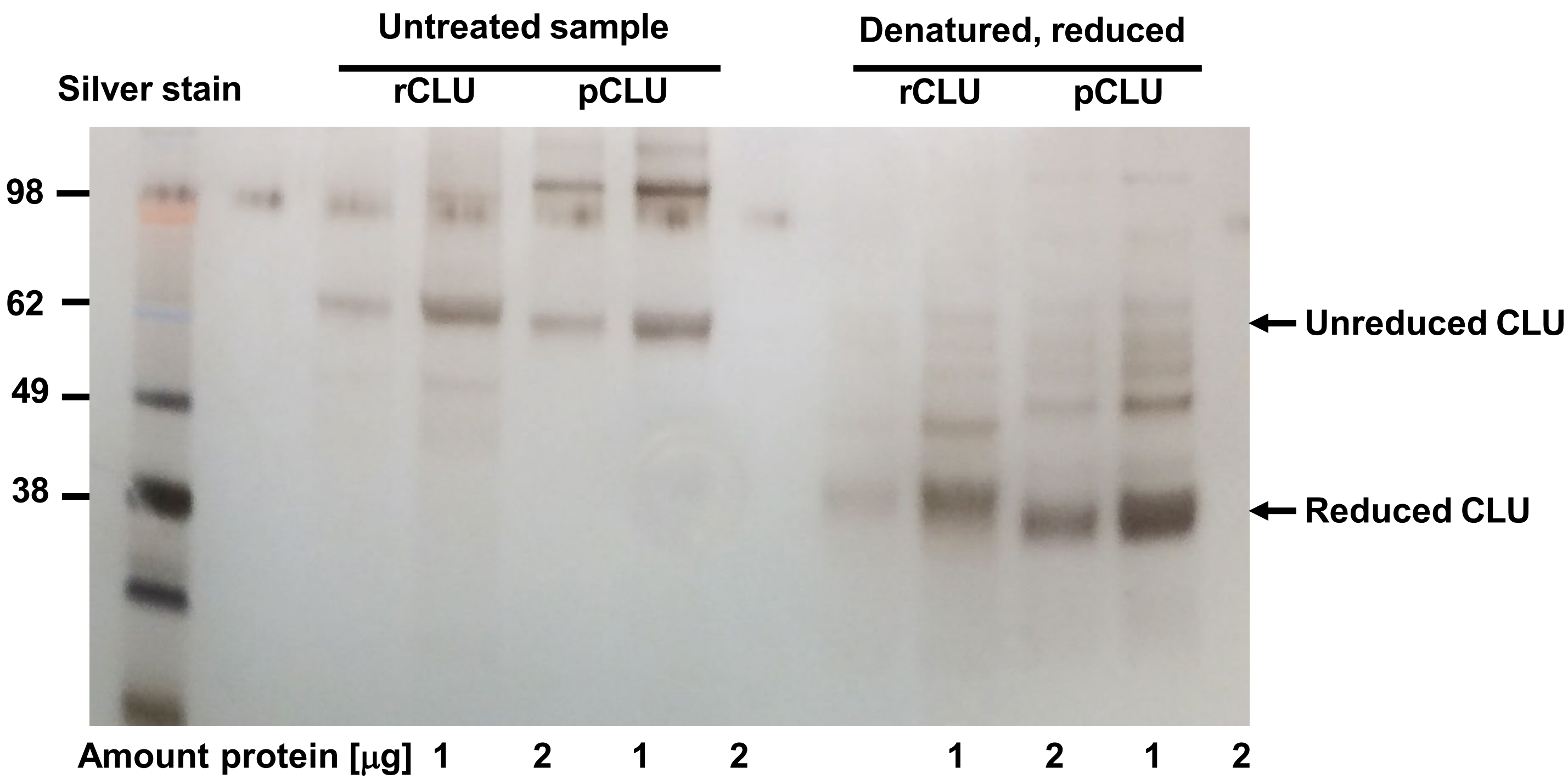

Unprocessed Silver stained gel of recombinant clusterin (rCLU) or purified from human plasma (pCLU) in the native state (left panel) and following denaturation and reduction (right panel).

SUPPLEMENTARY FIGURE 3B

U937

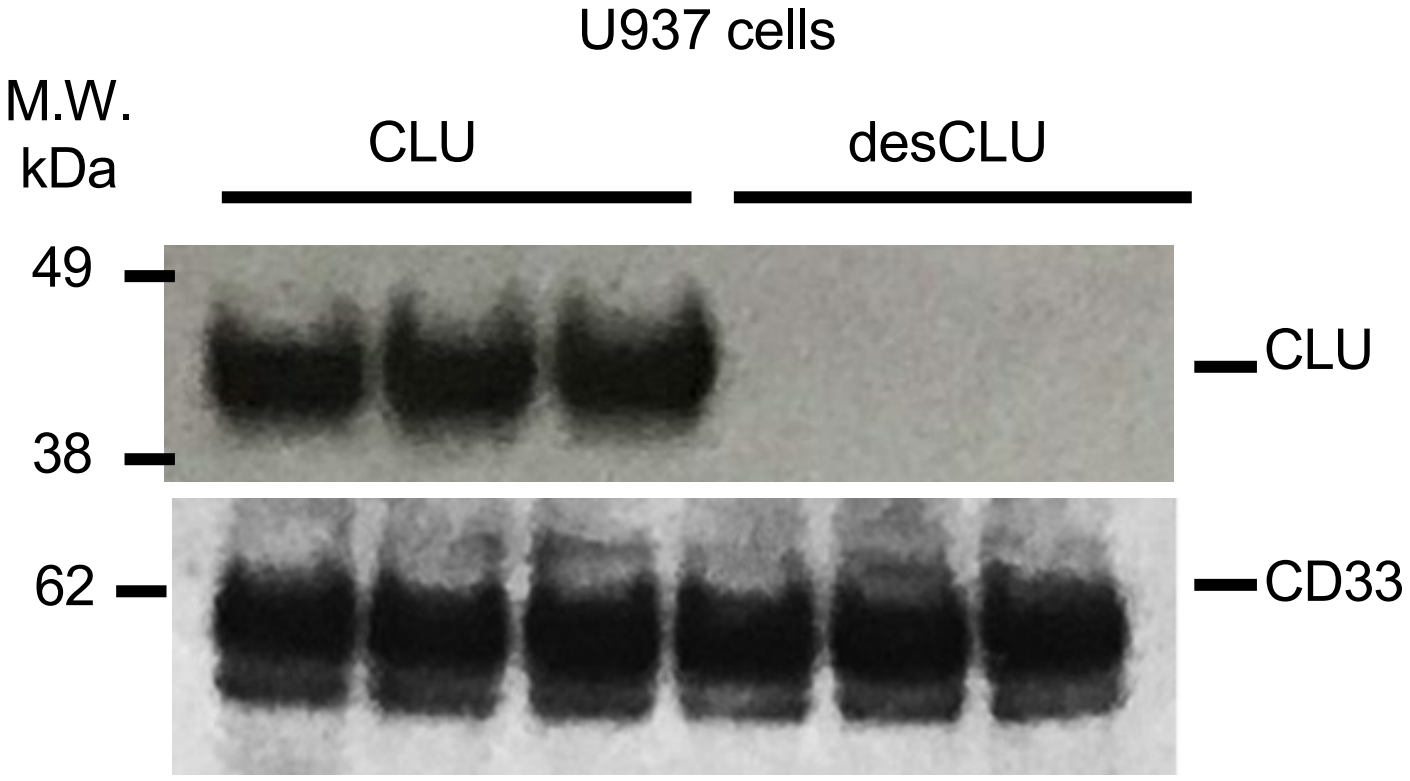

HEK293

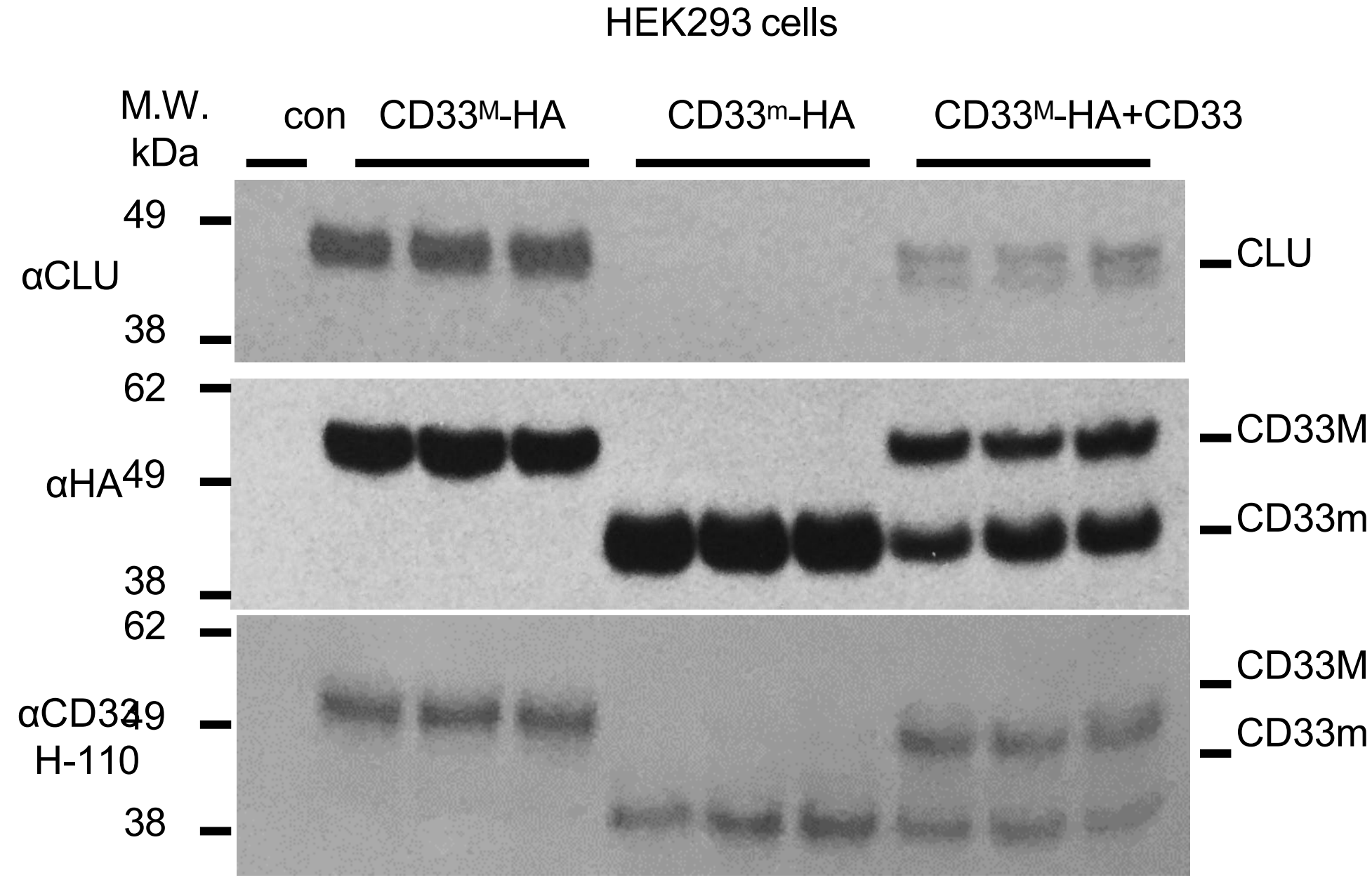

***Left*: endogenous $CD33^M$ co-immunoprecipitates sialylated CLU, but not desialylated CLU in U937 cells.** Representative western blot for Figure 3a. *Top blot*: representative western blots of anti-CD33 co-immunoprecipitation products, blotted with anti-CLU antibodies. *Lower blot*: anti-CD33 co-immunoprecipitation products probed with anti-CD33 antibody, showing equivalent pulldown. This data is quantified in Figure 3a, left panel. Each lane shown represents one independent biological replication of the respective condition.

***Right*: exogenous $CD33^M$ but not $CD33^m$ co-immunoprecipitates with CLU in HEK293 cells.** Representative western blot for Figure 3b. *Top panel:* Representative western blots showing co-precipitation of CLU with $CD33^M$ in HEK293 cells expressing $CD33^M$ only ($CD33^M$/$CD33^M$ dimers), or in HEK293 cells expressing both $CD33^M$ and $CD33^m$ ($CD33^M$/$CD33^M$*;* $CD33^M$*/*$CD33^m$ or $CD33^m$/$CD33^m$ dimers) (CLU does not co-precipitate with CD33 in cells expressing only $CD33^m$). *Top blot*: anti-HA pulldown probed with anti-CLU antibody. *Middle blot*: same IP products probed with anti-HA antibody. *Lower blot*: Protein input before precipitation probed with anti-CD33 antibody. This data is quantified in Figure 3b. In Figure 3B, the data for HEK293 cells expressing both CD33M and CD33m is displayed in the third column is the ratio of CLU immunoreactive band intensities versus just the CD33M band intensity. In the fourth column, the same CLU immunoreactive band intensity is expressed as the ratio of the cumulative band intensities as measured by HA immunoreactivity for both CD33m and CD33M isoforms.Each lane shown represents one independent biological replication of the respective condition.

SUPPLEMENTARY FIGURE 3C

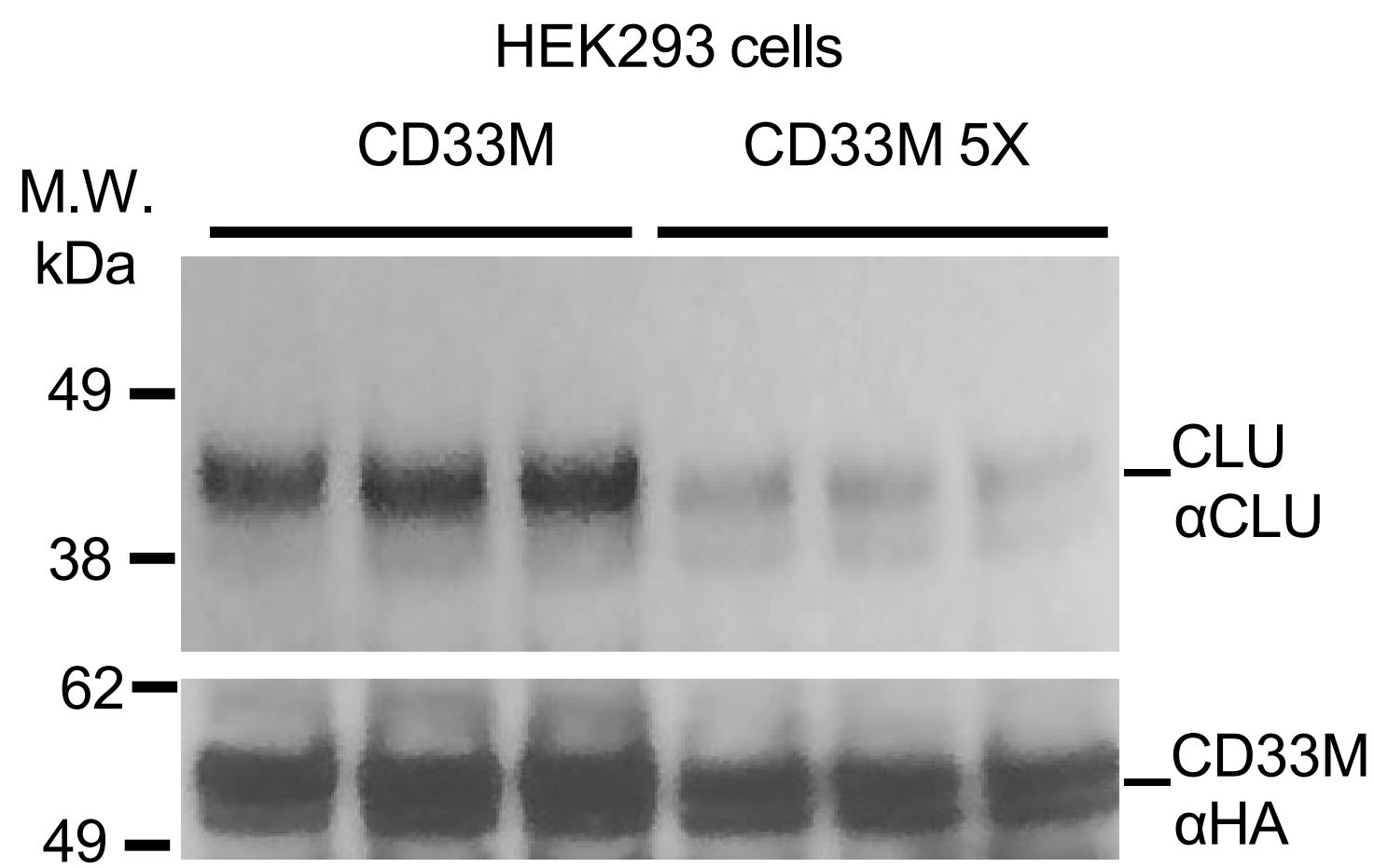

Dimerization of CD33 is required for CLU binding. Co-IP studies on lysates from HEK293 cells expressing similar quantities of $CD33^M$ (*bottom panel*) reveal that much less CLU (*top panel*) is co-precipitated with the $CD33^M$ 5X dimer interface mutant than with wild type $CD33^M$. This is a representative blot for data for multiple biologica; replicates and is quantified in Figure 3c. Each lane shown represents one independent biological replication of the respective condition.

SUPPLEMENTARY FIGURE 3D

CD33^M ectodomain binds 3'-sialyllactose via the canonical R119

BLI response curves representing the association (0-60 s) and dissociation (60-90 s) of CD33^{M} ectodomain (ECD) WT (left panel) or R119A (right panel) in the concentration range of 15.6-250 μM with the 3'-sialyllactose-sp-biotin immobilized on the Super Streptavidin biosensors. Binding curves analysis using a 1:2 bivalent analyte model revealed that the dimer of CD33^{M} ECD WT binds to 3'-syalyllactose with dissociation constants 6.13 ± 0.25 mM (mean ± SEM). CD33^{M} ECD R119A showed no detectable binding in the same concentration range with WT.

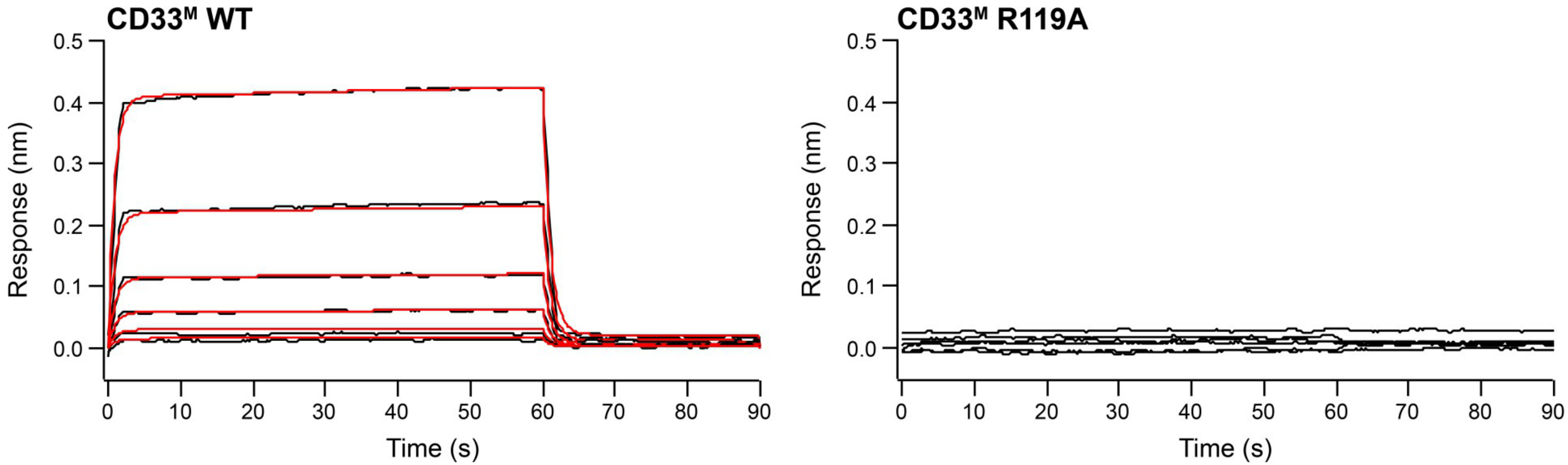

SUPPLEMENTARY FIGURE 3E

Biolayer Interferometry (BLI) studies on the clusterin/Aβ42 mixture binding to CD33^{M} ectodomain (ECD)

A. Binding curves of clusterin/Aβ42 mixture (left panel) or Aβ42 (right panel). Each binding curve is the average of four independent measurements. Clusterin and Aβ42 concentrations are indicated.

B. Steady-state BLI analysis of the clusterin/Aβ42 mixture (red square) or Aβ42 (blue diamond) binding to CD33^{M} ECD. Error bars represent SD.

C. The (Aβ42 -) condition corresponds to CLU alone and is a replot of the data in main figure 3d iv**.** The (Aβ42 +) condition corresponds to CLU:AB42 mixture, calculated by subtracting the corresponding AB-alone from the CLU+AB42 data shown in panel B of this figure. Error bars represent SD. Statistical analysis was performed using two-way ANOVA followed by Bonferroni's multiple comparison test (n = 4, **p*<0.05, ***p*<0.01 *vs* clusterin alone).

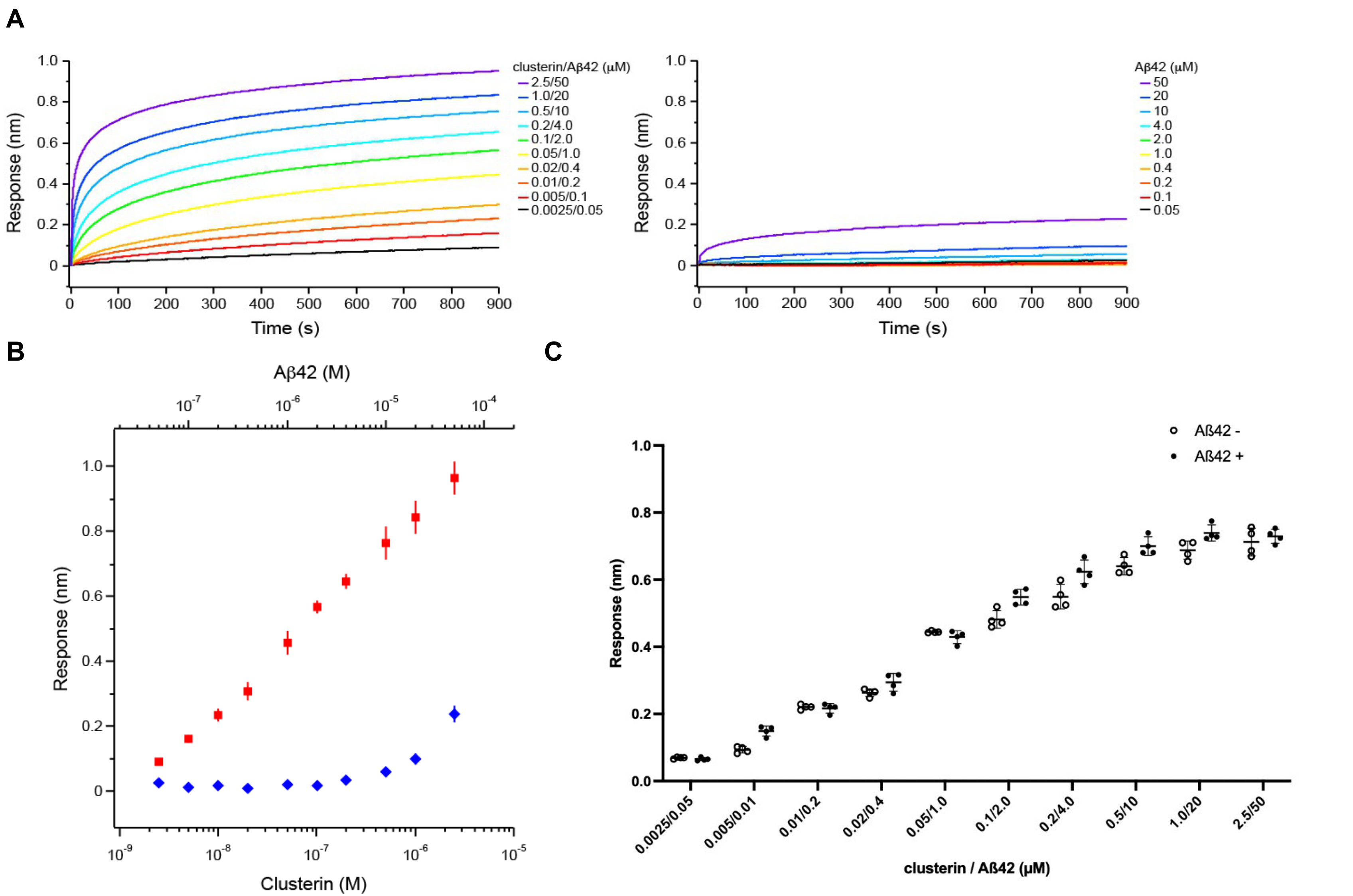

SUPPLEMENTARY FIGURE 3F

Clusterin/Aβ42 mixture bind to CD33[M] ectodomain (ECD) in a clusterin/Aβ42 ratio dependent manner

A. Binding curves of clusterin (black) or clusterin/Aβ42 mixture (red, blue and green), respectively. Each binding curve is the average of four independent experiments. Clusterin and Aβ42 concentrations are indicated.

B. Steady-state binding responses of clusterin or clusterin/Aβ42 mixture. Error bars represent SEM. Statistical analysis was performed using one-way ANOVA followed by Tukey's test (n = 4, ****p*<0.001 *vs* clusterin alone).

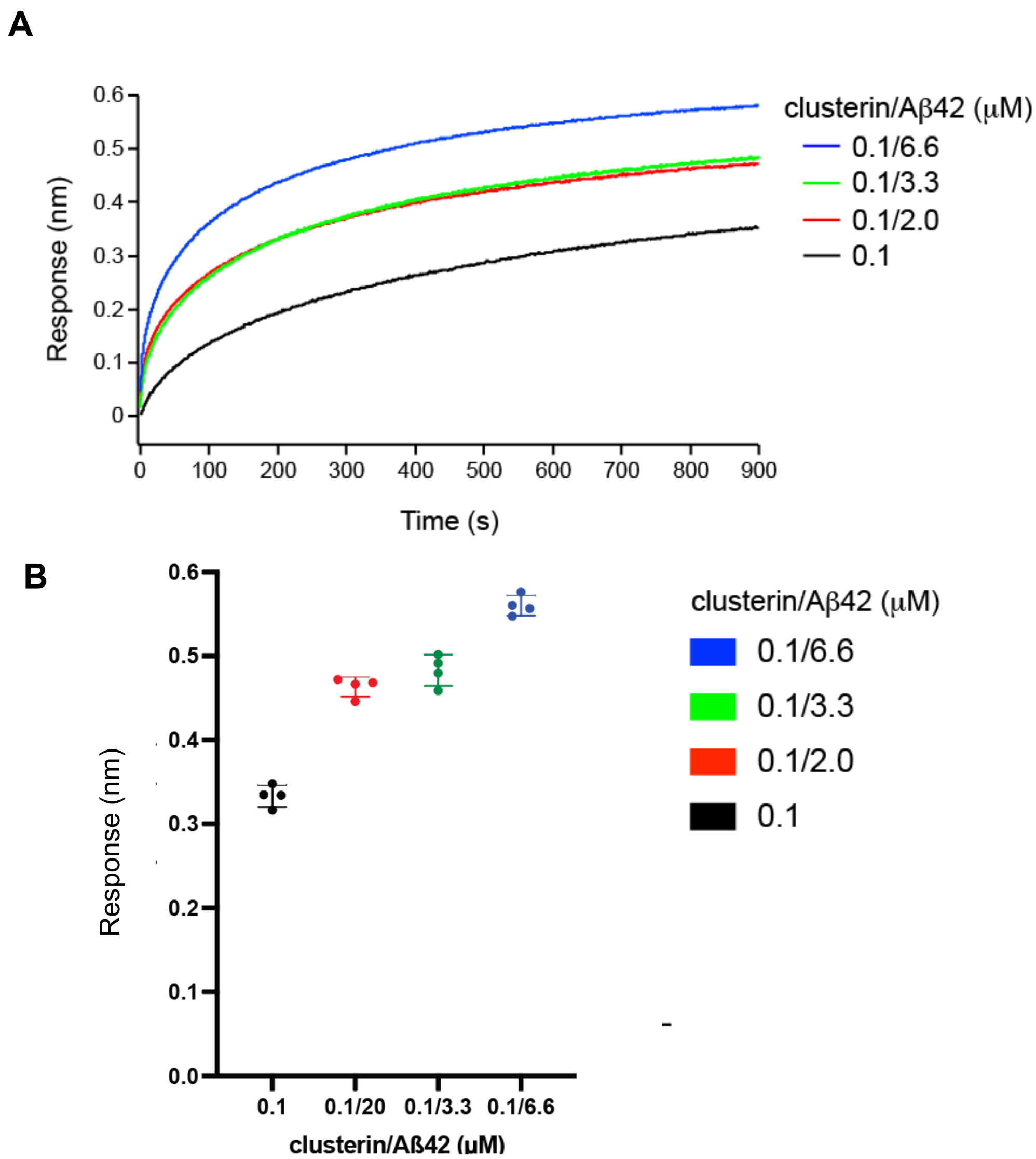

SUPPLEMENTARY FIGURE 3G

Binding analysis by microscale thermophoresis measuring the change in the fluorescence intensity of fluorescently labeled deglycosylated CD33 ECD (initial fluorescence)

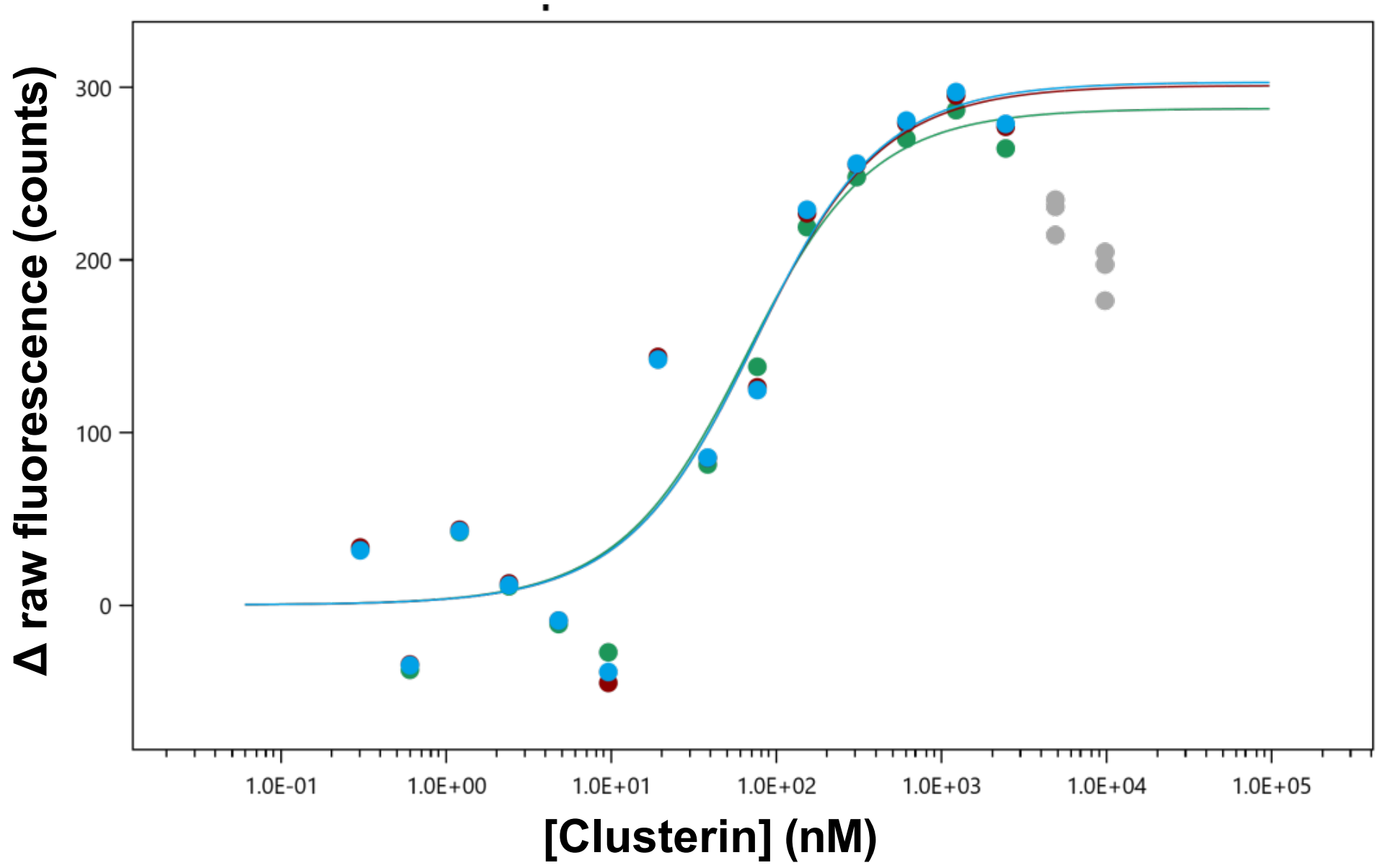

Kd 55.1 nM +/- 2.6 (SEM)

SUPPLEMENTARY FIGURE 3H

Far UV circular dichroism spectra for wild type CD33^M ectodomain (black) and R119A mutant CD33^M ectodomain (red) reveal similar folding states, indicating that the loss of sialic acid binding by the R119A protein is due to an authentic effect on ligand binding, rather than due to simple misfolding of the R119A mutant protein.. Spectra were recorded at 4°C with CD33 ECD at 0.2 mg/ml in 20 mM HEPES, 200 mM NaCl, pH7.5.

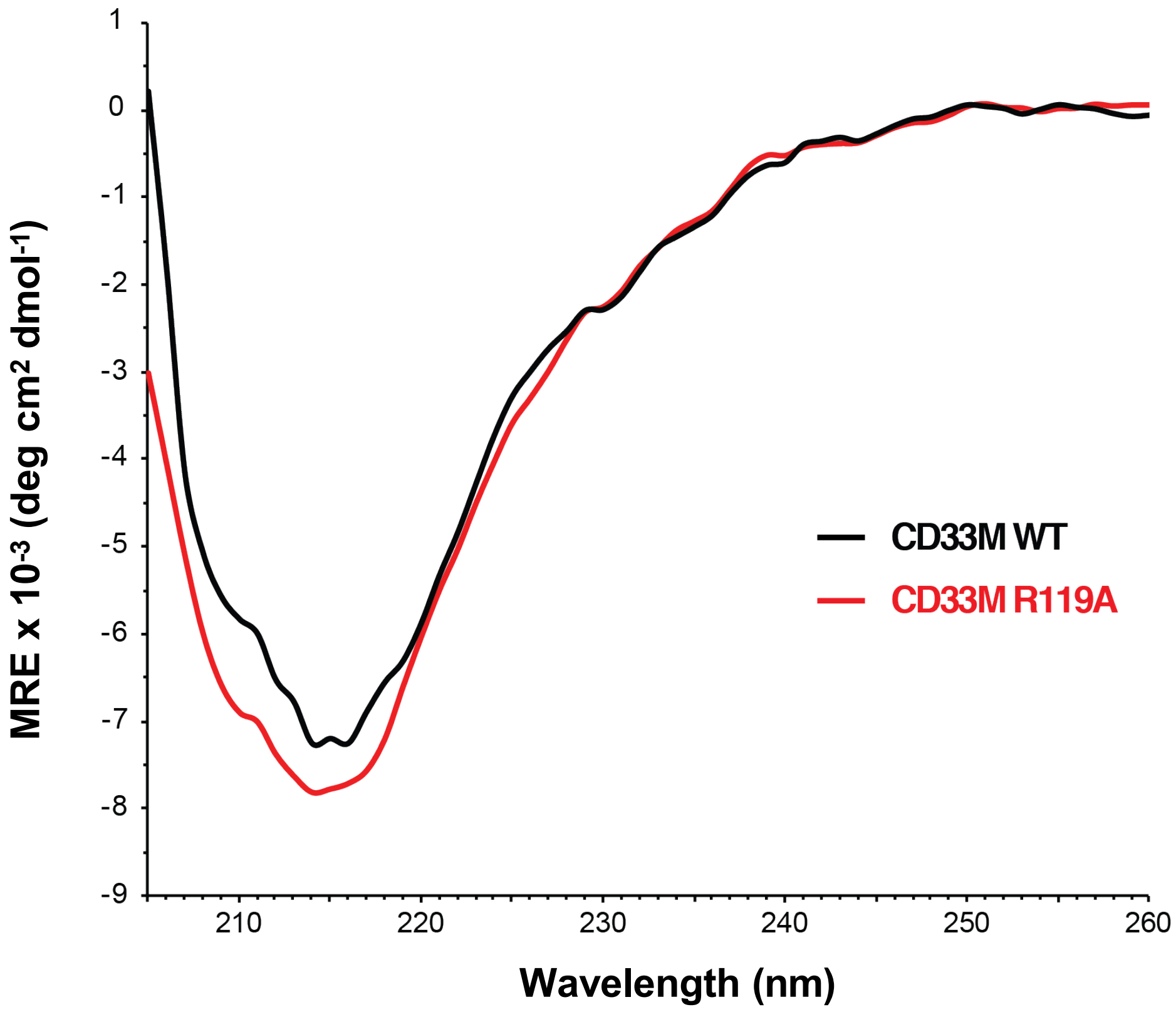

SUPPLEMENTARY FIGURE 4A

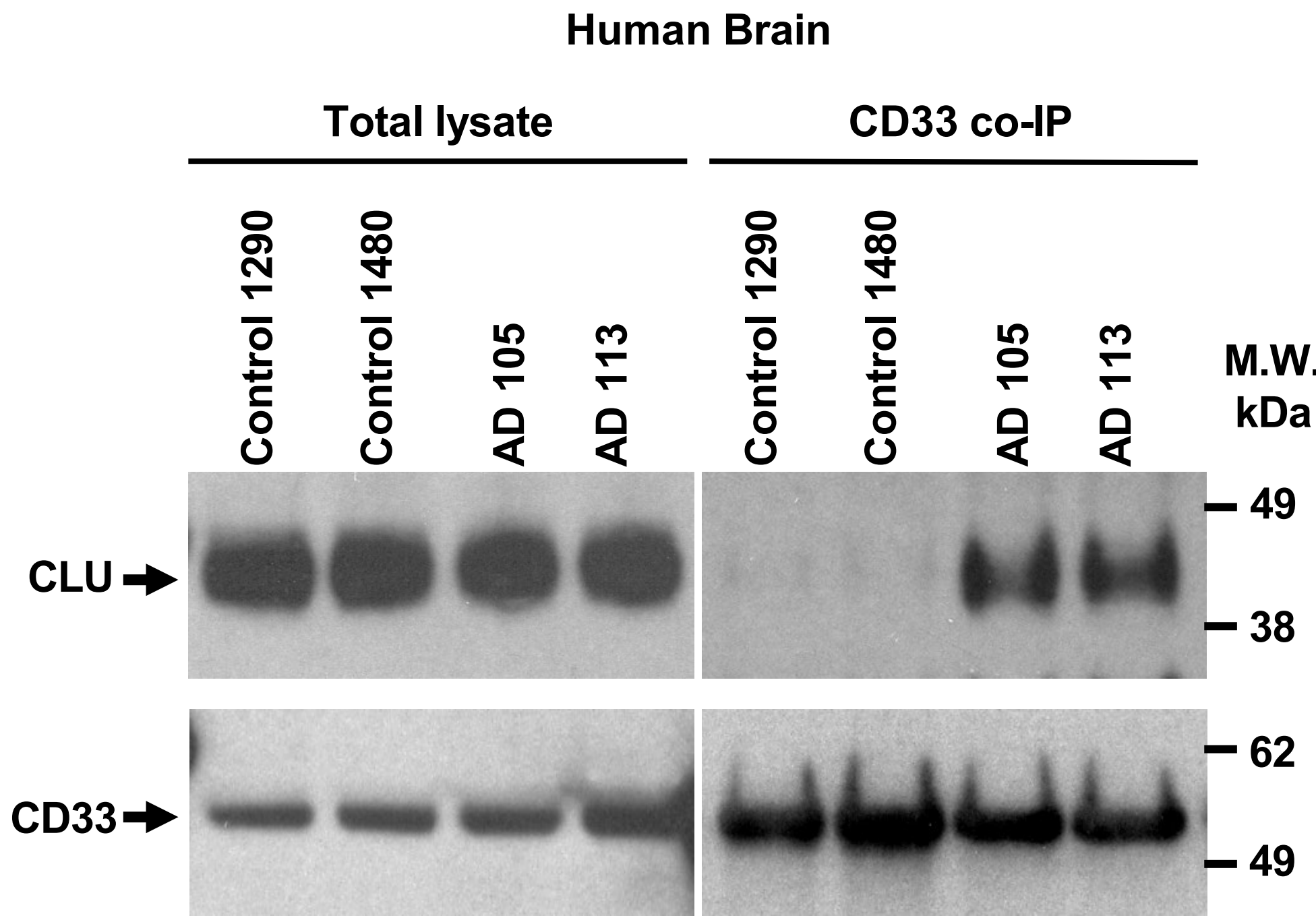

Representative Western blot for Figure 4c. Co-IP studies on lysates from HEK293 cells expressing similar quantities of $CD33^M$ (*bottom panel*) reveal that much less CLU (*top panel*) is co-precipitated with the $CD33^M$ 5X dimer interface mutant than with wild type $CD33^M$. This data is quantified in Extended Data Figure 3D. *Right panel;* quantification. *** $p<0.00001$, error bars = mean ± SEM $CD33^M$: 0.425±0.0073; $CD33^M$ 5X: 0.012±0.006; n = 5 replications. Each lane represents one independent biological replication of the respective condition.

SUPPLEMENTARY FIGURE 4B

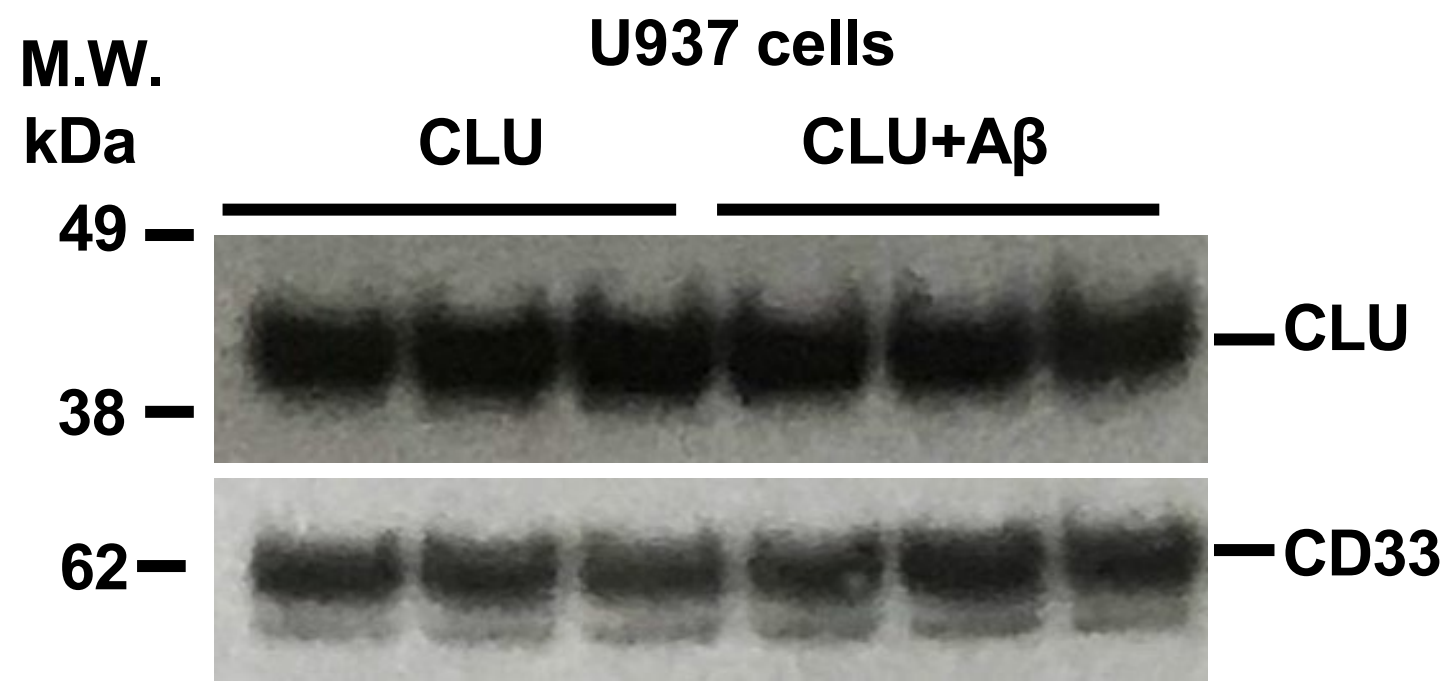

Representative Western blots for Figure 4e **showing that native human CLU alone, or complexed with Aβ oligomers also binds endogenous human CD33[M] in U937 cells.** *Upper blot* is a representative western blots for anti-CD33 co-IP of CLU alone or CLU plus Aβ oligomers. *Lower blot* is a representative wester blot of CD33 co-IP products probed with anti-CD33 antibody to show similar CD33 IP recovery. Each lane represents one independent biological replication of the respective condition.

SUPPLEMENTARY FIGURE 5A

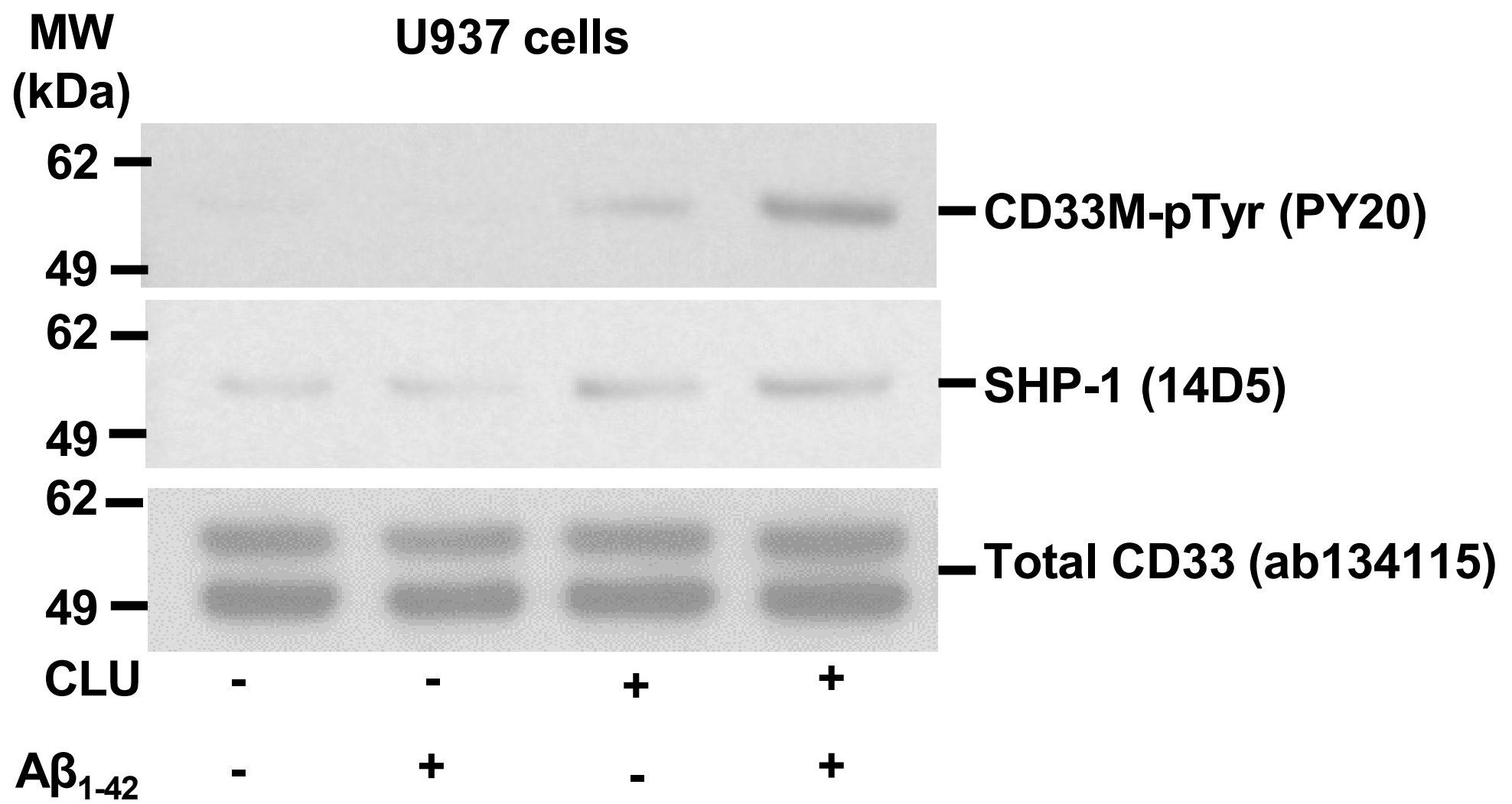

Representative blot of n ≥ 3 independent biological replications for the graph in Figure 5a, showing that human plasma CLU alone, and especially human CLU + Aβ oligomers induce ITIM tyrosine phosphorylation of endogenous CD33 *(top panel)* and recruitment of endogenous SHP-1 *(middle panel)* in human monocytic U937 cells..

SUPPLEMENTARY FIGURE 5B

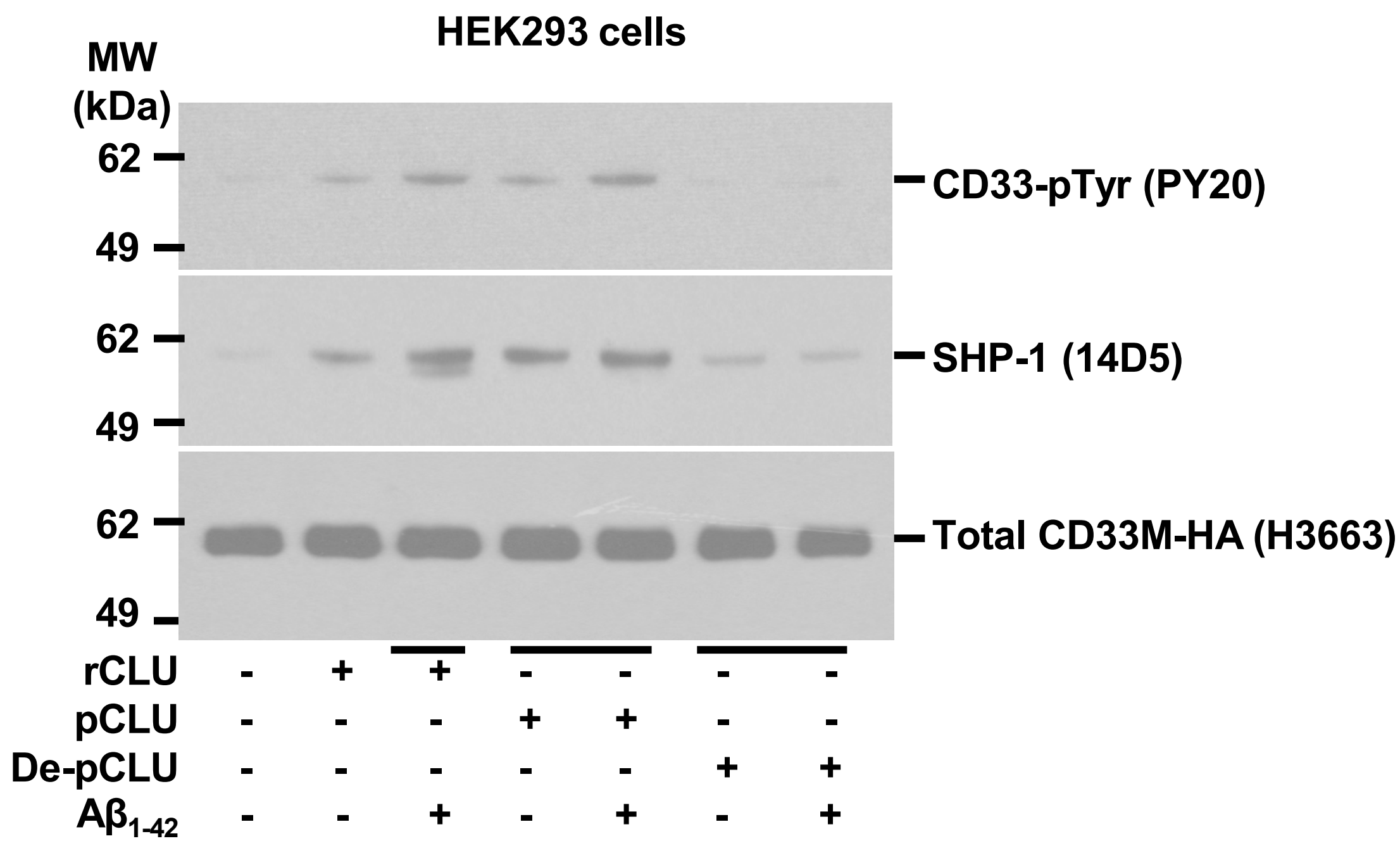

Representative western blot of n = 3-4 independent biological replications with different cell lines for Figure 5b. Recombinant human clusterin (rCLU) and native human plasma clusterin (pCLU), but not desialylated clusterin equivalently activate CD33M ITIM phosphorylation and SHP-1 recruitment in HEK293 cells. CD33M tyrosine phosphorylation (top panel) and SHP-1 recruitment (middle panel) were measured by Western blots band intensity ratios for pY20-phosphotyrosine immunoreactive CD33M versus total CD33M (bottom panel) by co-IP western blots..

SUPPLEMENTARY FIGURE 5C,D

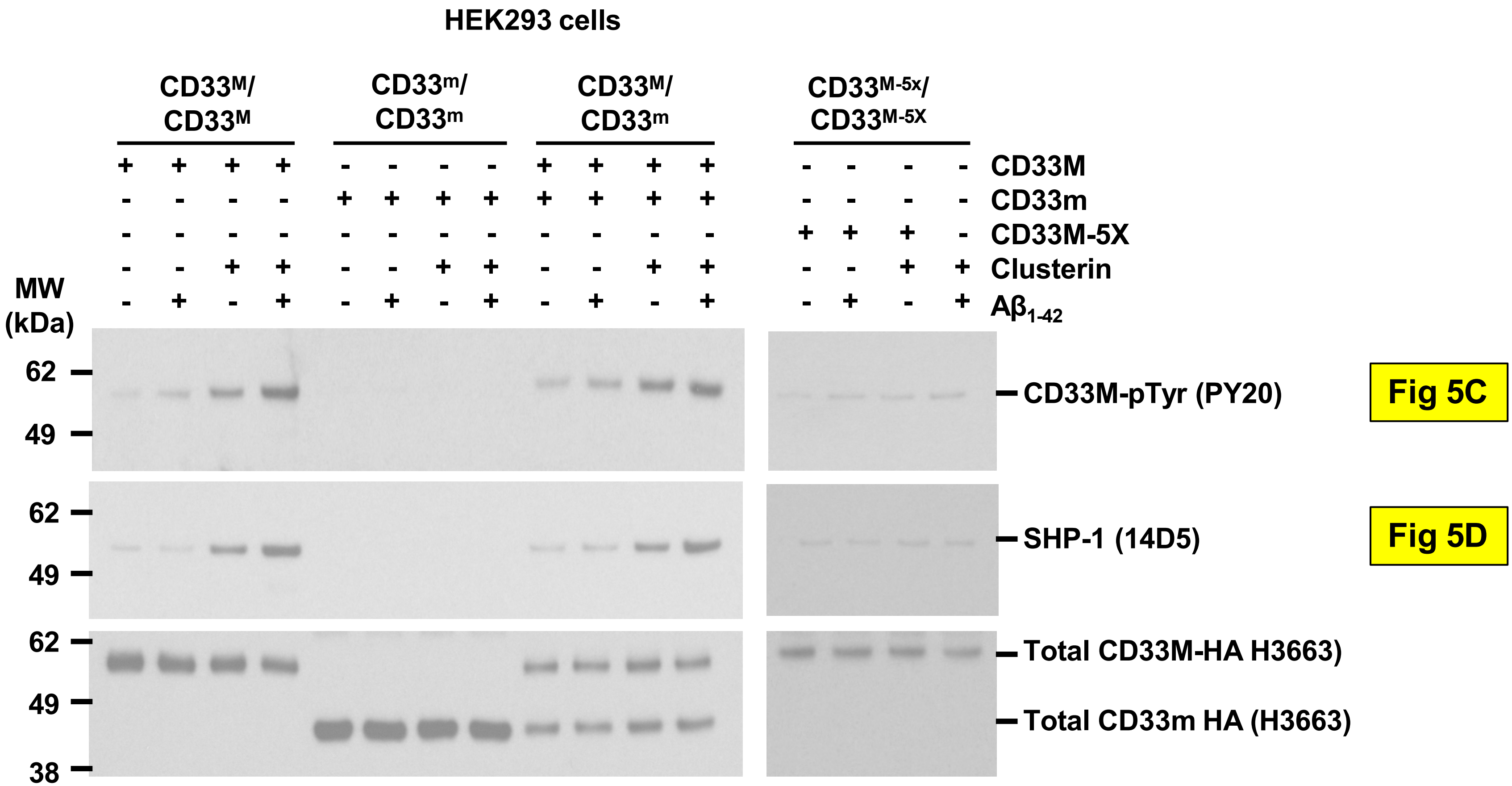

Representative western blot from n = 3 independent biological replications for Figure 5c,d. CLU + Aβ induces greater CD33 ITIM phosphorylation *(top panel)* and SHP-1 recruitment *(bottom panel)* than CLU alone, but only in CD33M expressing cells. Quantification of western blots of anti-CD33 immunoprecipitates probed with pY20 anti-phosphotyrosine, SHP-1 or anti-HA antibodies reveals that treatment with CLU alone, and especially treatment with CLU + Aβ oligomers, increases CD33 tyrosine phosphorylation and SHP-1 recruitment in cells expressing either CD33M only or CD33M plus CD33m. No CD33 phosphorylation or SHP-1 recruitment was observed in cells expressing only CD33m, and very little in CD33M 5X cells. n = 3 replications.

SUPPLEMENTARY FIGURE 5E

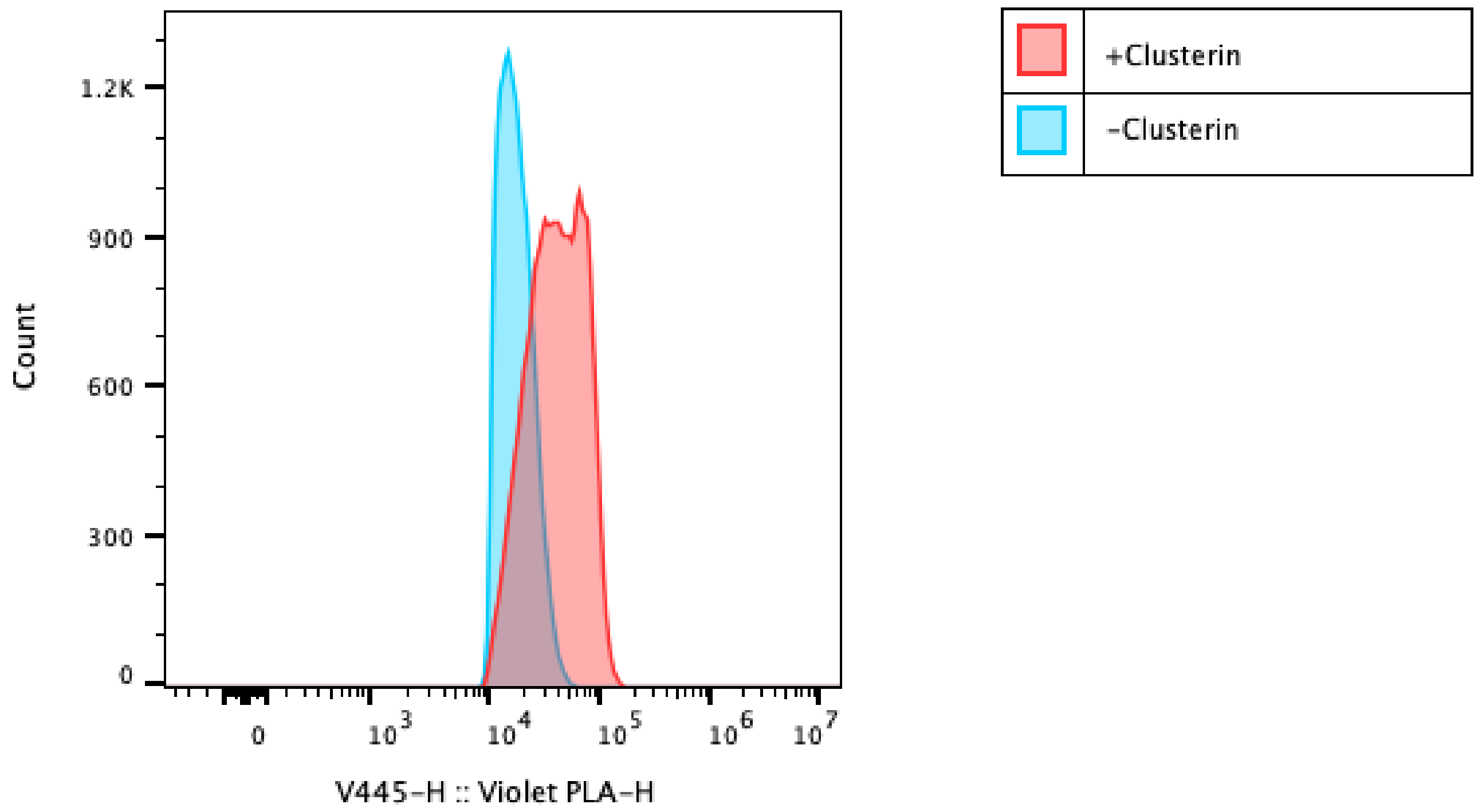

Clusterin significantly increases SHP1 recruitment to CD33 from Figure 5e. Example histogram showing the difference in CD33-SHP1 Flow PLA signal (violet 445) of human monocytes treated with CLU (red) and without CLU (blue) for 15 minutes

SUPPLEMENTARY FIGURE 5F

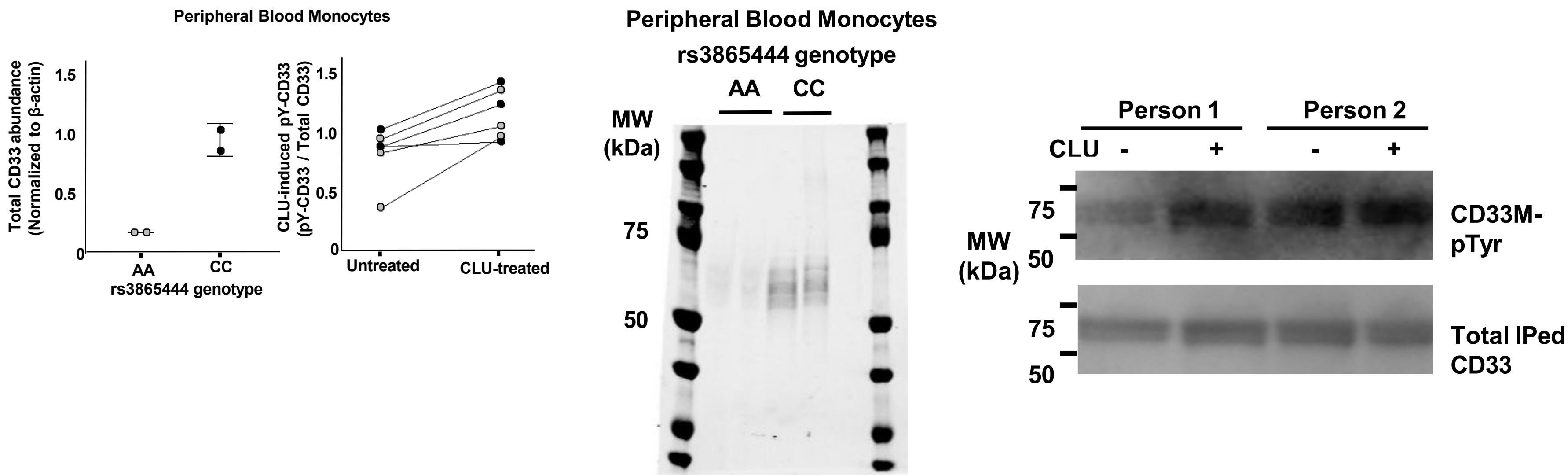

Treatment of Human monocytes with 0.06 µM CLU for 15 min at 37°C increases tyrosine phosphorylation of the ITIM domain on endogenous CD33M. Quantitative western blots of lysates for human blood monocytes reveal that monocytes from homozygous carriers of the rs3865444^{AA} reference /non-risk allele (gray dots) express less total CD33 and less CD33M than do homozygous carriers of the rs3865444^{CC} risk allele (black dots) (*Left panels*). Treatment of human monocytes with 0.06 µM CLU for 15 min at 37°C increases tyrosine phosphorylation of the ITIM domain on endogenous CD33M both in individuals homozygous for the rs3865444^{CC} risk allele (black dots) and in individuals homozygous for the rs3865444^{AA} reference /non-risk allele (gray dots) paired t-test, p=0.007 (2nd *left panel).* NB: As demonstrated in the left panel, carriers of the rs3865444^{CC} risk allele express much more CD33M than do carriers of the rs3865444^{AA} reference allele. However, when equal quantities of CD33M protein are loaded onto these blots to facilitate comparison of basal and CLU-induced CD33 ITIM phosphorylation, the *specific activity* of CD33M signaling is the same.

Representative Western blots for Supplemental Figure 5F. Treatment of Human monocytes with 0.06 µM CLU for 15 min at 37°C increases tyrosine phosphorylation of the ITIM domain on endogenous CD33M. *Middle panel*: Quantitative western blots of lysates for human blood monocytes reveal that monocytes from homozygous carriers of the rs3865444^{AA} reference express less total CD33 and less CD33M than do homozygous carriers of the rs3865444^{CC} risk allele. *Right panel*: Representative western blots showing that treatment of human monocytes with 0.06 µM CLU for 15 min at 37°C increases tyrosine phosphorylation of the ITIM domain on endogenous CD33M both in individuals homozygous for the rs3865444^{CC} risk allele (person 2) and in individuals homozygous for the rs3865444^{AA} reference /nonrisk allele (person 1). Representative western blots are available in Extended Data Figure 5C-G . NB: As demonstrated in the left panel, carriers of the rs3865444^{CC} risk allele express much more CD33M than do carriers of the rs3865444^{AA} reference allele. However, when equal quantities of CD33M protein are loaded (bottom right panel) onto these blots to facilitate comparison of basal and CLU-induced CD33 ITIM phosphorylation, the ***specific activity*** of CD33M signaling is the same.

SUPPLEMENTARY FIGURE 5G

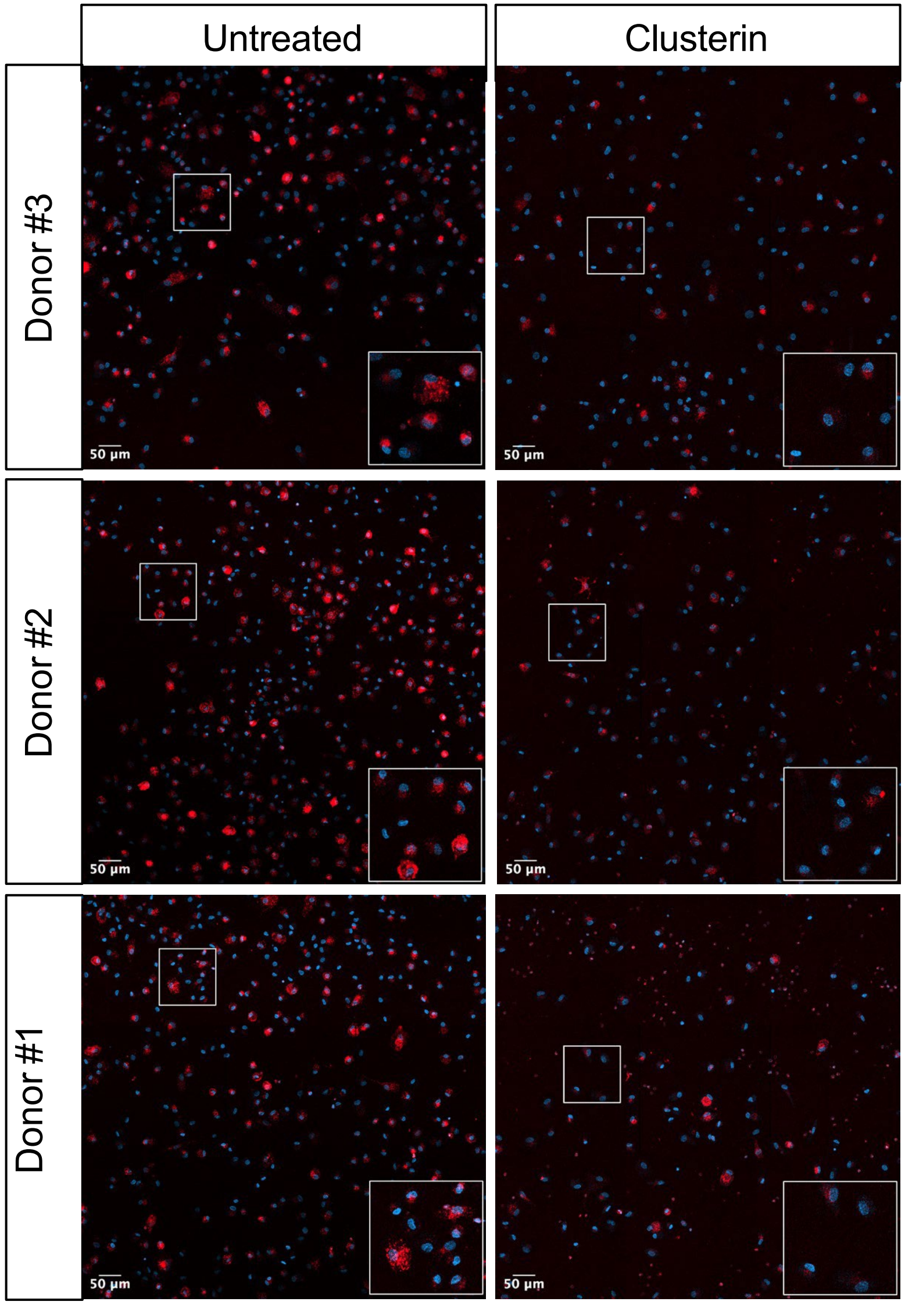

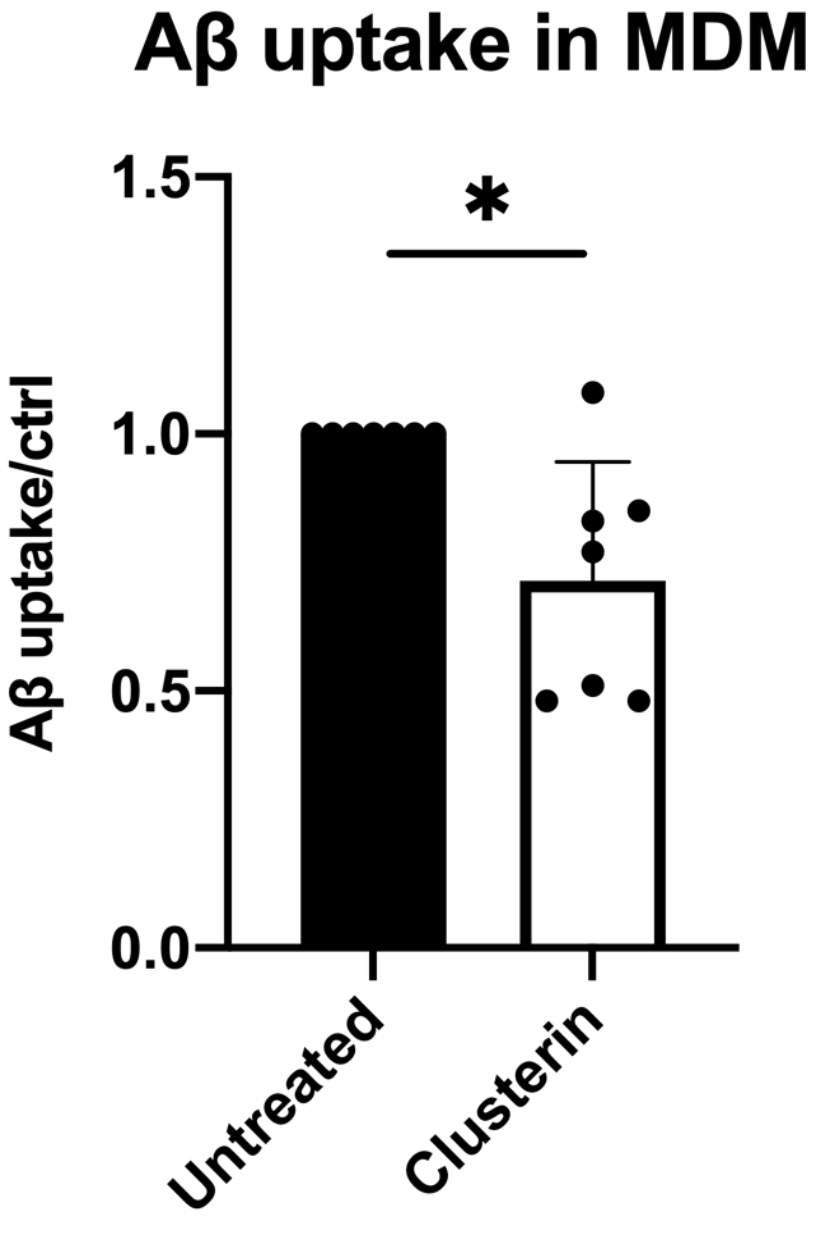

Clusterin treatment reduces Aβ uptake in MDMi. MDMi from 7 individuals were treated with 60nM clusterin for 15 minutes, then incubated with Aβ-647 and imaged for uptake. Representative images from 3 different donors are shown on the left. The bottom right corner of each image is a zoomed-in inlet of the white outlined region. Blue = DAPI. Red = Aβ. Scale bar is 50 µm. Quantification is shown in the bar graph on the right. *$p<0.05$. Wilcoxon test.

SUPPLEMENTARY FIGURE 6A

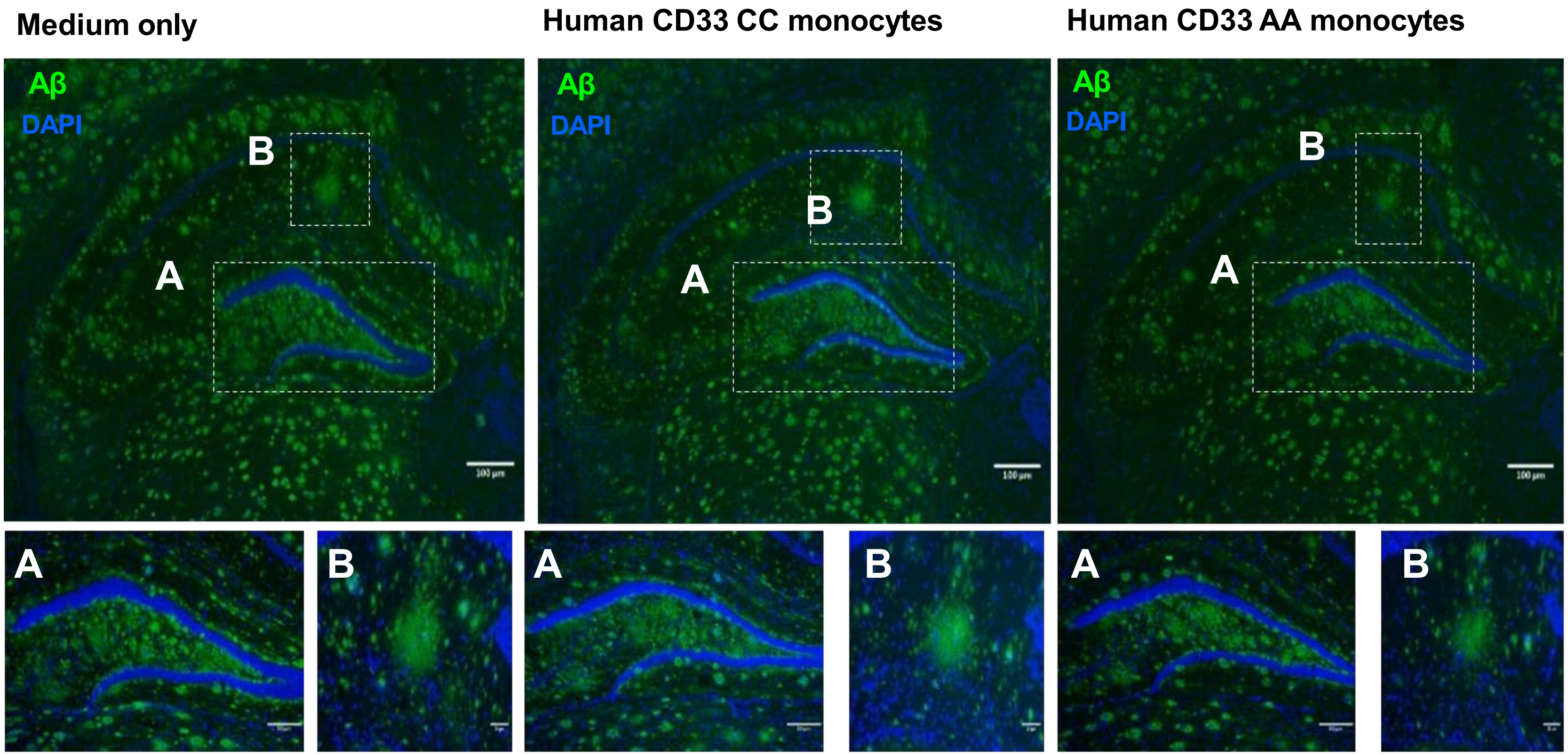

Human monocytes from CD33 "CC" risk allele carriers have reduced clearance of human amyloid plaques in *ex vivo* brain slices compared to monocytes from CD33 "AA" protective allele carriers. **Representative figures quantified in Figure 6a** of clearance of hippocampal Aβ plaques by human monocytes following incubation on serial sections of unfixed brain from 5XFAD mice. Immunofluorescent staining of hippocampal Aβ plaques (green) following 48h incubation with medium only with no cells (*left*) or with CC (risk allele) genotype monocytes (*middle*) or with AA (protective allele) monocytes (*right*). N = 10 independent replications). DAPI blue = nuclei.

SUPPLEMENTARY FIGURE 6B

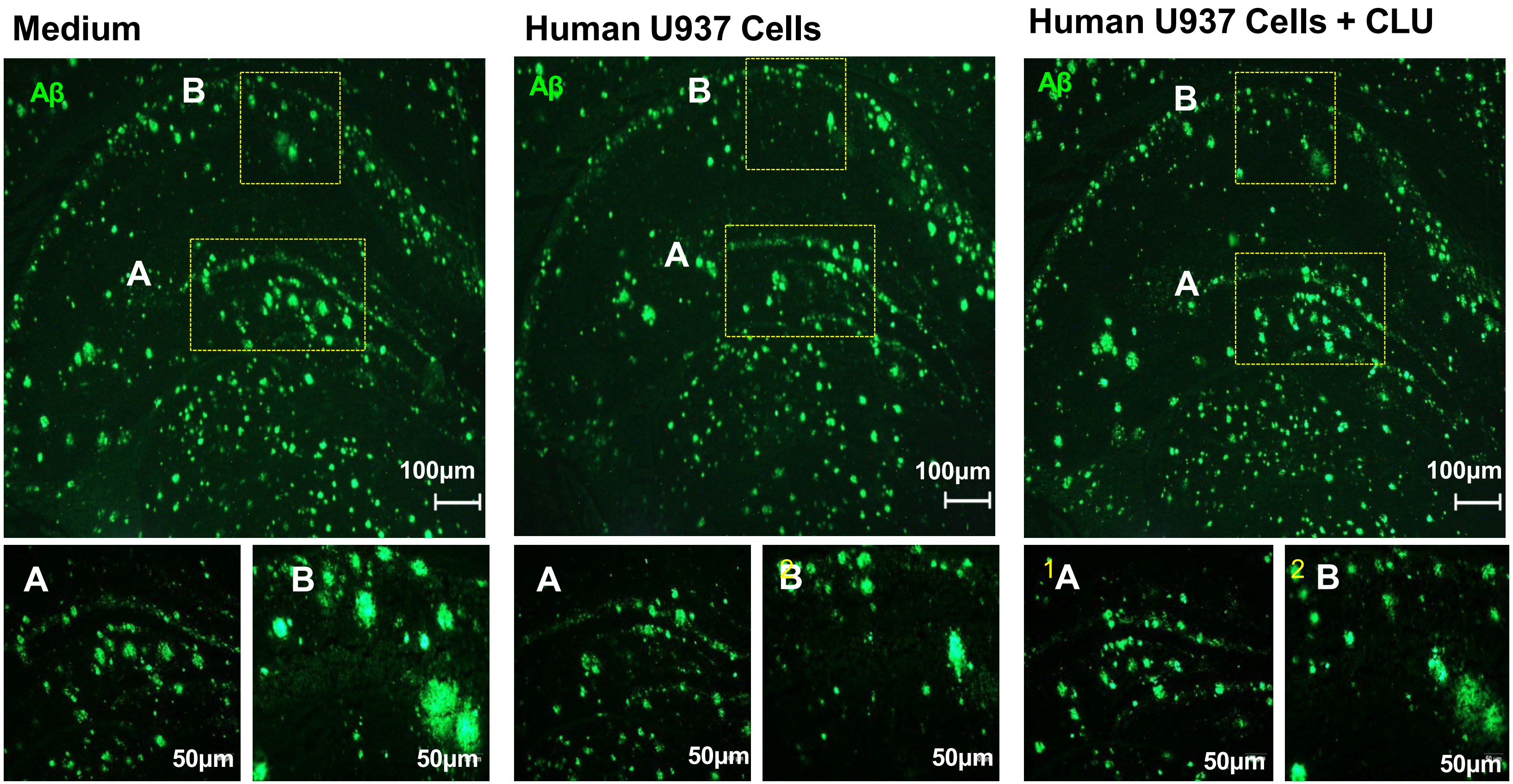

Human U937 monocytic cells clear amyloid plaques from *ex vivo* brain slices, and this is impaired by CLU. Representative figures quantified in Figure 6b of clearance of hippocampal Aβ plaques (green) by U937 cells following incubation on three serial sections of unfixed brain section of 5XFAD mice in the presence/ absence of CLU. Immunofluorescent staining of hippocampal Aβ plaques following 48h incubation with only medium (upper left figure) or with U937 cells alone (upper middle figure) or with U937 cells + CLU (upper right figure). Yellow boxes highlight two regions (A: 10X and B: 20X) that are shown at higher magnification in the corresponding images below the main figure). Scale bars: 100 μm in I and A; and 50 μm in B.

SUPPLEMENTARY FIGURE 6C

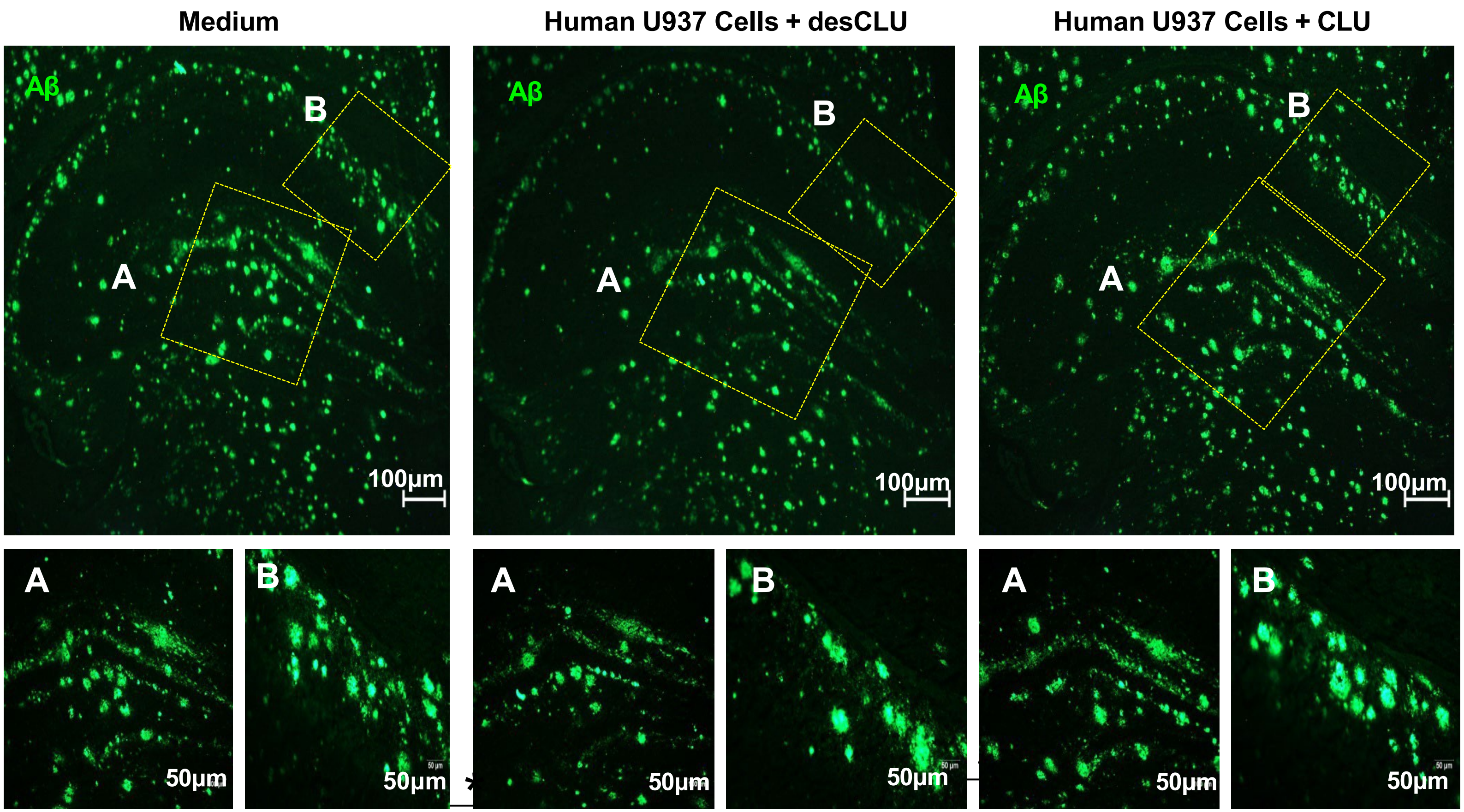

Compared to CLU, desCLU does not downregulate clearance of Aβ plaques by human U937 monocytic cells. Representative figures quantified in Figure 6c of clearance of hippocampal Aβ plaques (green) by U937 cells following incubation on serial sections of unfixed brain from 5XFAD mice in the presence of CLU or desCLU. Immunofluorescent staining of hippocampal Aβ plaques following 48h incubation with medium only but no cells (*left*) or with U937 cells + desCLU (*middle*) or with U937 cells + CLU (*right*). Small figure magnifications of regions of interest: A: x10; B: x20. Scale bars: 100 μm in I and A; and 50 μm in B. n = 10 replications

SUPPLEMENTARY FIGURE 6D

Interaction analyses for effect of CD33 gene expression X CLU gene expression on AD neuropathology traits in subjects from ROSMAP

Trait ~ CD33 gene expression + CLU gene expression + CD33 gene expression x CLU gene expression						
Test the interaction effect of CD33 gene expression x CLU gene expression on AD traits						
n=1092 subjects with gene expression in ROSMAP DLPFC						
Trait	variable	b	se	T	p	
pathological AD	CD33:CLU	-0.33784	0.200907	-1.68157	0.092651	
amyloid	CD33:CLU	-0.16951	0.109142	-1.55314	0.120682	
Arteriolosclerosis	CD33:CLU	0.227958	0.201958	1.128739	0.259008	
Cerebral amyloid angiopathy	CD33:CLU	-0.23336	0.211005	-1.10594	0.268751	
cognitive decline	CD33:CLU	0.01946	0.009501	2.048207	0.040795	
Cerebral atherosclerosis	CD33:CLU	0.139864	0.205417	0.680882	0.495946	
AD dementia	CD33:CLU	-0.4019	0.249055	-1.61372	0.106588	
dementia with lewy bodies	CD33:CLU	-0.71774	0.267301	-2.68512	0.00725	
Lewy bodies in neocortex	CD33:CLU	-0.75779	0.321843	-2.35453	0.018546	
Global AD pathology burden	CD33:CLU	-0.1321	0.059436	-2.22262	0.026446	
hippocampal sclerosis	CD33:CLU	-0.64932	0.3707	-1.75161	0.07984	
residual cognition	CD33:CLU	0.135469	0.09775	1.385872	0.16611	
tangles	CD33:CLU	-0.17213	0.131703	-1.30692	0.191517	
TDP-43 pathology	CD33:CLU	-0.07292	0.229708	-0.31745	0.750899	

SUPPLEMENTARY FIGURE 6E

Causal mediation of analyses for effect of CD33 gene or CLU gene exposure and CLU or CD33 mediator on AD neuropathology traits in subjects from ROSMAP

Causal Mediation Analysis

Exposure > Mediator > AD trait

1. CD33 -> CLU -> AD trait
2. CLU -> CD33 -> AD trait

Test whether mediator mediates the association between exposure and AD trait

n=1092 subjects with gene expression in ROSMAP DLPFC

Exposure	Mediator	Outcome	Effect	b	CI.L	CI.U	p
CD33	CLU	pathological AD	ACME	0.00061	-0.00501	0.00675	0.852
CD33	CLU	clinical AD	ACME	-5.07E-05	-0.00711	0.00769	0.988
CD33	CLU	amyloid	ACME	-0.00656	-0.01917	0.004657	0.27
CD33	CLU	tau	ACME	-0.00372	-0.02118	0.013113	0.688
CLU	CD33	pathological AD	ACME	0.010372	-0.00193	0.024887	0.112
CLU	CD33	clinical AD	ACME	0.016469	0.000454	0.03912	0.044
CLU	CD33	amyloid	ACME	0.036036	0.008041	0.070395	0.006
CLU	CD33	tau	ACME	0.025604	-0.01301	0.067861	0.176

SUPPLEMENTARY FIGURE 6F

eQTL analyses for effect of CLU SNPs on CD33 gene expression in subjects from ROSMAP

CD33 gene expression ~ CLU SNP + PC1 + PC2 + PC3

Test for CLU SNP effect on CD33 gene expression

n=986 subjects with gene expression and eQTL in ROSMAP

Result: One significant eQTL

SNP	b	se	t	p
rs10503814_T	-0.143374337	0.391388006	-0.366322767	0.715245105
rs9331950_A	0.249254062	0.143745747	1.733992598	0.087385452
rs9331949_C	0.223364351	0.60604381	0.368561393	0.713583092
rs9331947_G	-1.205710452	0.41653032	-2.894652306	0.005077918
rs9331942_G	0.223364351	0.60604381	0.368561393	0.713583092
rs3087554_C	-0.060739249	0.166472848	-0.364859794	0.716332
rs2279590_T	-0.136145574	0.127383626	-1.068783945	0.288891902
rs9331931_C	0.244479661	0.151676426	1.611850085	0.111559727
rs9331930_G	0.244479661	0.151676426	1.611850085	0.111559727
rs60056423_A	0.244479661	0.151676426	1.611850085	0.111559727
rs66969288_A	0.244479661	0.151676426	1.611850085	0.111559727
rs7812347_A	0.244479661	0.151676426	1.611850085	0.111559727
rs4732729_A	-0.135860763	0.14390816	-0.944079635	0.34842307
rs28541694_G	0.244479661	0.151676426	1.611850085	0.111559727
rs9331916_T	-0.010820865	0.148786167	-0.072727629	0.942233273
rs7982_A	-0.087349313	0.130835243	-0.66762832	0.506597773
rs9331908_T	-0.108768682	0.144436892	-0.753053328	0.45397962
rs9331905_C	0.244479661	0.151676426	1.611850085	0.111559727
rs11136000_T	-0.087349313	0.130835243	-0.66762832	0.506597773
rs4236673_A	-0.087349313	0.130835243	-0.66762832	0.506597773
rs11787077_T	-0.087349313	0.130835243	-0.66762832	0.506597773
rs1532276_T	-0.083278409	0.132315946	-0.629390576	0.53117184
rs1532277_T	-0.083278409	0.132315946	-0.629390576	0.53117184
rs1532278_T	-0.021155271	0.131553403	-0.160811279	0.87271194
rs867232_G	0.252606899	0.156808881	1.610922147	0.111762204
rs867231_C	0.242702789	0.15678716	1.547976181	0.12620272
rs9331896_C	-0.074715397	0.13043925	-0.572798425	0.568643106
rs2070926_C	-0.074715397	0.13043925	-0.572798425	0.568643106
rs867230_C	-0.06854476	0.132575919	-0.517022704	0.606793705
rs9331888_G	-0.073956958	0.14390857	-0.51391629	0.608952463
rs17515931_T	0.256567731	0.157284189	1.631236636	0.107396791
rs34109053_A	0.256567731	0.157284189	1.631236636	0.107396791
rs73231005_G	0.251148644	0.155275867	1.617435144	0.110347297

SUPPLEMENTARY FIGURE 6G

eQTL analyses for effect of CLU SNPs on CD33 gene expression in subjects from ROSMAP

CLU gene expression ~ CD33 SNP + PC1 + PC2 + PC3
Test for CD33 SNP effect on CLU gene expression
n=986 subjects with gene expression and eQTL in ROSMAP
Result: no significant eQTL

SNP	b	se	t	p
rs273634_T	-0.06565	0.061965	-1.05945	0.293088
rs273635_G	-0.00284	0.064255	-0.0442	0.964875
rs1566576_T	-0.06565	0.061965	-1.05945	0.293088
rs116623489_T	0.003629	0.360034	0.010078	0.991988
rs1710403_A	-0.00284	0.064255	-0.0442	0.964875
rs12609179_G	-0.06565	0.061965	-1.05945	0.293088
rs12609559_A	0.003629	0.360034	0.010078	0.991988
rs273637_G	-0.06565	0.061965	-1.05945	0.293088
rs273638_G	-0.06565	0.061965	-1.05945	0.293088
rs273639_G	-0.06565	0.061965	-1.05945	0.293088
rs200656_T	-0.00284	0.064255	-0.0442	0.964875
rs273640_T	-0.06565	0.061965	-1.05945	0.293088
rs1399837_C	-0.06565	0.061965	-1.05945	0.293088
rs3826656_G	-0.00284	0.064255	-0.0442	0.964875
rs1710398_C	-0.06565	0.061965	-1.05945	0.293088
rs1697553_G	-0.06565	0.061965	-1.05945	0.293088
rs117613660_A	0.003629	0.360034	0.010078	0.991988
rs3865444_A	0.063093	0.05959	1.058782	0.293391
rs2459141_C	-0.06565	0.061965	-1.05945	0.293088
rs117255828_T	0.003629	0.360034	0.010078	0.991988
rs12459419_T	0.063093	0.05959	1.058782	0.293391
rs2455069_G	-0.06565	0.061965	-1.05945	0.293088
rs7245846_A	0.063711	0.059828	1.06491	0.290629
rs74796228_A	0.003629	0.360034	0.010078	0.991988
rs33978622_C	0.063093	0.05959	1.058782	0.293391
rs73932887_A	0.003629	0.360034	0.010078	0.991988
rs34813869_G	0.063711	0.059828	1.06491	0.290629
rs73932888_C	0.003629	0.360034	0.010078	0.991988
rs1354106_G	0.063711	0.059828	1.06491	0.290629
rs35112940_A	0.034417	0.06956	0.494787	0.622322
rs61736475_C	0.003629	0.360034	0.010078	0.991988
rs10409348_A	0.054114	0.061206	0.884119	0.379702
rs1399839_A	-0.06216	0.059184	-1.05035	0.297222
rs273653_G	-0.06216	0.059184	-1.05035	0.297222
rs78347527_A	0.227497	0.179858	1.264865	0.210175
rs273652_C	-0.05551	0.057047	-0.97309	0.333909
rs1803254_C	-0.00688	0.103566	-0.0664	0.947248
rs8103085_T	-0.00688	0.103566	-0.0664	0.947248
rs1697531_G	-0.05551	0.057047	-0.97309	0.333909
rs1034521_G	-0.01284	0.101179	-0.12686	0.89942
rs12461354_A	-0.01284	0.101179	-0.12686	0.89942
rs76248157_T	-0.01284	0.101179	-0.12686	0.89942
rs73612300_C	-0.01284	0.101179	-0.12686	0.89942
rs273651_A	-0.06216	0.059184	-1.05035	0.297222
rs169275_T	-0.05266	0.056636	-0.92982	0.355704
rs273650_A	-0.06216	0.059184	-1.05035	0.297222
rs1566578_T	-0.01284	0.101179	-0.12686	0.89942
rs145323276_C	0.123174	0.116943	1.053281	0.295887
rs11084061_A	-0.01284	0.101179	-0.12686	0.89942
rs273649_C	-0.05266	0.056636	-0.92982	0.355704
rs11084062_C	-0.01284	0.101179	-0.12686	0.89942
rs12461282_C	-0.01284	0.101179	-0.12686	0.89942
rs273648_A	-0.05266	0.056636	-0.92982	0.355704
rs11084063_T	-0.01284	0.101179	-0.12686	0.89942
rs989504_T	-0.01284	0.101179	-0.12686	0.89942
rs273647_C	-0.06216	0.059184	-1.05035	0.297222
rs989502_A	-0.01284	0.101179	-0.12686	0.89942
rs989505_A	-0.01284	0.101179	-0.12686	0.89942
rs1319675_A	-0.01284	0.101179	-0.12686	0.89942
rs273646_T	-0.05266	0.056636	-0.92982	0.355704
rs273645_T	-0.05266	0.056636	-0.92982	0.355704
rs7253829_C	-0.01284	0.101179	-0.12686	0.89942
rs7253831_A	-0.01284	0.101179	-0.12686	0.89942
rs17801843_T	-0.01284	0.101179	-0.12686	0.89942
rs273644_A	-0.05266	0.056636	-0.92982	0.355704
rs9676731_C	-0.01284	0.101179	-0.12686	0.89942
rs273643_T	-0.06326	0.05822	-1.08664	0.280976
rs273642_A	-0.06739	0.059927	-1.1246	0.264654
rs273641_C	-0.06326	0.05822	-1.08664	0.280976
rs7256372_T	-0.00084	0.10394	-0.00811	0.993552
rs7256512_T	-0.00084	0.10394	-0.00811	0.993552
rs71358823_G	-0.01114	0.254009	-0.04386	0.965144
rs10402797_C	-0.00084	0.10394	-0.00811	0.993552
rs1501447_G	-0.00084	0.10394	-0.00811	0.993552
rs1501448_T	-0.00084	0.10394	-0.00811	0.993552
rs166653_G	-0.06326	0.05822	-1.08664	0.280976
rs273619_T	-0.06326	0.05822	-1.08664	0.280976
rs1501449_T	-0.00084	0.10394	-0.00811	0.993552

SUPPLEMENTAL TABLE 7

Statistical information including exact p values

Figure	Analysis	N	Statistical Value	Comparison	Exact P-value	Mean +- SEM
**2h**	Ordinary One way ANOVA with Sidak's multiple comparisons	3--4		CD33M donor/acceptor vs CD335X donor/acceptor		
				CD33M donor/acceptor vs CD33m donor/acceptor		
				CD33M donor/acceptor vs CD33M donor only		
				CD33M5x donor/acceptor vs CD33M donor only		
				CD33m donor/acceptor vs CD33M donor only		
**3a**	Two-sided, unpaired Student's t-test	6	Df = 10	CLU vs DesCLU	5.81×10^{-9}	CLU: 1.17 ± 0.033<br>DesCLU: 0.14 ± .047
**3b**	ANOVA w Tukey's multiple comparisons	6	F = 87.14<br>P = 1.59 x 10⁻10	M/M vs. m/m	1.371E-10	M/M: 0.913 ± 0.039<br>m/m: 0.039 ± 0.017<br>M/m: 0.553 ± 0.062<br>Total: 0.181 ± 0.009
				M/M vs. M/m	4.667E-05	
				M/M vs. total	4.798E-08	
				m/m vs. M/m	4.098E-07	
**3c**	Unpaired t-test	5	Df = 8	M/M vs 5X/5X	3.132×10^{-4}	M/M: 0.48 ± 0.034<br>5X/5X: 0.03 ± 0.02
**4b**	Spearman R	29	r = -0.74	Distance from plaque vs # of PLA positive signals	4.70×10^{-6}	
**4c**	Welch's T-test	3	n/a	AD vs Control	0.002	Control: 0.04 ± 0.007<br>AD:0.06 ± 0.029
**4d**	Welch's T-Test	3	t=1.941, df=3.939	CD33 bound CLU in CLU vs CLU + AB treated HEK293 cells	0.1253	CLU: 1.145 ± 0.09043<br>CLU + AB: 1.379 ± 0.07980
**4e**	Welch's T-Test	3	t=0.1331, df=9.174	CD33 bound CLU in CLU vs CLU + AB treated U937 Cells	0.9	CLU: 1.042 ± 0.06016<br>CLU + AB: 1.056 ± 0.08200

SUPPLEMENTAL TABLE 7

Statistical information including exact p values

**5ai**	Ordinary One-Way ANOVA with Tukey's Multiple Comparisons test	3	F = 27.09<br>P = 0.00015282843990	Control vs AB	0.997787819	Control: 1 ± 0.0<br>AB: 1.27 ± 0.3969<br>CLU: 6.019 ± 1.480<br>AB + CLU: 12.87 ± 1.485
				Control vs CLU	0.041998653	
				Control vs AB +CLU	0.000226956	
				AB vs CLU	0.054015435	
				AB vs AB + CLU	0.000267207	
				CLU vs AB +CLU	0.00821862	
**5aii**	Ordinary One-Way ANOVA with Tukey's Multiple Comparisons test	3	F = 35.61<br>P = 0.0000563	Control vs AB	0.846623914	Control: 1 ± 0.0<br>AB: 1.318 ± 0.2291<br>CLU: 2.504 ± 0.1330<br>AB + CLU:4.644 ± 0.4855
				Control vs CLU	0.020516252	
				Control vs AB +CLU	6.67357E-05	
				AB vs CLU	0.063572324	
				AB vs AB + CLU	0.000129854	
				CLU vs AB +CLU	0.00264097	
**5b**	Ordinary one way ANOVA with Tukey's Multiple	N = 3	F = 26.15<br>P = 0.00000076726	control vs. rCLU	0.062321205	rCLU: 5.17 ± 0.5636<br>rCLU + AB: 10.81 ± 0.8733<br>pCLU: 6.820 ± 0.5596<br>pCLU + AB: 11.99 ± 1.980<br>desCLU: 1.365 ± 0.4374<br>desCLU + AB: 1.480 ± 0.3206
				control vs. rCLU+Abeta	3.22402E-05	
				control vs. pCLU	0.005856401	
				control vs. pCLU+Abeta	8.61456E-06	
				control vs. DeClu	0.999933621	
				control vs. DeClu+Abeta	0.999674428	
				rCLU vs. rCLU+Abeta	0.007510933	
				pCLU vs. pCLU+Abeta	0.014805825	
				DeClu vs. DeClu+Abeta	0.99999993	

SUPPLEMENTAL TABLE 7

Statistical information including exact p values

**5c**	Ordinary One way ANOVA with Sidak's multiple comparisons	N = 4	F = 25.35 P = 0.000000000000106	M/M control vs. Abeta	0.866719262	M/M control: 1.000 ± 0.0 M/M AB: 1.770 ± 0.3636 M/M CLU: 4.020 ± 0.6866 M/M AB +CLU: 6.174 ± 0.4444 m/m control: 0.1502 ± 0.05288 m/m AB: 0.1710 ± 0.04380 m/m CLU: 0.1743 ± 0.03503 m/m AB + CLU: 0.2907 ± 0.03751 M/m control: 0.8398 ± 0.1073 M/m AB: 0.8673 ± 0.1303 M/m CLU: 2.087 ± 0.5298 M/m AB + CLU: 3.323 ± 0.7528
				M/M control vs. CLU	2.09815E-05	
				M/M control vs. Abeta+CLU	1.40136E-10	
				M/M CLU vs. Abeta+CLU	0.002988315	
				m/m control vs. Abeta	>0.999999999999999	
				m/m control vs. CLU	>0.999999999999999	
				m/m control vs. Abeta+CLU	0.999999994	
				m/m CLU vs. Abeta+CLU	0.999999999	
				M/m control vs. Abeta	>0.999999999999999	
				M/m control vs. CLU	0.254241795	
				M/m control vs. Abeta+CLU	0.000467347	
				M/m CLU vs. Abeta+CLU	0.265251347	
**5c + 5X**	Ordinary one way ANOVA with Sidak's multiple comparisons	N = 4	F = 0.5950 P = 0.63	control vs. a beta	0.998895766	5X Control: 1.0 ± 0.0 5X AB: 1.175 ± 0.1540 5X CLU: 1.084 ± 0.2021 5X AB + CLU: 1.513 ± 0.5255
				control vs. clu	0.999984594	
				control vs. a beta + CLU	0.803297006	
				a beta vs. clu	0.999974539	
				a beta vs. a beta + CLU	0.965288321	
				clu vs. a beta + CLU	0.900022802	

SUPPLEMENTAL TABLE 7

Statistical information including exact p values

**5d**	Ordinary One way ANOVA with Sidak's multiple comparisons	N = 3	F = 22.16 P = 6.24107E-10	control vs. Abeta	0.999950953	M/M Control: 1.0 ± 0.0<br>M/M AB: 1.313 ± 0.3024<br>M/M CLU: 3.750 ± 0.8513<br>M/M AB + CLU: 5.748 ± 0.6049<br><br>m/m control: 0.1120 ± 0.02852<br>m/m AB: 0.1138 ± 0.02852<br>m/m CLU: 0.1389 ± 0.01229<br>m/m CLU + AB: 0.1491 ± 0.01992<br><br>M/m control: 0.7506 ± 0.1691<br>M/m AB: 0.8848 ± 0.1905<br>M/m CLU: 2.216 ± 0.5092<br>M/m AB + CLU: 3.038 ± 0.4427
				control vs. CLU	0.000333293	
				control vs. Abeta+CLU	5.36026E-08	
				CLU vs. Abeta+CLU	0.011821572	
				control vs. Abeta	>0.999999999999999	
				control vs. CLU	>0.999999999999999	
				control vs. Abeta+CLU	0.999999994	
				CLU vs. Abeta+CLU	>0.999999999999999	
				control vs. Abeta	0.999999997	
				control vs. CLU	0.125780637	
				control vs. Abeta+CLU	0.003007615	
				CLU vs. Abeta+CLU	0.827174384	
**5d + 5X**	Ordinary One way ANOVA with Sidak's multiple comparisons	N = 4	F = 0.1713 P = 0.91	control vs. Abeta	0.99997169	5X control: 1.0 ± 0.0<br>5X AB: 1.033 ± 0.3372<br>5X CLU: 1.134 ± 0.2455<br>5X AB + CLU: 1.222 ± 0.2511
				control vs. CLU	0.992744298	
				control vs. A Beta + CLU	0.954289007	
				CLU vs. A Beta + CLU	0.99853692	
**5e**	Paired T-test	N = 10	t=2.981, df=9	Clusterin vs. "No treatment"	1.540E-02	No treatment: 32052 ± 3109<br>Clusterin: 36479 ± 2320
**5f**	RM one way ANOVA w Tukey's Multiple Comparisons	N =10	F: 10.07<br>P value = 0.0025	Untreated vs. CLU (60 nM)	0.0024	Untreated 1.000 ± 0.000<br>CLU (60 nM) 0.8546 ± 0.03000<br>deCLU (60 nM) 1.021 ± 0.04431
				Untreated vs. deCLU (60 nM)	0.8856	
				CLU (60 nM) vs. deCLU (60 n M)	0.0124	

SUPPLEMENTAL TABLE 7

Statistical information including exact p values

**6ai**	Unpaired t-test	N= 10	t=3.016, df=18	CC vs AA	0.0074	CC: 0.9208 ± 0.05755<br>AA: 0.6817 ± 0.05453
**6aii**	Unpaired t-test	N = 10	t=3.283, df=18	CC vs AA	0.0041	CC: 0.776 ± 0.0744<br>AA: 0.489 ± 0.046
**6aiii**	Unpaired t-test	N= 10	t=2.157, df=18	CC vs AA	0.0448	CC: 0.819 ± 0.0356<br>AA: 0.711 ± 0.0348
**6bi**	One Way ANOVA with Tukey's multiple comparisons	N = 8	F = 15.85 P = 0.0000893936068	Medium vs Cells	5.70633E-05	Medium: 1.0 ± 0.038<br>Cells: 0.6319 ± 0.04161<br>Cells + CLU: 0.8385 ± 0.05914
				Medium vs Cells + CLU	0.057730164	
				Cells vs Cells + CLU	0.016986993	
**6bii**	One way ANOVA with Tukey's multiple comparisons	N = 7	F = 14.64 P = 0.000141778	Medium vs Cells	9.13917E-05	Medium: 1 ± 0.07989<br>Cells: 0.5353 ± 0.03527<br>Cells + CLU: 0.7915 ± 0.05706
				Medium vs Cells + CLU	0.069264097	
**6ci**	One way ANOVA with Tukey's multiple comparisons	N = 7	F = 9.598 P = 0.000865493	Medium vs desCLU	0.000664543	Medium: 1.0 ± 0.02399<br>DesCLU: 0.7112 ± 0.04066<br>CLU: 0.9142 ± 0.06798
				Medium vs CLU	0.395783843	
				desCLU vs CLU	0.017919483	
**6cii**	One way ANOVA with Tukey's multiple comparisons	N = 8	F = 9.57 P= 0.0015533710308	Medium vs desCLU	0.001298788	Medium: 1.0 ± 0.07524<br>DesCLU: 0.5937 ± 0.02240<br>CLU: 0.8932 ± 0.08340
				Medium vs CLU	0.523523546	
				desCLU vs CLU	0.020011574	

SOURCE IMAGES FOR SUPPLEMENTARY FIGURES

SUPPLEMENTARY DATA FIGURE 2A – source blot for Supplementary Figure 2A

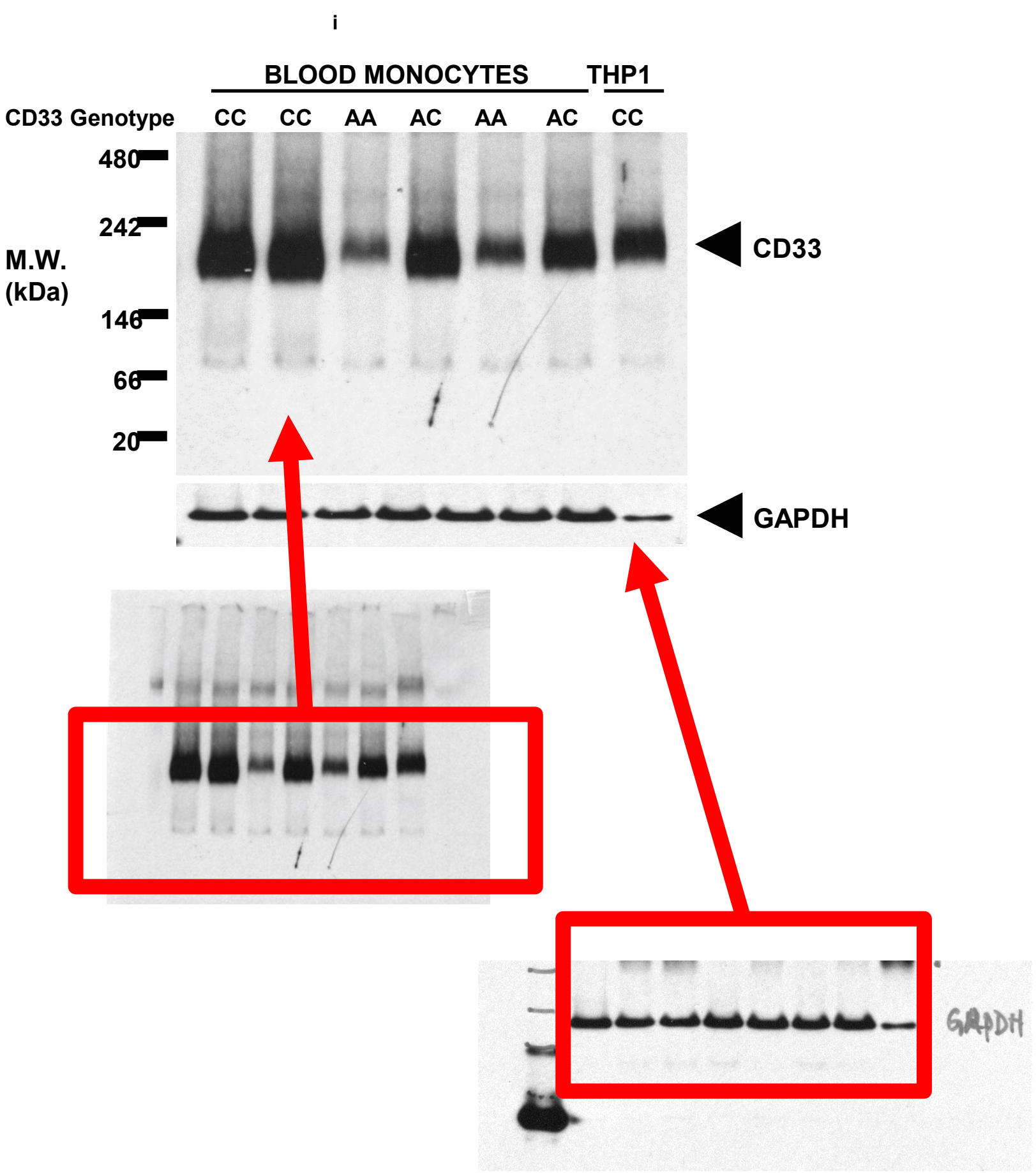

SUPPLEMENTARY FIGURE 2B – source blot for Supplementary Figure 2B

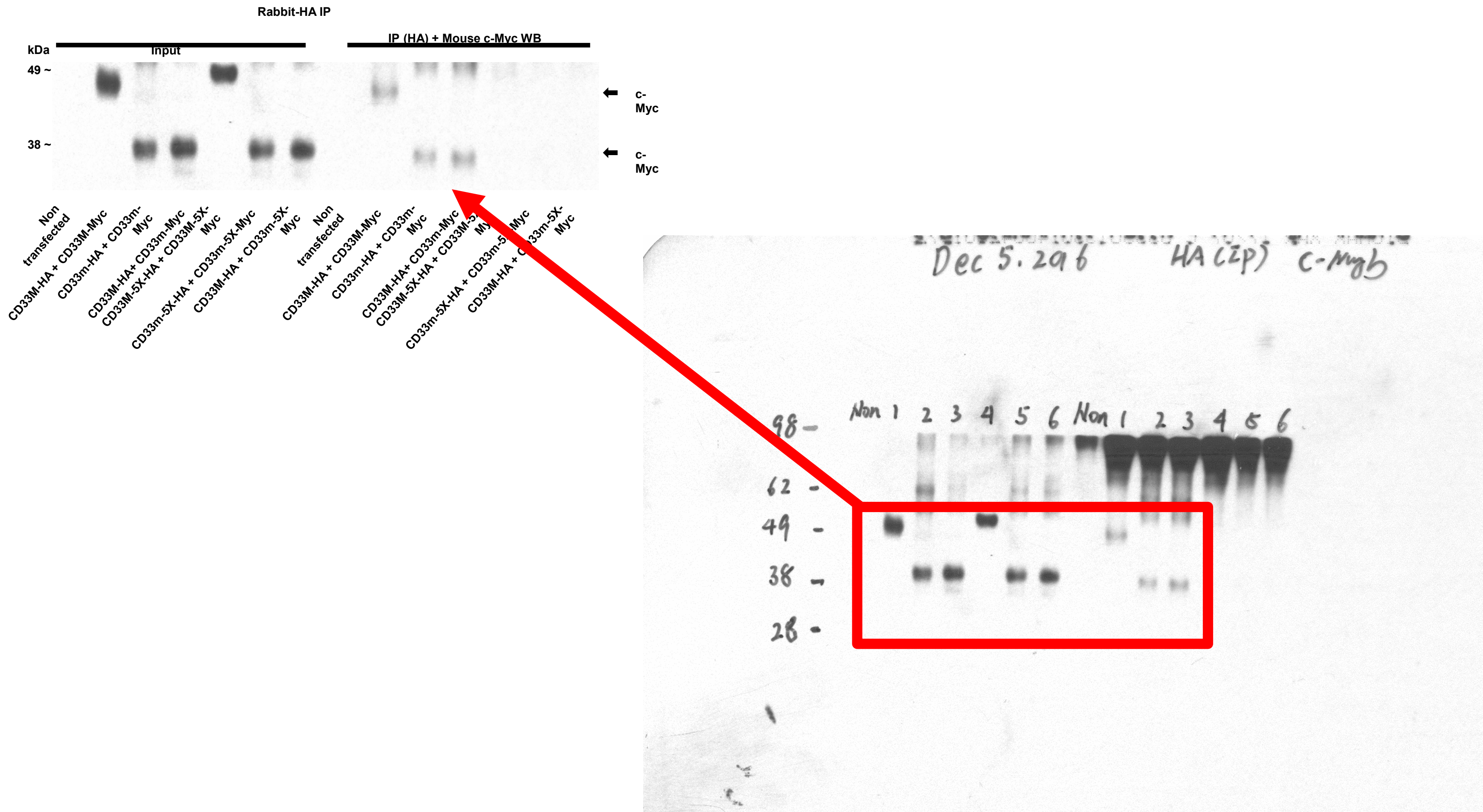

SUPPLEMENTARY FIGURE 3A – source blot for Supplementary Figure 3A

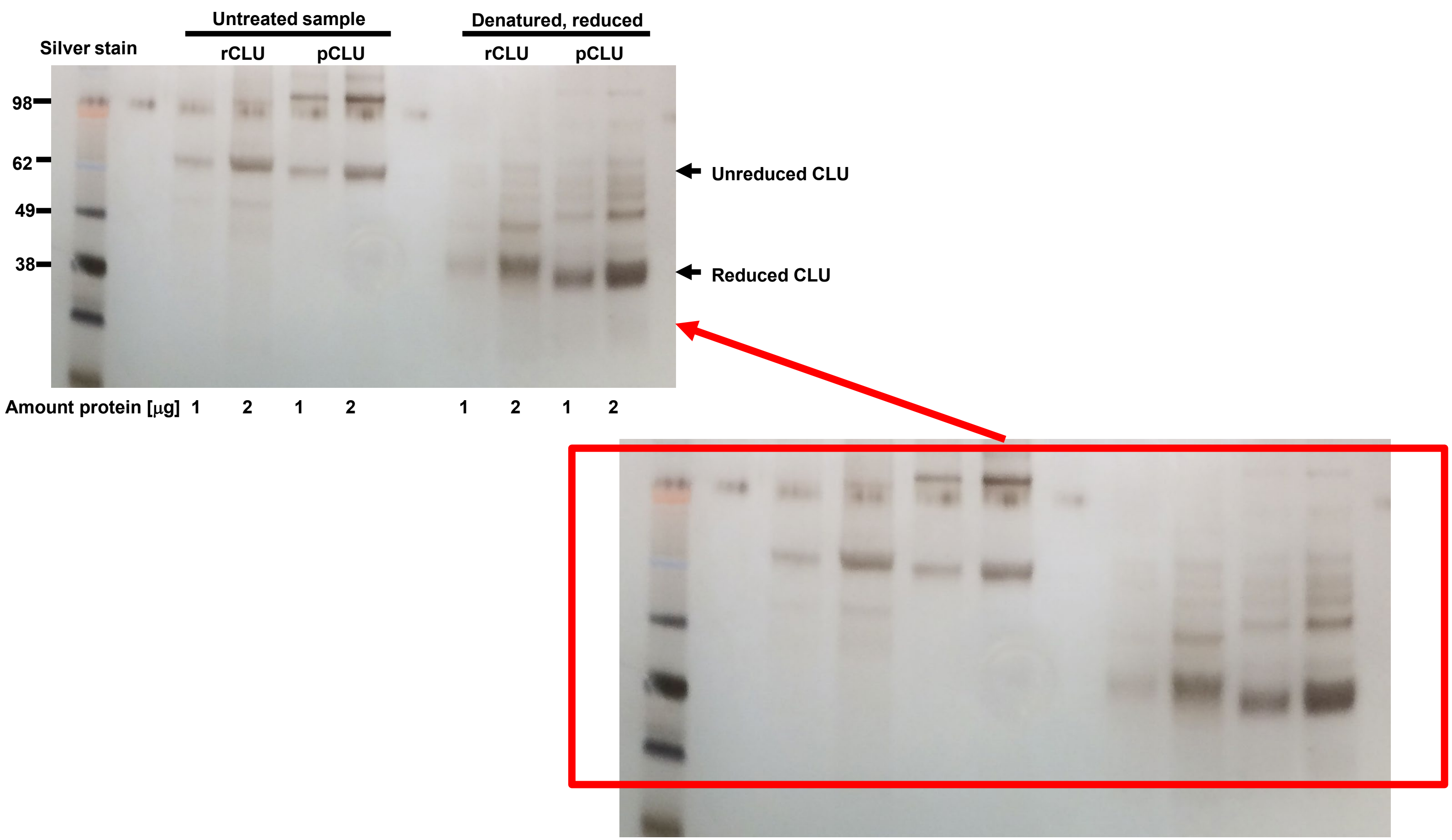

SUPPLEMENTARY FIGURE 3B – source blots for Supplementary Figure 3B

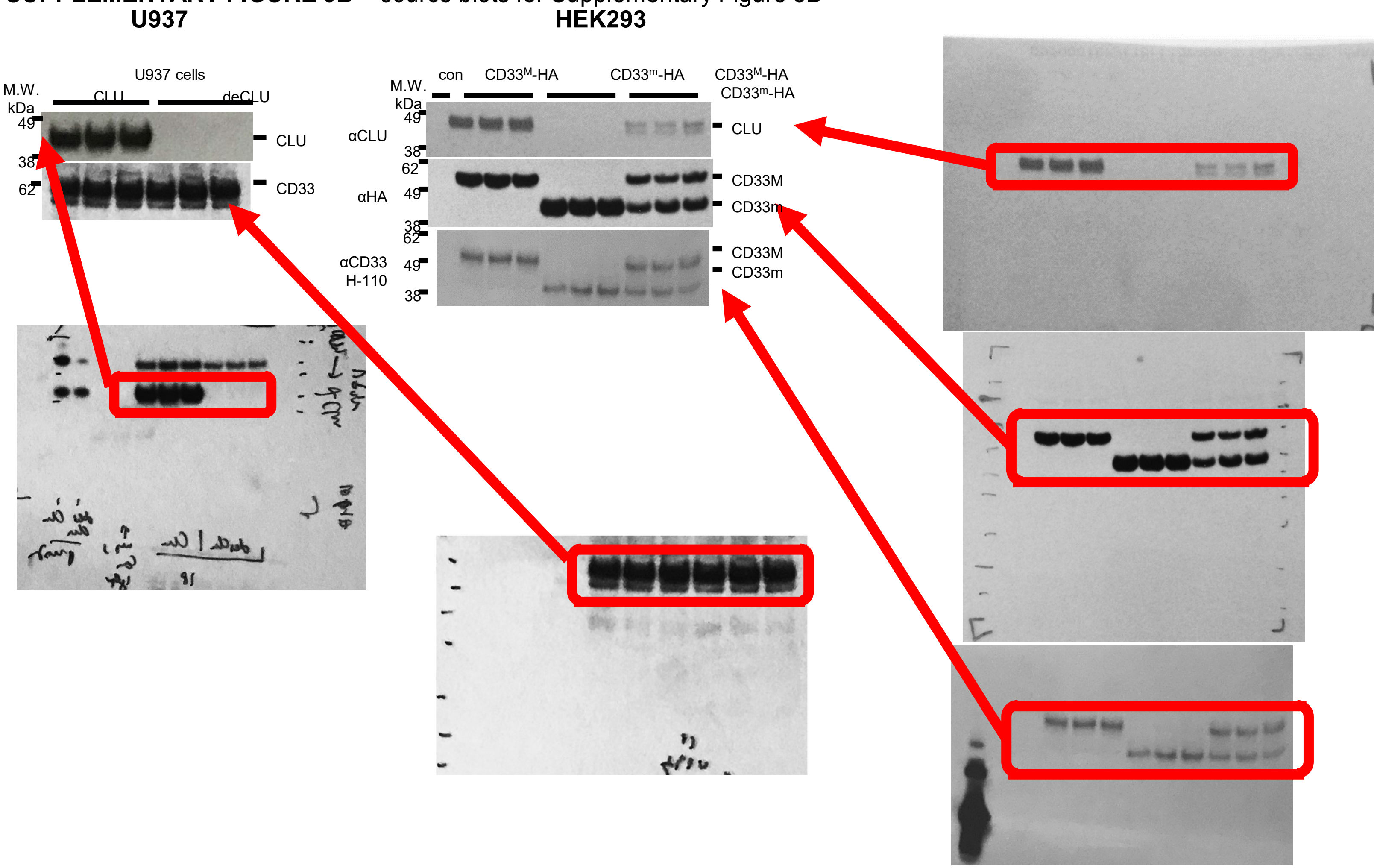

SUPPLEMENTARY FIGURE 3C – source blot for Supplementary Figure 3C

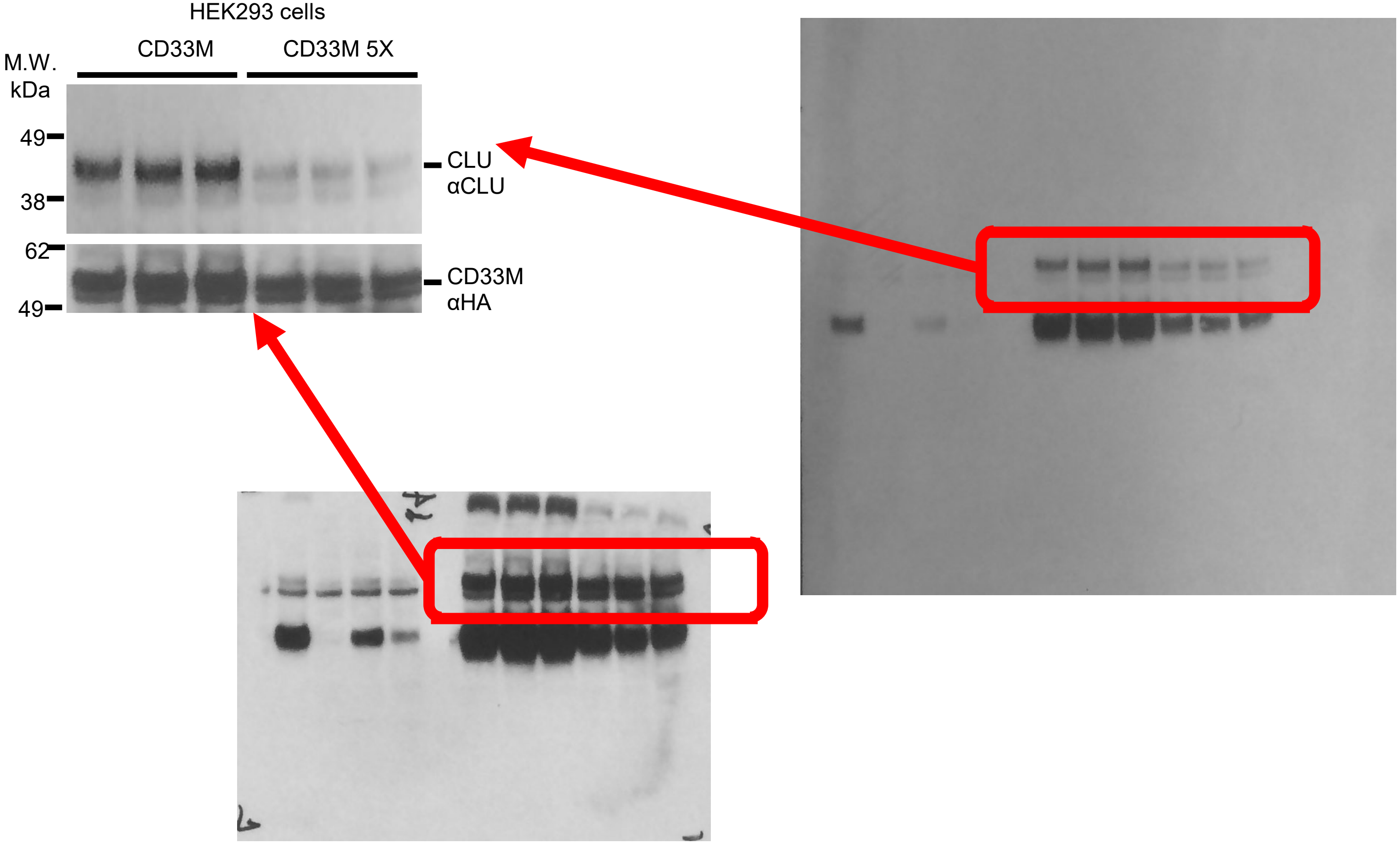

SUPPLEMENTARY FIGURE 4A – source blots for Supplementary Figure 4A

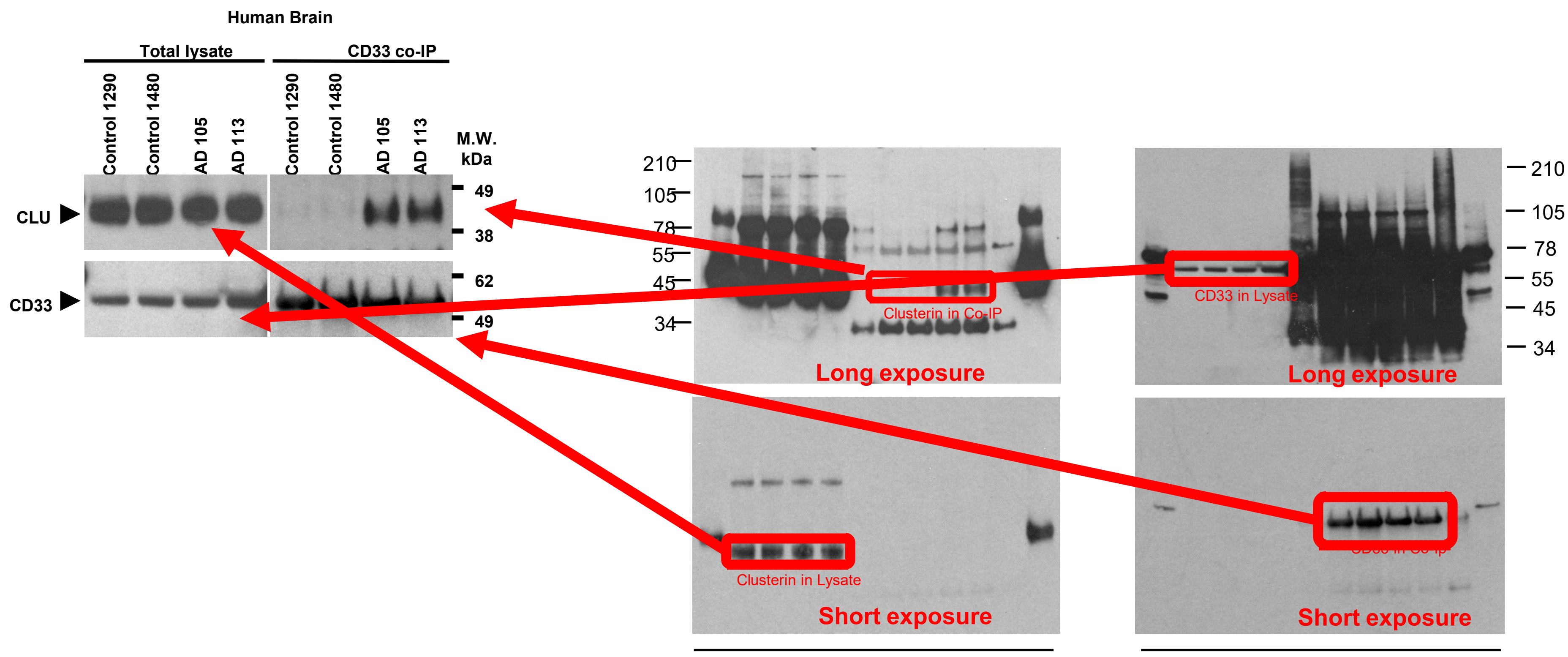

SUPPLEMENTARY FIGURE 4B – source blots for Supplementary Figure 4B

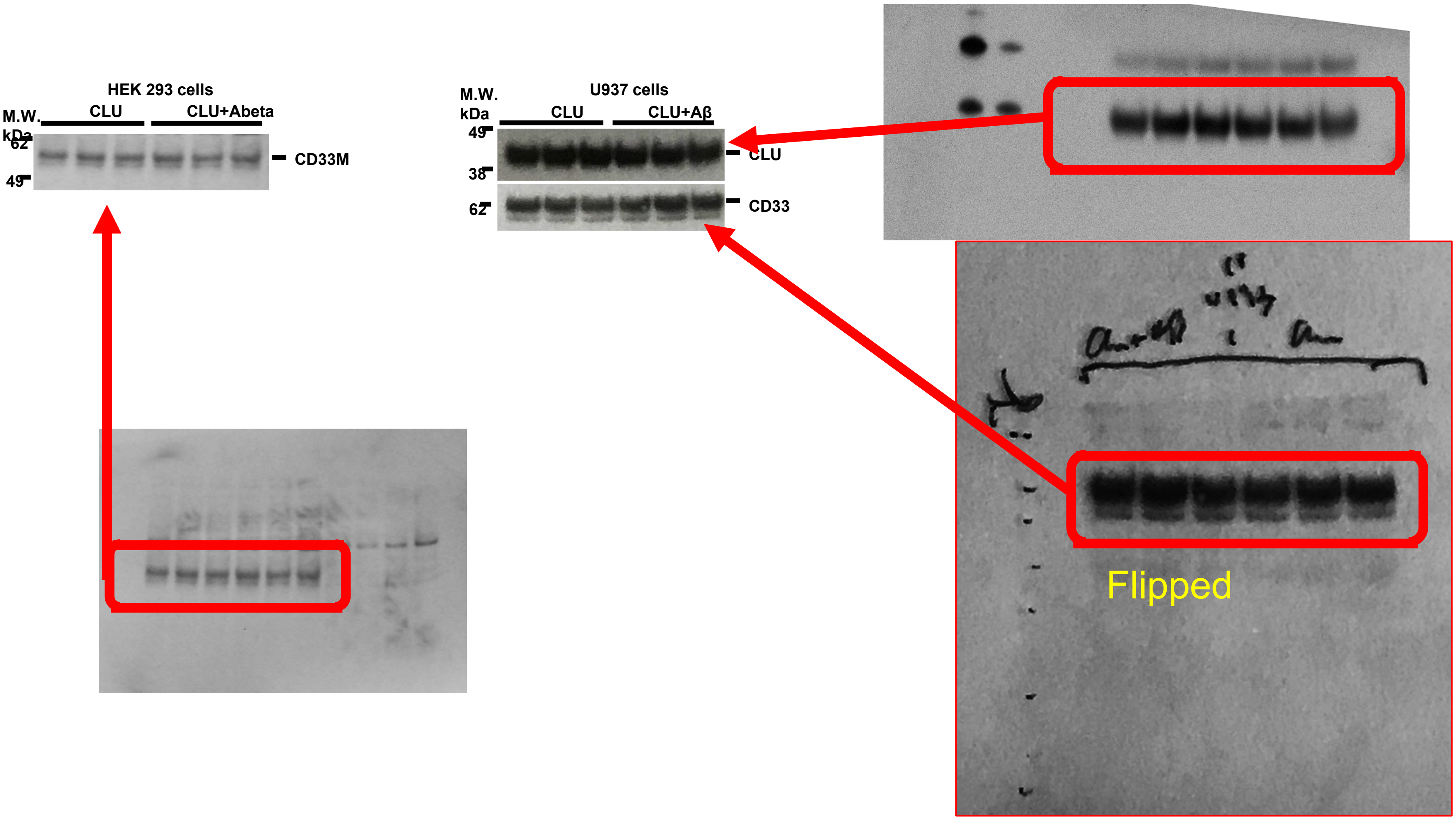

SUPPLEMENTARY FIGURE 5A – source blots for Supplementary Figure 5A

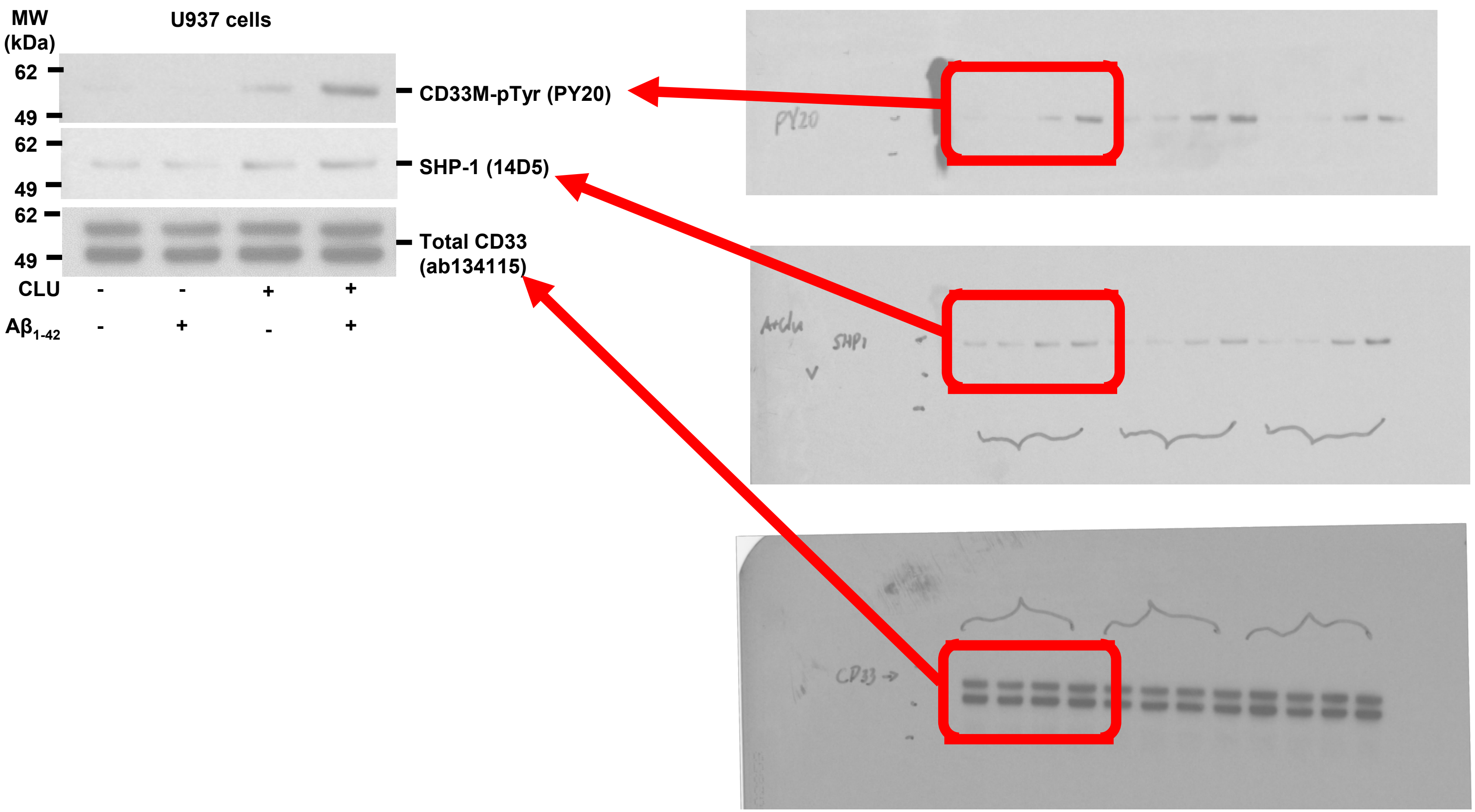

SUPPLEMENTARY FIGURE 5B – source blots for Supplementary Figure 5B

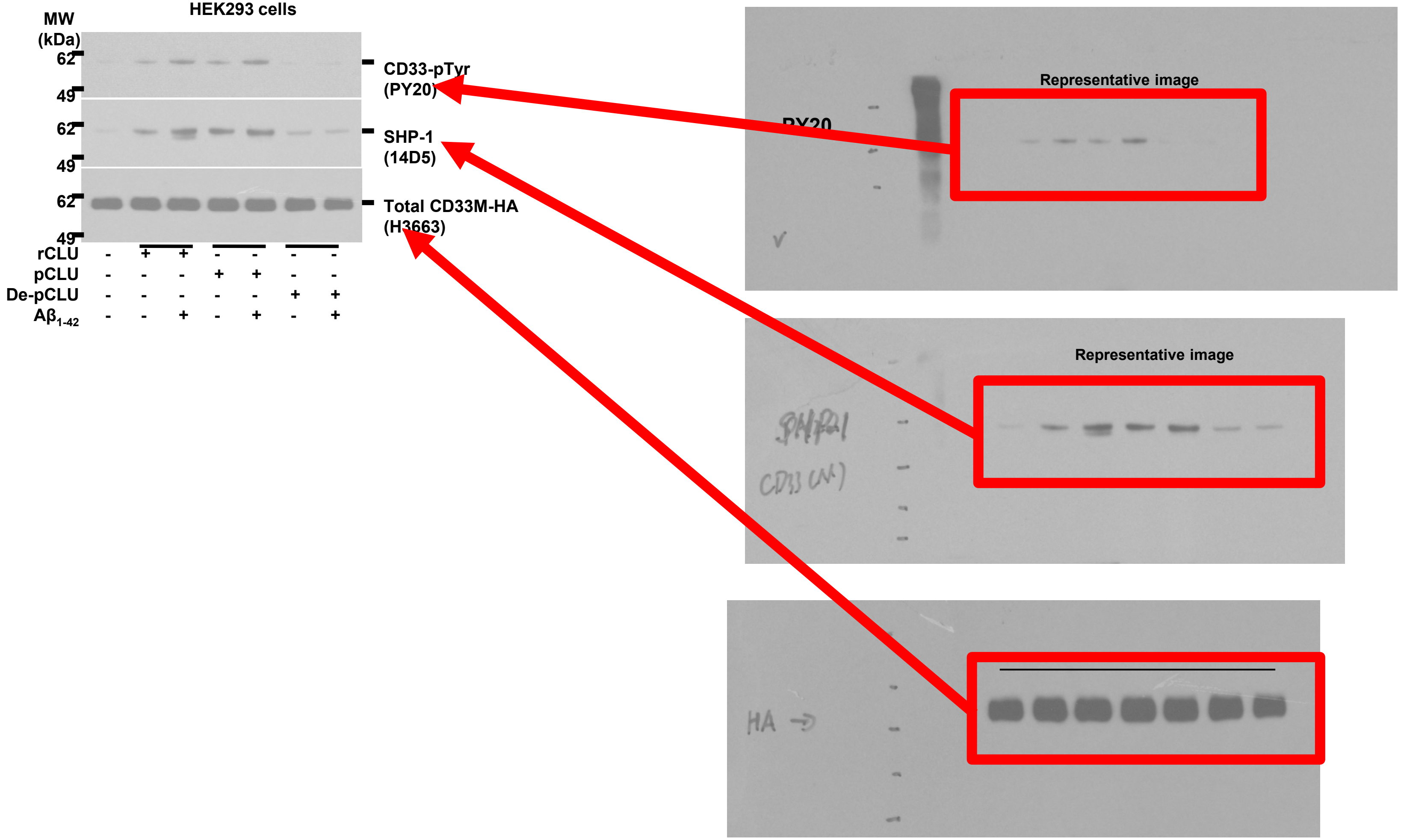

SUPPLEMENTARY DATA FIGURE 5C,D – source blots for Supplementary Figures 5C,D

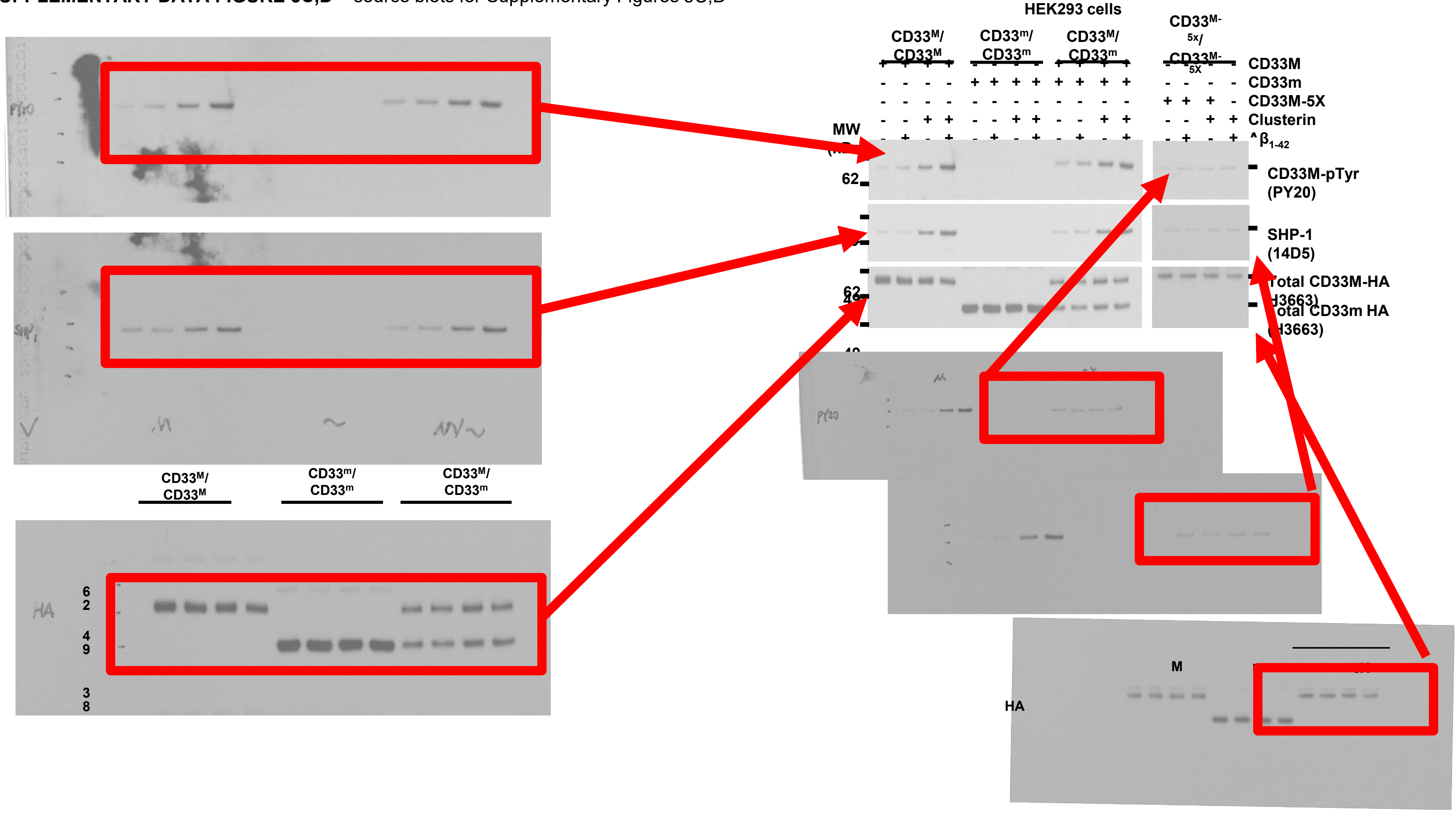

SUPPLEMENTARY FIGURE 5F – source blots for Supplementary Figure 5F

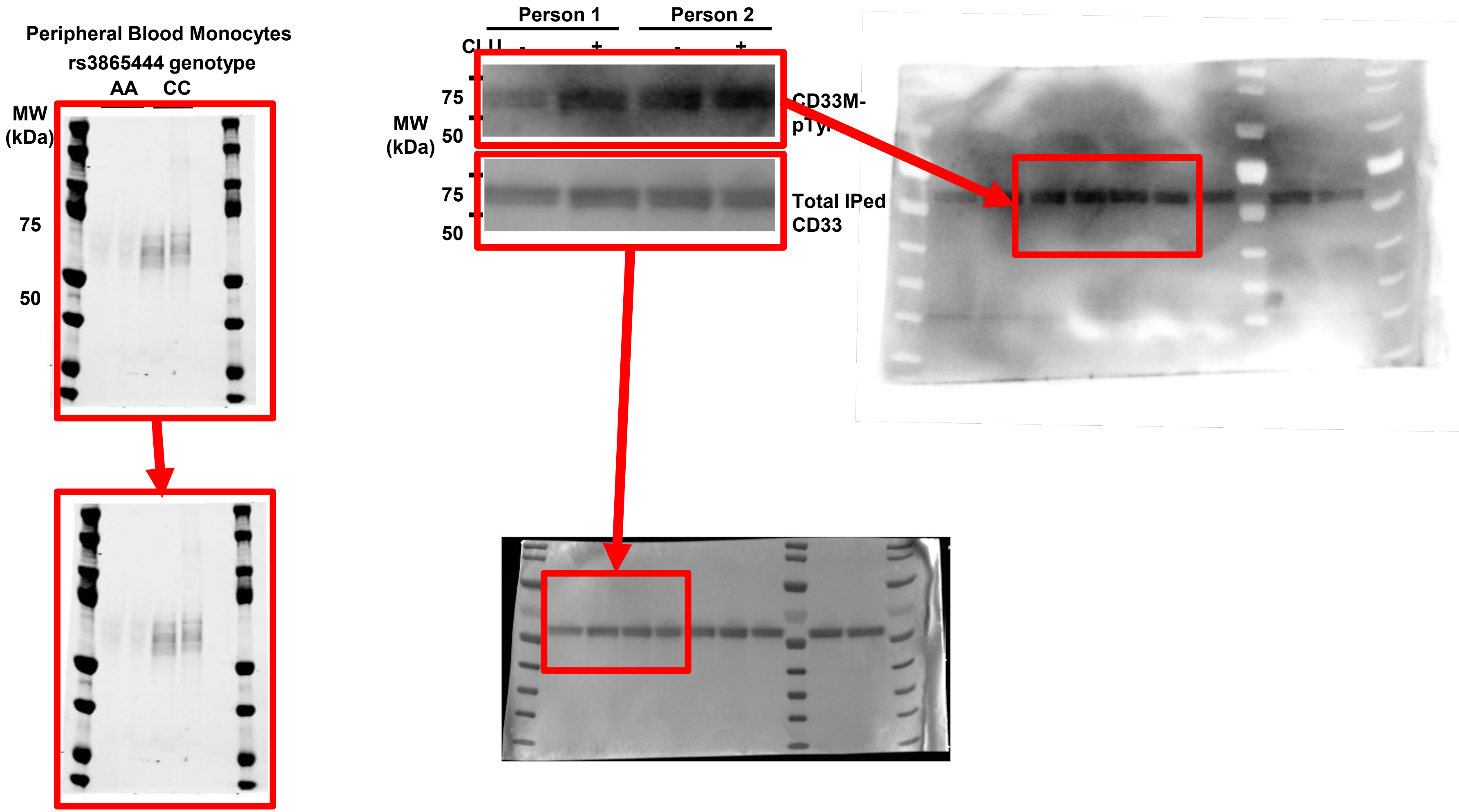

Supplement: Supplementary file 1 [file 41467_2026_75140_MOESM1_ESM.pdf]
