# Supplementary material for: CD33 and clusterin interact biophysically and genetically to modulate Alzheimer risk
Source: Nat Commun. 2026 Jul 17;17:8983. doi: 10.1038/s41467-026-75140-3 (PMC13507242; doi:10.1038/s41467-026-75140-3)
Supplement: Supplementary file 2 [file 41467_2026_75140_MOESM2_ESM.pdf]

nature portfolio

Corresponding author(s): Wesley Asher, Elizabeth Bradshaw; Peter St George-Hyslop

Last updated by author(s): June 03 2026

Reporting Summary

Nature Portfolio wishes to improve the reproducibility of the work that we publish. This form provides structure for consistency and transparency in reporting. For further information on Nature Portfolio policies, see our Editorial Policies and the Editorial Policy Checklist.

Statistics

For all statistical analyses, confirm that the following items are present in the figure legend, table legend, main text, or Methods section.

n/a	Confirmed	
☐	☒	The exact sample size (*n*) for each experimental group/condition, given as a discrete number and unit of measurement
☐	☒	A statement on whether measurements were taken from distinct samples or whether the same sample was measured repeatedly
☐	☒	The statistical test(s) used AND whether they are one- or two-sided<br>*Only common tests should be described solely by name; describe more complex techniques in the Methods section.*
☒	☐	A description of all covariates tested
☐	☒	A description of any assumptions or corrections, such as tests of normality and adjustment for multiple comparisons
☐	☒	A full description of the statistical parameters including central tendency (e.g. means) or other basic estimates (e.g. regression coefficient) AND variation (e.g. standard deviation) or associated estimates of uncertainty (e.g. confidence intervals)
☐	☒	For null hypothesis testing, the test statistic (e.g. *F*, *t*, *r*) with confidence intervals, effect sizes, degrees of freedom and *P* value noted<br>*Give P values as exact values whenever suitable.*
☒	☐	For Bayesian analysis, information on the choice of priors and Markov chain Monte Carlo settings
☒	☐	For hierarchical and complex designs, identification of the appropriate level for tests and full reporting of outcomes
☒	☐	Estimates of effect sizes (e.g. Cohen's *d*, Pearson's *r*), indicating how they were calculated

Our web collection on statistics for biologists contains articles on many of the points above.

Software and code

Policy information about availability of computer code

Data collection	Standard, publicly available software programs were used for analysis of: x-ray crystal data and protein folding data (Xia2 with the 3dii protocol, XDS, XSCALE, MATTPROB, PHASER, SCULPTOR, COOT, PHENIX, phenix.refine PDBeFold), analytical ultra-centrifugation (Sedfit, HeteroAnalysis), binding affinity (ASTRA), circular dichroism (Spectra Manager), flow cytometry (FlowJo V10) and autoradiogram band intensities (ImageJ) were used. TIRF and smFRET imaging data was acquired with MetaMorph Premier for Olympus (v. 7.7.11.0, Molecular Devices, LLC). No proprietary software packages were employed.
Data analysis	Standard, publicly available software programs were used for analysis of: x-ray crystal data and protein folding data (Xia2 with the 3dii protocol, XDS, XSCALE, MATTPROB, PHASER, SCULPTOR, COOT, PHENIX, phenix.refine PDBeFold), analytical ultra-centrifugation (Sedfit, HeteroAnalysis), binding affinity (ASTRA), circular dichroism (Spectra Manager), flow cytometry (FlowJo V10) and autoradiogram band intensities (ImageJ) were used. No proprietary software packages were employed. The smCellFRET data analysis pipeline is freely available for academic use. The software and updated versions can be downloaded at http://innovation.columbia.edu/technologies/CU15268. The surface density of single molecules and cell region of interest were determined using the open-source TrackMate software (v. 4.0.0.; https://github.com/fiji/TrackMate/releases/tag/ TrackMate_-4.0.0) in Fiji/ImageJ (v. 1.49m). Statistical comparisons for all single-molecule data were carried out using GraphPad Prism (Version 10.3.1 (509) GraphPad Software LLC)

For manuscripts utilizing custom algorithms or software that are central to the research but not yet described in published literature, software must be made available to editors and reviewers. We strongly encourage code deposition in a community repository (e.g. GitHub). See the Nature Portfolio guidelines for submitting code & software for further information.

Data

Policy information about availability of data

All manuscripts must include a data availability statement. This statement should provide the following information, where applicable:

- Accession codes, unique identifiers, or web links for publicly available datasets
- A description of any restrictions on data availability
- For clinical datasets or third party data, please ensure that the statement adheres to our policy

X-ray crystal structure data have been deposited in PDB under accession codes 5IHB (Apo CD33), 5J06 (3-SL complex) and 5J0B (6-SL complex). cDNA and cells are available without restriction from public repositories or the authors. The raw single-molecule image data generated and analyzed that support the findings of this study are available from WBA upon request. These image data are not deposited in a public database because of their large file sizes. Numerical data for graphs in the main figures and supplementary data figures are included and in the excel spreadsheets in the on-line data files. Machine data for the ultracentrifugation is included as separate machine-readable files. Source images for image data in the main and supplementary data figures are included in the online data files bearing labels indicating their content. ROSMAP data is available through application to the ROSMAP data coordinator at the Rush Alzheimer Disease Center (DAB).

Human research participants

Policy information about studies involving human research participants and Sex and Gender in Research.

Reporting on sex and gender	Both genders were used in a non-biased way. There is no separate analysis for each gender because this was not the focus of the current analyses. The sex and gender data is available from ROSMAP and from the New York Blood Center (NYBC)
Population characteristics	The age of the blood and brain tissue donor varies between 20 years to 70 years old. The population comprises of diverse ethnicity including African American, Hispanics, Whites, and Asians.
Recruitment	De-identifed human blood was obtained from the New York Blood Center (NYBC https://www.nybc.org) without any selection bias for age, race, sex, ethnicity, etc. Monocytes were obtained from venous blood samples from healthy blood donors at the Brigham and Women's Hospital Blood Bank. The age and sex of the subjects is unknown. The genotype of the rs8356444 SNP was determined experimentally. The subjects are healthy donors. Anomymised, neuropathologically well characterisedpost mortem brain tissue was obtained from the Maritime Brain Bank https://www.mtbtdatabase.dal.ca . Anonymised omic's data was obtained from the ROSMAP study https://dss.niagads.org/cohorts/religious-orders-study-memory-and-aging-project-rosmap/
Ethics oversight	Columbia University Irving Medical Center and Brigham and Women's Hospital for blood cells and Rush University Medical Center

Note that full information on the approval of the study protocol must also be provided in the manuscript.

Field-specific reporting

Please select the one below that is the best fit for your research. If you are not sure, read the appropriate sections before making your selection.

[x] Life sciences [ ] Behavioural & social sciences [ ] Ecological, evolutionary & environmental sciences

For a reference copy of the document with all sections, see nature.com/documents/nr-reporting-summary-flat.pdf

Life sciences study design

All studies must disclose on these points even when the disclosure is negative.

Sample size	Sample sizes were determined empirically by the experimental conditions, which typically were cell lines or protein isolates with 3 different genotype/phenotype conditions (CD33M/CD33M; CD33M/CD33m; and CD33m/CD33m).
Data exclusions	No data items were excluded
Replication	The data in all figures was reliable and reproduced as described below:<br>FIGURE 1: 1a-1e and related items in Supplementary Data Figure 1, and Figure 2a,bb and associated data in Supplmentary data Figure 2: The three atomic structures (apo-CD33, CD33 with 3'- and 6'-siallylactose ligand was solved once, but were cross correlate with each other.<br>FIGURE 2: 2c - k and D panels i-ii and related items in Supplementary Data Figure 2: Flow cytometry experiments were conducted by two different personnel on different instruments with different cells, with the same results (n = 3 independent biological replications (each with 6 technical replicates) with similar results). smFRET experiments: total number of cells (n cells) are indicated and were collected over N = 3 or 4 independent experiments. 2i-k were conducted in HEK293 cells with n = 3 independent replicas. The iPSC iMGs were generated from the same undifferentated iPSC cell, with N > 2 independent differentiations.<br>FIGURE 3: 3a-c and related items in Supplementary Data Figure 3 reflect N = 6 independent biological experiments (fully independent cell lines and immunoprecipitations). 3d-i-iv show representative images from N = 4 technical replications from the same CD33 protein preparation. Figure 3e underwent N= 4 independent technical replications.<br>FIGURE 4: 4a, b represent N=2 independent biological replications (i.e. 2 different AD affected brain samples). N= 29 independent

measurements were taken. 4c underwent N=3 independent biological replications (3 AD brains and 3 control brains). 4d N=3 independent biological replications.Figure 4e N=6 independent biological replications.
FIGURE 5: 5ai-ii and related items in Supplementary Data Figure 5 underwent N = 3 independent biological experiments (fully independent U937 cell lines and immunoprecipitations). 5b underwent N = 3 independent biological experiments (fully independent HEK293 cell lines and immunoprecipitations). 5c and 5d; and and related items in Supplementary Data Figure 5A-D underwent N = 3-4 independent biological replications. 5e and 5f;and related items in Supplementary Data Figure 5 underwent N = 10 independent biological replications. 5F of supplementary data underwent N= 3 independent biological replications (3 AA homozygotes and 3 CC homozygotes). Supplementary data Figure 5 underwent N = 3 independent biological replications. N=7 measurements.
FIGURE 6: 6ai-iii underwent N = 10 independent biological replications. 6bi-ii underwent N = 7-8 independent biological replications. 6ci-ii underwent N = 8-10 independent biological replications.

Randomization	Not applicable
Blinding	Data were collected from x-ray crystallographic experiments, co-immunoprecipitation, binding affinity and phosphorylation experiments using unbiased, quantitative machine based methods. Blinding was therefore not necessary.

Reporting for specific materials, systems and methods

We require information from authors about some types of materials, experimental systems and methods used in many studies. Here, indicate whether each material, system or method listed is relevant to your study. If you are not sure if a list item applies to your research, read the appropriate section before selecting a response.

Materials & experimental systems

n/a	Involved in the study	
☐	☒	Antibodies
☐	☒	Eukaryotic cell lines
☒	☐	Palaeontology and archaeology
☐	☒	Animals and other organisms
☐	☒	Clinical data
☒	☐	Dual use research of concern

Methods

n/a	Involved in the study	
☒	☐	ChIP-seq
☐	☒	Flow cytometry
☒	☐	MRI-based neuroimaging

Antibodies

Antibodies used	The following antibodies were used: Myc: (Cell Signalling, 22776S); HA: (rabbit polyclonal, Abcam, ab9110; mouse monoclonal clone HA-7, Sigma-Aldrich, H9658; mouse monoclonal, Sigma-Aldrich, H3663); CD33: (Santa Cruz, 28811; rabbit monoclonal, Abcam, Ab134115; mouse monoclonal, Millipore Sigma, 133M-1, (PWS44); mouse monoclonal EMD Millipore, CBL163 (Clone WM53); eBioscience, 12-0339-41; eBioscience, 12-0338-42 (WM53-PE); Biolegend, 94835 (A16121H - not yet commercially released); eBioscience, #12-0339-41 (HIM3-4-PE); rabbit polyclonal, Sigma Aldrich HPA035832); SHP1: (Thermo Fisher Invitrogen 14D5); Anti-phosphotyrosine (PY20, Abcam Ab10321). CLU: (The anti-clusterin monoclonal antibodies were obtained from Mark Wilson (G7 and 41D hybridoma); mouse monoclonal, Santa Cruz sc-32264, clone 78E); Iba1: (rabbit polyclonal, Wako, 01919741,); Aß: (mouse monoclonal, Creative Biolabs, TAB0792CLV, clone NAB61); Isotype control: rat IgG2a (Biolegend #405416, AF647, 1:80 for flow cytometry); mouse IgG1k PE (eBioscience #12-4714-82, 1:80 for flow cytometry); mouse IgGκ (from Wilson Lab for PLA); rat IgG2ak (Biolegend #400501, for flow cytometry); mouse IgG1 (EMD Millipore MABC002, for CBL163 WM53 control). All antibodies have been used in previously published work by other groups.
Validation	All antibodies have been previously validated in published work by other groups.

Eukaryotic cell lines

Policy information about cell lines and Sex and Gender in Research

Cell line source(s)	HEK293T and HEK293F (ATCC human embryonic kidney cell line; validated by morphology and expression of human proteins; tested for mycoplasma by Sigma). THP-1 (ATCC human monocyte cell line; validated by morphology and expression of human proteins; tested for mycoplasma by ATCC).U937 (ATCC human monocyte cell line; validated by morphology and expression of human proteins; tested for mycoplasma by ATCC). The FA0000010 (FA10) hiPSC line was generously provided by Barbara Corneo. The Flp-In-CHO cell line was obtained from Thermo Fisher Scientific (Cat.#: R758-07; parent line CHO-Kl, ATCC # CCL-61). The LEx FITR CHO cell line was generated from T-REx-CHO cells obtained from Thermo Fisher Scientific (Cat.# R718-07; parent line CHO-Kl, ATCC # CCL-61). The CHO lines stable expressing CD33 proteins were generated from LEx FITR CHO cells described above.
Authentication	Human cell lines (HEK293T, HEK293F, THP1, U937 and FA10) were validated by morphology and by the endogenous expression of appropriate human proteins. The parental CHO cell lines used in this study were authenticated by manufacturer.
Mycoplasma contamination	Human cell lines (HEK293T, HEK293F, THP1, U937 and FA10) were validated by morphology and by the endogenous expression of appropriate human proteins. The parental LEx FITR CHO cells tested negative for mycoplasma contamination.

Commonly misidentified lines (See ICLAC register)	Not applicable

Animals and other research organisms

Policy information about studies involving animals; ARRIVE guidelines recommended for reporting animal research, and Sex and Gender in Research

Laboratory animals	Frozen post-mortem brain tissue slices were used in Figure 6 and were obtained from female animals from the well-documented 5XFAD transgenic mouse line.
Wild animals	Not applicable
Reporting on sex	Post mortem frozen brain was only used from female animals to ensure a consistent and abundant source of amyloid plaques
Field-collected samples	Not applicable
Ethics oversight	The acquisition and usage of these tissues was approved by the Tel Aviv University Research Ethics Board.

Note that full information on the approval of the study protocol must also be provided in the manuscript.

Clinical data

Policy information about clinical studies

All manuscripts should comply with the ICMJE guidelines for publication of clinical research and a completed CONSORT checklist must be included with all submissions.

Clinical trial registration	Not applicable
Study protocol	Not applicable
Data collection	De-identifed human blood was obtained from the New York Blood Center (NYBC) without any selection bias for age, race, sex, ethnicity, etc. Monocytes were obtained from venous blood samples from healthy blood donors at the Brigham and Women's Hospital Blood Bank. The age and sex of the subjects is unknown. The genotype of the rs8356444 SNP was determined experimentally. The subjects are healthy donors.<br><br>Human postmortem brain tissue samples were either taken from the Canadian Brain Tissue Bank or the Barcelona Brain Tissue Bank. The acquisition and usage of those samples adhered to the standards of the University of Toronto Research Ethics Board.
Outcomes	Cells were used to assess CD33 and SHP-1 phosphorylation and Abeta uptake. No clinical outcomes were assessed

Flow Cytometry

Plots

Confirm that:

- [x] The axis labels state the marker and fluorochrome used (e.g. CD4-FITC).
- [x] The axis scales are clearly visible. Include numbers along axes only for bottom left plot of group (a 'group' is an analysis of identical markers).
- [x] All plots are contour plots with outliers or pseudocolor plots.
- [x] A numerical value for number of cells or percentage (with statistics) is provided.

Methodology

Sample preparation	HEK 293T cells were plated into 6-well plates one day before transfection. Cells were transfected with GFP (1μg) plus CD33M (2 μg), CD33m (2 μg) or CD33M+CD33m (1 μg+1 μg) plasmids using Lipofectamine3000. Twenty-four hours after transfection, cells were collected by trypsinization and then washed twice with 1xPBS. Cells then were stained by Viability Dye eFluor450 (65-0863, eBioscience) at 4°C for 30 min. After washing, cells were resuspended with 1x PBS and 1 million cells per test were then stained with primary antibodies (WM53-PE and A16121H for the double staining; HIM3-4-PE for the single staining) for 30 min at 4°C. After washed with 1x PBS 3 times, double stained cells were stained with secondary antibody (anti-ratIgG2a-AF647) for 30 min at 4°C. After washed 3 times, cells were transferred to flow cytometry test tubes and data were acquired by BD FACS LSRII with corresponding compensation controls.
Instrument	BD FACS LSRII
Software	FlowJo v10
Cell population abundance	Pure HEK 293T cells were used for the experiment. Cells were analyzed and not sorted.

Gating strategy

FSC-A v SSC-A were used to gate on cells; FSC-W v SSC-A were used to remove doublets; Dead cells were excluded as eFluor450 positive; GFP negative cells were excluded as non-transfected cells; Cells with very high GFP signals that could not be compensated properly were excluded (Gate were created by the transfected/unstained control); only the GFP positive, live single cells were analyzed.

☐ Tick this box to confirm that a figure exemplifying the gating strategy is provided in the Supplementary Information.
